# Filling the BINs of life: Report of an amphibian and reptile survey of the Tanintharyi (Tenasserim) Region of Myanmar, with DNA barcode data

**DOI:** 10.3897/zookeys.757.24453

**Published:** 2018-05-10

**Authors:** Daniel G. Mulcahy, Justin L. Lee, Aryeh H. Miller, Mia Chand, Myint Kyaw Thura, George R. Zug

**Affiliations:** 1 Global Genome Initiative, National Museum of Natural History, Smithsonian Institution, 10th & Constitution Ave., Washington, DC, 20013 USA; 2 College of Computer, Mathematical and Natural Sciences, University of Maryland, College Park Maryland, 20742 USA; 3 Department of Biology, University of North Carolina Asheville, Asheville, NC 28804 USA; 4 College of William & Mary, Williamsburg, Virginia, 23187 USA; 5 Myanmar Environment Sustainable Conservation (MESC), Yangon, Myanmar; 6 Department of Vertebrate Zoology, National Museum of Natural History, Smithsonian Institution, Washington, DC, 20013 USA

**Keywords:** Anura, biodiversity, Gymnophiona, Thai-Malay Peninsula, natural history, Southeast Asia, species diversity, Squamata, Testudines, Thailand

## Abstract

Despite threats of species extinctions, taxonomic crises, and technological advances in genomics and natural history database informatics, we are still distant from cataloguing all of the species of life on earth. Amphibians and reptiles are no exceptions; in fact new species are described nearly every day and many species face possible extinction. The number of described species continues to climb as new areas of the world are explored and as species complexes are examined more thoroughly. The use of DNA barcoding provides a mechanism for rapidly estimating the number of species at a given site and has the potential to record all of the species of life on Earth. Though DNA barcoding has its caveats, it can be useful to estimate the number of species in a more systematic and efficient manner, to be followed in combination with more traditional, morphology-based identifications and species descriptions. Herein, we report the results of a voucher-based herpetological expedition to the Tanintharyi (Tenasserim) Region of Myanmar, enhanced with DNA barcode data. Our main surveys took place in the currently proposed Tanintharyi National Park. We combine our results with photographs and observational data from the Chaung-nauk-pyan forest reserve. Additionally, we provide the first checklist of amphibians and reptiles of the region, with species based on the literature and museum. Amphibians, anurans in particular, are one of the most poorly known groups of vertebrates in terms of taxonomy and the number of known species, particularly in Southeast Asia. Our rapid-assessment program combined with DNA barcoding and use of Barcode Index Numbers (BINs) of voucher specimens reveals the depth of taxonomic diversity in the southern Tanintharyi herpetofauna even though only a third of the potential amphibians and reptiles were seen. A total of 51 putative species (one caecilian, 25 frogs, 13 lizards, 10 snakes, and two turtles) were detected, several of which represent potentially undescribed species. Several of these species were detected by DNA barcode data alone. Furthermore, five species were recorded for the first time in Myanmar, two amphibians (Ichthyophis cf. kohtaoensis and *Chalcorana eschatia*) and three snakes (*Ahaetulla mycterizans*, *Boiga dendrophila*, and *Boiga drapiezii*).

## Introduction

Despite advances in technologies, warnings of taxonomic crises, and increased interest in taxonomy (Mallet and Willmott 2003, Tautz et al. 2003), scientists are still trying to provide an accurate measure of global biodiversity in terms of absolute numbers of extant species of life on Earth (e.g. Costello et al. 2013, Caley et al. 2014, Wilson 2017). Amphibians and reptiles are no exceptions to groups with accurate estimates of extant species because new species are described nearly every day and estimates suggest ca. 30% of amphibians (Stuart et al. 2004) and ca. 20% of reptiles (Böhm et al. 2013) may be threatened. The benefits of knowing and understanding global biodiversity are enormous and span fields of human interest from agriculture, pest management, disease control, natural products, conservation, and wildlife management. Many of the discrepancies in estimates are the result of non-statistical calculations (i.e. “simple best guesses”), statistical calculation that contain wide ranges of error (e.g. “+/- three million”), and estimates that do not build on one another, and overlap with previous analyses (Giller 2014). Furthermore, without careful comparisons of known material (voucher specimens), estimates of unknown species may contain significant overlap with currently recognized species (synonymies). Certainly, in this age of genomics and bioinformatics, we have the ability to accurately measure and record global species diversity with resources like the Encyclopedia of Life (EOL), Tree of Life projects (e.g. http://www.tolweb.org/tree/), the Barcode of Life Database (BOLD), GenBank, and taxonomic specific databases such as Amphibian Species of the World 6.0 (ASoW, Frost 2017) and the Reptile Database (Uetz et al. 2018). To confound matters, there have been recent requests to regulate and standardize practices in taxonomy (Garnett and Christidis 2017), which has stirred debate on the theoretical aspects of species and the practicality of regulating ‘taxonomic freedom’ (Raposo et al. 2017). While issues of regulating taxonomic actions remain unresolved, DNA barcoding (Hebert et al. 2003a) offers a standardized mechanism for measuring biodiversity at the species level and a database to manage it (BOLD). However, DNA taxonomy has many caveats and limitations, such as proposed thresholds of percent sequence divergence vary among groups, and it is less effective among recently diverged groups (Lipscomb et al. 2003; Tautz et al. 2003), and we are still a long way from obtaining a complete DNA barcode library of life of Earth. Currently, the BOLD database contains DNA barcodes for approximately 275,000 formally described species of fungi, plants, and animals (http://www.boldsystems.org/index.php, queried 11 February 2018), of the approximately 1.5 million catalogued (only ~18%), and of the ~7 million estimated (< 3%) species (Caley et al. 2014).

The use of DNA barcoding offers a starting point for recording the number of species of life on Earth (Hebert et al. 2003a, 2000b). The concept of a database containing representatives of every species with a common molecular marker, shared among all living organisms (though different for plants and animals) for comparison is attractive. Once a reference library is established, it provides researchers with resources of numerous possibilities, ranging from agriculture, ecological and environmental studies, biodiversity surveys, conservation, food and drug administration, and the prevention of wildlife trafficking. In animals, the DNA barcode is a portion of the mitochondrial DNA gene cytochrome oxidase subunit 1 (COI); different markers are used for plants (chloroplast DNA), fungi, etc. Furthermore, a system of Barcode Index Numbers (BINs) has been developed to assist in specimen identifications, by using several algorithms to compare COI data, combining sequences into operational taxonomic units (OTUs), which likely correspond to biological species (Ratnasingham and Hebert 2013). Investigators can quickly compare COI sequences in a database (BOLD) and rapidly determine whether their samples are unique, or similar to described, or even un-described species, thus eliminating or reducing the number of synonymies in species estimates. For example, if one identifies a species as “sp. A” and if they, or someone else identifies other individuals of the same species and refers to them as “sp. B,” this creates a synonymy. This is why it is important to compare newly acquired material with all available data, and to maintain current usage of place-holding names. For example, a researcher might identify a specimen to be the same species as “sp. A” of another study (e.g. Diechmann et al. 2017). By DNA barcoding newly discovered species, one can quickly verify its degree of difference via the BINs, only if other closely related species are also barcoded. The BINs are automatically generated if the sequences are deposited in BOLD, and new sequences will be placed in existing BINs if within ~2%, or new BINs will be created; discordant BINs (e.g. a single BIN with specimens bearing different names) are flagged and easily identified. However, there are several caveats to consider when evaluating whether a BIN represents a legitimate species, or whether a BIN is discordant because of disagreements on higher-level classification (e.g. constantly changing generic names). Additionally, some wide-ranging, genetically variable species may occupy multiple BINs. A point worth noting is that the BINs are not formally named (i.e., they do not bear species names), thus they provide an objective, standardized measure of comparison for evaluating species boundaries.

Presently, there are approximately 518,000 BINs, representing ~180,000 formally described animal species currently in BOLD. This indicates that there are currently only DNA barcodes for a fraction of formally described animal species (< 10%), and BINs for ~338,000 un-described animal species (granted legitimate species may occupy several BINs, thus reducing the estimated number of BINs of undescribed species). Contributions of DNA barcodes for known taxa (identified to species by traditional morphological characters – and complemented with molecular DNA data) are appreciated and can be provided in terms of “data release papers” (e.g. Zuniga et al. 2017). However, current researchers conducting biodiversity surveys, particularly of poorly known taxonomic groups, and groups also poorly represented by DNA barcode data, are left with the challenge of identifying cryptic species diversity using whatever molecular data is available (e.g. Stuart et al. 2006a). This identification must be done with some level of taxonomic expertise, where the specimens in hand are compared with species descriptions, and sequence data aligned with known reference material (e.g. GenBank). When multiple OTUs, or clades, are discovered among specimens identified with the same name, careful comparisons must be made to the original type descriptions, geographic distributions, and genetic data. As cryptic species are revealed, original descriptions of species and their geographic ranges must be modified to account for current taxonomic understanding. Nevertheless, there is a pressing need for biodiversity surveys in many parts of the world, and especially including groups in taxonomic disarray such as amphibians.

**Figure 1.**
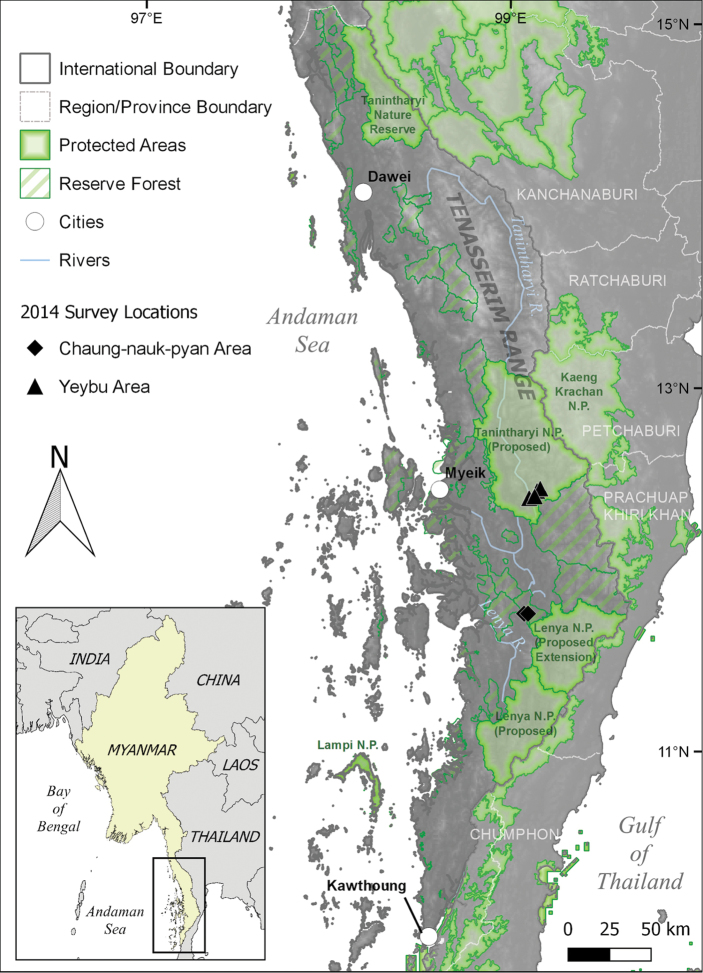
Map of Tanintharyi Region, Myanmar. The Tanintharyi Nature Reserve, north of Dawei, and the Lampi National Park, island northwest of Kawthoung, are officially designated as national parks in Myanmar. The other areas in Myanmar are proposed as national parks (Protected Areas) or being considered for future protection (Reserve Forests). The main survey reported here was conducted in the Yeybu area of the proposed Tanintharyi National Park (triangles: Forest 1–2 and Gardens sites). A shorter survey, with fewer people, was conducted in the Reserve Forest near the Chaung-nauk-pyan area (diamonds: Forest 3 and slash & burnt sites) and is also included in this report. Map provided by Grant M. Connette of the Smithsonian Conservation Biology Institute (SCBI).

Here, we provide an example by incorporating DNA barcode data with biodiversity inventory survey data of amphibians and reptiles collected in a poorly known region of the world, the Tanintharyi Region of Myanmar (the ‘Tenasserim’). This includes one of the most poorly known vertebrate groups in terms of taxonomy – anurans in Southeast Asia. Prior to this study, there were only 1259 anuran species with DNA barcodes in BOLD for the approximately 7727 currently recognized species of anurans known globally (Frost 2017). Specifically, we set out to determine how many species occur at our study site. We surveyed a region within the proposed Tanintharyi National Park near the village of Yeybu (Fig. 1), conducting day and night surveys, collecting representative voucher specimens with tissue samples for genetic analyses. We used DNA barcoding in conjunction with traditional methods to assist in our specimen identifications, not to delimit species (Collins and Cruickshank 2013). In the process, we discovered what likely represent new, undescribed species, ‘species discovery’ (Collins and Cruickshank 2013). We recommend the use of additional data and analyses to formally evaluate, describe, and recognize potential species identified with the barcode data. Here, we combine our survey results with a shorter survey in an area to the south, near the village of Chaung-nauk-pyan (Fig. 1), consisting of reserve forest and a recently slash and burned area, where only photographs and other observations were made. Our results show how the use of DNA barcode data can augment and increase the accuracy of biodiversity inventory surveys and suggest caution should be taken when identifications are made solely on morphological identifications, particularly for some of the more cryptic species complexes of anurans in this region. We offer our protocol and results as a model for others working with groups in taxonomic disarray. Finally, we provide the first comprehensive checklist of amphibians and reptiles of the region (excluding marine species) based on our results, distinguished by DNA barcode verification versus observation only, and other expected species based on museum records and the literature.

## Methods

### The Tanintharyi

Tanintharyi is the southern-most political division of Myanmar, now formally known as the Tanintharyi Region. This region occupies about the southern two-thirds of the former colonial British administrative unit of Tenasserim; the northern portion is now Mon State. Biological surveys of Tanintharyi have been limited in postcolonial times owing to political disagreements and military activities. The last herpetofaunal summary of Tanintharyi is Theobald’s 1868 report. The Myanmar Herpetological Survey (MHS) was permitted access to southernmost Tanintharyi (Kawthaung area) in 2002 and again to north-central Tanintharyi (Dawei area) in 2009 and 2010. Since then political change has allowed broader access. The Tenasserim, or Tanintharyi, contains type localities for at least seven amphibian and seven reptile species. Some of these, referenced near “Moulmein” (= Mawlamyine) are in present-day Mon State, while others, referenced near Dawei, "Mergui" (= Myeik), and the “Valley of the Tenasserim” are in the Tanintharyi Region. Theobald (1868) provided the first and last report of the amphibians and reptiles of the Tenasserim. Other researchers have reported on the occurrence of individual species or sets of species but no single attempt has been made to review the herpetofauna of the entire region. We joined Fauna & Flora International’s biodiversity survey team in June 2014 to provide a preliminary assessment of the amphibian and reptile biodiversity survey in the proposed Tanintharyi National Park, of southern Myanmar. We realize that a rapid assessment survey would sample at best only a quarter to a third of the herpetofauna (Zug 2011) and only the species active in the early monsoon. The timing of this survey emphasizes amphibian species. The details of the sites are presented below in the survey itineraries. We supplement our morphological identifications with DNA barcoding. We provide COI data to build upon the taxonomic representation in BOLD and the Barcode Index Number system (BINs). We also include 16S data that can be directly compared with currently available published sequences in GenBank to provide better molecular identifications of our specimens. Many sequences in GenBank are incorrectly identified, which subsequently pollutes the database, especially so among Southeast Asian anurans. Tracking voucher specimen information can sometimes be difficult or nearly impossible if the information was not appropriately provided or was lost. Therefore, we attempt to exemplify how to efficiently review the taxonomy on a species-group basis, compare specimen morphology to species descriptions, genetic data with GenBank and BOLD records, evaluate those records, and how to interpret proper taxonomic nomenclatural assignments.

Lacking a recent review for the Tanintharyi, we relied on reptile and amphibian checklists and studies of Thailand (Nabhitabhata et al. 2004, Pauwels et al. 2002, Pauwels and Chan-ard 2006, Matsui et al. 2005a, Vogel et al. 2009, Grismer et al. 2010, Chan-ard and Makchai 2011, Ohler et al. 2011, Grismer et al. 2012, Sumontha et al. 2012, Grosjean et al. 2015a, Grismer et al. 2016, 2017, Wood et al. 2017, Pauwels et al. 2000, 2009), reports from other regions in Myanmar (Dowling and Jenner 1988, Zug et al. 2006a, b, 2007), and recent work in the Tanintharyi (e.g. Wilkinson et al. 2012, Connette et al. 2017, Mulcahy et al. 2017; Zug et al. 2017; Lee et al. 2018). An earlier report by Pauwels et al. (2002) for Phang-nga Province, Thailand, was south of the southern tip of Tanintharyi and below the Isthmus of Kra, which is useful for comparison of species that may cross the isthmus and occur in the southernmost Tanintharyi. We compiled a comprehensive species list of amphibians and reptiles documented in the Tanintharyi, either by our collections, observations, or specimens at the California Academy of Sciences (CAS). We also generated some DNA barcode data for specimens previously collected in Myanmar from the National Museum of Natural History, Smithsonian Institution (USNM) and CAS tissue collections. Some of the specimens we barcoded remain in the Myanmar Biodiversity Museum (MBM) in Hlawga National Park, north of Yangon. These specimens have yet to be catalogued at the MBM; therefore, we refer to them as MBM-Collector Number (e.g. MBM-JBS 19825). Comments on the occurrence and biological aspects of single species or groups of related species are included in the individual species accounts below. The Red List Status for each species was taken from www.iucnredlist.org on 9 March 2018.

### Team members

The survey team for the proposed Tanintharyi National Park area comprised Myint Kyaw Thura (ENCA), Daniel G. Mulcahy (NMNH-SI)) and Thaw Zin, for the Reserve Forest area Myint Kyaw Thura and Thaw Zin.

### Survey itineraries and sites

1) Proposed Tanintharyi National Park – Yeybu area (Fig. 1). The survey team traveled to Myeik (12.4359°N, 98.5941°E, 7 m ASL; all latitude and longitude coordinates were taken with WGS84 datum) on 4 June 2014, hired a 4wd vehicle and drove to Tegu, then hired a boat to Yeybu on 5 June. Yeybu village (12.3927°N, 99.1044°E) is about 168 km northeast of Myeik. The village is 500 m east of the Tanintharyi River. On 6 June, the team with porters and cooks walked in and established the first camp (“Forest 1”: 12.4345°N, 99.1442°E, 93 m ASL) alongside Yeybuchaung-ngal (ngal = stream). Our searching for amphibians and reptiles was conducted within a 500 m radius of the camp, principally along the creek and its smaller feeder streams owing to the absence of trails through the dense forest. The team moved upstream to a second camp (“Forest 2”: 12.4478°N, 99.1621°E, 116 m ASL) on 10 June. Exceptionally heavy monsoonal rain on 12 June and rapidly rising stream level forced the team’s return to the eastern edge of Yeybu village, where they used the cook’s house as the third camp (“Gardens”: 12.4039°N, 99.1312°E, 30m ASL) and searched for amphibians and reptiles in this area through the morning of 16 June, and then returned by boat to Tegu, whence by 4wd vehicle to Myeik. The first and second camps were within the evergreen forest. At both sites bamboo was a dominant feature of the vegetation and the canopy was closed, or nearly so. The third survey site was open agricultural land, principally of small gardens, orchards, and numerous small temporary ponds along the floodplain of the Tanintharyi River. Total survey time was 10 days, voucher specimens were taken.

2) Reserve forest – Chaung-nauk-pyan area (Fig. 1). A smaller survey team (over a shorter period) traveled by road from Myeik to the village of Chaung-nauk-pyan on 4 July 2014 and whence by foot the following day to a degraded evergreen forest site, approximately 4.25 km southwest of the village. Surveys at this site (“Forest 3”: 11.7574°N, 99.0730°E, 49m ASL) occurred from 5 July through the morning of 7 July when they shifted to a recently cleared secondary forest site (“Slash & burnt”: 11.7573°N, 99.0945°E, 67m ASL) and searched for amphibians and reptiles for the next 24 hours, returning to Chaung-nauk-pyan on the afternoon of 8 July and returned to Myeik on 10 July. Total survey time was four days. Captured frogs and reptiles were photographed and released at the site of capture.

Collections of amphibians and reptiles were made at four sites: hotel in Myeik (commensal); Yeybuchaung-ngal stream Camp 1 (Forest 1); Yeybuchaung stream Camp 2 (Forest 2); and vicinity of Yeybu village (Gardens). Only observations and photographs were taken at two sites: near the village of Chaung-nauk-pyan in a Reserve Forest (Forest 3) and nearby in a recently cleared site (Slash & burnt). Dates and latitude and longitude coordinates are identified above in the itinerary. The survey protocol was visual searching along Yeybuchang-ngal stream and its smaller side-branches. The stream was searched both during the day and at night (with flash-lights and head lamps). All amphibians were captured by hand; reptiles by hand, rubber-bands, sling-shots (catapults), and snake tongs for large or dangerous species. Transport of specimens from the field to the camp was done in plastic bags for amphibians and cloth bags for reptiles. Unique individuals were usually photographed. All specimens to be retained as vouchers were euthanized following IUCAC protocols, with a drop of 5% benzocaine on the head (amphibians) or into the oral cavity (reptiles). Genomic tissue samples (piece of liver and/or muscle) were taken from all specimens. The genomic samples were harvested prior to formalin preservation; each sample was place in individual 1.5 ml tube with salt-saturated ethylene-diamine-tetracetic acid/ Dimethyl sulfoxide (EDTA/DMSO) buffer for long-term storage and future genetic analyses modified from Seutin et al. (1991) with 25% DMSO instead of 20% (Mulcahy et al. 2016). Specimens were individually tagged with a unique field number and preserved in 10% formalin. Voucher specimens and tissues were deposited at the National Museum of Natural History, Smithsonian Institution (USNM) collection.

### Molecular data

We attempted several rounds of PCR and sequencing for each specimen collected, with the exception of four *Odorrana hosii* Boulenger and three *Ansonia thinthinae* Wilkinson, Sellas, & Vindum. In addition to our samples from our expedition, we DNA barcoded 108 additional specimens of amphibians from previous USNM collections in Myanmar, mostly northern states, to verify if these were the same species that we collected in the Tanintharyi. Tissue of these specimens are from the USNM tissue collection and were initially collected into 95% EtOH and subsequently stored at -80 °C. Extractions of genomic DNA from all specimens were performed on an AutoGenprep 965 (2011 AutoGen, Inc.), using standard phenol manufacturer protocols. Genomic DNA was eluted in 100 µl of AutoGen R9 re-suspension buffer. Polymerase chain reactions (PCR) were conducted for the mtDNA large ribosomal subunit (rrnL: 16S) and cytochrome oxidase subunit I (COI) using the primers: 16Sar 5’ CGCCTGTTTATCAAAAACAT 3’ and 16Sbr 5’ CCGGTCTGAACTCAGATCACGT 3’ (Palumbi et al. 1991) and COI-ReptBCF 5’ TCAACAAACCAYAAAGAYATYGG 3’ and COI-ReptBCR 5’ TAAACTTCAGGGTGGCCRAARAATCA 3’ (Castañeda and de Queiroz 2011). For some specimens, we also sequenced either part of the ND2 gene using the primers L4437–H5934 (Macey et al. 1997) or 12S (12SI: 5’ TGCCAGCAGYCGCGGTTA 3’ and 12SIII 5’ AGAGYGRCGGGCGATGTGT 3’; Puillandre et al. 2009) in order to compare with sequences available for these, or closely related species in GenBank. The PCRs were performed in 96-well plates, in 10 µl reactions, following protocols “3.6 PCR Methods: Amplification” and “3.8 PCR Purifications: EXOSAP-IT” of Weigt et al. (2012), with annealing temperatures of 54 °C for 16S and 12S, 48 °C for COI, and 52 °C for ND2. Sequence reactions were performed in 96-well plates with the PCR primers using BigDye^®^ Terminator v3.1 Cycle Sequencing Kit’s in 0.25 × 10 µl reactions and run on an Automated ABI3730 Sequencer (2011 Life Technologies). Raw chromatograms were edited in Sequencher v5.1 (2012 Gene Codes Corp.), complementary strands were aligned, and COI was inspected for proper translation, alignments were done using the MUSCLE option in Sequencher. Neighbor-joining (NJ) trees were generated in PAUP* v4.0b10 (Swofford, 2002) for the 16S and COI data separately, and of the combined data. Scale bars at bottom of each tree represent uncorrected p-distances.

### Specimen identification

Sequences for uncertain taxa were further assessed by multiple methods. First, we considered specimens placed in the same COI BINS (Ratnasingham and Hebert 2013) to represent the same species. Specimens placed in separate BINs from different geographic localities, which grouped together in the NJ trees, and that were indistinguishable based on morphology, were considered the same species with genetic variation associated with geography. Specimens that were placed in separate BINs that were either different morphologically or did not group together (i.e. grouped with other taxa) in the NJ trees, were considered different species. For specimens that we could not identify based on COI BINs, we created alignments with material from GenBank representing the same genera with as many species as possible. Neighbor-joining trees were estimated from these alignments at the family-level. This was mostly done for amphibians using 16S sequence data from GenBank. The 16S locus is known to evolve much slower than protein-encoding mitochondrial loci. Therefore, our assessments of specimen identification based on 16S data were done on a case-by-case basis, considering the geographic distance between specimens being compared and whether or not our specimens met the morphological description of the species they clustered with.

## Results

Our compiled list of species documented in the Tanintharyi contains 46 amphibians and 110 reptiles, including one caecilian, 45 anurans, 100 squamates (42 lizards and 58 snakes), and 10 turtles (Table 1). Results from our surveys in the Tanintharyi represent total observations of 51 species, 43 species (24 amphibians and 19 reptiles) in the proposed Tanintharyi National Park area, and eight additional species (one amphibian and seven reptiles) in the lowland areas (Table 1). We produced COI DNA barcode data for 297 specimens (GenBank MG935416–MG935712) and 16S data for 292 specimens (GenBank MG935713–MG936004), representing 72 species (55 amphibians and 17 reptiles), including 25 amphibians and 17 reptiles observed in the Tanintharyi (Table 1) and an additional 30 species of amphibians from northern Myanmar from our reference material (Table 2). Eleven of the species barcoded from the northern Myanmar material were also discovered in the Tanintharyi. Our COI sequences were placed into 93 BINs, of which 18 already existed. The BIN results are only mentioned in the text below if sequences went into pre-existing BINs, or if specimens of the same species were placed in separate BINs. In total, we provide sequence data for 81 species of amphibians and reptiles (Fig. 2). We provide accounts for each species observed in the Tanintharyi below, with additional comments on the reference material from northern Myanmar. The following descriptions offer brief characterization of the specimens vouchered and examined; the general distributions contain condensed and abstracted geographic data derived from ASoW (Frost 2017) and the Reptile Database (Uetz et al. 2018); both accessed 20–21 January 2018. All species were recorded in the Yeybu area unless otherwise noted. Species only observed in the Forest Reserve (Chaung-nauk-pyan area) are noted in the Natural History Notes. See Table 1 for a complete list of species observed at each site.

**Figure 2.**
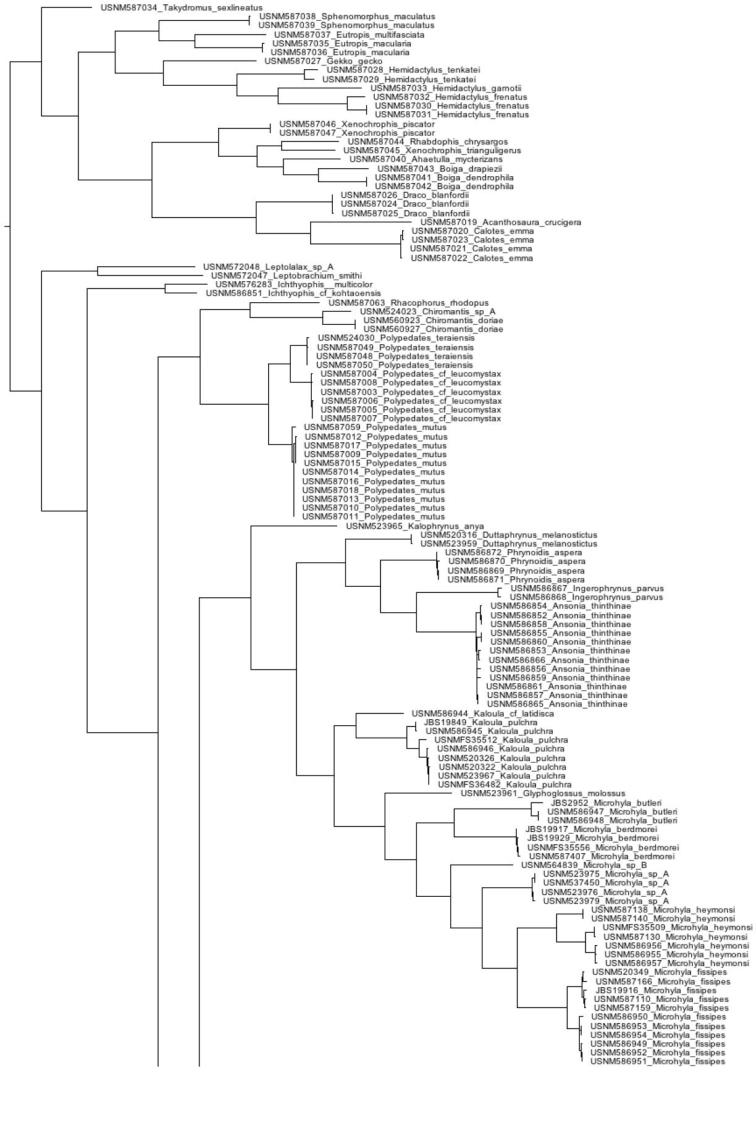
Combined (16S and COI) Neighbor-joining tree for all specimens sequenced in this study.

**Figure 2.**
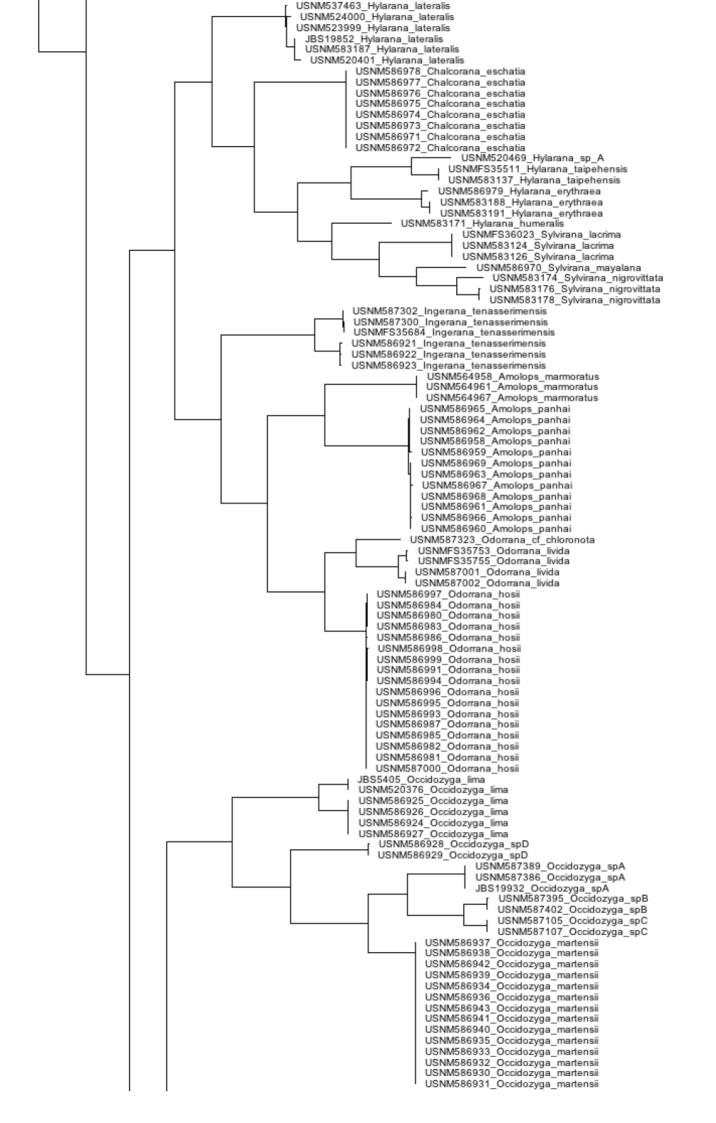
Continued.

**Figure 2.**
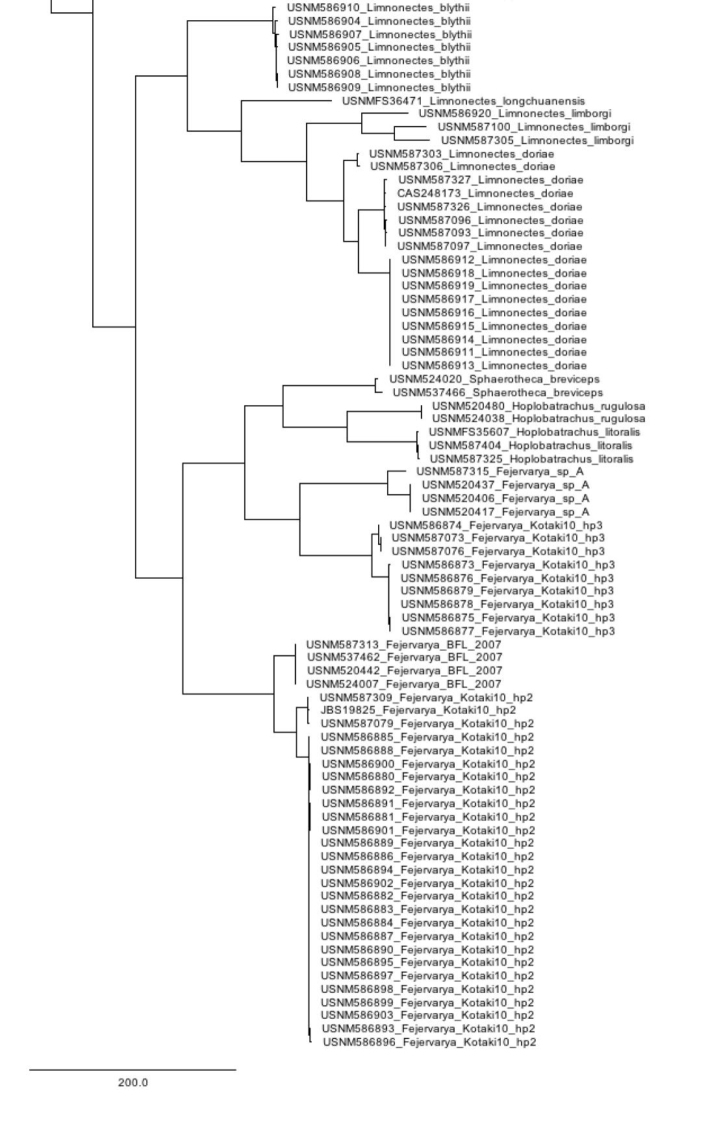
Continued.

**Table 1.** The herpetofauna of southern Tanintharyi. Marine or estuarine species are excluded. Occurrence data is derived from the California Academy of Sciences (CAS) or our observations; only species identification for the Tanintharyi Proposed National Park (PNP) area and a few of the Reserve Forest (RF) species were confirmed by specimen examination and DNA barcode data. Abbreviations: D = Dawei area, K = Kawthaung area, M = Myeik, ? = have specimens but not identified to species and/or DNA barcoding needed to confirm identification; + species present; – species absent; √ DNA barcoded.

Order	Family – Subfamily/Species	CAS	PNP Area^‡^	RF
**ANURANS**	**Bufonidae**			
	*Ansonia thinthinae*	D	√	–
	*Duttaphrynus melanostictus*	DK	–	–
	*Ingerophrynus parvus*	DK	√	+
	*Phrynoidis asper*	DK	√	+
	**Ceratobatrachidae**			
	*Alcalus tasanae*	K	–	–
	**Dicroglossidae – Dicroglossinae**			
	*Fejervarya* sp. (hp2)	?	√	–
	*Fejervarya* sp. (hp3)	?	√	+
	*Hoplobatrachus rugulosus*	D	–	–
	*Limnonectes blythii*	DK	√	+
	*Limnonectes doriae*	DK	√	+
	*Limnonectes hascheanus*	DK	–	–
	*Limnonectes kohchangae*	D	–	–
	*Limnonectes laticeps*	K	–	–
	*Limnonectes limborgi*	D	√	–
	*Limnonectes macrognathus*	D	–	–
	**Dicroglossidae – Occidozyginae**			
	*Ingerana tenasserimensis*	D	√	–
	*Occidozyga lima*	?	√	–
	*Occidozyga martensii*	DK	√	+
	**Megophryidae**			
	*Leptobrachium smithi*	DK	–	–
	*Megophrys* sp.	D	–	–
	**Microhylidae – Kalophryninae**			
	*Kalophrynus interlineatus*	D	–	–
	**Microhylidae – Microhylinae**			
	*Kaloula latidisca*	D	√	–
	*Kaloula pulchra*	DK	–	M√
	*Microhyla berdmorei*	DK	–	–
	*Microhyla butleri*	?	√	–
	*Microhyla heymonsi*	DK	√	–
	*Microhyla fissipes*	?	√	+
	*Microhyla pulchra*	D	–	–
	*Micryletta inornata*	DK	–	–
	**Ranidae**			
	*Amolops marmoratus*	D	–	–
	*Amolops panhai*	D	√	–
	*Chalcorana eschatia*	?	√	–
	*Clinotarsus alticola*	DK	–	–
**ANURANS**	*Hydrophylax leptoglossa*	D	–	–
	*Hylarana erythraea*	DK	√	–
	*Odorrana hosii*	K	√	–
	*Odorrana livida*	D	√	–
	*Sylvirana malayana*	?	√	–
	*Sylvirana nigrovittata*	DK	–	–
	**Rhacophoridae – Rhacophorinae**			
	*Chirixalus vittatus*	D	–	–
	*Nyctixalus pictus*	K	–	–
	*Polypedates mutus*	?	√	–
	*Polypedates leucomystax*	DK	√	+
	*Rhacophorus cyanopunctatus*	D	–	–
	*Rhacophorus verrucosus*	D	–	–
	*Theloderma phyrnoderma*	D	–	–
**CAECILIANS**	**Ichthyophiidae**			
	*Ichthyophis cf. kohtaoensis*	DK	√	–
**TESTUDINES**	**Geoemydidae**			
	*Cyclemys dentate*	D	–	–
	**Testudinidae**			
	*Indotestudo elongata*	–	+	–
	**Trionychidae – Trionychinae**			
	*Dogania subplana*	–	+	–
	*Nilssonia formosa*	D	–	–
**SQUAMATES – LIZARDS**	**Agamidae**			
	*Acanthosaura crucigera*	DK	√	–
	*Bronchocela burmana*	K√	–	–
	*Calotes emma*	DK	√	+
	*Calotes* “versicolor”	DK	–	–
	*Draco blanfordii*	DK	√	–
	*Draco maculatus*	DK	–	–
	*Draco taeniopterus*	DK	–	+
	**Gekkonidae**			
	*Cyrtodactylus brevipalmatus*	D	–	–
	*Cyrtodactylus lenya*	√	–	–
	*Cyrtodactylus oldhami*	DK	–	–
	*Cyrtodactylus payarhtanensis*	√	–	–
	*Gehyra mutilata*	DK	–	–
	*Gekko gecko*	DK	√	+
	*Hemidactylus frenatus*	DK	–	M√
	*Hemidactylus garnotii*	DK	√	–
	*Hemidactylus karenorum*	?	–	–
	*Hemidactylus platyurus*	K	–	–
	*Hemidactylus tenkatei*	K	–	M√
	**Lacertidae**			
	*Takydromus sexlineatus*	–	√	–
	**Scincidae**			
	*Dasia olivacea*	K√	–	–
	*Eutropis longicauda*	?	–	–
**SQUAMATES – LIZARDS**	*Eutropis macularia*	DK	√	–
	*Eutropis multifasciata*	DK	√	+
	*Lipinia vittigera*	DK	–	–
	*Lygosoma bowringii*	K	–	–
	*Scincella reevesi*	D	–	–
	*Sphenomorphus maculatus*	DK	√	+
	*Tropidophorus robinsoni*	DK	–	–
	**Varanidae**			
	*Varanus rudicollis*	–	+	–
**SQUAMATES – SNAKES**	**Acrochordidae**			
	*Acrochordus granulatus*	D	–	–
	**Colubridae – Colubrinae**			
	*Ahaetulla mycterizans*	K	√	–
	*Ahaetulla fronticincta*	D	–	–
	*Ahaetulla prasina*	DK	–	+
	*Boiga cyanea*	DK	–	–
	*Boiga dendrophila*	–	√	–
	*Boiga drapiezii*	K	√	–
	*Boiga multomaculata*	D	–	–
	*Boiga siamensis*	D	–	–
	*Coelognathus radiatus*	M	–	–
	*Dendrelaphis formosanus*	K	–	–
	*Dendrelaphis haasi*	D	–	–
	*Dendrelaphis pictus*	D	–	+
	*Dendrelaphis striatus*	DK	–	+
	*Dryocalamus subannulatus* ^†^	–	–	–
	*Gonyosoma oxycephalum*	D	–	–
	*Lycodon aulicus*	D	–	–
	*Lycodon subcinctus*	D	–	–
	**Colubridae – Natricinae**			
	*Rhabdophis chrysargos*	DK	√	–
	*Rhabdophis nigrocinctus*	D	–	+
	*Xenochrophis piscator*	D	√	+
	*Xenochrophis punctulatus*	D	–	–
	*Xenochrophis trianguligerus*	DK	√	–
	**Elapidae**			
	*Bungarus* sp.	K	–	–
	*Calliophis maculiceps*	D	–	–
	*Hydrophis schistosus*	D	–	–
	*Naja kaouthia*	–	–	+
	**Homalopsidae**			
	*Cantoria violacea*	D	–	–
	*Homalopsis semizonata*	D	–	–
	*Cerberus rynchops*	D	–	–
	*Fordonia leucobalia*	D	–	–
	**Lamprophiidae – Pseudaspidinae**			
	*Psammodynastes pulverulentus*	DK	–	–
**SQUAMATES – SNAKES**	**Pareatidae**			
	*Pareas carinatus*	DK	–	–
	*Pareas macularius*	D	–	–
	*Pareas margaritophorus*	?	–	–
	**Pythonidae**			
	*Malayopython reticulatus*	–	+	–
	**Typhlopidae**			
	*Indotyphlops braminus*	D	–	–
	**Uropeltidae**			
	*Cylindrophis burmanus*	K	–	–
	**Viperidae – Crotalinae**			
	*Trimeresurus purpureomaculatus*	DK	–	–
	*Trimeresurus stejnegeri*	K	–	–
	*Trimeresurus* sp.^§^	√	–	–
	**Xenopeltidae**			
	*Xenopeltis unicolor*	D	–	–

^‡^Most of the species observed in the proposed National Park were vouchered and barcoded. Four taxa (*Dogania cartilaginea*, *Indotestudo elongata*, *Varanus rudicollis*, and *Malayopython reticulatus*) were seen in possession of locals and only photos were taken. At the Reserve Forest sites, specimens were identified in field and released after being photographed. ^§^See Mulcahy et al. (2017) for a taxonomic treatment of the *Trimeresurus* in the Tanintharyi. ^†^See Lee et al. (2018).

**Table 2.** Additional species for which DNA barcodes were generated from the reference material from northern Myanmar. Asterisks indicate species also detected in the Tanintharyi Region.

Family	Species	State/Province
**Ichthyophiidae**	*Ichthyophis multicolor*	Ayeyawady
**Bufonidae**	*Duttaphrynus melanostictus*	Sagaing
**Dicroglossidae**	*Fejervarya* sp. (BFL2007)	Sagaing
		Magway
	*Fejervarya* sp. A (DGM2018)	Sagaing
		Mandalay
	*Fejervarya* sp. (hp2)*	Yangon
		Bago
	*Fejervarya* sp. (hp3)*	Bago
	*Hoplobatrachus litoralis*	Yangon
		Bago
	*Hoplobatrachus rugulosus*	Sagaing
	Ingerana tenasserimensis*	Mon State
	Limnonectes doriae*	Yangon
		Bago
		Mon State
	Limnonectes limborgi*	Bago
		Mon State
**Dicroglossidae**	*Limnonectes longchuanensis*	Mandalay
	Occidozyga lima*	Mandalay
		Sagaing
	*Occidozyga* sp. A (DGM2018)	Yangon
	*Occidozyga* sp. B (DGM2018)	Yangon
	*Occidozyga* sp. C (DGM2018)	Bago
	*Sphaerotheca breviceps*	Sagaing
**Megophryidae**	*Leptobrachium smithi*	Mon State
	*Leptolalax* sp. A (DGM2018)	Mandalay
**Microhylidae**	*Glyphoglossus molossus*	Sagaing
	*Kalophrynus anya*	Sagaing
	Kaloula pulchra*	Yangon
		Bago
		Sagaing
		Mandalay
	*Microhyla berdmorei*	Yangon
		Bago
	Microhyla fissipes*	Yangon
		Sagaing
		Bago
		Mandalay
		Magway
	Microhyla heymonsi*	Bago
		Mandalay
	*Microhyla* sp. A (DGM2018)	Sagaing
	*Microhyla* sp. B (DGM2018)	Magway
**Ranidae**	*Amolops marmoratus*	Mon State
	*Humerana humeralis*	Bago
	Hylarana erythraea*	Yangon
	*Hylarana lateralis*	Yangon
		Sagaing
	*Hylarana* sp. A	Sagaing
	*Hylarana tytleri*	Bago
	Odorrana cf. chloronota	Mandalay
	Odorrana livida*	Mon State
	*Sylvirana nigrovittata*	Mon State
		Mandalay
	*Sylvirana lacrima*	Mandalay
**Rhacophoridae**	*Chiromantis* sp. A (DGM2018)	Sagaing
	*Chiromantis doriae*	Mandalay
	*Polypedates teraiensis*	Yangon
		Sagaing
		Bago
	*Rhacophorus rhodopus*	Mandalay

### Caecilians

#### Ichthyophiidae – Asian caecilians (Suppl. material 1: Fig. 1)

##### Ichthyophis cf. kohtaoensis

Taxon classificationAnimaliaGymnophionaIchthyophiidae

(Nishikawa et al., 2012)

###### Description.

A single individual was found. Not dissected, sex and maturity unknown, likely juvenile; 144 mm SVL, 2 mm TailL. This individual had a bright yellow ventrolateral stripe in life (white in preservation) on each side ending below eye, ~273 primary annuli, 3 caudal annuli, eye visible, and tentacle opening much nearer eye than external choana.

###### Natural history notes.

This individual was discovered on the forest floor, immediately following a heavy rain.

###### General distribution.

Tanintharyi and peninsular Thailand.

###### Molecular data.

The 16S sequence is 98% identical to several sequences in GenBank, including GB AB686168, Ichthyophis cf. supachaii UKMHC 877 and KUHE 23189 from Malaysia and Thailand, respectively. However, our specimen fell outside of the 16S clade containing I. cf. supachaii, I. cf. hypocyaneus, and I. cf. kohtaoensis in a neighbor-joining tree of *Ichthyophis* 16S sequences in GenBank. Additional 12S data for our specimen (GenBank MG944814) placed it in the “Ichthyophis cf. kohtaoensis” clade (Suppl. material 1: Fig. 1) sister to KUHE 19615, 19617, and 19659 (GenBank AB686107–9), from Ko Samui Island, Thailand (Nishikawa et al. 2012). We note a fourth specimen identified as “I. cf. kohtaoensis” (GenBank AB686146) by Nishikawa et al. (2012), from the southern Malaysian Peninsula, is placed in a “I. cf. supachaii” + I. cf. hypocyaneus + *Ichthyophis* sp. 1” clade, consistent with their study (Suppl. material 1: Fig. 1).

###### Specimens examined.

USNM 586851.

###### Red List status.

*Ichthyophis kohtaoensis* listed as LC (Least Concern).

###### Additional *Ichthyophis*.

We included one caecilian from the legacy collection, an *Ichthyophis multicolor* from Ayeyarwady Region, Myanmar (USNM 576283). This specimen is 14% different (COI) and 8.3% different (16S) from our I. cf. kohtaoensis specimen, and is identical to GenBank FR716007, *I. multicolor*, CAS 212254, a paratype (Wilkinson et al. 2014) also collected from Ayeyarwady Region, Myanmar (Suppl. material 1: Fig. 1). Additionally, we sequenced four individuals from the California Academy of Sciences, two “*Ichthyophis* sp.” from Bago Division (CAS 239657; 12S GenBank MG944807; and CAS 239722; 12S and 16S GenBank MG944808–9) and they were placed in the *I. multicolor* clade, thus extending the known distribution of this species. The other two specimens were from near Dawei (CAS 247969; 12S and 16S GenBank MG944812–13) and near Kawthaung (CAS 247466; 12S and 16S GenBank MG944810–11), both in the Tanintharyi Region, and were placed in our I. cf. kohtaoensis clade, expanding the range of this clade from the southern Tanintharyi Region and the Thai-Malay Peninsula into the northern Tanintharyi.

### Anurans

#### Bufonidae – toads (Suppl. material 1: Fig. 2)

##### Ansonia thinthinae

Taxon classificationAnimaliaAnuraBufonidae

Wilkinson, Sellas & Vindum, 2012

###### Description.

Sample of two immature males 22.0, 22.3 mm SVL, mature males 19.5–23.4 mm (*n* = 5), immature females 21.1–22.8 (*n* = 6) and two mature females 23.3–25.6 mm SVL.

###### Natural history notes.

All individuals were on rocks in and alongside small cascades in full canopied areas of forest streams.

###### General distribution.

Known only from Tanintharyi, Myanmar.

###### Molecular data.

Our specimens form a single clade with 99–100% similarities based on 16S data, and are 96–97% similar to the type series from northern Tanintharyi, Myanmar (Wilkinson et al. 2012). We note that the type series forms a clade with our samples, and that clade is sister to *A. kraensis* (AB435250–52) to the exclusion of other peninsular species (Grismer et al. 2017). The long branch between our samples and the type series may represent genetic variation associated with geography in a low-dispersal group, or it suggests this may represent a species complex (see Suppl. material 1: Fig. 2).

###### Comments.

The sample appears to represent a single reproductive-season cohort amid maturation. If our assessment of maturity is correct, this population has slightly smaller adults than the more northern topotypic population where adult males were 22–28 mm SVL and a single adult female was 31.8 mm.

###### Specimens examined.

USNM 586852–866.

###### Red List status.

EN (Endangered).

##### Ingerophrynus parvus

Taxon classificationAnimaliaAnuraBufonidae

(Boulenger, 1887)

###### Description.

Adult male 37.7 mm and adult female, 45.2 mm SVL.

###### Natural history notes.

Both individuals were found in the leaf-litter of forest sites 1–2, and also observed in the slash & burnt area.

###### General Distribution.

Southern Myanmar and southwestern Thailand through Malay Peninsula into Greater Sunda Islands.

###### Molecular Data.

Our specimens are genetically similar to one another (99.6% identical) and, based on 16S data are placed in a clade with other *I. parvus*, though showing substantial genetic differences (91–94% identical) from GenBank material (AB746455 and AB530649–51). GenBank specimens are from Malaysia, suggesting either this may represent a species complex, or this represents a single species that shows high genetic diversity, possibly attributed to a low dispersal rate of a leaf-litter species.

###### Specimens examined.

USNM 586867–868.

###### Red List status.

LC.

##### Phrynoidis asper

Taxon classificationAnimaliaAnuraBufonidae

(Gravenhorst, 1829)

**Figure 3.**
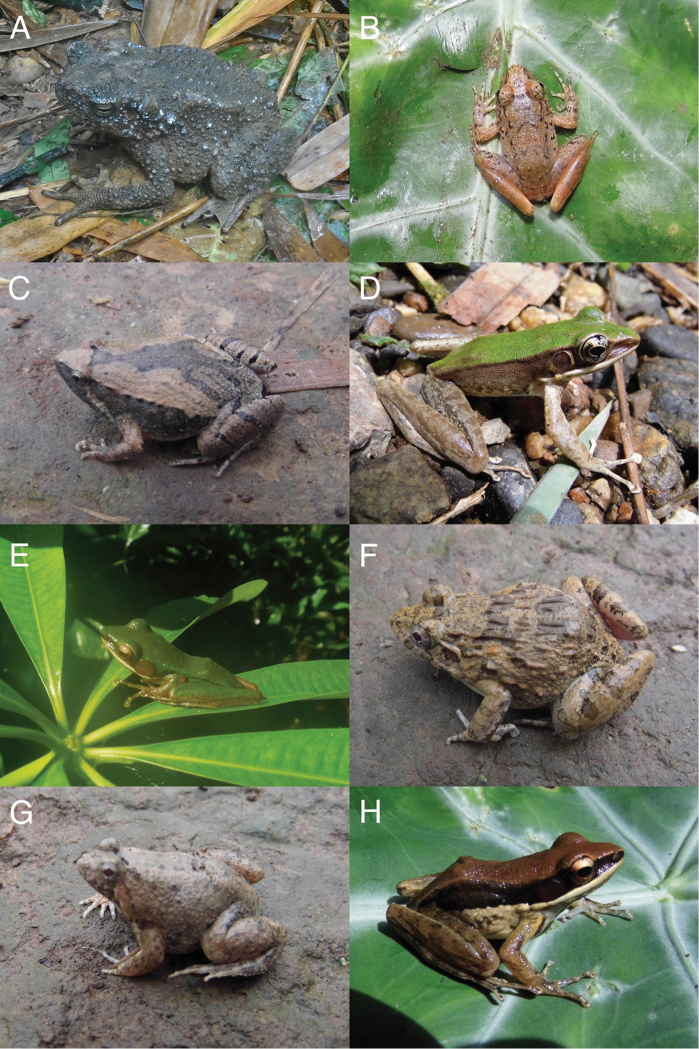
Selected amphibians found during this study’s expedition. **A** *Phrynoidis aspera* (USNM 586871) **B** *Limnonectes doriae* (USNM 586911) **C** *Microhyla fissipes* (USNM 586949) **D** *Odorrana hosii* (USNM 586980) **E** *Chalcorana eschatia* (USNM 586971) **F** *Fejervarya* sp. (USNM 586881) **G** *Occidozyga martensii* (USNM 586930) **H** *Sylvirana malayana* (USNM 586970). Photos by Myint Kyaw Thura and Daniel G. Mulcahy.

Asian Giant Toad Fig. 3A

###### Description.

Three adult males 103.0, 104.3, 104.5 mm SVL, and juvenile 34.5 mm SVL.

###### Natural history notes.

This riverine species occurs along stream borders but is principally a terrestrial species.

###### General Distribution.

Tanintharyi, peninsular Thailand and Malaysia to Sumatra, Java, and Borneo.

###### Molecular Data.

Our specimens are genetically nearly identical to one another (99–100% identical) and some are identical to one specimen in GenBank (DQ158432; FMNH 248148) from Brunei, suggesting low genetic diversity in a potentially high rate of dispersal species. These specimens form a clade with other *P. asper* from GenBank (Suppl. material 1: Fig. 2).

###### Specimens examined.

USNM 586969–972.

###### Red List status.

LC.

###### Additional bufonids.

We also sequenced two specimens of *Duttaphrynus melanostictus* from Sagaing, Myanmar (USNM 523959 and USNM 520316), for genetic comparisons. The *D. melanostictus* species complex is in need of taxonomic revision (e.g. Wogan et al. 2016). Our samples were nested among other *D. melanostictus* specimens in GenBank (not shown), identical to one (KF665340) specimen (CAS 247174), also from Sagaing but a different locality. We refer to these specimens as *D. melanostictus* until the species complex is revised.

#### Dicroglossidae (Suppl. material 1: Fig. 3)

##### Dicroglossinae – grass and fanged frogs

###### Fejervarya

Taxon classificationAnimaliaAnuraDicroglossidae

sp. ‘hp2’ (Clade 21 of Kotaki et al. 2010)

####### Description.

Medium-sized morph, adult females (7) 41.1–55.3 mm, adult males (16) 38.5–45.0 mm SVL.

####### Natural history notes.

These frogs occur in a variety of human-modified habitats from drainage ditch to rice fields. All females are gravid and bear a mix of pigmented ova and small developing follicles, although only one had a full complement of pigmented ova. Presumably the other females had bred and deposited about half of their mature ova.

####### General Distribution.

Western Thailand, Bangkok to Mae Hong Son and Three Pagoda Pass, to Yangon, Bago, and Tanintharyi, Myanmar.

####### Molecular Data.

In addition to the specimens collected in Tanintharyi, we sequenced three other individuals from Magway (USNM 587309), Yangon (MBM-JBS 19825), and Bago (USNM 587079). Our specimens were placed into two COI BINs, one containing all of the Tanintharyi specimens, and one containing the rest. The Tanintharyi specimens were placed in a pre-existing BIN (ACT3129) identified as *F. triora*. The specimens in that BIN do not appear to be publicly available, though they appear to be from Grosjean et al. (2015b). The other BIN (ADG3054) comprising our Magway, Yangon, and Bago specimens is novel. Our specimens were placed in a 16S clade with a specimen identified as *Fejervarya limnocharis* hp2, Clade 21 from Thailand (AB277299, Kotaki et al. 2010), as well as another specimen identified as *Fejervarya limnocharis* (AB162444, Sumida et al. 2007), and specimens identified as *F. triora* (Grosjean et al. 2015b, KR827756–61), all from Thailand. One of the paratypes of *F. triora* (FMNH 266160), an additional specimen (FMNH 266337) from the type description (DQ860094–95, Stuart et al. 2006b), and a third individual (AB488883) identified as *F. triora* are placed elsewhere in the phylogeny (Suppl. material 1: Fig. 3). Thus, it appears the specimens from Grosjean et al. (2015b) appear to be mis-identified, including the COI BIN BOLD:ACT3129. We refer to this clade as “*Fejervarya* sp. hp2,” belonging to the ‘hp2,’ Clade 21 of Kotaki et al. (2010).

####### Comments.

Our material extends the range of this clade from Mae Hong Son and Bangkok, Thailand to the Tanintharyi, Myanmar.

####### Specimens examined.

MBM-JBS 19825, USNM 587079, USNM 587309, USNM 587313, USNM 586880–903.

####### Red List status.

NE (Not Evaluated).

###### Fejervarya

Taxon classificationAnimaliaAnuraDicroglossidae

sp. ‘hp3’ (Clade 11 of Kotaki et al. 2010)

####### Description.

Adult females (*n* = 3) 32.3–38.5 mm, adult males (*n* = 4) 31.0–33.5 mm SVL.

####### Natural history notes.

These frogs occurred in a variety of human-modified habitats from drainage ditch to rice fields.

####### General Distribution.

Pilok, western Thailand, to Bago and Tanintharyi, Myanmar.

####### Molecular Data.

We included two individuals from Bago (USNM 587073, USNM 587076) that were related to our specimens. Our specimens were placed into two COI BINs, one for the Bago specimens (ADG3052) and one for the Tanintharyi specimens (ADG2768). We did not obtain COI sequence from one of our Tanintharyi specimens (USNM 586874), yet it was placed sister to the Bago specimens in our combined tree. All of our specimens were placed in a 16S clade with a specimen (AB277300) from GenBank identified as *Fejervarya* sp. ‘hp3’ Clade 11 of Kotaki et al. (2010). Thus, we refer to our specimens and this clade as *Fejervarya* sp. belonging to the ‘hp3,’ Clade 11 of Kotaki et al. (2010). This entire clade was placed sister to a specimen (AB488889) from the Andaman Islands, India identified as *Fejervarya* sp. ‘hp6.’ Clade 12 (Kotaki et al. 2010).

####### Comments.

Our material extends the range of this clade from Bangkok, Thailand to Bago and the Tanintharyi, Myanmar. The Grassfrogs, *Fejervarya limnocharis* complex, has gone from a single species of widespread tropical Asian frog in the early 1950s to twenty plus species in 2008 (Zug 2011) to double that number now. The number will likely continue to increase over the next decade. In Myanmar, two species commonly occur together as represented by our Tanintharyi vouchers. The sympatric species display non-overlapping size ranges between males and females of the two species, although the males of the larger species may overlap in size with that of the females of smaller species.

####### Specimens examined.

USNM 587073, USNM 587076, USNM 586873–879,

####### Red List status.

NE.

####### Additional *Fejervarya*.

We sequenced three specimens from Sagaing (USNM 520442, USNM 524007, USNM 537462) and one from Magway (USNM 587313) that were placed in one COI BIN, and were placed in a 16S clade with material in GenBank identified as *Fejervarya* sp. BFL 2007, large types 1–2 from Bangladesh (Islam et al. 2008, Hasan et al. 2012a). These were placed sister to three *F. orissaensis*, from Odisha, India and are over 8% sequence divergence from the *Fejervarya* sp. hp2 clade for COI. To be consistent, we refer to these specimens as *Fejervarya* sp. BFL 2007, which extends this Bangladesh clade into Myanmar. We sequenced three additional specimens from Sagaing (USNM 520406, USNM 520417, USNM 520437) and one from Mandalay (USNM 587315) that were placed in two COI BINs, respectively. These specimens were placed in a 16S clade with a sequence in GenBank (AF206466) of a specimen (USNM 520407) collected from the same locality in Sagaing. This clade was placed sister to a clade consisting of two new species (*F. dhaka* and *F. asmati*) recently described from Bangladesh (Howlader et al. 2016). We refer to our specimens as *Fejervarya* sp. A.

###### Limnonectes blythii

Taxon classificationAnimaliaAnuraDicroglossidae

(Boulenger, 1920)

####### Description.

Adult females (2) 114.8, 127.6 mm SVL, immature female 89.1 mm, presumed adult males 88.1 mm SVL. The vouchers, also including three juveniles, range from 30.2 to 56.3 mm SVL. All individuals have dark or black soles of hindfeet.

####### Natural history notes.

These frogs occurred in or at the edge of the forest streams.

####### General Distribution.

Southern Myanmar and western Thailand southward to Sumatra and Borneo.

####### Molecular Data.

Our specimens were placed in a 16S clade with material from GenBank identified as *L. blythii* from neighboring Thailand (GU934328) and elsewhere (RBU55270, RBU66127, RBU66131, RBU66133, and RBU66135; no locality data provided). There are several other sequences from GenBank identified as “*L. blythii*” elsewhere in the tree (e.g. RBU55269, RBU66115). The type locality for *L. blythii* is “Tenasserim valley”, Myanmar. Therefore, we consider our specimens to represent *L. blythii* based on material closest to the type locality (and fitting the description), and the other specimens in GenBank are misidentified.

####### Comments.

These semi-aquatic frogs are the largest anurans in this area in both mass and length.

####### Specimens examined.

USNM 586904–910

####### Red List status.

NT (Near Threatened).

###### Limnonectes doriae

Taxon classificationAnimaliaAnuraDicroglossidae

(Boulenger, 1887)

####### Description.

Juveniles (*n* = 4) 27.6–33.1 mm, subadult female 48.6 mm, adult males (4) 48.7–53.5 mm SVL.

####### Natural history notes.

The collection contains two distinct size classes. The gonads of the adults were sexually quiescent; this information and the “half-grown” juveniles indicate an end of the monsoon- early dry season breeding cycle.

####### General Distribution.

Myanmar and western and peninsular Thailand.

####### Molecular Data.

We included other individuals from Yangon (USNM 587326–27, and CAS 248173), Bago (USNM 587093 and 587097), and Mon (USNM 587303 and 587306) states. These were placed into three COI BINs, one for the Yangon and Bago specimens (ADG3667), one for the Mon State specimens (ADG3666), and one for the Tanintharyi specimens (AAB2123). All of these specimens formed a 16S clade with a specimen from GenBank (GU934330) identified as *L. doriae* from Myanmar, Pegu (CAS 208425) and another specimen identified as “*L. nitidus*” from peninsular Thailand (Grosjean et al. 2015b; KR827897), a species known only from the Cameron Highlands and Fraser’s Hills, of Peninsular Malaysia. The type locality for *L. doriae* is northern Tenasserim, near Mawlamyine, between the Tanintharyi and northern individuals. We note that Grosjean et al. (2015b) did not report having any *L. doriae* in their study, a species that extends onto the Thai-Malay Peninsula. Therefore, we consider the Tanintharyi specimens, the CAS specimens, and the Grosjean et al. (2015b) *L. nitidus* to be *L. doriae*.

####### Specimens examined.

USNM 586911–919, USNM 587326–27, USNM 587093, USNM 587097, USNM 587303, USNM 587306, CAS 248173.

####### Red List status.

LC

###### Limnonectes limborgi

Taxon classificationAnimaliaAnuraDicroglossidae

(Sclater, 1892)

####### Description.

A single juvenile, 24.8 mm SVL.

####### Natural history notes.

Found along forest stream.

####### General Distribution.

Northwestern Myanmar and adjacent Thailand.

####### Molecular Data.

We included two individuals, one each from Bago (USNM 587100) and Mon (USNM 587305) states. Each specimen was placed in its own COI BIN. These all form a single clade in our combined analysis (Fig. 2). These specimens all form a 16S clade with a large number of *L. limborgi* from GenBank (GU934334–36, GU934339–48, GU934353–55, and GU934357–65). Our Tanintharyi specimen (USNM 586920) is at the base of this *L. limborgi* clade, with a sequence in GenBank (AB981417) from Malaysia and the two are quite different from the rest of the clade that contains specimens from Malaysia and northern Myanmar. The type locality for *L. limborgi* is the “Tenasserim”, Myanmar. Thus, this clade likely represents multiple species that needs further investigation. Our Tanintharyi specimen represents the closest sampled to the type locality.

####### Specimens examined.

USNM 586920, USNM 587100, USNM 587305

####### Red List status.

LC

####### Additional *Limnonectes*.

We sequenced an additional specimen (MBM-USNM-FS 36471) from Mandalay Region and it was placed in a 16S clade with specimens representing a newly described species, *L. longchuanensis*, from China and northern (Kachin, Chin, and Sagaing) Myanmar (Suwannapoom et al. 2016).

##### Occidozyginae – puddle frogs

###### Ingerana tenasserimensis

Taxon classificationAnimaliaAnuraDicroglossidae

(Sclater, 1892)

####### Description.

Adult females (*n* =3) 17.6–19.7 mm SVL.

####### Natural history notes.

These frogs were found along the edges of streams in areas of low falls caused by flat rocky outcrops. All were gravid with two or three unpigmented ova.

####### General Distribution.

Eastern Myanmar and adjacent Thailand to northern West Malaysia.

####### Molecular Data.

We included three individuals from Mon State (USNM 587300, USNM 587302, and MBM USNM-FS 35684). These and our specimens were placed in two separate COI BINS (ADG3231 and ADG3230) that were 7.04% sequence divergence, and they all formed a 16S clade with another individual in GenBank from near Dawei (KF991266; CAS 246787) also identified as *Ingerana tenasserimensis*.

####### Comments.

Two other specimens in GenBank are placed sister to our clade based on 16S, one (KR827831) identified as *I.* te*nasserimensis* from Thailand (Grosjean et al. 2015b) and another (KU589219) from India identified as “*I.* sp. SB2016”. These two individuals each probably represent a different species. The type locality for this species is Tenasserim, southern Myanmar. Therefore, if this species is split into multiple species, our specimens likely represent true *I. tenasserimensis*. Another sequence in GenBank (AY322302) is identical to another (DQ283235) identified as *I. borealis*.

####### Specimens examined.

MBM-USNM-FS 35684, USNM 587300, USNM 587302, USNM 587306, USNM 586921–923.

####### Red List status.

LC.

###### Occidozyga lima

Taxon classificationAnimaliaAnuraDicroglossidae

(Gravenhorst, 1829)

####### Description.

Adult females (*n* = 3) 26.5–32.3 mm SVL, adult male (*n* = 1) 26.5 mm SVL. All had strongly tuberculate skin dorsally on trunk, bold black horizontal stripe on rear of thighs, and strongly patterned venter with pair of dark chin stripes.

####### Natural history notes.

These frogs occurred in human-modified habitats. All females were gravid.

####### General Distribution.

Widespread, eastern India to southern China southward through Southeast Asia to Java.

####### Molecular Data.

We included one individual from Sagaing (USNM 520376) and one from Mandalay (MBM-JBS 5405). These two were placed in the same COI BIN and the Tanintharyi specimens formed a separate BIN. These were sister to each other in our combined analysis (Fig. 2). These specimens formed a 16S clade with specimens from GenBank identified as *O. lima* from Java (AB530619), Myanmar (DQ283224), Thailand, Cambodia, and Laos, (KR827958–60, respectively). We note other specimens identified as *O. lima* in GenBank are placed elsewhere in the tree but are misidentified, such as AF215398 placed with *O. laevis*, and AB488903 placed with *O. martensii* specimens.

####### Comments.

The Common Puddlefrog in Myanmar or the frogs that have been identified as *O. lima* contain at least three distinct morphotypes. The taxa vary in size and coloration. The southern Tanintharyi “*O. lima*” is smaller and has a bold black and white ventral pattern lacking in the “*O. lima*” from northern Mon State and adjacent Bago, but it does share the bold, dark thigh stripe of the northern frogs.

####### Specimens examined.

MBM-JBS 5405, USNM 520376, USNM 586924–927.

####### Red List status.

LC.

###### Occidozyga martensii

Taxon classificationAnimaliaAnuraDicroglossidae

(Peters, 1867)

####### Description.

Adult females (*n* = 7) 26.4–28.4 mm SVL, adult males (*n* = 7) 19.0–24.3 mm SVL. Dorsal skin lightly rugose; dorsum dusky brown and few individuals with a faint pattern of mid-dorsal dark stripe bordered by lighter parasagittal stripe on each side; posterior thigh with faint and narrow dark horizontal stripe; venter immaculate from chest to pubis, chin and throat dark in males, dusky to immaculate in females.

####### Natural history notes.

Found in flooded fields and other human-modified habitats.

####### General Distribution.

Tanintharyi to northern West Malaysia, Thailand to southern China.

####### Molecular Data.

The Tanintharyi specimens were nearly identical to each other (<1% sequence divergence COI and 16S) and formed a 16S clade with specimens in GenBank identified as *O. martensii* (AB530610 and KP318725) from Thailand. Other sequences in GenBank identified as *O. martensii* from Vietnam (AF285214, DQ283357) and Yunnan China (DQ458255–56) form a separate clade sister to ours, indicating this may represent a species complex.

####### Specimens examined.

USNM 586930–943.

####### Red List status.

LC.

###### Occidozyga

Taxon classificationAnimaliaAnuraDicroglossidae

sp. A–D

####### Description.

We were unable to identify two of the Tanintharyi specimens to species, an adult female 20.8 mm, adult male 22.1 mm SVL. Dorsal skin lightly rugose; broad mid-dorsal brown stripe bordered by broad parasagittal tan stripes on dorsum; posterior thigh without dark horizontal bar although with sharp delineation between dorsal brown and ventral white; venter immaculate white.

####### Natural history notes.

Occurred in the same area as the previous two *Occidozyga* species.

####### General Distribution.

*Occidozyga* sp. A–B are known from Yangon, *Occidozyga* sp. C is known from Bago, and *Occidozyga* sp. D is known from Tanintharyi.

####### Molecular Data.

We included other *Occidozyga* from the legacy collection for comparative purposes. These individuals were very different genetically from the Tanintharyi specimens, and some from each other, including individuals from the same geographic regions forming different clades. This is likely a cryptic species complex; therefore, we treat each of these clades as separate, unidentified species, each was placed in its own COI BIN: *O.* sp. A from Yangon (USNM 587386, USNM 587389, and MBM JBS 19932; ADG1328), *O.* sp. B from Yangon (USNM 587395 and USNM 587402; ADG1330), *O.* sp. C from Bago (USNM 587105 and USNM 587107; ADG2685), and our specimens from the Tanintharyi Region as *O.* sp. D (USNM 586928–29; ADG1329).

####### Specimens examined.

spA-DGM2018: USNM 587386, USNM 587389, JBS 19932; spB-DGM2018: USNM 587395, USNM 587402; spC-DGM2018: USNM 587105, USNM 587107; spD-DGM2018: USNM 586928–29.

####### Red List status.

NE.

####### Additional dicroglossids.

We sequenced several other dicroglossids for comparison, including three specimens of *Hoplobatrachus tigerinus* from Yangon (USNM 587325, USNM 587404) and Bago (MBM-USNM-FS 35607). These were placed in a 16S clade with specimens from GenBank labeled as H. cf. tigerinus MS 2009 (AB530502 and AB543600) and MS 2011 (AB671173–81). These specimens are now considered to be *H. litoralis*, a recently described species from Cox’s Bazar district of Bangladesh (Hasan et al. 2012b), which extends the range of this species into Myanmar. Our 16S sequences range from 2.0–3.5% sequence divergence from the Bangladesh sequences, including one of the paratypes (AB671174). Two specimens of *Hoplobatrachus rugulosus* from Sagaing (USNM 520480, USNM 524038) were sequenced and placed in a 16S clade with other individuals in GenBank identified as *H. rugulosus*. We sequenced two individuals of *Sphaerotheca breviceps* from Sagaing (USNM 524020, USNM 537466) that were placed in a 16S clade with individuals in GenBank identified as *S. breviceps*.

#### Megophryidae

Though we did not encounter any megophryid frogs during our surveys, we sequenced one *Leptobrachium smithi* (USNM 572047) from Mon State and one *Leptolalax* (USNM 572048) from Mandalay. There are three species of *Leptolalax* known to occur in Myanmar (fide Frost 2017): *L. lateralis*, *L. melanoleucus*, and *L. pelodytoides*. The 16S data from our specimen is 90% similar to four species in GenBank (*L. bourreti*, *L. fuliginosus*, *L. petrops*, and *L. tengchongensis*), while it ranges from 85–89% similar to *L. melanoleucus* and *L. pelodytoides*. No *L. lateralis* genetic data are available for comparison; however, this species is known only from northern Myanmar, from Bhamò to Nagaland, northeastern India. We tentatively refer to our specimen as *Leptolalax* sp. A.

#### Microhylidae (Suppl. material 1: Fig. 4)

##### Kaloula latidisca

Taxon classificationAnimaliaAnuraMicrohylidae

Chan, Grismer & Brown, 2014

###### Description.

Immature female 41.6 mm SVL.

###### Natural history notes.

This single individual was found in a field near the village.

###### General Distribution.

Tanintharyi, Myanmar to northern peninsular Malaysia.

###### Molecular Data.

Our individual was placed at the base of a 16S clade containing many other specimens in GenBank, some labeled *K. baleata* (AB634687, KC822570, KM509153) and many others labeled *K.* sp. from Palawan, Peninsular Malaysia, Sulawesi, and Vietnam. Two other individuals in GenBank identified as *K. baleata* (KC179969, KC180032) were placed elsewhere in the tree with other specimens identified as *K.* sp. from Vietnam. These sequences are from a study focused on the Philippine Archipelago (Blackburn et al. 2013), in which these new species in the *K. baleata* complex were identified, each from Vietnam, Peninsular Malaysia, Palawan, and Sulawesi, and *K. baleata* was restricted to Java. The Tanintharyi specimen is at the base of the 16S clade (with the addition of 12S data, GenBank MG944815) containing the Palawan, Peninsular Malaysia, Sulawesi specimens, and the Vietnam specimens are elsewhere in the tree. Chan et al. (2013) described the Vietnam specimens as *K. indochinensis*, and Chan et al. (2014) described the Peninsular Malaysian specimens as *K. latidisca.* Given the geographic proximity, the Tanintharyi specimen likely represents *K. latidisca*, which could be confirmed with additional sequence data. We note one individual from the Blackburn et al. (2013) study (TNHC 67086) identified as *K.* sp. nov. Vietnam, but is here placed in the *K. baleata* sensu stricto clade. This specimen is actually from Java, thus incorrectly labelled in the 2013 study.

###### Specimen examined.

USNM 586944.

###### Red List status.

NE.

##### Kaloula pulchra

Taxon classificationAnimaliaAnuraMicrohylidae

(Gray, 1831)

###### Description.

Immature female 52.8, immature male 52.9 mm SVL.

###### Natural history notes.

Both individuals were collected from the border of a hotel’s parking lot in Myeik.

###### General Distribution.

Widespread, northeast India and Bangladesh to southern China and Thailand southward through Thai-Malay Peninsula to Greater Sunda Islands.

###### Molecular Data.

We sequenced other individuals from Sagaing (USNM 520322, USNM 520326, USNM 523967), Mandalay (MBM-USNM-FS 36482), Bago (MBM-USNM-FS 35512), and Yangon (MBM-JBS 19849). Our specimens were placed into three COI BINS, one for the Bago specimen, one for the Sagaing and Mandalay specimens, and one of our Tanintharyi specimens (USNM 586946) was placed in a BIN with the Sagaing and Mandalay specimens, and the other (USNM 586945) was placed a BIN with the Yangon specimen. This BIN contains nine individuals from Vietnam, Cambodia, Thailand, and Myanmar. Other specimens in BOLD identified as *K. pulchra* are placed in a different BIN, but these records are not publicly available. This variable placement suggests significant genetic variation in this group and likely indicates a species complex. Our specimens were all similar to one another based on 16S data and were placed in a 16S clade with many other individuals in GenBank identified as *K. pulchra*.

###### Specimens examined.

USNM 586945–46, USNM 520322, USNM 520326, USNM 523967

###### Red List status.

LC.

##### Microhyla butleri

Taxon classificationAnimaliaAnuraMicrohylidae

Boulenger, 1900

###### Description.

Immature males 22.9, 24.2 mm SVL.

###### Natural history notes.

All three species of *Microhyla* were captured in the same flooded fields. Data were not taken on which species were calling or difference in vocalization.

###### General Distribution.

Northeast India to southern China and Taiwan southward through Myanmar and Southeast Asia to Singapore.

###### Molecular Data.

We sequenced one individual from Yangon (MBM-JBS 2952). It was placed in a separate COI BIN from our Tanintharyi specimen, and they were placed in a 16S clade with many other *M. butleri* sequences in GenBank.

###### Comments.

Of the three species in the voucher collection, the two *M. butleri* do not display expanded vocal sacs and internally the testes appear immature.

###### Specimens examined.

USNM 586947–948.

###### Red List status.

LC.

##### Microhyla fissipes

Taxon classificationAnimaliaAnuraMicrohylidae

Boulenger, 1884

###### Description.

Adult females (*n* = 3) 24.6–28.3 mm, adult males (*n* = 3) 25.9–27.0 mm SVL.

###### Natural history notes.

Based on our limited sampling, this species appears to have been the most abundant of the breeding *Microhyla* in the flooded fields.

###### General Distribution.

Southern and central China, Myanmar and Southeast Asia to Singapore.

###### Molecular Data.

We sequenced other individuals from Sagaing (USNM 520349), Mandalay (USNM 587159), Magway (USNM 587166), Bago (USNM 587110), and Yangon (MBM-JBS 19916). These latter specimens were all placed in a single COI BIN, our Tanintharyi specimens were placed in a separate COI BIN. Based on 16S data, the northern Myanmar samples were similar to our Tanintharyi specimens; all were place in a 16S clade with other specimens in GenBank identified as *M. fissipes*, *M. ornata*, *M. mukhlesuri*, and *M. mymensinghensis*. The latter species formed a clade nested within the greater *M. fissipes* clade, which may be an artifact of limited data (only 16S). *Microhyla ornata* sequences from GenBank were nested throughout this clade (see comment below).

###### Comments.

*Microhyla fissipes* was recently resurrected (Matsui et al. 2011) for populations ranging in Myanmar, Indochina, and China previously recognized as *M. ornata*. *Microhyla ornata* is now restricted to southern India and Sri Lanka. While we are using the name *M. fissipes*, we recognize that Myanmar and Indo-China populations are a different species than the eastern China ones from which the holotype of *M. fissipes* derives; however, no systematist has yet sorted out the taxonomy of these more western Southeast Asian populations.

###### Specimens examined.

USNM 586949–954, USNM 520349, USNM 587159, USNM 587166, USNM 587110.

###### Red List status.

LC.

##### Microhyla heymonsi

Taxon classificationAnimaliaAnuraMicrohylidae

Vogt, 1911

###### Description.

Adult female (*n* = 1) 25.1 mm, adult males (2) 20.1, 20.6 mm SVL.

###### Natural history notes.

From flooded fields.

###### General Distribution.

Northeast India through southern China to Taiwan, southward through Southeast Asia to Sumatra.

###### Molecular Data.

We sequenced four other individuals, two from Bago (USNM 587130, MBM-USNMFS 35509) and two from Mandalay (USNM 587138, USNM 587140). These were each placed in their own COI BIN, as were our specimens from the Tanintharyi. These were similar to our Tanintharyi specimens based on 16S data and were all placed in a 16S clade with specimen in GenBank identified as *M. heymonsi*. There were two distinct clades within the GenBank *M. heymonsi* material; ours were placed in one with other specimens from Myanmar (e.g. KC179993), and Singapore (e.g. HM359093). Sheridan et al. (2010) identified three clades within *M. heymonsi*. These data indicate this represents a species complex in need of further revision.

###### Specimens examined.

USNM 586955–957, USNM 587130, USNM 587138, USNM 587140.

###### Red List status.

LC.

###### Additional *Microhyla*.

We sequenced nine additional specimens of *Microhyla* from northern Myanmar. Four (USNM523975, USNM523976, USNM523979, USNM 537450) from Sagaing did not match any species description, were placed in their own COI BIN, were placed in their own clade in the 16S tree, and likely represent a new species (*M.* sp. A.). One identified as *M. rubra* was placed in a COI BIN with another individual identified as *M. rubra* from Myanmar and the 16S sequence is identical to a specimen in GenBank (KM509166) from Magway, Myanmar. Other specimens identified as *M. rubra* in GenBank were placed elsewhere in the tree. However, six of these (KU214856–61) represent a recently described species (*M. mihintalei*, Wijayathilaka et al. 2016), while the other two (AB201192 and KU214855) represent *M. rubra* from Sri Lanka and India (Karnataka). The type locality for *M. rubra* is “in the Carnatic near rivers, in sandy banks… also Ceylon” India and Sri Lanka (fide Frost 2017). Therefore, our specimen, the KM509166, and the other specimen in the BOLD BIN (BOLD:ACW0810) likely represent a new species. We refer to our specimen as *M.* sp. B. Two others identified as *M. berdmorei* from Yangon (MBM-JBS19917 MBM-JBS 19929), and two *M. berdmorei* from Bago (MBM-USNM-FS 35556, USNM 587407) were placed in the same COI BIN, and were placed in a 16S clade identical to one from Sagaing, Myanmar (KC179981; de Sá et al. 2012) and similar to three others identified as “*M. sp*. B MS-2009” from Bangladesh (Hasan et al. 2012a). This clade was placed sister to two other clades identified as *M. berdmorei* (and one *M. fowleri*), which suggests a species complex in need of revision. We sequenced a paratype (USNM 523965) of *Kalophrynus anya* (Zug 2015) and one *Glyphoglossus molossus* (USNM 523961), both from Sagaing, Myanmar.

#### Ranidae – true frogs (Suppl. material 1: Figs 5–6)

Because a considerable amount of COI barcode data are available for ranid frogs, we also conducted a similar comparison using a neighbor-joining tree with material from GenBank.

##### Amolops panhai

Taxon classificationAnimaliaAnuraRanidae

Matsui & Nabhitabhata, 2006

###### Description.

All juveniles (*n* = 12) 29.4–35.2 SVL.

###### Natural history notes.

Moderately common in the areas of large rocks and splash zones.

###### General Distribution.

Tanintharyi, Myanmar and western/central peninsular Thailand.

###### Molecular Data.

Our specimens were less than 1% (COI and 16S) sequence divergence from each other. These were placed in a COI BIN with two individuals of *A. panhai* from Thailand (Grosjean et al. 2015b). Our specimens were placed in a 16S clade with other individuals from GenBank identified as *A. panhai* from Dawei, Tanintharyi, Myanmar (JF794451, Dever et al. 2012) and Thailand (AB211487–8, Matsui et al. 2006; KR827705–6, Grosjean et al. 2015b). Our specimens were placed sister to two COI barcodes for *A. panhai* (KR087620–1), two of the same individuals for which 16S data were available (Grosjean et al. 2015b).

###### Specimens examined.

USNM 586958–969.

###### Red List status.

LC.

###### Comments.

We included three individuals (USNM 564958, USNM 564961, USNM 564967) of *Amolops marmoratus* from Mon State, Myanmar. These were placed in a COI BIN with another individual from Thailand identified as *A. marmoratus*, and in a 16S clade with other individuals identified as *A. marmoratus* from Myanmar (Dever et al. 2012) in the Tanintharyi (JF794450), Mon State (JF794452–56), and Shan State (JF794470) and Thailand (AB211486, Matsui et al. 2006). Our specimens were placed sister to one COI barcode for *A. marmoratus* (KR087617), one of the same individuals for which 16S data were available (Grosjean et al. 2015b).

##### Chalcorana eschatia

Taxon classificationAnimaliaAnuraRanidae

Inger, Stuart & Iskandar, 2009

Peninsular Copper-cheeked Frog Fig. 3E

###### Description.

An immature female, 35.4 mm SVL, two adult females, 47.3–48.4 mm and five adult males, 30.5–34.6 mm SVL. Both adult females are gravid.

###### Natural history notes.

Streamside in the primary forest.

###### General Distribution.

Tanintharyi Myanmar to southern Thailand.

###### Molecular Data.

Our specimens were placed in a single COI BIN with four specimens from Thailand identified as *Hylarana eschatia*. Our specimens were placed in a 16S clade with material from GenBank, with all specimens of *C. eschatia* from neighboring Thailand, including type material (e.g. FMNH 268523–30, Inger et al. 2009). Our specimens ranged from 0–1.4% sequence divergence from these individuals in GenBank. Our specimens were placed sister to a clade of COI barcodes for *C. eschatia* (KR087702–5), some of the same individuals for which 16S date were available (Grosjean et al. 2015b).

###### Comments.

The Malayan populations were formerly included in *Hylarana chalconota*, which is now restricted to southern Sumatra, Java, and Bali. Our findings represent new country records for this species.

###### Specimens examined.

USNM 586971–979.

###### Red List status.

NE.

##### Hylarana erythraea

Taxon classificationAnimaliaAnuraRanidae

(Schlegel, 1837)

###### Description.

Adult female, 71.8 mm SVL.

###### Natural history notes.

This gravid female was found in a flooded field.

###### General Distribution.

Eastern Myanmar and Southeast Asia southward to Borneo.

###### Molecular Data.

In addition to our single specimen, we sequenced two individuals (USNM 583188 and USNM 583191) from the Yangon region. Our specimen was 2.3% sequence divergence from the Yangon specimens for COI and they were placed in separate BINs, and ranged from 0–0.2% sequence from each other for 16S, and were placed in a 16S clade with other individuals identified as *H. erythraea* from the Yangon area (KR264118–19; USNM 583188 and USNM 583190), and Tanintharyi (KR264061, KR264066; CAS 229614 and CAS 247465), Myanmar (Oliver et al. 2015), and Thailand (KR827786, Grosjean et al. 2015b). We note there are several other clades of *H. erythraea* in our 16S tree, and though they all represent a monophyletic group, there is great molecular divergence among them, indicating another species complex in need of revision. Our specimens were placed sister to a COI barcode for *H. erythraea* (KR087693), one of the same individuals for which 16S date were available (Grosjean et al. 2015b), and this clade was sister to another clade of *H. erythraea*, similar to the 16S results.

###### Specimens examined.

USNM 586979, USNM 583188–91.

###### Red List status.

LC.

###### Additional *Hylarana*.

When we began the barcode analysis of our Tanintharyi *Hylarana*, the genus contained more than three dozen species. *Hylarana* sensu lato was clearly not a monophyletic group, as Oliver et al. (2015) subsequently demonstrated by restricting it to four species (*H. erythraea*, *H. macrodactyla*, *H. tytleri*, and *H. taipehensis*) of which the first three species likely have populations in Myanmar. Because of the larger content of *Hylarana* s. l., we sequenced several other individuals from elsewhere in Myanmar, including six *H. lateralis*, two from Yangon (USNM 583187 and MBM-JBS 19852), four from Sagaing (USNM 520401, USNM 523999, USNM 524000, and USNM 537463), two *H. macrodactyla* from Bago (USNM 583137 and MBM-USNM-FS 35511), one *H. macrodactyla* from Sagaing (USNM 520469), and one Humerana cf. humeralis (USNM 583171) from Bago. Our *H. lateralis* were placed in a single COI BIN, and in a 16S clade with specimens identified as *Humerana lateralis* (see Oliver et al. 2015 for new generic allocations), which is sister to *Humerana miopus*. Our COI data placed our *H. lateralis* sister to two *H. lateralis*, the same specimens as in the 16S tree (Grosjean et al. 2015b). The *H. macrodactyla* from Sagaing was placed in its own COI BIN, and the two from Bago were placed in a separate COI BIN, and in a 16S clade with several ‘H. cf. taipehensis’ (AB530522–5, AB543603; we note that these identifications are almost certainly incorrect) from Bangladesh (Hasan et al. 2012a), and a H. cf. tytleri (KM069012) from Tripura, India (Biju et al. 2014). This clade was sister to a clade of *H. macrodactyla* from Myanmar and Laos. Our *H. macrodactyla* specimen (USNM 520469) from Sagaing was placed sister to this *H. macrodactyla* + H. cf. taipehensis clade in our 16S tree. Our COI data placed our three specimens in a clade with the “H. cf. tytleri” specimen from Tripura, India, though with considerable sequence variation. This latter clade was sister to an *H. macrodactyla* COI clade. The type locality for *H. tytleri* is Bangladesh, whereas the type localities for *H. taipehensis* and *H. macrodactyla* are Taiwan and Hong Kong, respectively. Oliver et al. (2015) identified four specimens as *H. tytleri*, all from Myanmar (their materials examined in Appendix A), but mistakenly labelled them in GenBank as *H. erythraea*; these specimens are placed in the *H. erythraea* clade of the 16S tree. Clearly, *H. erythraea* is another group in need of revision. We tentatively refer to our Bago specimens as H. cf. tytleri because the H. cf. tytleri Tripura, India (Biju et al. 2014) specimen is the closest geographically to the type locality of *H. tytleri* and we refer to our Sagaing specimen (USNM 520469) as *H.* sp. A. Our *Humerana humeralis* is identical to a specimen (USNM 583170, collected contemporaneously and already in GenBank, KR264113) identified as *Humerana* sp., and the two were placed sister to other specimens identified as Humerana cf. humeralis (KM069010) and *Humerana humeralis* (KU589217, KU589223–4); though with considerable genetic differences (6–15% sequence divergence) this clade ranges from Assam, India to Bago, Myanmar and the type locality is Bhamò, Kachin State, Myanmar. The COI data for our specimen are considerably different (>18%) from the Humerana cf. humeralis from Assam, India.

##### Odorrana hosii

Taxon classificationAnimaliaAnuraRanidae

(Boulenger, 1891)

Green Odor Frog Fig. 3D

###### Description.

A total of 17 individuals were collected in the Tanintharyi. Two adult females, 78.3, 87.8 mm SVL, adult males (*n* = 13) 52.5–60.4 mm SVL (measurements for adults only). All individuals share dark lores, a white upper lip with white stripe extending to above axilla, and an immaculate (nearly white) venter from chin to pubic area.

###### Natural history notes.

The unpigmented follicles and enlarging oviducts in females and the modest ductus deferens and no external visible vocal sacs of males suggest that breeding had not yet begun in this population. All were found on branches over and adjacent to forest streams.

###### General distribution.

Peninsular Myanmar, Thailand, West Malaysia to Sumatra and Borneo.

###### Molecular data.

Our specimens ranged from 0–0.5% sequence divergence from each other based on COI data and were placed in one BIN. These were also placed in a 16S clade identical to other individuals (e.g. DQ650595–604) identified as *O. hosii* from neighboring Thailand (Stuart et al. 2006a). This clade also contained two individuals identified as *O. livida* (KR827970–1) from Thailand (Grosjean et al. 2015b), which are presumably misidentified. Our specimens were placed sister to the same two specimens identified as *O. livida* (KR087841–2) in the 16S tree (Grosjean et al. 2015b), which are misidentified. Two other clades of *O. hosii* from GenBank were recovered in the 16S tree, sister to each other, and that clade is sister to the one containing our specimens.

###### Specimens examined.

USNM 586981–87, 586991, 586993–587000.

###### Red List status.

Least Concern.

##### Odorrana livida

Taxon classificationAnimaliaAnuraRanidae

(Blyth, 1856)

###### Description.

Single adult female 86.8 mm SVL, adult male 73.0 mm SVL. These individuals have strongly dusky colored chins and anterior chests.

###### Natural history notes.

N/A

###### General Distribution.

Northeast India to peninsular Myanmar and Thailand.

###### Molecular Data.

We included two individuals from Mon (MBM-USNM-FS 35753, MBM-USNM-FS 35755). These and our Tanintharyi specimens ranged from 0–2.5% sequence divergence from each other based on COI data and were placed in two COI BINs (Mon and Tanintharyi) and were placed in a 16S clade with other individuals (DQ650612–615) identified as *O. livida* from neighboring Thailand and Myanmar (Stuart et al. 2006a). Two other specimens from GenBank (AB200949–50) identified as *O. supranarina*, from the Ryukyu Islands, Japan, were also in this clade. However, these specimens may be misidentified, because a sequence identified as *O. livida* (AB200955) from the same study (Matsui et al. 2005b) was placed in the *O. chloronota* clade, and the *O. chloronota* sequence (AB200954) from that study (Matsui et al. 2005b) was placed in the *O. graminea* clade. There were no other *O. livida* COI sequences available, except for the two mis-identified specimens (see *O. hosii* above).

###### Specimens examined.

USNM 587001–02.

###### Red List status.

DD (Data Defficient).

###### Additional *Odorrana*.

We sequenced one individual (USNM 587323) of *Odorrana* from Mandalay, Myanmar. This individual was placed in its own COI BIN and in a 16S clade with specimens identified as *Odorrana graminea* (KR827967–8) and O. cf. chloronota (DQ650605–11) from Thailand (Stuart et al. 2006a). Therefore, we tentatively identify this specimen as O. cf. chloronota (sensu Stuart et al. 2006a).

##### Sylvirana malayana

Taxon classificationAnimaliaAnuraRanidae

Sheriden & Stuart, 2018

Black-sided Forest Frog Fig. 3H

###### Description.

Immature female, 39.9 mm SVL.

###### Natural history notes.

Specimen was found adjacent to the forest stream.

###### General Distribution.

Once thought to be widespread, Nepal, northern peninsular and Northeast India to southwest China and Southeast Asia. However, several recent studies based on molecular data suggest “*S. nigrovittata*” represents a multiple-species complex, with this species (*S. malayana*) being recently described from the Thai-Malay Peninsula our our specimen extends the range into central Tanintharyi, ostensibly overlapping with *S. nigrovittata* sensu strico (see below).

###### Molecular Data.

One specimen was collected in 2014, it was placed in its own COI BIN and in a 16S clade with other individuals identified as *S. nigrovittata* in GenBank from Phang-Nga, Thailand (KR827826, Grosjean et al. 2015b), and other un-published sequences that lack specimen information (KF738999–9002, EU604197). Our specimen was placed sister to the COI barcode for the same individual for which 16S date were available (Grosjean et al. 2015b). A very recent paper (Sheriden and Stuart 2018), published during revision of this manuscript, describes four new species in this complex. Our specimen falls (not shown) within one of their newly described peninsular-Malaysian species (*S. malayana*), extending it into Tanintharyi. Dubois (1992) designated a lectotype of *Limnodytes nigrovittatus* Blyth 1856 and restricted the type locality to “Mergui and the valley of the Tenasserim River.” Mergui is present day Myeik, adjacent to the mouth of the Tanintharyi River. We find it peculiar that our specimen, from a tributary of the Tanintharyi River, represents this newly described species and not *S. nigrovittata* as it was collected in between their *S. nigrovittata* genetic samples (from western Thailand) and the type locality (Myeik). Nevertheless, two clades appear to extend across the Isthmus of Kra on the eastern (*S. nigrovittata*) and western (*S. malayana*) sides. The type of *S. nigrovittata* is a female specimen for which distinguishing characteristics are lacking. Until sequence data can be obtained from the type specimen, or additional material can be collected from the Myeik area proper, we remain skeptical that the newly described *S. malayana* may represent *S. nigrovittata* sensu stricto and populations sampled by Sheriden and Stuart (2018) to the north, and east may represent a new species.

###### Specimens examined.

USNM 586970.

###### Red List status.

LC.

###### Additional *Sylvirana*/*Hylarana.*

We sequenced several other individuals from northern Myanmar identified as *Sylvirana/Hylarana* sp. We found several clades of “*S. nigrovittata*” in our 16S tree. We sequenced two individuals from Mon State (USNM 583176 and USNM 583178) and one from Mandalay (USNM 583174) that were identified as *S. menglaensis* and were placed in two COI BINs (Mon and Mandalay). The Mandalay specimen was placed in a BIN with nine other individuals, seven identified as *H. menglaensis*, and two as *S. nigrovittata*. The COI data placed these individuals in a clade with other *S. menglaensis* for which COI data were available, with the BINs forming clades, with other clades of specimens identified as *S. menglaensis*. These were all placed in a 16S clade containing other specimens identified as *S. menglaensis* in GenBank (KR827810–22, Grosjean et al. 2015b). Sheriden and Stuart (2018) placed *S. menglaensis* in synonymy with *S. nigrovittata*. We sequenced three additional specimens from Mandalay (USNM 583124, USNM 583126, and MBM-USNM-FS 36020). These specimens were placed in their own COI BIN and in a 16S clade, nearly identical to two individuals identified as *Hylarana* sp. C MS-2010 (AB543604–5, Hasan et al. 2012a) from Bangladesh, and two sequences in GenBank (KR264116–7), from the same series as ours (USNM 583124–5) identified as Sylvirana cf. nigrovittata (Oliver et al. 2015). Our sequences differ by two base-pairs from the ones in GenBank, including USNM 583124 (Oliver et al. 2015). We sequenced this specimen twice and provide the raw trace files in BOLD. There are many other 16S sequences in GenBank identified as *Sylvirana nigrovittata* elsewhere in the tree, but no other COI sequences to compare. Sheriden and Stuart (2018) described specimens of this clades as a new species *S. lacrima*, and our specimens fall out within this clade (not shown).

#### Rhacophoridae – Whipping Frogs (Suppl. material 1: Fig. 7)

##### Polypedates cf. leucomystax

Taxon classificationAnimaliaAnuraRhacophoridae

(Gravenhorst, 1829)

###### Description.

Three immature females 61.7, 62.8, 69.7 mm, adult female 82.0, and two adult males 45.7, 45.8 mm SVL.

###### Natural history notes.

Two species of *Polypedates* were found in a rural landscape during heavy rains. At the time of collection, all specimens (six adult males and ten females) were assumed to represent a single species and the location of individual specimens was not noted, though all were collected from the same flooded fields. The results from the barcode analysis revealed that two genetic lineages were present in the total *Polypedates* sample, and one of lineages was represented by only males and the other by only females. The males (45.3–50.2 mm SVL) have the vocal sacs open although there is no indication externally (i.e., stretched throat skin and pigmented) and the testes are enlarged. The majority (*n* = 9) of the females range from 60.7–70.0 mm SVL; their oviducts have only begun to enlarge and the follicles within the ovaries are small and presumably pre-vitellogenic or in early vitellogenesis; a single large female 82.0 mm SVL has mature oviducts and ovarian follicles are well yolked but not pigmented. All females have distinct dark brown longitudinal stripes (commonly broken) on the dorsum; stripes are absent or reduced on most of the males. Additionally, the lower lip of the females is black bordered and immaculate in the males. It is notable that without the barcode data, we would have interpreted the vouchers as a single species with distinctly smaller males and larger females. The reproductive data suggest that the smaller species breeds early in the monsoon and the larger one in the late monsoon or early dry season.

###### General Distribution.

Widespread in South Asia, eastern India to southwestern China through Southeast Asia to the Greater Sunda Islands and Philippine Islands.

###### Molecular Data.

Our specimens were placed in their own COI BIN, and were placed at the base of a large 16S clade of *P. leucomystax*, from GenBank (sensu Kuraishi et al. 2012). These specimens may represent a new species, closely related to *P. leucomystax* (see comments below). For now, we refer to them as P. cf. leucomystax.

###### Comments.

Initially, the specimens collected were considered to represent a single species, but the DNA barcoding revealed two distinct lineages. The *P. leucomystax* complex of frogs remains contentious. Several recent studies have produced 16S (e.g. Kuraishi et al. 2012, Pan et al. 2013) and COI (Buddhachat and Suwannpoom 2018) sequence data, resolving some of the issues within this group. Our Tanintharyi frog loosely fits the morphological description of the *P. leucomystax.* Our clade was placed sister to the COI Polypedates cf. leucomystax clade of Buddhachat and Suwannpoom (2018; not shown) However, a detailed morphological comparison and additional sequence data are supporting our lineage represents a new species that occurs from northern Tanintharyi, and further to the north in Myanmar (Wilkinson, Mulcahy, Zug, in prep.).

###### Specimens examined.

USNM 587003–7008

###### Red List status.

*Polypedates leucomystax* is listed as LC.

##### Polypedates mutus

Taxon classificationAnimaliaAnuraRhacophoridae

(Smith, 1940) Burmese

###### Description.

All individuals are immature; six females 59.9–66.6 mm, three males 45.3–50.3, and a sex indeterminate specimen 45.2 mm SVL (*n*=10).

###### Natural history notes.

See preceding species account.

###### General Distribution.

Tanintharyi Myanmar, Thailand, Yunnan and Guangxi, China.

###### Molecular Data.

The second clade of our *Polypedates*, and an additional specimen from Mandalay (USNM 587059) were placed in a COI BIN with four individuals from Thailand identified as *Polypedates* sp. Our specimens were placed in a 16S clade with individuals identified as *P. impresus* (Pan et al. 2013) and Polypedates cf. mutus 2 of Kuraishi et al. (2012). Note, older specimens in GenBank in this clade are labeled as *P. leucomystax*, *P. megacephalus*, and *P.* sp. The very recently published paper examining Thailand species of *Polypedates* with COI data (Buddhachat and Suwannpoom 2018) identified five major clades in the *P. leucomystax* complex. Our specimens were placed in their “Northern A *Polypedates* sp.” clade (not shown). Our ongoing work (Wilkinson, Mulcahy, Zug, in prep.) suggests that this clade represents *P. mutus* senus stricto (the *P. mutus* 1 clade of Kuraishi et al. 2012).

###### Specimens examined.

USNM 587009–018, USNM 587059.

###### Red List status.

LC.

###### Additional rhacophorids.

We sequenced three individuals initially identified as *Polypedates teraiensis* from Sagaing (USNM 524030), Yangon (USNM 587048), and Bago (587049). These were all placed in their own COI BIN and in a 16S clade with specimens from GenBank identified as *P. teraiensis* (AB530512–21) and two individuals (AB728167–8) labeled *P. leucomystax*, presumably misidentified. Additionally, we sequenced three additional specimens initially identified as *Chiromantis* spp. Two specimens (USNM 560923, USNM 560927) from Mandalay initially identified as *C. hansenae*, were placed in their own COI BIN and in a 16S clade with other individuals from GenBank identified as *Chiromantis doriae*. There is considerable genetic variation among the *C. doriae* specimens in GenBank, indicating that *C. doriae* as currently used is a species complex in need of revision. The third specimen (USNM 524023) from Sagaing was initially identified as *C. nongkhorensis*, but was placed at the base of the 16S clade containing *C. nongkhorensis* and *C. doriae* specimens from GenBank (Aowphol et al. 2013). This specimen may represent a new species; however, we treat it as *Chiromantis* sp. A for now.

### Testudines

#### Testudinidae – tortoises

##### Indotestudo elongata

Taxon classificationAnimaliaTestudinesTestudinidae

(Blyth, 1853)

**Figure 4.**
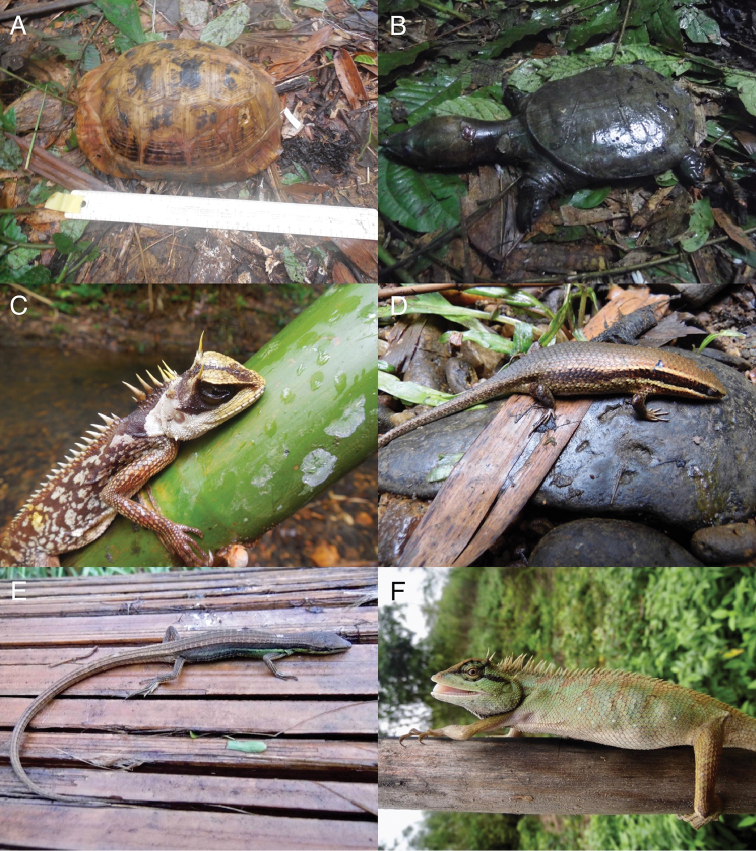
Selected turtles and lizards found during this study’s expedition. **A** *Indotestudo elongata* (USNM HerpImage 2896) **B** *Dogania subplana* (USNM HerpImage 2897) **C** *Acanthosaura crucigera* (USNM 587019) **D** *Eutropis multifasciata* (USNM 587035) **E** *Takydromus sexlineatus* (USNM 587034) **F** *Calotes emma* (USNM 587022). Photos A & C by Daniel G. Mulcahy, all others by Myint Kyaw Thura.

Elongate Tortoise Fig. 4A

###### Description.

A shell of this species was seen in Yeybu village. Carapace length (straight) was approximately 22 cm; sex indeterminate owing to absence of a plastron. Nine distinct growth annuli were visible on the second right pleural scute.

###### Natural history notes.

The tortoise from which the shell was derived was presumably from the adjacent forest.

###### General Distribution.

Widespread, Nepal, northern peninsular and Northeast India to Southeast Asia into northernmost West Malaysia.

###### Molecular Data.

No molecular data available.

###### Specimens examined.

The specimen was found in a camp and photo vouchered. USNM Herp Image 2896

###### Red List status.

EN (Endangered); CITES Appendix II.

#### Trionychidae – softshell turtles

##### Dogania subplana

Taxon classificationAnimaliaTestudinesTrionychidae

(Saint Hilaire, 1809)

Hillstream Softshell Turtle Fig. 4B

###### Description.

No measurements were taken.

###### Natural history notes.

Uncertain origin, but presumably from the nearby river.

###### General Distribution.

Myanmar and Thailand through Peninsular Malaysia, south to Sumatra, Java, and Borneo.

###### Molecular Data.

No molecular data available.

###### Comments.

This individual was seen in Yeybu village. No measurements were taken.

###### Specimens examined.

USNM Herp Image 2897

###### Red List status.

LR/LC (Low Risk/Least Concern; needs updating); CITES Appendix II.

### Squamata - Lizards

#### Agamidae – spiny lizards (Suppl. material 1: Fig. 8)

##### Acanthosaura crucigera

Taxon classificationAnimaliaSquamataAgamidae

Boulenger, 1885

Masked Prickly-naped Lizard Fig. 4C

###### Description.

Adult male 110.4 mm SVL, 222 mm TailL, 27.3 mm HeadL; 42% TrunkL/SVL, 30% Forarm/CrusL, 25% HeadL/SVL, 82% HeadW/HeadL, 61% HeadH/HeadL, 61% SnEye/HeadL, 23% EyeEar/HeadL.

*Acanthosaura crucigera* is a striking lizard with its postorbital and nape spines, highlighted by dark brown face mask bordered below by white lips and jowl. This specimen has seven cervical spines, 20 fourth finger lamellae and 26 fourth toe lamellae.

###### Natural history notes.

Only one individual was seen during the six days of survey days in the forest. This individual was on a branch overhanging the stream.

###### General Distribution.

Tanintharyi Myanmar through southern Thailand and Cambodia, southward to northern West Malaysia.

###### Molecular Data.

No COI sequences are currently available for *A. crucigera*, and our specimen was placed in its own COI BIN and differs from other *Acanthosaura* species by 16–18%. Our specimen is 97% similar to an *A. crucigera* in GenBank (AB031980) from Koh Chang Island, Thailand (Honda et al. 2000a) based on 16S data and is placed sister to this specimen in our 16S tree.

###### Specimens examined.

USNM 587019.

###### Red List status.

NE.

##### Calotes emma

Taxon classificationAnimaliaSquamataAgamidae

(Gray, 1845)

Barred Forest Lizard Fig. 4F

###### Description.

Adult females (*n* = 2) 101.7, 106.1 mm SVL, 279, 284 mm TailL, adult males (*n* = 2) 84.5, 102.2 mm SVL, 236, 279 mm TailL; 16.4 , 17.4 mm & 12.2, 16.7 mm, respectively HeadL; 46–50% TrunkL/SVL,14–17% HeadL/SVL, 68–79% HeadW/HeadL, 33–46% HeadH/HeadL, 43–44% SnEye/HeadL, 25–29% EyeEar/HeadL. Dorsal spines range from 39 to 52, fourth finger lamellae 22 to 25, and fourth toe lamellae 25 to 31.

###### Natural history notes.

Although *C. emma* was found within the forest, these individuals were in an area of open canopy. It appears to be mainly a forest-edge and woody fencerow denizen.

###### General Distribution.

Northeast India to southwestern China southward to northern West Malaysia.

###### Molecular Data.

Our specimens were placed in their own COI BIN. No other COI sequences are currently available for *C. emma*, and our sequences differ from other *Calotes* species by 10–12%. Based on 16S data, our specimens were placed sister to all other sequences of *Calotes* currently in GenBank.

###### Specimens examined.

USNM 587020–023.

###### Red List status.

NE.

##### Draco blanfordii

Taxon classificationAnimaliaSquamataAgamidae

Boulenger, 1885

###### Description.

Adult females (*n* = 2) 101.4, 107.9 mm SVL, adult male (*n* = 1) 96.4 mm SVL, 183–190, 178 mm TailL respectively; 175, 178 mm & 174 mm HeadL; 46–50% TrunkL/SVL, 16–18% HeadL/SVL, 68–74% HeadW/HeadL, 42–51% HeadH/HeadL, 11% SnEye/HeadL, 25–29% EyeEar/HeadL. Fourth finger lamellae range from 26 to 28 and fourth toe lamellae 26 to 30.

###### Natural history notes.

As for the preceding *C. emma, Draco* occurs in open-canopied situations and along forest edges. Each of the females was gravid; the smaller female bears four eggs, two on each side; the larger one has seven eggs, three on right, four on left. The shelled eggs are ~10–12 mm in length.

###### General Distribution.

Bangladesh through southern Myanmar and Thailand to northern West Malaysia, also Vietnam.

###### Molecular Data.

Our specimens were placed in their own COI BIN, no other COI sequences are currently available for *D. blanfordii*. Our 16S sequences are 100% identical to *D. blanfordii* specimens in GenBank (AB023751) from Thailand and Peninsular Malaysia (Honda et al. 1999).

###### Specimens examined.

USNM 587024–026.

###### Red List status.

NE.

#### Gekkonidae – geckos (Suppl. material 1: Figs 9–10)

##### Gekko gecko

Taxon classificationAnimaliaGekkoGekkonidae

(Linnaeus, 1758)

###### Description.

Adult male 179 mm SVL, 111 mm TailL (half regenerated), 81.7 mm TrunkL, 19.7 ForeaL, 24.3 mm CrusL, 43.9 mm HeadL, 35.4 mm HeadW, 17.9 mm SnEye, 13.7 mm NarEye, 16.4 mm SnW. Adult proportions 46% TrunkL/SVL, 34% SnFor/SVL, 41% ForeaL/CrusL, 18% CrusL/SVL, 24% HeadL/SVL (23.0±1.0), 81% HeadW/HeadL, 41% SnEye/HeadL, 43% EyeEar/HeadL.

Head and trunk scalation predominantly granular, enlarged scales bordering the mouth, 13 supralabials to rictus, 10 infralabials; 96 scales at midbody, 148 scales ventrally from mental to vent border; 21 enlarged lamellae on 4^th^ finger, 20 on fourth toe.

###### Natural history notes.

This specimen derived from Yeybu village; others were heard in the residual forest at the July slash and burnt site.

###### General Distribution.

Widespread, Nepal and Northeast India to South China southward into Lesser Sundas.

###### Molecular Data.

Our specimen was placed in its own COI BIN and was between 94.5–96.3% similar to other *G. gecko* COI in GenBank. Our sequence was placed in a clade at the base of the *G. gecko* clade; these basal diverging clades show substantial sequence divergence, indicating this may represent a species complex.

###### Specimens examined.

USNM 587027.

###### Red List status.

NE.

##### Hemidactylus berdmorei

Taxon classificationAnimaliaSquamataGekkonidae

(Blyth, 1853)

###### Comments.

No species of the *Hemidactylus bowringii* group were seen at any of the sites visited. *Hemidactylus berdmorei* is known only from a disintegrating holotype collected in 1853 in Mergui (= Myeik), hence vouchers of the Smooth Gecko from this area are essential for resolving the taxonomic status of this named taxon (McMahan and Zug 2007). If it is an autochthonous species, this gecko is likely a valid species; however, because Mergui was an active seaport in the mid 19^th^ century, it is possible that the specimen was a recent arrival from elsewhere in Asia and did not become established.

###### Red List status.

NE.

##### Hemidactylus frenatus

Taxon classificationAnimaliaSquamataGekkonidae

Duméril & Bibron, 1836

###### Description.

Adult females (*n* = 2) 52.1–53.2 mm SVL, 51–37 mm TailL; both regenerated; adult male (*n* = 1) 54.9 mm SVL; 25.3 mm TailL; 23.8 mm HeadL; 43–49% TrunkL/SVL, 13–15% CrusL/SVL, 25–26% HeadL/SVL, 38–39% HeadW/HeadL, 64–66% HeadH/HeadL, 42–44% SnEye/HeadL, 29–33% EyeEar/HeadL.

###### Natural history notes.

A synanthrope. Collected on the outside wall of the hotel in Myeik.

###### General Distribution.

Widespread human commensal, worldwide in subtropics and tropics.

###### Molecular Data.

Three specimens of *H. frenatus* were collected in Myeik. They were placed in two COI BINs, two (USNM 587030 and 587032) in their own BIN, and USNM 587031 was placed in the same BIN with 18 other *H. frenatus* from Honduras. Our specimens were placed with other specimens in GenBank from the “Myanmar clade” of *H. frenatus* from Tonione et al. (2011). Two other specimens were placed together, on a long branch, sister to all other *H. frenatus*. These specimens were subsequently identified as *H. tenkatei* (see below).

###### Natural history notes.

Individuals collected from the outside wall of a hotel.

###### Comments.

The House Gecko is a widespread and invasive species. It seldom occurs on vegetation away from human buildings. The tissue gathered from this small sample and barcode analyzed reveals that the Myeik population contains two genetic lineages.

###### Specimens examined.

USNM 587030–032.

###### Red List status.

LC.

##### Hemidactylus garnotii

Taxon classificationAnimaliaSquamataGekkonidae

Duméril & Bibron, 1836

###### Description.

Adult female 59.0 mm SVL. 14.6 mm HeadL; 47% TrunkL/SVL, 14% CrusL/SVL, 25% HeadL/SVL, 79% HeadW/HeadL, 34% HeadH/HeadL, 47% SnEye/HeadL, 28% EyeEar/HeadL, 14% SnW/HeadL.

###### Natural history notes.

A synanthrope.

###### General Distribution.

Widespread human commensal, native to South Asia and Pacific islands.

###### Molecular Data.

Our specimen was placed in a COI BIN with three other *H. garnotii*, and two *H. stejnegeri*. There are currently no COI nor 16S sequences available in GenBank for *H. garnotii*. Ours is 100% identical to two other sequences in BOLD, not publicly available (from New Caledonia). However, these are also identical to two *H. stejnegeri* in BOLD, also not publicly available (from Vietnam and the United States).

###### Comments.

The Fox Gecko is an all-female species with a broad distribution in Asia and the Pacific. Of all invasive *Hemidactylus*, it regularly occurs in the vegetation of disturbed habitats rather than on human buildings.

###### Specimens examined.

USNM 587033.

###### Red List status.

NE.

##### Hemidactylus tenkatei

Taxon classificationAnimaliaSquamataGekkonidae

Lidth de Jeude, 1895

###### Description.

Adult males (*n* = 2) 60.8–61.3 mm SVL, 43–30 mm TailL both regenerated; 15.7–15.8 mm HeadL; 44–45% TrunkL/SVL, 80–87% Forearm/CrusL, 26% HeadL/SVL, 75–76% HeadW/HeadL, 36–39% HeadH/HeadL, 13% SnEye/HeadL, 28–31% EyeEar/HeadL. .

###### Natural history notes.

Collected on the outside wall of the hotel in Myeik.

###### General Distribution.

Myanmar, West Malaysia, Timor; although likely more widespread in South Asia.

###### Molecular Data.

Two specimens initially thought to be *H. frenatus* were placed at the base of the *H. frenatus* COI tree (see above), these were each placed in their own COI BIN. We then sequenced the ND2 locus (GenBank MG948675 and MG944816) for these individuals to align with the sequences from Kathriner et al. (2014). Our specimens were each placed in one of the *H. tenkatei* clades of Kathriner et al. (2014). See Suppl. material 1: Fig. 10 for the ND2 tree.

###### Comments.

Kathriner et al. (2014) have demonstrated that the Be Burmese specimens of this taxon from Yangon and Tanintharyi associate genetically with *H. tenkatei* from Timor and other Sundan areas.

###### Specimens examined.

USNM 587028–29.

###### Red List status.

NE.

#### Lacertidae – Grass lizards

##### Takydromus sexlineatus

Taxon classificationAnimaliaSquamataLacertidae

Daudin, 1802

Long-tailed Grass Lizard Fig. 4E

###### Description.

Small adult female 48.2 mm SVL, 94 mm TailL tip regenerated; 10.8 mm HeadL; 44% TrunkL/SVL, 12% CrusL/SVL, 22% HeadL/SVL, 52% HeadW/HeadL, 38% HeadH/HeadL, 45% SnEye/HeadL, 29% SnW/HeadL.

Head with 7 supralabial scales, 5^th^ very large and beneath eye, 6 infralabials; 34 dorsal scales at nape, midbody, and above vent; 17 enlarged lamellae on 4^th^ finger, 20 on fourth toe. In preservative, dark above and white below, dorsal ground color medium olive with lighter olive dorsolateral stripe from snout onto base of tail, medium brown loreal stripe from snout through eye to inguina and border below by white stripe from snout tip across supralabials onto trunk, fading at two-third of trunk.

###### Natural history notes.

This species was seen in the weedy fencerows of the June gardens survey and subsequently at the July slash & burnt site. The June voucher specimen is gravid with large yolked but unshelled follicles.

###### General Distribution.

Widespread, Northeast India through southern China to Taiwan and southward into Greater Sunda Islands.

###### Molecular Data.

Our specimen was placed in its own COI BIN, it is 3.0–7.7% sequence divergence (COI) from four specimens in BOLD. Three of those were mined from GenBank (AY248546–48), with no locality data available.

###### Specimens examined.

USNM 587034

###### Red List status.

LC

#### Scincidae – skinks

##### Eutropis macularia

Taxon classificationAnimaliaSquamataScincidae

(Blyth, 1853)

###### Description.

Two adult females 59.4 (crushed), 61.8 mm SVL, incomplete, 83 mm regenerated TailL; NA, 12.6 mm HeadL; 45–53% TrunkL/SVL, 37–42% HindlL/SVL, NA, 20% HeadL/SVL, NA, 84% HeadW/HeadL, 51% HeadH/HeadL, NA, 47% SnEye/HeadL, NA, 32% EyeEar/HeadL. Supralabials 7, 5^th^ largest and beneath eye, 7 or 8 infralabials; 34, 36 dorsal scale rows from nape to above vent, dorsal scales 5 to 7 keeled, predominately 7 keels; 30, 32 scales around midbody; 11 fourth finger lamellae, 14 fourth toe lamellae. In preservative, dark above and dusky below; dorsum medium reddish brown from snout onto tail, laterally lighter reddish brown broad stripe from snout to hind limbs, bordered above by tannish stripe from snout to mid trunk and below by white stripe from snout across supralabials to anterior trunk.

###### Natural history notes.

Seen on the banks of a forest stream.

###### General Distribution.

Widespread, Pakistan through northern peninsular India to Southeast Asia and northern West Malaysia.

###### Molecular Data.

Our specimens were placed in their own COI BIN and are 15–16% divergent from six *E. macularia* in BOLD from Vietnam (not public). The 16S sequences are 98–99% similar to two specimens in GenBank (AY159078, KX231450) from Myanmar, Ayeyarwady Region (CAS 212475) and Tanintharyi, Dawei (CAS 247949) and were placed in the same clade as these individuals. It is likely that the Myanmar and Vietnam populations represent different species.

###### Comments.

This taxon likely contains multiple cryptic species (Barley et al. 2015).

###### Specimens examined.

USNM 587035–036

###### Red List status.

NE.

##### Eutropis multifasciata

Taxon classificationAnimaliaSquamataScincidae

(Kuhl, 1820)

Common Sun Skink Fig. 4D

###### Description.

Immature male 60.8 mm SVL, 82 mm regenerated TailL; 27.2 mm HeadL; 45% TrunkL/SVL, 41% HindlL/SVL, 23% HeadL/SVL, 65% HeadW/HeadL, 42% HeadH/HeadL, 40% SnEye/HeadL, 30% EyeEar/HeadL. Supralabials 7, 5^th^ largest and beneath eye, 7 infralabials; 47 dorsal scale rows from nape to above vent, dorsal scales tricarinate; 32 scales around midbody; 13 fourth finger lamellae, 19 fourth toe lamellae. In preservative, dark above and dusky white below; scattered small white spots laterally between ear and forelimb.

###### Natural history notes.

Specimens were seen in both primary and secondary forest.

###### General Distribution.

Widespread, Northeast India through southern China to Taiwan southward into Sundanese Indonesia and Philippines.

###### Molecular Data.

Our specimen was placed in its own COI BIN and is 3.65–7.9% sequence divergence from many other specimens in BOLD, ranging from Vietnam to Indonesia. Our 16S sequence is 98–99% similar to specimens in GenBank, including CAS 212916 from Ayeyarwady Region, Myanmar (erroneously reported as “CAS 2120916” in GenBank), and was placed in a 16S clade with other *E. multifasciata*. There are several clades in the 16S tree, with deep divergences (~8%), indicating that this represents a species complex in need of revision.

###### Specimens examined.

USNM 587037.

###### Red List status.

NE.

##### Sphenomorphus indicus

Taxon classificationAnimaliaSquamataScincidae

(Gray, 1853)

###### Natural history notes.

Seen in the slash & burnt site.

###### General Distribution.

Widespread, northern peninsular India and Nepal eastward to southern China southward into Myanmar and Southeast Asia.

###### Molecular Data.

No molecular data available.

###### Comments.

There are no vouchers to confirm this field identification. Because these specimens were observed in the slash & burnt site versus the forest and lack vouchers, we tentatively accept the field identification.

###### Specimens examined.

Field observation by Myint Kyaw Thura; captured for confirmation of identification and then released.

###### Red List status.

NE

##### Sphenomorphus maculatus

Taxon classificationAnimaliaSquamataScincidae

(Blyth, 1853)

###### Description.

Two adult males 49.9–54.7 mm SVL, 97–98 regenerated mm SVL; 11.5–12.2 mm HeadL; 45–48% TrunkL/SVL, 54–55% HindlL/SVL, 22–23% HeadL/SVL, 62–63% HeadW/HeadL, 44–46% HeadH/HeadL, 39–40% SnEye/HeadL, 33–34% EyeEar/HeadL.

###### Natural history notes.

Seen and captured among forest leaf litter.

###### General Distribution.

Widespread, Nepal to western China southward through Myanmar and Southeast Asia into Peninsular Malaysia.

###### Molecular Data.

Our COI sequences are 7.1–7.5% divergent from specimens in BOLD from Vietnam (not publicly available). Our 16S sequences are 100% identical to a specimen in GenBank (AB028821) from Kaeng Krachan, Thailand (Honda et al. 2000b) and were placed with this individual and other *S. maculatus* in GenBank.

###### Specimens examined.

USNM 587038–039.

###### Red List status.

NE.

#### Varanidae – monitor lizards

##### Varanus rudicollis

Taxon classificationAnimaliaSquamataVaranidae

(Gray, 1845)

###### Description.

Sex unknown, 57.0 cm SVL, 70.0 cm TailL.

###### General Distribution.

Southern Myanmar and Thailand through Malay Peninsula into Greater Sunda Islands.

###### Molecular Data.

No molecular data available.

###### Comments.

Captured by a villager (Yeybu); photographed; likely subadult or adult.

###### Specimens examined.

USNM Herp Image 2891

###### Red List status.

NE; CITES II.

### Snakes

#### Pythonidae – pythons

##### Malayopython reticulatus

Taxon classificationAnimaliaSquamataPythonidae

(Schneider, 1801) Reticulated Python

Fig. 5A

###### Description.

Female, maturity uncertain, 119 cm SVL, 18.5 cm TailL.

**Figure 5.**
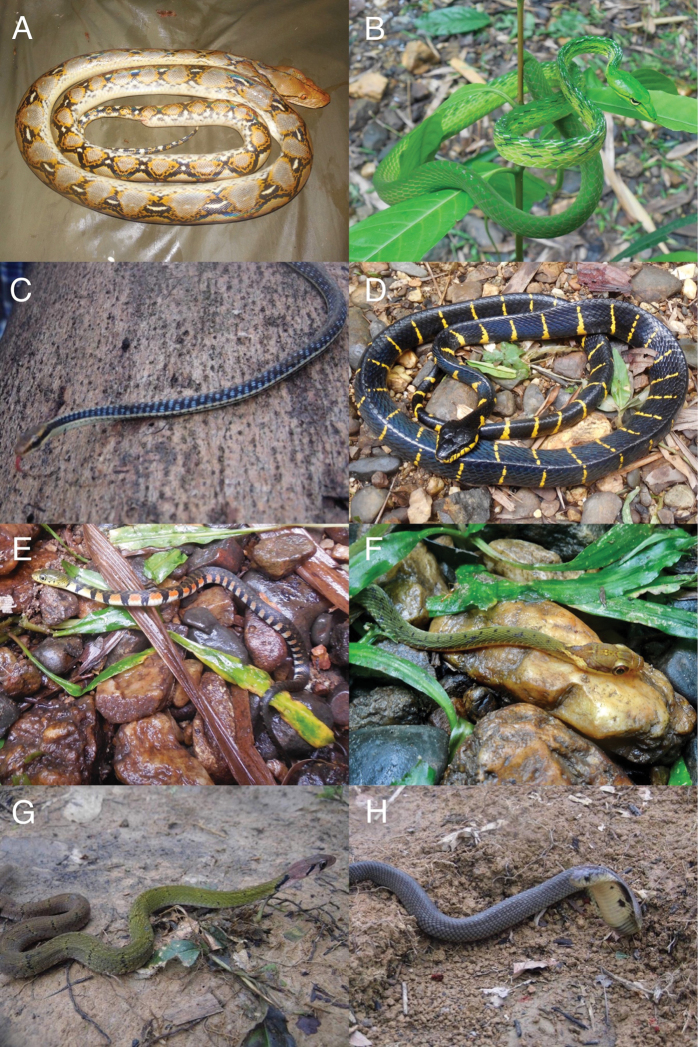
Selected snakes found during this study’s expedition. **A** *Malayopython reticulatus* (USNM HerpImage 2892) **B** *Ahaetulla mycterizans* (USNM 587040) **C** *Dendrelaphis pictus* (USNM HerpImage 2893) **D** *Boiga dendrophila* (USNM 587041) **E** *Xenochrophis trianguligerus* (USNM 587045) **F** *Rhabdophis chrysargos* (USNM 587044) **G** *Rhabdophis nigrocinctus* (USNM HerpImage 2894) **H** *Naja kaouthia* (USNM HerpImage 2895). Photos **A–B, D–F** by Daniel G. Mulcahy, **C, H**–**I** by Myint Kyaw Thura.

###### Natural history notes.

The specimen was collected in a fisherman’s net, in the stream, near Camp 1.

###### General Distribution.

Widespread, Bangladesh through Southeast Asia to Philippines and the Maluku Islands.

###### Molecular Data.

No molecular data available.

###### Comments.

We note that the genus name *Broghammerus* is not nomenclaturally available for the Reticulated Python, because it was promulgated in a herpetological blog, which did not and does not meet the International Nomenclatural Code’s criteria for the valid establishment of formal taxonomic names. A recent study (Reynolds et al. 2014) has proposed the name *Malayopython* for the clade containing the *M. reticulatus* and *M. timoriensis*.

###### Specimens examined.

Specimen remained in FFI field office in Yangon, but it was subsequently destroyed.

###### Specimens examined.

USNM Herp Image 2892

###### Red List status.

NE; CITES II.

#### Colubridae – colubrid snakes

##### Ahaetulla mycterizans

Taxon classificationAnimaliaSquamataColubridae

(Linnaeus, 1758)

Malayan Vinesnake Fig. 5B

###### Description.

Adult female 745 mm SVL, 385 TailL in life; dorsal scales in 15-15-13 rows, ventrals 190, 148 paired subcaudals with undamaged tip; single precloacal (anal) scale. Snout is blunt, rostral scale truncate anteriorly; 2 loreal scales on right, 1 left, each a small lanceolate scale, isolated in suture between the supralabials and internasal and prefrontal scales; internasal (dorsal surface of snout) flat anteriorly and convex posteriorly. In preservative, the head and nuchal area are bright green dorsally and laterally; trunk gradually become darker green and at about one-third length is olive to tip of tail; laterally trunk pale green to narrow white ventrolateral edge of upturned ventral scales (forming longitudinal stripe), then bordered medially by narrow dark green longitudinal stripe; white stripe becomes yellow by midbody and continues yellow onto base of tail; green stripe disappears 15–20 ventrals anterior to the vent; remainder of venter is white from tip of chin to about midbody then becoming greenish yellow continuing onto tail.

###### Natural history notes.

Collected at 1000 hours near Yeybuchaung-ngal stream, approximately 100 meters downstream from Camp 1.

###### General Distribution.

Tanintharyi southward through Malaya Peninsula to Java and Sumatra.

###### Molecular Data.

There are no other COI sequences in BOLD for *A. mycterizans*. Our specimen was placed in its own COI BIN and the 16S is 99% similar to other *A. mycterizans* in GenBank (KX660161, KX660205; no locality data provided) and was placed in a 16S clade with these specimens and an “*A. prasina*” (FMNH 269042 from Borneo) that is likely misidentified (KX660195).

###### Comments.

This species was an unsuspected find, because it had not been reported previously from Myanmar and the closest Thai records are in southern Thailand south of the Isthmus of Kra. This specimen represents the northernmost record of this species (see Lee et al. 2015). A second specimen (CAS 247859) was confirmed (JLL) further south from Kawthaung, Tanintharyi Region, Myanmar previously identified as *A. prasina*.

###### Specimens examined.

USNM 587040.

###### Red List status.

LC.

##### Boiga dendrophila

Taxon classificationAnimaliaSquamataColubridae

(Boie, 1827)

Mangrove Cat Snake Fig. 5D

###### Description.

Adult female 1450 mm SVL, 362 mm TailL in life; dorsal scales in 21-21-17 rows, ventrals 225, subcaudals 96 with single precloacal scale; 8 supralabials, 3^rd^, 4^th^ & 5^th^ touch eye; 44 lateral white bars on trunk from neck to vent, venter become entirely black at ventral 126. Adult male 1450 mm SVL, 350 mm TailL in life; dorsal scales in 21-23-17 rows, ventrals 211, subcaudals 91 with single precloacal scale; 8 supralabials, 3^rd^, 4^th^ & 5^th^ touch eye; 42 lateral white bars on trunk from neck to vent, venter becomes entirely black at ventral 84.

###### Natural history notes.

Collected in primary rainforest along a small tributary to Yeybuchaung-ngal downstream from Camp 1.

###### General Distribution.

Widespread, Tanintharyi to Greater Sunda and Philippine Islands.

###### Molecular Data.

Our COI sequences are 1% different (and placed in the same BIN) from two specimens in BOLD (currently private), from Nakhon Si Thammarat Province, Thailand, south of the Isthmus of Kra. There are currently no other 16S sequences available for *B. dendrophila*.

###### Comments.

This species was also an unsuspected find owing to the absence of previous records for Myanmar; the closest Thai records are from southern Thailand south of the Isthmus of Kra (see Lee et al. 2015). The morphology of these specimens matches that of the subspecies *B. dendrophila melanota* (Boulenger, 1896).

###### Specimens examined.

Adult female, adult male (USNM 587041–042, respectively).

###### Red List status.

NE.

##### Boiga drapiezii

Taxon classificationAnimaliaSquamataColubridae

(Boie, 1827)

###### Description.

Adult female 1340 mm SVL, 380 mm TailL in life; dorsal scales in 19-19-15 rows, ventrals 279, subcaudals 144 with single precloacal scale; 8 supralabials, 3^rd^, 4^th^ & 5^th^ touch eye. 48 dark brown lateral blotches from neck to above vent, venter tan heavily dusted with brownish gray.

###### Natural history notes.

Collected at 1900–2000 hours in primary rainforest near Yeybuchaung-ngal stream, approximately 100 meters upstream from Camp 1 (N 12.43413’, E 99.14505’) at an elevation of 179 meters ASL The female is gravid, bearing four shelled eggs, each ~34 mm long, 19 mm diameter.

###### General Distribution.

Tanintharyi and Thai-Malay Peninsula through Greater Sundas to Philippines.

###### Molecular Data.

There are currently no other available COI sequences for *B. drapiezii*, it was placed in its own BIN. Our 16S sequence is 99% similar to two *B. drapiezii* in GenBank (KX660209–10; no locality data provided).

###### Comments.

This species was also an unsuspected find owing to the absence of previous records for Myanmar and the closest Thai records in southern Thailand south of the Isthmus of Kra. Two unreported specimens (CAS 247770, CAS 247864) were collected further south in Tanintharyi 40–60 km north of Kawthung (see Lee et al. 2015).

###### Specimens examined.

USNM 587043.

###### Red List status.

LC.

##### Dendrelaphis pictus

Taxon classificationAnimaliaSquamataColubridae

(Gmelin, 1789)

Painted Bronzeback Fig. 5C

###### Description.

Not sexed or measured; field identification.

###### Natural history notes.

From disturbed areas.

###### General Distribution.

Bangladesh and Northeast India through southern Myanmar to northern West Malaysia.

###### Molecular Data.

No molecular data available.

###### Comments.

Field observation by Myint Kyaw Thura; captured for confirmation of identification, photographed and then released.

###### Specimens examined.

USNM Herp Image 2893

###### Red List status.

NE.

#### Natricidae – water snakes

##### Rhabdophis chrysargos

Taxon classificationAnimaliaSquamataNatricidae

(Schlegel, 1837)

Speckle-bellied Keelback Fig. 5F

###### Description.

Juvenile, not sexed, 258 mm SVL, 88 mm TailL; dorsal scales in 17-15-15 rows, ventrals 168, subcaudals 112 with divided precloacal scale; 9 supralabials, 4^th^, 5^th^ & 6^th^ touch eye. 12–14 anterior maxillary teeth, 2 or 3 short ones, then 2 to 3 slightly enlarge posterior maxillary teeth. White nuchal chevron with arms extending onto supralabials to beneath eye; dorsum and sides of trunk medium olive brown with lateral series of small, faint light spots; venter immaculate white.

###### Natural history notes.

Collected at Camp 1 along the Yeybuchaung-ngal stream.

###### General Distribution.

Southern Southeast Asia through Sundas to Philippines.

###### Molecular Data.

Our COI sequence was placed in its own BIN and is 5.3% divergent from a specimen in BOLD (private) from Vietnam. There are currently no other COI sequences are available at this time. There are currently no other 16S sequences with which to compare.

###### Comments.

*Rhabdophis chrysargos* occurs widely through the southern half of Southeast Asia including the Greater Sunda Islands, the Philippines and Peninsular Malaysia. All records from Myanmar are from the Tanintharyi (Dowling and Jenner 1988).

###### Specimens examined.

USNM 587044.

###### Red List status.

LC.

##### Rhabdophis nigrocinctus

Taxon classificationAnimaliaSquamataNatricidae

(Blyth, 1856)

Banded Green Keelback Fig. 5G

###### Description.

Not sexed or measured; field identification.

###### Natural history notes.

Seen in the slash & burnt site.

###### General Distribution.

Southeast Asia, Myanmar and Yunnan, China.

###### Molecular Data.

No molecular data available.

###### Comments.

Field observation by Myint Kyaw Thura; captured for confirmation of identification, photographed, and then released.

###### Specimens examined.

USNM Herp Image 2894

###### Red List status.

LC.

##### Xenochrophis piscator

Taxon classificationAnimaliaSquamataNatricidae

(Schneider, 1799)

###### Description.

Juvenile 225 mm SVL, 101 mm TailL; 131 ventrals, 89 subcaudals, 19-19-17 dorsals; adult male 515 mm SVL, 261 mm TailL; 130 ventrals, 93 subcaudals, 19-19-17 dorsals. In both specimens a single rectangular loreal, single preocular, 3 postoculars, 9 supralabials with the 4^th^–5^th^ touching eye, 10 infralabials. No data recorded for the individual observed in the slash & burnt area. Dorsum olive-brown, gray-brown with rows of darker rectangular checkered blotches, “V” nuchal mark on the head. Venter plain, lighter, dark fringes on ventrals absent, but some speckling along the margins.

###### Natural history notes.

Occurs in ponds and streams in human-impacted areas.

###### General Distribution.

Widespread, Pakistan to Southeast Asia and southern China.

###### Molecular Data.

There are currently no other COI sequence for *X. piscator* available for comparison. The closest COI sequences available are from *X. flavipunctatus* and they are more than 10% divergent. Our 16S sequences are 4% different from a *X. piscator* in GenBank (KX277271; no locality data provided).

###### Comments.

Vogel & David (2012) did not record this species from the Tanintharyi. However, recent records deposited from CAS exist from Dawei. These two specimens extend the distribution of this species ~175 km due south. *Xenochrophis flavipunctatus* should also occur here, but as yet its presence has not been confirmed. *Xenochrophis piscator* may represent a species complex, as significant morphological variation occurs in different populations, especially those in India and Sri Lanka (Vogel and David 2012).

###### Specimens examined.

USNM 587046 (adult)–587047 (juvenile).

###### Red List status.

NE.

##### Xenochrophis trianguligerus

Taxon classificationAnimaliaSquamataNatricidae

(Boie, 1827)

Red-sided Keelback (Fig. 5E)

###### Description.

Not sexed, juvenile specimen 245 mm SVL, 95 TailL; 225 ventrals, 96 subcaudals, 19-19-15 dorsals; single loreal rectangular, preocular single, 3 postoculars. Dorsum olive brown, dark rectangular blotches laterally with the anterior portion of the sides yellow and red, fading in coloration towards the midbody. Head hued with blue, dark sutures on some of the supralabials.

###### Natural history notes.

Discovered streamside in the primary forest.

###### General Distribution.

Northeast India to Southeast Asia through Malay Peninsula into Greater Sunda Islands.

###### Molecular Data.

There are currently no other COI nor 16S sequences available for *X. trianguligerus* with which to compare.

###### Comments.

Earlier country-wide herpetofaunal surveys of Myanmar indicate that *X. trianguligerus* occurs only in Tanintharyi.

###### Specimens examined.

USNM 587045.

###### Red List status.

LC.

#### Elapidae – cobras and kraits

##### Naja kaouthia

Taxon classificationAnimaliaSquamataElapidae

(Lesson, 1831)

Monocled Cobra (Fig. 5H)

###### Description.

Juvenile, not sexed or measured; field identification.

###### Natural history notes.

Found in human-disturbed habitats. Field observation at Forest 3 site by Myint Kyaw Thura; photographed for confirmation of identification and then released.

###### General Distribution.

Widespread, Nepal and northern peninsular India to Southeast Asia and southern China.

###### Molecular Data.

No molecular data available.

###### Specimens examined.

USNM Herp Image 2895

###### Red List status.

LC; CITES II.

## Discussion

### DNA Barcode data

The DNA barcode data greatly improved our estimate of species numbers in the Tanintharyi, within anurans in particular, similar to another recent study using DNA barcode data for estimating species diversity (Diechmann et al. 2017). The placement of sequences from individual specimens of the same genus into multiple COI BINs indicated it was likely that multiple species were collected. For instance, collections at the edge of the forest and on the Tanintharyi River floodplain, at the eastern edge of Yebu Village, yielded multiple anuran specimens identified initially as four morphospecies in four genera (*Polypedates*, *Microhyla*, *Fejervarya*, and *Occidozyga*). However, DNA barcoding and comparisons with our northern Myanmar reference material revealed each genus was likely represented by two to three species. We determined this based on the fact that at least one of the Tanintharyi clades (within each genus) grouped with specimens from the north, rather than with the other clades (of the respective genus) in the Tanintharyi. This increased our total number of species from four to ten. We note that the short sequence data from DNA barcode data, while useful for determining relationships among closely related groups, such as these cases of populations within genera, falls short at resolving higher-level relationships. For instance, even our combined dataset (COI + 16S) fails to recover several families as monophyletic, such as Microhylidae and Dicroglossidae, and even fails to recover some genera as monophyletic, such as *Xenochrophis* (Fig. 2). Whereas analyses with more complete taxonomic sampling, more loci, and more robust analyses recover these families as monophyletic (e.g. de Sá et al. 2012).

The DNA barcode data allowed us to identify several cryptic species of anurans in multiple genera and families, including Rhacophoridae: *Polypedates* – two species in the Tanintharyi, and a third in the north; Microhylidae: *Microhyla* – three species in the Tanintharyi, all of those and a three more species in the north; Dicroglossidae: *Fejervarya* – two species in the Tanintharyi, and two in the north; and *Occidozyga* – three species in the Tanintharyi, one of the same and three additional species in the north. Likewise, we were able to determine the number of species present at the forest sites based on the COI barcode data, in the family Dicroglossidae: *Limnonectes* – three species in the Tanintharyi, two of the same and a fourth in the north; the family Microhylidae: *Kaloula* – two species in the Tanintharyi, one also occurring in the north; Ranidae: *Hylarana* – one species in the Tanintharyi and the north, and three additional species in the north; *Odorrana* – two species in the Tanintharyi, one of these and a third in the north. Because a comprehensive COI barcode library is lacking for southeast Asian anurans, we relied on 16S sequence data as a supplemental barcode marker to help identify specimens to species based on comparisons with known material published in GenBank. The inclusion of the 16S data allowed us to compare our specimens with published material in GenBank, and enabled us to identify several specimens to named species (e.g. *Limnonectes blythii*, *L. doriae*, *L. limborgi*, and *L. longchuanensis*, *Microhyla berdmorei*, *M. butleri*, *M. fissipes*, and *M. heymonsi*), including several recently described species (*Hoplobatrachus litoralis*, *Kaloula latidisca*), and some recently identified, but not formally described, species such as *Fejervarya* sp. ‘hp2–3’ (Kotaki et al. 2010), *Fejervarya* sp. BFL 2007 (Islam et al. 2008, Hasan et al. 2012a), and *Sylvirana* sp. C ‘MS-2010’ (Hasan et al. 2012a), as well as some of our own new discoveries (*Fejervarya* sp. A, *Occidozyga* spp. A–D, *Leptolalax* sp. A, *Microhyla* spp. A–B, and *Chiromantis* sp. A). The use of the DNA barcode database (BOLD) allows us to “BIN” these un-named species, such that researchers conducting future expeditions can compare their specimens to ours to determine if they are the same un-named species, “known unknowns”, or yet newly discovered un-named species “unknown unknowns” (Collins and Cruickshank 2014). Traditional, morphological species descriptions need to follow in order to properly assess the biodiversity of this region. However, this process can be slow, requires taxonomic expertise, and is less supported by many academic institutions. Given the high rate of putative cryptic species, particularly among anuran genera, we issue caution when using morphological identifications alone, as in the Reserve Forest areas presented in this study. The fact remains that guide books of the region (SE Asia) that attempt to include morphological identifications contain overlapping character descriptions (e.g. Grismer 2011), or lack identifications altogether (e.g. Koch 2012). Once more robust taxonomic treatments of each group are conducted, more reliable morphological diagnoses may become available.

A recent study suggests that species delimitations based solely on mtDNA may be misleading, and over-estimating species in biodiversity studies (Chan et al. 2017). We maintain that our method, preliminary as it is because it acts as a triage assessment, is still a valid method in rapid biodiversity surveys for a number of reasons. First of all, it may be better to over-estimate species diversity in rapid biodiversity surveys, rather than under-estimating in order to secure proper protection of the area. Secondly, this method may be valid because estimates made using this method may be correct and documenting the accurate number of species. Thirdly, even if species numbers are over-estimated, recognizing several lineages (or operational taxonomic units, OTUs) at a minimum recognizes the genetic diversity in a lineage (if they are later determined to represent the same species), and is thus beneficial from a conservation-genetics point of view. And fourthly, rapid assessments identifying potential cryptic species can direct future research to taxonomic groups in need of further investigation. It is largely for this reason that we recommend the use of place-holder names, such as “sp. A” until more in-depth investigations can be conducted, including additional taxonomic and geographic sampling, and additional markers (e.g. nuclear). In reality, the forests may disappear before such in-depth analyses can be conducted, as each group (e.g. genus) may require essentially a dissertation chapter’s-worth of work (e.g. Chan et al. 2017), and for the number of groups covered in this report, for example, may take over a decade to complete, by which time the forests could be gone (Connette et al. 2017).

### Important absences and presences in the Tenasserim

Our brief surveys are inadequate to address the presence or absence of all potential members of the southern Tanintharyi herpetofauna. Studies of the herpetofauna of the Myanmar Central Dry Zone at the Chatthin Wildlife Sanctuary (Zug et al. 1998, Zug 2011) had a team of four regular members with the assistance of the entire Sanctuary staff. Even with all these eyes and hands and with weekly transect surveys and monthly drift-fence trapping, 40–41 weeks were required to record 90% of the Chatthin herpetofauna, and previously undocumented species were still being discovered in the third and final year of that survey. We especially note that nine species of turtles have been recorded on the Thailand side of the Tanintharyi mountain range. Crocodilians were not expected owing to shallowness of the streams in the immediate area of the survey.

At this stage of our inventory of the Tanintharyi proposed National Park and its environs, we wish to emphasize the discovery of taxa previously undocumented for Myanmar. Surprisingly these undocumented taxa include only two amphibians (Ichthyophis cf. kohtaoensis and *Chalcorana eschatia*) and three species of snakes (*Ahaetulla mycterizans*, *Boiga dendrophila*, and *Boiga drapiezii*; see Lee et al. 2015). None of these taxa were reported from Phetchaburi Province, the Thailand province immediately east of our survey site. They all represent species whose primary distribution is south of the Isthmus of Kra; the two *Boiga* were reported in Pauwels et al. (2000) survey of Phang-Nga Province, Thailand.

Some recently described taxa were also detected in Myanmar based on our study, including the likely occurrence of *Kaloula latidisca*, a species recently described from Peninsular Malaysia (Chan et al. 2014). As we included the reference material from more northern Myanmar, we identified species that have not been previously detected in Myanmar. This includes the dicroglossid *Hoplobatrachus litoralis*, a recently described species from Cox’s Bazar district of Bangladesh (Hasan et al. 2012b), which now includes two specimens from the Yangon area and one from Mon State, extending the range of this species from Bangladesh to south-central Myanmar. In total, the formally threatened species according to the IUCN Red List encountered include two anurans (*Ansonia thinthinae*, *Limnonectes blythii)*, and the following represent CITES II species: two turtles (*Indotestudo elongata*), a lizard (*Varanus rudiocolis*), and two snakes (*Naja kaouthia* and *Malayopython reticulatus*).

## Conclusion

The use of DNA barcoding allowed us to determine how many species were present at the site of our biodiversity inventory survey. The inclusion of the supplementary marker 16S allowed us to assign several individuals to named species for which 16S data were available for comparison (whereas the COI reference library is less complete), and to identify others as previously identified, undescribed species. The use of the DNA barcode database (BOLD) allows us to “BIN” these un-named species, such that researchers conducting future expeditions can compare their specimens to ours to determine if they are the same un-named species. Biodiversity research needs more “boots on the ground,” because an incomplete taxonomy hinders our ability to protect biodiversity and guide conservation (Wilson 2017). As we proceed to fill the “BINs of life,” we will eventually be able to record and catalogue all species of life on Earth. We encourage researchers to continue to add to these databases, and most importantly, to update existing records as our knowledge increases.

## Supplementary Material

XML Treatment for Ichthyophis cf. kohtaoensis

XML Treatment for Ansonia thinthinae

XML Treatment for Ingerophrynus parvus

XML Treatment for Phrynoidis asper

XML Treatment for Fejervarya

XML Treatment for Fejervarya

XML Treatment for Limnonectes blythii

XML Treatment for Limnonectes doriae

XML Treatment for Limnonectes limborgi

XML Treatment for Ingerana tenasserimensis

XML Treatment for Occidozyga lima

XML Treatment for Occidozyga martensii

XML Treatment for Occidozyga

XML Treatment for Kaloula latidisca

XML Treatment for Kaloula pulchra

XML Treatment for Microhyla butleri

XML Treatment for Microhyla fissipes

XML Treatment for Microhyla heymonsi

XML Treatment for Amolops panhai

XML Treatment for Chalcorana eschatia

XML Treatment for Hylarana erythraea

XML Treatment for Odorrana hosii

XML Treatment for Odorrana livida

XML Treatment for Sylvirana malayana

XML Treatment for Polypedates cf. leucomystax

XML Treatment for Polypedates mutus

XML Treatment for Indotestudo elongata

XML Treatment for Dogania subplana

XML Treatment for Acanthosaura crucigera

XML Treatment for Calotes emma

XML Treatment for Draco blanfordii

XML Treatment for Gekko gecko

XML Treatment for Hemidactylus berdmorei

XML Treatment for Hemidactylus frenatus

XML Treatment for Hemidactylus garnotii

XML Treatment for Hemidactylus tenkatei

XML Treatment for Takydromus sexlineatus

XML Treatment for Eutropis macularia

XML Treatment for Eutropis multifasciata

XML Treatment for Sphenomorphus indicus

XML Treatment for Sphenomorphus maculatus

XML Treatment for Varanus rudicollis

XML Treatment for Malayopython reticulatus

XML Treatment for Ahaetulla mycterizans

XML Treatment for Boiga dendrophila

XML Treatment for Boiga drapiezii

XML Treatment for Dendrelaphis pictus

XML Treatment for Rhabdophis chrysargos

XML Treatment for Rhabdophis nigrocinctus

XML Treatment for Xenochrophis piscator

XML Treatment for Xenochrophis trianguligerus

XML Treatment for Naja kaouthia

## Supplementary materials

Supplementary material 1 12S and 16S neighbor-joining trees Data type: molecular data This is an open access article distributed under the terms of the Creative Commons Attribution License (CC BY 4.0), which permits unrestricted use, distribution, and reproduction in any medium, provided the original author and source are credited.Mulcahy DG, Lee JL, Miller AH, Chand M, Thura MK, Zug GR
