# Supplementary material for: Filling the BINs of life: Report of an amphibian and reptile survey of the Tanintharyi (Tenasserim) Region of Myanmar, with DNA barcode data
Source: Zookeys. 2018 May 10;(757):85–152. doi: 10.3897/zookeys.757.24453 (PMC5958176; doi:10.3897/zookeys.757.24453)
Supplement: Supplementary material 1 — 12S and 16S neighbor-joining trees [file zookeys-757-085-s001.pdf]

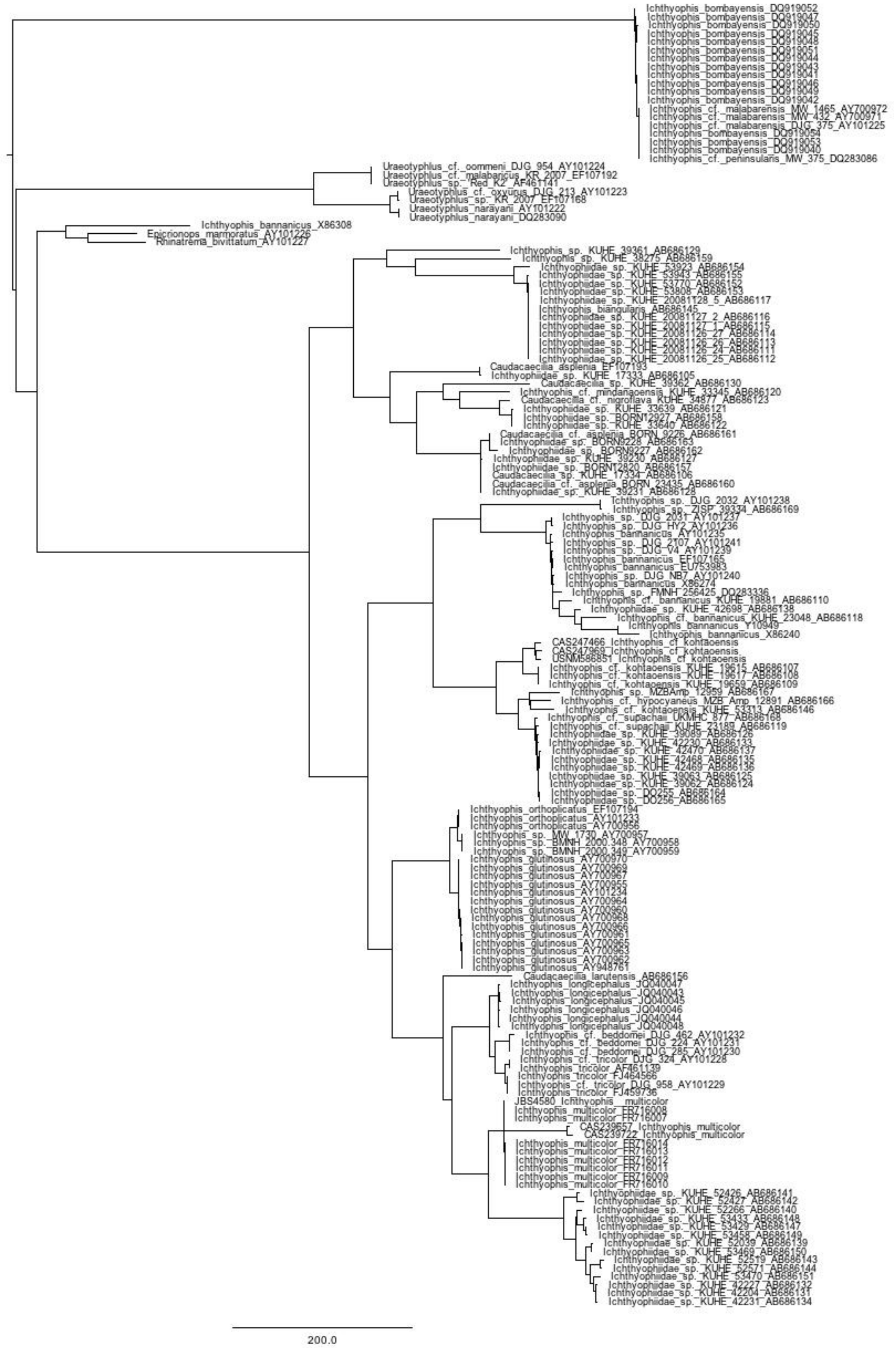

Fig. 1. Ichthyophiidae 12S and 16S neighbor-joining tree.

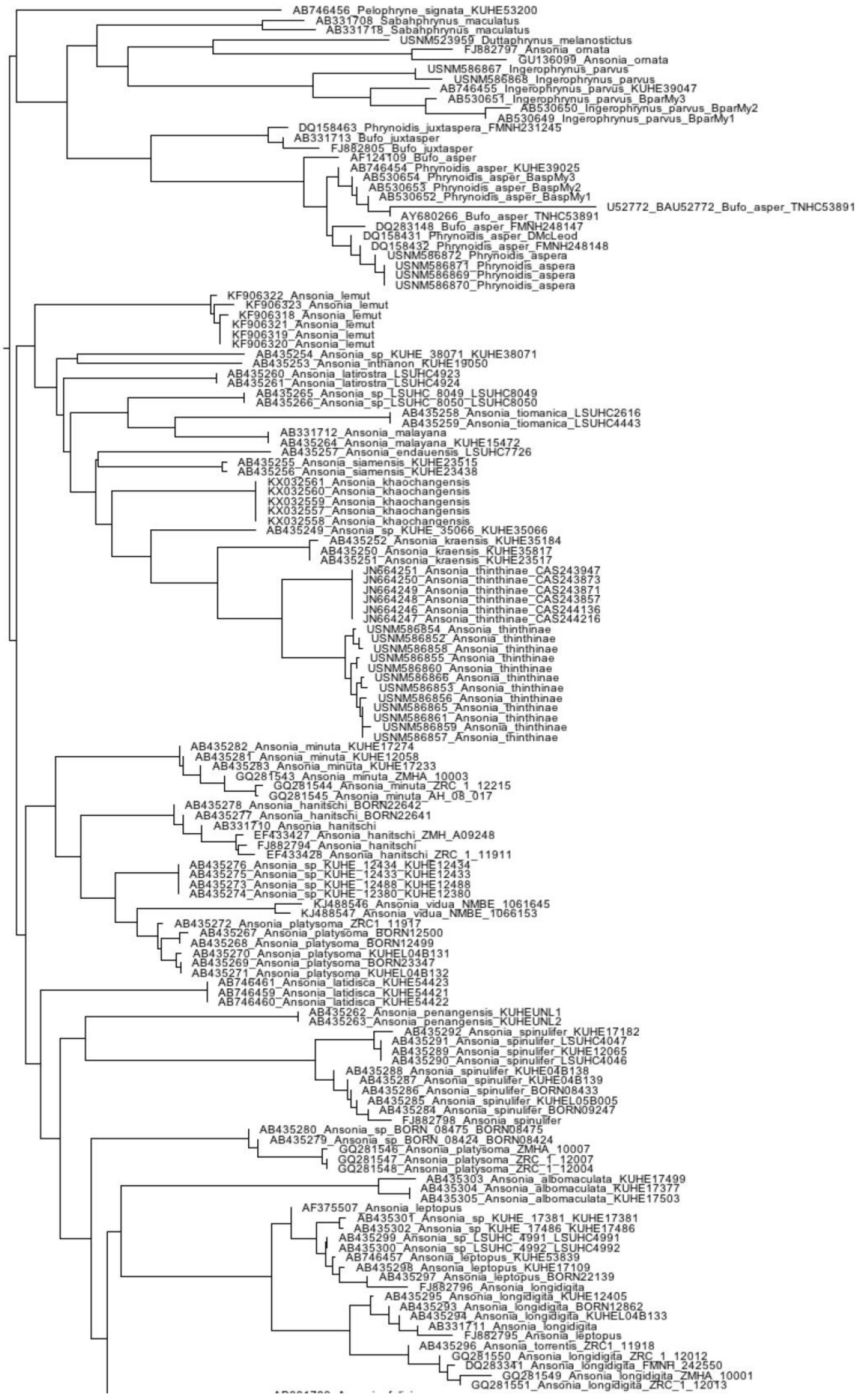
AB746456_Pelophryne_signata_KUHE53200
AB331708_Sabahphrynus_maculatus
AB331718_Sabahphrynus_maculatus
USNM523959_Duttaphrynus_melanostictus
FJ882797_Ansonia_ornata
GU136099_Ansonia_ornata
USNM586867_Ingerophrynus_parvus
USNM586868_Ingerophrynus_parvus
AB746455_Ingerophrynus_parvus_KUHE39047
AB530651_Ingerophrynus_parvus_BparMy3
AB530650_Ingerophrynus_parvus_BparMy2
AB530649_Ingerophrynus_parvus_BparMy1
DQ158463_Phrynoidis_juxtaspera_FMNH231245
AB331713_Bufo_juxtasper
FJ882805_Bufo_juxtasper
AF124109_Bufo_asper
AB746454_Phrynoidis_asper_KUHE39025
AB530654_Phrynoidis_asper_BaspMy3
AB530653_Phrynoidis_asper_BaspMy2
AB530652_Phrynoidis_asper_BaspMy1
U52772_BAU52772_Bufo_asper_TNHC53891
AY680266_Bufo_asper_TNHC53891
DQ283148_Bufo_asper_FMNH248147
DQ158431_Phrynoidis_asper_DMcLeod
DQ158432_Phrynoidis_asper_FMNH248148
USNM586872_Phrynoidis_aspera
USNM586871_Phrynoidis_aspera
USNM586869_Phrynoidis_aspera
USNM586870_Phrynoidis_aspera
KF906322_Ansonia_lemut
KF906323_Ansonia_lemut
KF906318_Ansonia_lemut
KF906321_Ansonia_lemut
KF906319_Ansonia_lemut
KF906320_Ansonia_lemut
AB435254_Ansonia_sp_KUHE_38071_KUHE38071
AB435253_Ansonia_inthanon_KUHE19050
AB435260_Ansonia_latirostra_LSUHC4923
AB435261_Ansonia_latirostra_LSUHC4924
AB435265_Ansonia_sp_LSUHC_8049_LSUHC8049
AB435266_Ansonia_sp_LSUHC_8050_LSUHC8050
AB435258_Ansonia_tiomanica_LSUHC2616
AB435259_Ansonia_tiomanica_LSUHC4443
AB331712_Ansonia_malayana
AB435264_Ansonia_malayana_KUHE15472
AB435257_Ansonia_endauensis_LSUHC7726
AB435255_Ansonia_siamensis_KUHE23515
AB435256_Ansonia_siamensis_KUHE23438
KX032561_Ansonia_khaochangensis
KX032560_Ansonia_khaochangensis
KX032559_Ansonia_khaochangensis
KX032557_Ansonia_khaochangensis
KX032558_Ansonia_khaochangensis
AB435249_Ansonia_sp_KUHE_35066_KUHE35066
AB435252_Ansonia_kraensis_KUHE35184
AB435250_Ansonia_kraensis_KUHE35817
AB435251_Ansonia_kraensis_KUHE23517
JN664251_Ansonia_thinthinae_CAS243947
JN664250_Ansonia_thinthinae_CAS243873
JN664249_Ansonia_thinthinae_CAS243871
JN664248_Ansonia_thinthinae_CAS243857
JN664246_Ansonia_thinthinae_CAS244136
JN664247_Ansonia_thinthinae_CAS244216
USNM586854_Ansonia_thinthinae
USNM586852_Ansonia_thinthinae
USNM586858_Ansonia_thinthinae
USNM586855_Ansonia_thinthinae
USNM586860_Ansonia_thinthinae
USNM586866_Ansonia_thinthinae
USNM586853_Ansonia_thinthinae
USNM586856_Ansonia_thinthinae
USNM586865_Ansonia_thinthinae
USNM586861_Ansonia_thinthinae
USNM586859_Ansonia_thinthinae
USNM586857_Ansonia_thinthinae
AB435282_Ansonia_minuta_KUHE17274
AB435281_Ansonia_minuta_KUHE12058
AB435283_Ansonia_minuta_KUHE17233
GQ281543_Ansonia_minuta_ZMHA_10003
GQ281544_Ansonia_minuta_ZRC_1_12215
GQ281545_Ansonia_minuta_AH_08_017
AB435278_Ansonia_hanitschi_BORN22642
AB435277_Ansonia_hanitschi_BORN22641
AB331710_Ansonia_hanitschi
EF433427_Ansonia_hanitschi_ZMH_A09248
FJ882794_Ansonia_hanitschi
EF433428_Ansonia_hanitschi_ZRC_1_11911
AB435276_Ansonia_sp_KUHE_12434_KUHE12434
AB435275_Ansonia_sp_KUHE_12433_KUHE12433
AB435273_Ansonia_sp_KUHE_12488_KUHE12488
AB435274_Ansonia_sp_KUHE_12380_KUHE12380
KJ488546_Ansonia_vidua_NMBE_1061645
KJ488547_Ansonia_vidua_NMBE_1066153
AB435272_Ansonia_platysoma_ZRC1_11917
AB435267_Ansonia_platysoma_BORN12500
AB435268_Ansonia_platysoma_BORN12499
AB435270_Ansonia_platysoma_KUHEL04B131
AB435269_Ansonia_platysoma_BORN23347
AB435271_Ansonia_platysoma_KUHEL04B132
AB746461_Ansonia_latidisca_KUHE54423
AB746459_Ansonia_latidisca_KUHE54421
AB746460_Ansonia_latidisca_KUHE54422
AB435262_Ansonia_penangensis_KUHEUNL1
AB435263_Ansonia_penangensis_KUHEUNL2
AB435292_Ansonia_spinulifer_KUHE17182
AB435291_Ansonia_spinulifer_LSUHC4047
AB435289_Ansonia_spinulifer_KUHE12065
AB435290_Ansonia_spinulifer_LSUHC4046
AB435288_Ansonia_spinulifer_KUHE04B138
AB435287_Ansonia_spinulifer_KUHE04B139
AB435286_Ansonia_spinulifer_BORN08433
AB435285_Ansonia_spinulifer_KUHEL05B005
AB435284_Ansonia_spinulifer_BORN09247
FJ882798_Ansonia_spinulifer
AB435280_Ansonia_sp_BORN_08475_BORN08475
AB435279_Ansonia_sp_BORN_08424_BORN08424
GQ281546_Ansonia_platysoma_ZMHA_10007
GQ281547_Ansonia_platysoma_ZRC_1_12007
GQ281548_Ansonia_platysoma_ZRC_1_12004
AB435303_Ansonia_albomaculata_KUHE17499
AB435304_Ansonia_albomaculata_KUHE17377
AB435305_Ansonia_albomaculata_KUHE17503
AF375507_Ansonia_leptopus
AB435301_Ansonia_sp_KUHE_17381_KUHE17381
AB435302_Ansonia_sp_KUHE_17486_KUHE17486
AB435299_Ansonia_sp_LSUHC_4991_LSUHC4991
AB435300_Ansonia_sp_LSUHC_4992_LSUHC4992
AB746457_Ansonia_leptopus_KUHE53839
AB435298_Ansonia_leptopus_KUHE17109
AB435297_Ansonia_leptopus_BORN22139
FJ882796_Ansonia_longidigita
AB435295_Ansonia_longidigita_KUHE12405
AB435293_Ansonia_longidigita_BORN12862
AB435294_Ansonia_longidigita_KUHEL04B133
AB331711_Ansonia_longidigita
FJ882795_Ansonia_leptopus
AB435296_Ansonia_torrentis_ZRC1_11918
GQ281550_Ansonia_longidigita_ZRC_1_12012
DQ283341_Ansonia_longidigita_FMNH_242550
GQ281549_Ansonia_longidigita_ZMHA_10001
GQ281551_Ansonia_longidigita_ZRC_1_12013

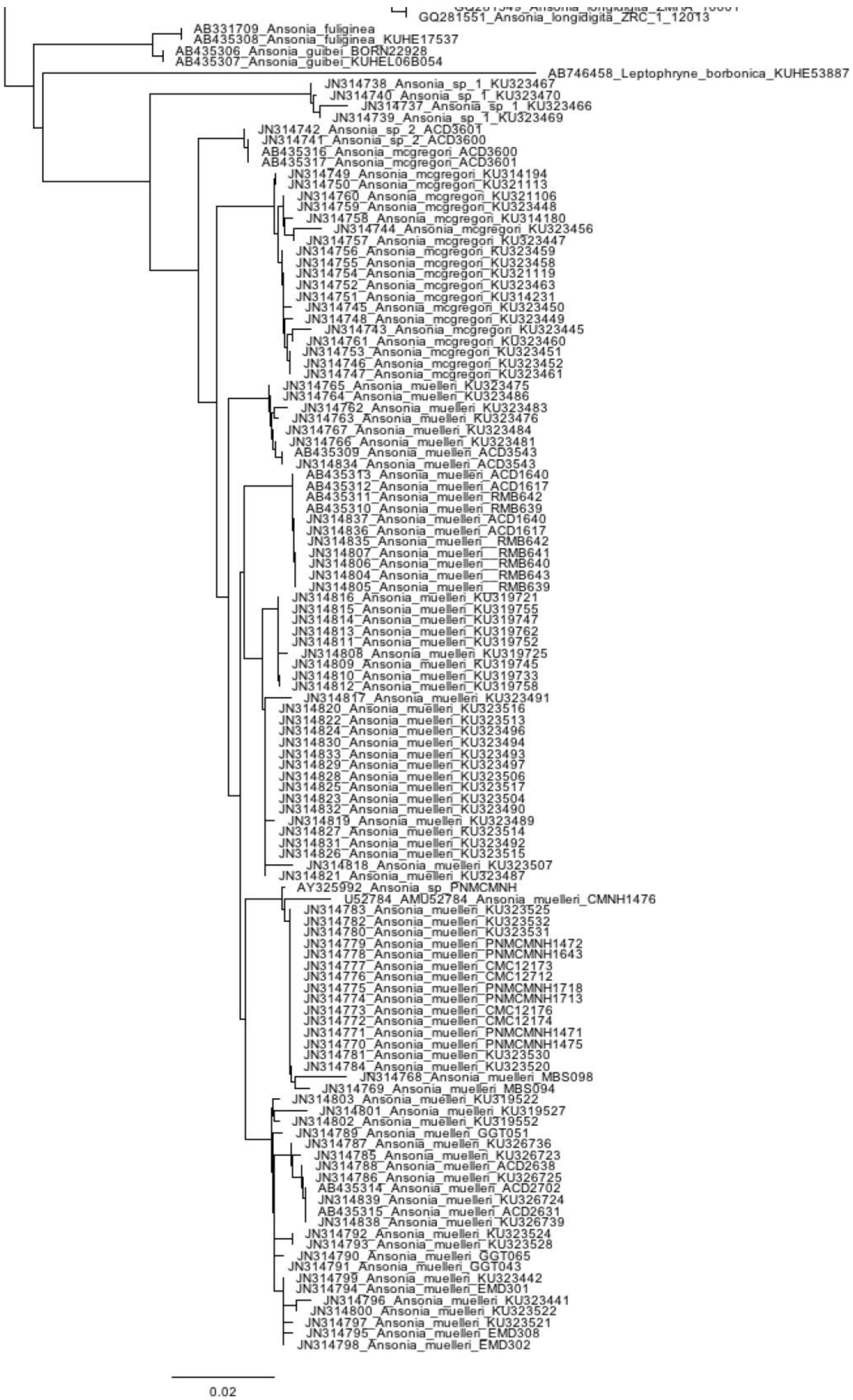

Fig. 2. Bufonidae 16S neighbor-joining tree.

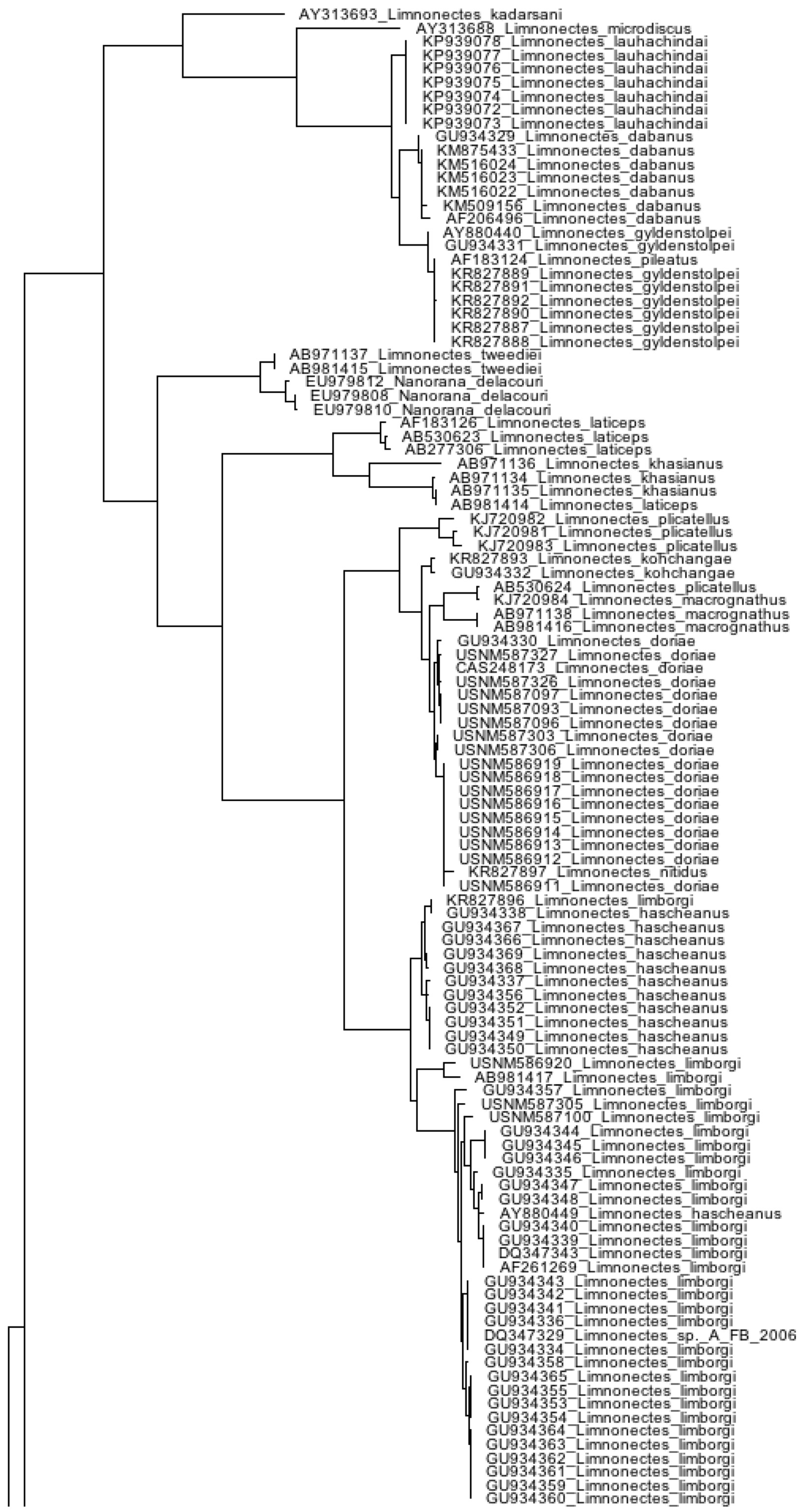
AY313693_Limnonectes_kadarsani
AY313688_Limnonectes_microdiscus
KP939078_Limnonectes_lauhachindai
KP939077_Limnonectes_lauhachindai
KP939076_Limnonectes_lauhachindai
KP939075_Limnonectes_lauhachindai
KP939074_Limnonectes_lauhachindai
KP939072_Limnonectes_lauhachindai
KP939073_Limnonectes_lauhachindai
GU934329_Limnonectes_dabanus
KM875433_Limnonectes_dabanus
KM516024_Limnonectes_dabanus
KM516023_Limnonectes_dabanus
KM516022_Limnonectes_dabanus
KM509156_Limnonectes_dabanus
AF206496_Limnonectes_dabanus
AY880440_Limnonectes_gyldenstolpei
GU934331_Limnonectes_gyldenstolpei
AF183124_Limnonectes_pileatus
KR827889_Limnonectes_gyldenstolpei
KR827891_Limnonectes_gyldenstolpei
KR827892_Limnonectes_gyldenstolpei
KR827890_Limnonectes_gyldenstolpei
KR827887_Limnonectes_gyldenstolpei
KR827888_Limnonectes_gyldenstolpei
AB971137_Limnonectes_tweediei
AB981415_Limnonectes_tweediei
EU979812_Nanorana_delacouri
EU979808_Nanorana_delacouri
EU979810_Nanorana_delacouri
AF183126_Limnonectes_laticeps
AB530623_Limnonectes_laticeps
AB277306_Limnonectes_laticeps
AB971136_Limnonectes_khasianus
AB971134_Limnonectes_khasianus
AB971135_Limnonectes_khasianus
AB981414_Limnonectes_laticeps
KJ720982_Limnonectes_plicatellus
KJ720981_Limnonectes_plicatellus
KJ720983_Limnonectes_plicatellus
KR827893_Limnonectes_kohchangae
GU934332_Limnonectes_kohchangae
AB530624_Limnonectes_plicatellus
KJ720984_Limnonectes_macrognathus
AB971138_Limnonectes_macrognathus
AB981416_Limnonectes_macrognathus
GU934330_Limnonectes_doriae
USNM587327_Limnonectes_doriae
CAS248173_Limnonectes_doriae
USNM587326_Limnonectes_doriae
USNM587097_Limnonectes_doriae
USNM587093_Limnonectes_doriae
USNM587096_Limnonectes_doriae
USNM587303_Limnonectes_doriae
USNM587306_Limnonectes_doriae
USNM586919_Limnonectes_doriae
USNM586918_Limnonectes_doriae
USNM586917_Limnonectes_doriae
USNM586916_Limnonectes_doriae
USNM586915_Limnonectes_doriae
USNM586914_Limnonectes_doriae
USNM586913_Limnonectes_doriae
USNM586912_Limnonectes_doriae
KR827897_Limnonectes_nitidus
USNM586911_Limnonectes_doriae
KR827896_Limnonectes_limborgi
GU934338_Limnonectes_hascheanus
GU934367_Limnonectes_hascheanus
GU934366_Limnonectes_hascheanus
GU934369_Limnonectes_hascheanus
GU934368_Limnonectes_hascheanus
GU934337_Limnonectes_hascheanus
GU934356_Limnonectes_hascheanus
GU934352_Limnonectes_hascheanus
GU934351_Limnonectes_hascheanus
GU934349_Limnonectes_hascheanus
GU934350_Limnonectes_hascheanus
USNM586920_Limnonectes_limborgi
AB981417_Limnonectes_limborgi
GU934357_Limnonectes_limborgi
USNM587305_Limnonectes_limborgi
USNM587100_Limnonectes_limborgi
GU934344_Limnonectes_limborgi
GU934345_Limnonectes_limborgi
GU934346_Limnonectes_limborgi
GU934335_Limnonectes_limborgi
GU934347_Limnonectes_limborgi
GU934348_Limnonectes_limborgi
AY880449_Limnonectes_hascheanus
GU934340_Limnonectes_limborgi
GU934339_Limnonectes_limborgi
DQ347343_Limnonectes_limborgi
AF261269_Limnonectes_limborgi
GU934343_Limnonectes_limborgi
GU934342_Limnonectes_limborgi
GU934341_Limnonectes_limborgi
GU934336_Limnonectes_limborgi
DQ347329_Limnonectes_sp._A_FB_2006
GU934334_Limnonectes_limborgi
GU934358_Limnonectes_limborgi
GU934365_Limnonectes_limborgi
GU934355_Limnonectes_limborgi
GU934353_Limnonectes_limborgi
GU934354_Limnonectes_limborgi
GU934364_Limnonectes_limborgi
GU934363_Limnonectes_limborgi
GU934362_Limnonectes_limborgi
GU934361_Limnonectes_limborgi
GU934359_Limnonectes_limborgi
GU934360_Limnonectes_limborgi

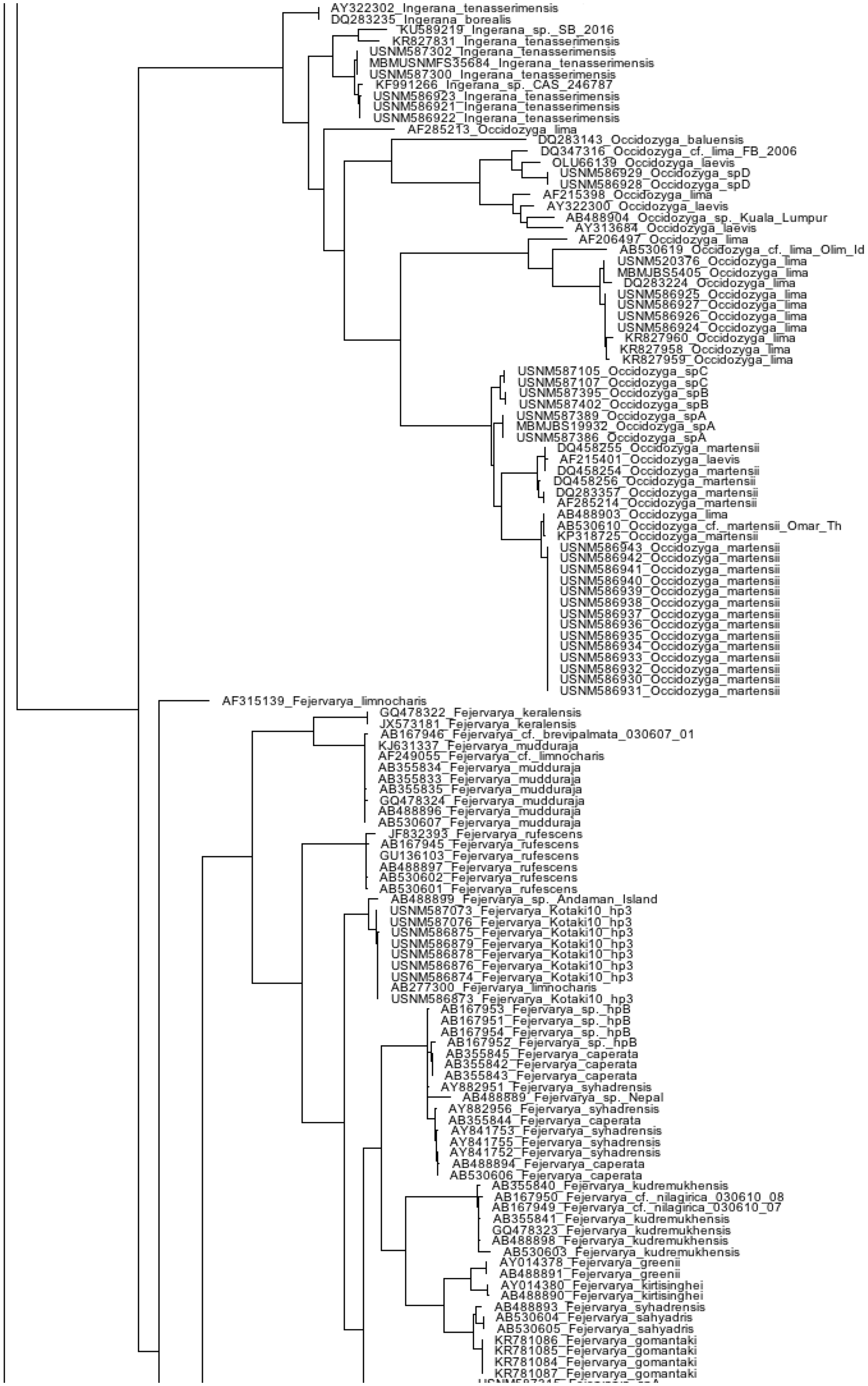
AY322302_Ingerana_tenasserimensis
DQ283235_Ingerana_borealis
KU589219_Ingerana_sp._SB_2016
KR827831_Ingerana_tenasserimensis
USNM587302_Ingerana_tenasserimensis
MBMUSNMFS35684_Ingerana_tenasserimensis
USNM587300_Ingerana_tenasserimensis
KF991266_Ingerana_sp._CAS_246787
USNM586923_Ingerana_tenasserimensis
USNM586921_Ingerana_tenasserimensis
USNM586922_Ingerana_tenasserimensis
AF285213_Occidozyga_lima
DQ283143_Occidozyga_baluensis
DQ347316_Occidozyga_cf._lima_FB_2006
OLU66139_Occidozyga_laevis
USNM586929_Occidozyga_spD
USNM586928_Occidozyga_spD
AF215398_Occidozyga_lima
AY322300_Occidozyga_laevis
AB488904_Occidozyga_sp._Kuala_Lumpur
AY313684_Occidozyga_laevis
AF206497_Occidozyga_lima
AB530619_Occidozyga_cf._lima_Olim_Id
USNM520376_Occidozyga_lima
MBMJBS5405_Occidozyga_lima
DQ283224_Occidozyga_lima
USNM586925_Occidozyga_lima
USNM586927_Occidozyga_lima
USNM586926_Occidozyga_lima
USNM586924_Occidozyga_lima
KR827960_Occidozyga_lima
KR827958_Occidozyga_lima
KR827959_Occidozyga_lima
USNM587105_Occidozyga_spC
USNM587107_Occidozyga_spC
USNM587395_Occidozyga_spB
USNM587402_Occidozyga_spB
USNM587389_Occidozyga_spA
MBMJBS19932_Occidozyga_spA
USNM587386_Occidozyga_spA
DQ458255_Occidozyga_martensii
AF215401_Occidozyga_laevis
DQ458254_Occidozyga_martensii
DQ458256_Occidozyga_martensii
DQ283357_Occidozyga_martensii
AF285214_Occidozyga_martensii
AB488903_Occidozyga_lima
AB530610_Occidozyga_cf._martensii_Omar_Th
KP318725_Occidozyga_martensii
USNM586943_Occidozyga_martensii
USNM586942_Occidozyga_martensii
USNM586941_Occidozyga_martensii
USNM586940_Occidozyga_martensii
USNM586939_Occidozyga_martensii
USNM586938_Occidozyga_martensii
USNM586937_Occidozyga_martensii
USNM586936_Occidozyga_martensii
USNM586935_Occidozyga_martensii
USNM586934_Occidozyga_martensii
USNM586933_Occidozyga_martensii
USNM586932_Occidozyga_martensii
USNM586930_Occidozyga_martensii
USNM586931_Occidozyga_martensii
AF315139_Fejervarya_limnocharis
GQ478322_Fejervarya_keralensis
JX573181_Fejervarya_keralensis
AB167946_Fejervarya_cf._brevipalmata_030607_01
KJ631337_Fejervarya_mudduraja
AF249055_Fejervarya_cf._limnocharis
AB355834_Fejervarya_mudduraja
AB355833_Fejervarya_mudduraja
AB355835_Fejervarya_mudduraja
GQ478324_Fejervarya_mudduraja
AB488896_Fejervarya_mudduraja
AB530607_Fejervarya_mudduraja
JF832393_Fejervarya_rufescens
AB167945_Fejervarya_rufescens
GU136103_Fejervarya_rufescens
AB488897_Fejervarya_rufescens
AB530602_Fejervarya_rufescens
AB530601_Fejervarya_rufescens
AB488899_Fejervarya_sp._Andaman_Island
USNM587073_Fejervarya_Kotaki10_hp3
USNM587076_Fejervarya_Kotaki10_hp3
USNM586875_Fejervarya_Kotaki10_hp3
USNM586879_Fejervarya_Kotaki10_hp3
USNM586878_Fejervarya_Kotaki10_hp3
USNM586876_Fejervarya_Kotaki10_hp3
USNM586874_Fejervarya_Kotaki10_hp3
AB277300_Fejervarya_limnocharis
USNM586873_Fejervarya_Kotaki10_hp3
AB167953_Fejervarya_sp._hpB
AB167951_Fejervarya_sp._hpB
AB167954_Fejervarya_sp._hpB
AB167952_Fejervarya_sp._hpB
AB355845_Fejervarya_caperata
AB355842_Fejervarya_caperata
AB355843_Fejervarya_caperata
AY882951_Fejervarya_syhadrensis
AB488889_Fejervarya_sp._Nepal
AY882956_Fejervarya_syhadrensis
AB355844_Fejervarya_caperata
AY841753_Fejervarya_syhadrensis
AY841755_Fejervarya_syhadrensis
AY841752_Fejervarya_syhadrensis
AB488894_Fejervarya_caperata
AB530606_Fejervarya_caperata
AB355840_Fejervarya_kudremukhensis
AB167950_Fejervarya_cf._nilagirica_030610_08
AB167949_Fejervarya_cf._nilagirica_030610_07
AB355841_Fejervarya_kudremukhensis
GQ478323_Fejervarya_kudremukhensis
AB488898_Fejervarya_kudremukhensis
AB530603_Fejervarya_kudremukhensis
AY014378_Fejervarya_greenii
AB488891_Fejervarya_greenii
AY014380_Fejervarya_kirtisinghei
AB488890_Fejervarya_kirtisinghei
AB488893_Fejervarya_syhadrensis
AB530604_Fejervarya_sahyadris
AB530605_Fejervarya_sahyadris
KR781086_Fejervarya_gomantaki
KR781085_Fejervarya_gomantaki
KR781084_Fejervarya_gomantaki
KR781087_Fejervarya_gomantaki

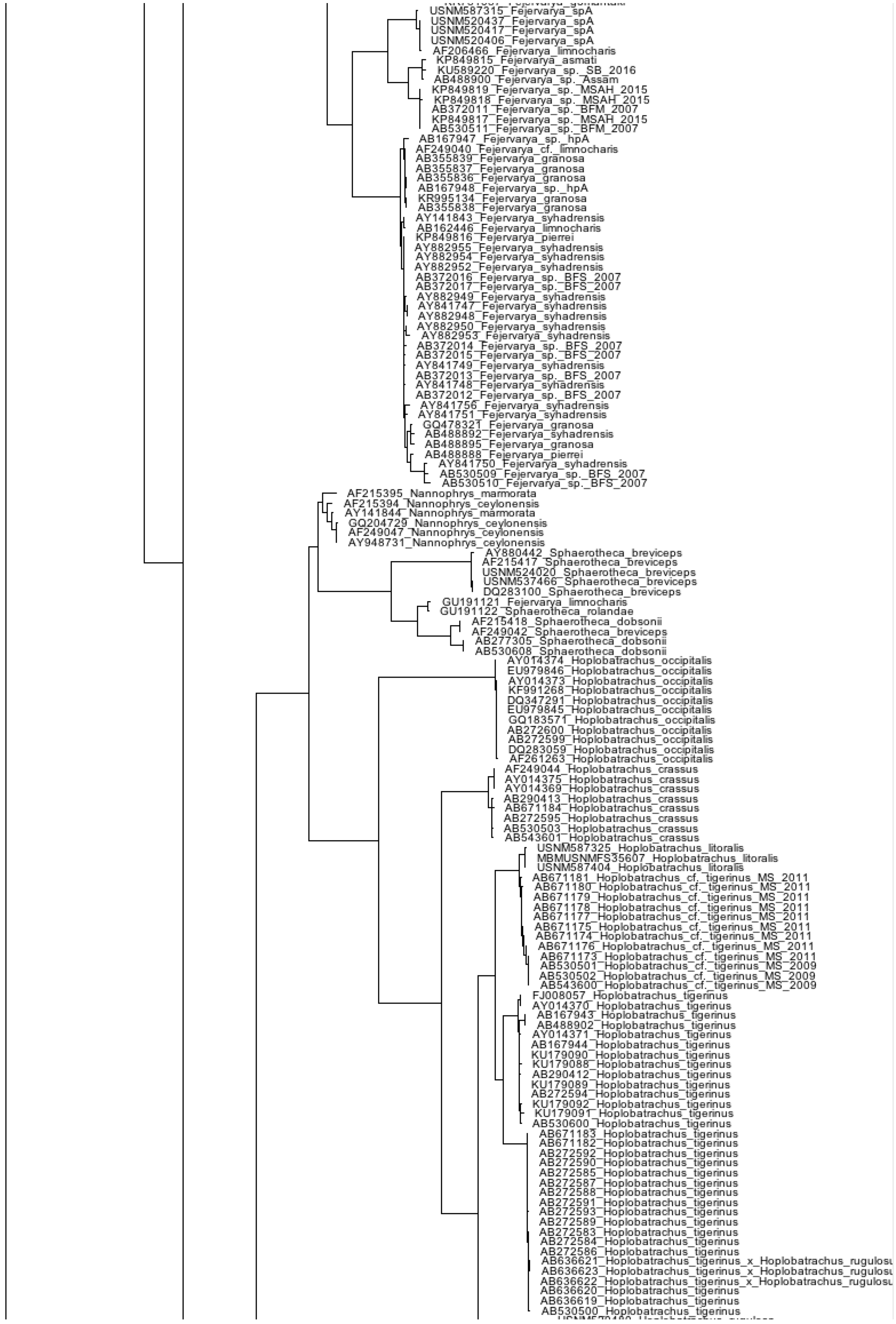
USNM587315_Fejervarya_spA
USNM520437_Fejervarya_spA
USNM520417_Fejervarya_spA
USNM520406_Fejervarya_spA
AF206466_Fejervarya_limnocharis
KP849815_Fejervarya_asmati
KU589220_Fejervarya_sp._SB_2016
AB488900_Fejervarya_sp._Assam
KP849819_Fejervarya_sp._MSAH_2015
KP849818_Fejervarya_sp._MSAH_2015
AB372011_Fejervarya_sp._BFM_2007
KP849817_Fejervarya_sp._MSAH_2015
AB530511_Fejervarya_sp._BFM_2007
AB167947_Fejervarya_sp._hpA
AF249040_Fejervarya_cf._limnocharis
AB355839_Fejervarya_granosa
AB355837_Fejervarya_granosa
AB355836_Fejervarya_granosa
AB167948_Fejervarya_sp._hpA
KR995134_Fejervarya_granosa
AB355838_Fejervarya_granosa
AY141843_Fejervarya_syhadrensis
AB162446_Fejervarya_limnocharis
KP849816_Fejervarya_pierrei
AY882955_Fejervarya_syhadrensis
AY882954_Fejervarya_syhadrensis
AY882952_Fejervarya_syhadrensis
AB372016_Fejervarya_sp._BFS_2007
AB372017_Fejervarya_sp._BFS_2007
AY882949_Fejervarya_syhadrensis
AY841747_Fejervarya_syhadrensis
AY882948_Fejervarya_syhadrensis
AY882950_Fejervarya_syhadrensis
AY882953_Fejervarya_syhadrensis
AB372014_Fejervarya_sp._BFS_2007
AB372015_Fejervarya_sp._BFS_2007
AY841749_Fejervarya_syhadrensis
AB372013_Fejervarya_sp._BFS_2007
AY841748_Fejervarya_syhadrensis
AB372012_Fejervarya_sp._BFS_2007
AY841756_Fejervarya_syhadrensis
AY841751_Fejervarya_syhadrensis
GQ478321_Fejervarya_granosa
AB488892_Fejervarya_syhadrensis
AB488895_Fejervarya_granosa
AB488888_Fejervarya_pierrei
AY841750_Fejervarya_syhadrensis
AB530509_Fejervarya_sp._BFS_2007
AB530510_Fejervarya_sp._BFS_2007
AF215395_Nannophrys_marmorata
AF215394_Nannophrys_ceylonensis
AY141844_Nannophrys_marmorata
GQ204729_Nannophrys_ceylonensis
AF249047_Nannophrys_ceylonensis
AY948731_Nannophrys_ceylonensis
AY880442_Sphaerotheca_breviceps
AF215417_Sphaerotheca_breviceps
USNM524020_Sphaerotheca_breviceps
USNM537466_Sphaerotheca_breviceps
DQ283100_Sphaerotheca_breviceps
GU191121_Fejervarya_limnocharis
GU191122_Sphaerotheca_rolandae
AF215418_Sphaerotheca_dobsonii
AF249042_Sphaerotheca_breviceps
AB277305_Sphaerotheca_dobsonii
AB530608_Sphaerotheca_dobsonii
AY014374_Hoplobatrachus_occipitalis
EU979846_Hoplobatrachus_occipitalis
AY014373_Hoplobatrachus_occipitalis
KF991268_Hoplobatrachus_occipitalis
DQ347291_Hoplobatrachus_occipitalis
EU979845_Hoplobatrachus_occipitalis
GQ183571_Hoplobatrachus_occipitalis
AB272600_Hoplobatrachus_occipitalis
AB272599_Hoplobatrachus_occipitalis
DQ283059_Hoplobatrachus_occipitalis
AF261263_Hoplobatrachus_occipitalis
AF249044_Hoplobatrachus_crassus
AY014375_Hoplobatrachus_crassus
AY014369_Hoplobatrachus_crassus
AB290413_Hoplobatrachus_crassus
AB671184_Hoplobatrachus_crassus
AB272595_Hoplobatrachus_crassus
AB530503_Hoplobatrachus_crassus
AB543601_Hoplobatrachus_crassus
USNM587325_Hoplobatrachus_litoralis
MBMUSNMFS35607_Hoplobatrachus_litoralis
USNM587404_Hoplobatrachus_litoralis
AB671181_Hoplobatrachus_cf._tigerinus_MS_2011
AB671180_Hoplobatrachus_cf._tigerinus_MS_2011
AB671179_Hoplobatrachus_cf._tigerinus_MS_2011
AB671178_Hoplobatrachus_cf._tigerinus_MS_2011
AB671177_Hoplobatrachus_cf._tigerinus_MS_2011
AB671175_Hoplobatrachus_cf._tigerinus_MS_2011
AB671174_Hoplobatrachus_cf._tigerinus_MS_2011
AB671176_Hoplobatrachus_cf._tigerinus_MS_2011
AB671173_Hoplobatrachus_cf._tigerinus_MS_2011
AB530501_Hoplobatrachus_cf._tigerinus_MS_2009
AB530502_Hoplobatrachus_cf._tigerinus_MS_2009
AB543600_Hoplobatrachus_cf._tigerinus_MS_2009
FJ008057_Hoplobatrachus_tigerinus
AY014370_Hoplobatrachus_tigerinus
AB167943_Hoplobatrachus_tigerinus
AB488902_Hoplobatrachus_tigerinus
AY014371_Hoplobatrachus_tigerinus
AB167944_Hoplobatrachus_tigerinus
KU179090_Hoplobatrachus_tigerinus
KU179088_Hoplobatrachus_tigerinus
AB290412_Hoplobatrachus_tigerinus
KU179089_Hoplobatrachus_tigerinus
AB272594_Hoplobatrachus_tigerinus
KU179092_Hoplobatrachus_tigerinus
KU179091_Hoplobatrachus_tigerinus
AB530600_Hoplobatrachus_tigerinus
AB671183_Hoplobatrachus_tigerinus
AB671182_Hoplobatrachus_tigerinus
AB272592_Hoplobatrachus_tigerinus
AB272590_Hoplobatrachus_tigerinus
AB272585_Hoplobatrachus_tigerinus
AB272587_Hoplobatrachus_tigerinus
AB272588_Hoplobatrachus_tigerinus
AB272591_Hoplobatrachus_tigerinus
AB272593_Hoplobatrachus_tigerinus
AB272589_Hoplobatrachus_tigerinus
AB272583_Hoplobatrachus_tigerinus
AB272584_Hoplobatrachus_tigerinus
AB272586_Hoplobatrachus_tigerinus
AB636621_Hoplobatrachus_tigerinus_x_Hoplobatrachus_rugulosu
AB636623_Hoplobatrachus_tigerinus_x_Hoplobatrachus_rugulosu
AB636622_Hoplobatrachus_tigerinus_x_Hoplobatrachus_rugulosu
AB636620_Hoplobatrachus_tigerinus
AB636619_Hoplobatrachus_tigerinus
AB530500_Hoplobatrachus_tigerinus

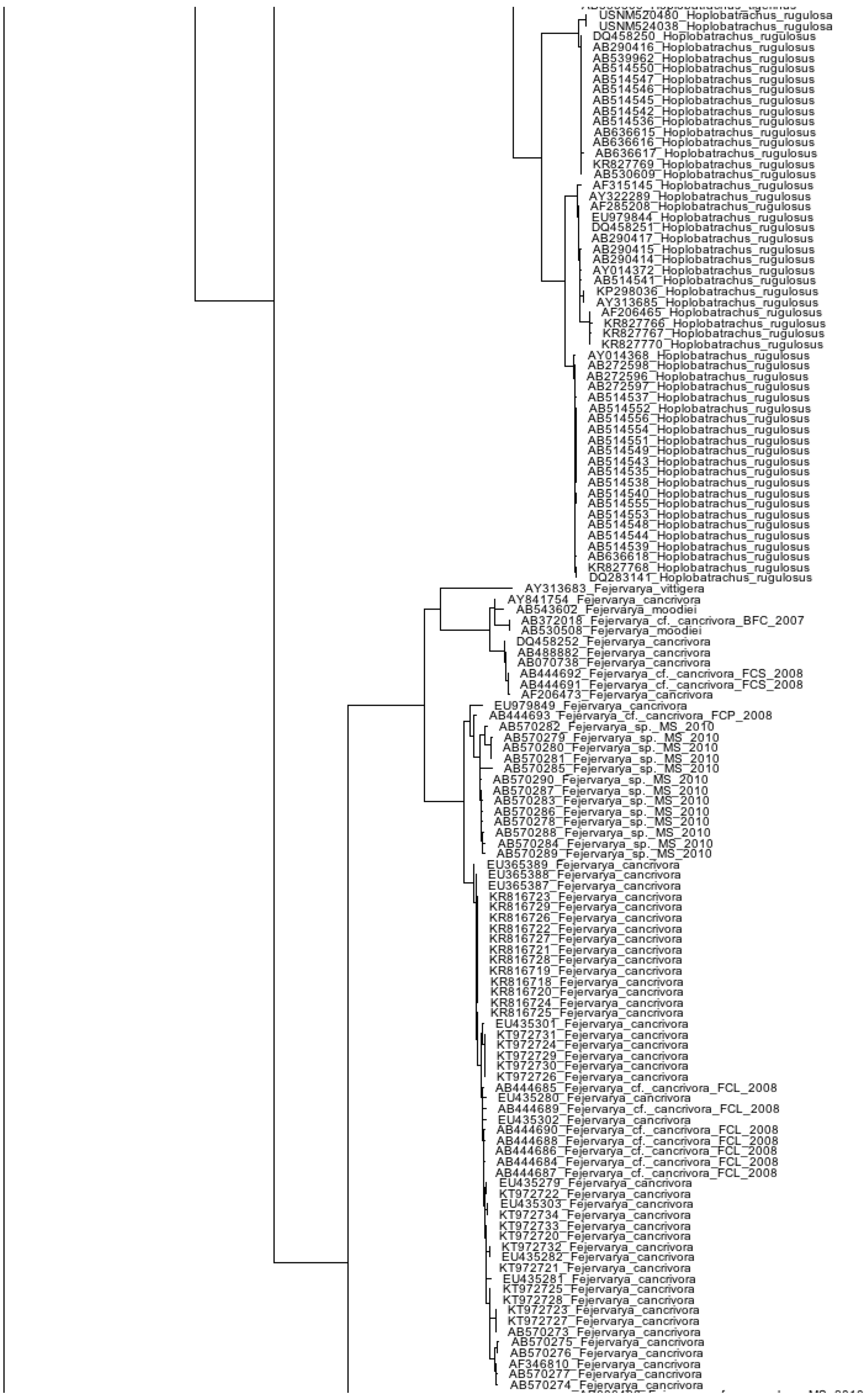
USNM520480_Hoplobatrachus_rugulosa
USNM524038_Hoplobatrachus_rugulosa
DQ458250_Hoplobatrachus_rugulosus
AB290416_Hoplobatrachus_rugulosus
AB539962_Hoplobatrachus_rugulosus
AB514550_Hoplobatrachus_rugulosus
AB514547_Hoplobatrachus_rugulosus
AB514546_Hoplobatrachus_rugulosus
AB514545_Hoplobatrachus_rugulosus
AB514542_Hoplobatrachus_rugulosus
AB514536_Hoplobatrachus_rugulosus
AB636615_Hoplobatrachus_rugulosus
AB636616_Hoplobatrachus_rugulosus
AB636617_Hoplobatrachus_rugulosus
KR827769_Hoplobatrachus_rugulosus
AB530609_Hoplobatrachus_rugulosus
AF315145_Hoplobatrachus_rugulosus
AY322289_Hoplobatrachus_rugulosus
AF285208_Hoplobatrachus_rugulosus
EU979844_Hoplobatrachus_rugulosus
DQ458251_Hoplobatrachus_rugulosus
AB290417_Hoplobatrachus_rugulosus
AB290415_Hoplobatrachus_rugulosus
AB290414_Hoplobatrachus_rugulosus
AY014372_Hoplobatrachus_rugulosus
AB514541_Hoplobatrachus_rugulosus
KP298036_Hoplobatrachus_rugulosus
AY313685_Hoplobatrachus_rugulosus
AF206465_Hoplobatrachus_rugulosus
KR827766_Hoplobatrachus_rugulosus
KR827767_Hoplobatrachus_rugulosus
KR827770_Hoplobatrachus_rugulosus
AY014368_Hoplobatrachus_rugulosus
AB272598_Hoplobatrachus_rugulosus
AB272596_Hoplobatrachus_rugulosus
AB272597_Hoplobatrachus_rugulosus
AB514537_Hoplobatrachus_rugulosus
AB514552_Hoplobatrachus_rugulosus
AB514556_Hoplobatrachus_rugulosus
AB514554_Hoplobatrachus_rugulosus
AB514551_Hoplobatrachus_rugulosus
AB514549_Hoplobatrachus_rugulosus
AB514543_Hoplobatrachus_rugulosus
AB514535_Hoplobatrachus_rugulosus
AB514538_Hoplobatrachus_rugulosus
AB514540_Hoplobatrachus_rugulosus
AB514555_Hoplobatrachus_rugulosus
AB514553_Hoplobatrachus_rugulosus
AB514548_Hoplobatrachus_rugulosus
AB514544_Hoplobatrachus_rugulosus
AB514539_Hoplobatrachus_rugulosus
AB636618_Hoplobatrachus_rugulosus
KR827768_Hoplobatrachus_rugulosus
DQ283141_Hoplobatrachus_rugulosus
AY313683_Fejervarya_vittigera
AY841754_Fejervarya_cancrivora
AB543602_Fejervarya_moodiei
AB372018_Fejervarya_cf._cancrivora_BFC_2007
AB530508_Fejervarya_moodiei
DQ458252_Fejervarya_cancrivora
AB488882_Fejervarya_cancrivora
AB070738_Fejervarya_cancrivora
AB444692_Fejervarya_cf._cancrivora_FCS_2008
AB444691_Fejervarya_cf._cancrivora_FCS_2008
AF206473_Fejervarya_cancrivora
EU979849_Fejervarya_cancrivora
AB444693_Fejervarya_cf._cancrivora_FCP_2008
AB570282_Fejervarya_sp._MS_2010
AB570279_Fejervarya_sp._MS_2010
AB570280_Fejervarya_sp._MS_2010
AB570281_Fejervarya_sp._MS_2010
AB570285_Fejervarya_sp._MS_2010
AB570290_Fejervarya_sp._MS_2010
AB570287_Fejervarya_sp._MS_2010
AB570283_Fejervarya_sp._MS_2010
AB570286_Fejervarya_sp._MS_2010
AB570278_Fejervarya_sp._MS_2010
AB570288_Fejervarya_sp._MS_2010
AB570284_Fejervarya_sp._MS_2010
AB570289_Fejervarya_sp._MS_2010
EU365389_Fejervarya_cancrivora
EU365388_Fejervarya_cancrivora
EU365387_Fejervarya_cancrivora
KR816723_Fejervarya_cancrivora
KR816729_Fejervarya_cancrivora
KR816726_Fejervarya_cancrivora
KR816722_Fejervarya_cancrivora
KR816727_Fejervarya_cancrivora
KR816721_Fejervarya_cancrivora
KR816728_Fejervarya_cancrivora
KR816719_Fejervarya_cancrivora
KR816718_Fejervarya_cancrivora
KR816720_Fejervarya_cancrivora
KR816724_Fejervarya_cancrivora
KR816725_Fejervarya_cancrivora
EU435301_Fejervarya_cancrivora
KT972731_Fejervarya_cancrivora
KT972724_Fejervarya_cancrivora
KT972729_Fejervarya_cancrivora
KT972730_Fejervarya_cancrivora
KT972726_Fejervarya_cancrivora
AB444685_Fejervarya_cf._cancrivora_FCL_2008
EU435280_Fejervarya_cancrivora
AB444689_Fejervarya_cf._cancrivora_FCL_2008
EU435302_Fejervarya_cancrivora
AB444690_Fejervarya_cf._cancrivora_FCL_2008
AB444688_Fejervarya_cf._cancrivora_FCL_2008
AB444686_Fejervarya_cf._cancrivora_FCL_2008
AB444684_Fejervarya_cf._cancrivora_FCL_2008
AB444687_Fejervarya_cf._cancrivora_FCL_2008
EU435279_Fejervarya_cancrivora
KT972722_Fejervarya_cancrivora
EU435303_Fejervarya_cancrivora
KT972734_Fejervarya_cancrivora
KT972733_Fejervarya_cancrivora
KT972720_Fejervarya_cancrivora
KT972732_Fejervarya_cancrivora
EU435282_Fejervarya_cancrivora
KT972721_Fejervarya_cancrivora
EU435281_Fejervarya_cancrivora
KT972725_Fejervarya_cancrivora
KT972728_Fejervarya_cancrivora
KT972723_Fejervarya_cancrivora
KT972727_Fejervarya_cancrivora
AB570273_Fejervarya_cancrivora
AB570275_Fejervarya_cancrivora
AB570276_Fejervarya_cancrivora
AF346810_Fejervarya_cancrivora
AB570277_Fejervarya_cancrivora
AB570274_Fejervarya_cancrivora

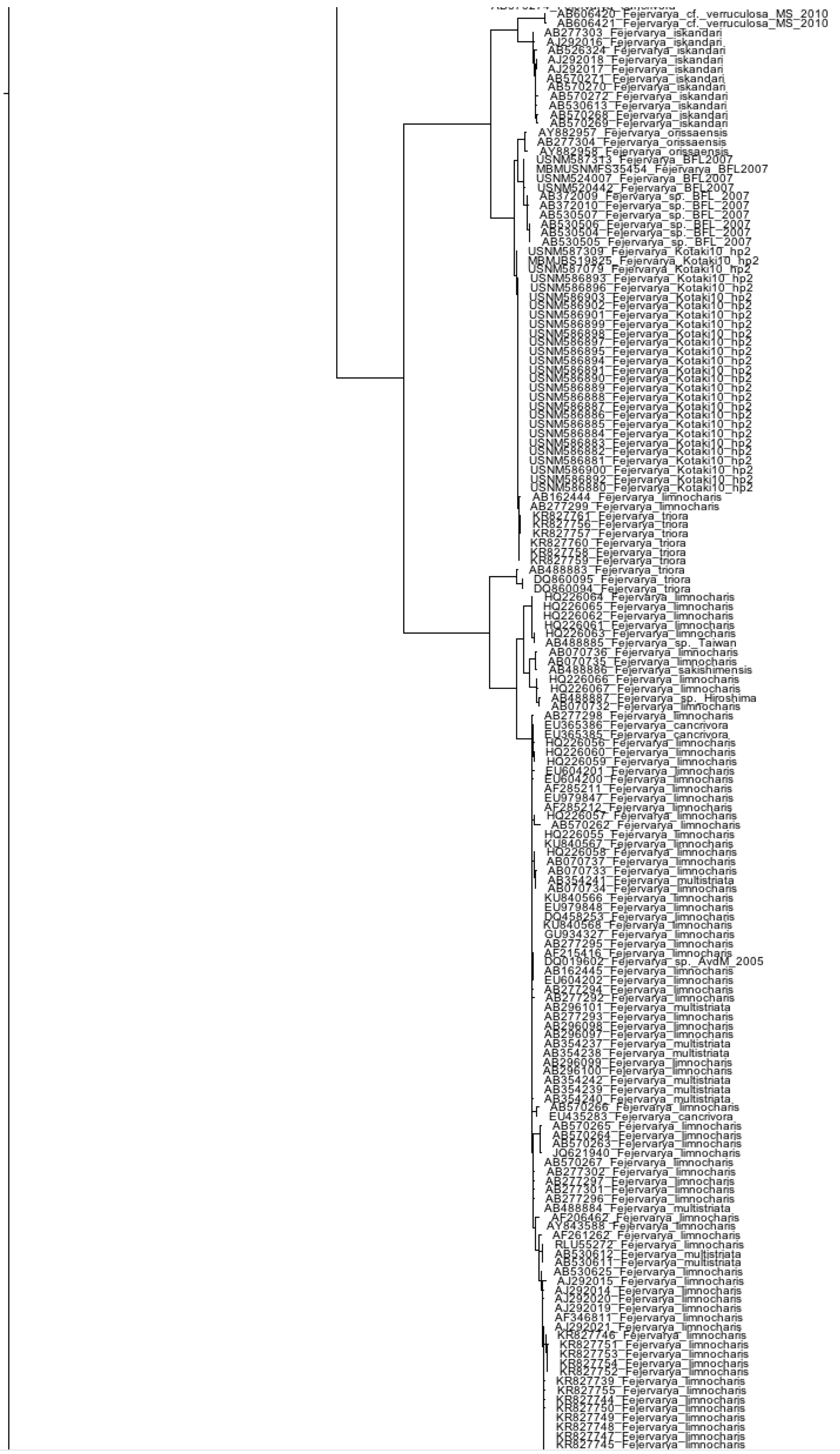
AB606420_Fejervarya_cf._verruculosa_MS_2010
AB606421_Fejervarya_cf._verruculosa_MS_2010
AB277303_Fejervarya_iskandari
AJ292016_Fejervarya_iskandari
AB526324_Fejervarya_iskandari
AJ292018_Fejervarya_iskandari
AJ292017_Fejervarya_iskandari
AB570271_Fejervarya_iskandari
AB570270_Fejervarya_iskandari
AB570272_Fejervarya_iskandari
AB530613_Fejervarya_iskandari
AB570268_Fejervarya_iskandari
AB570269_Fejervarya_iskandari
AY882957_Fejervarya_orissaensis
AB277304_Fejervarya_orissaensis
AY882958_Fejervarya_orissaensis
USNM587313_Fejervarya_BFL2007
MBMUSNMFS35454_Fejervarya_BFL2007
USNM524007_Fejervarya_BFL2007
USNM520442_Fejervarya_BFL2007
AB372009_Fejervarya_sp._BFL_2007
AB372010_Fejervarya_sp._BFL_2007
AB530507_Fejervarya_sp._BFL_2007
AB530506_Fejervarya_sp._BFL_2007
AB530504_Fejervarya_sp._BFL_2007
AB530505_Fejervarya_sp._BFL_2007
USNM587309_Fejervarya_Kotaki10_hp2
MBMJBS19825_Fejervarya_Kotaki10_hp2
USNM587079_Fejervarya_Kotaki10_hp2
USNM586893_Fejervarya_Kotaki10_hp2
USNM586896_Fejervarya_Kotaki10_hp2
USNM586903_Fejervarya_Kotaki10_hp2
USNM586902_Fejervarya_Kotaki10_hp2
USNM586901_Fejervarya_Kotaki10_hp2
USNM586899_Fejervarya_Kotaki10_hp2
USNM586898_Fejervarya_Kotaki10_hp2
USNM586897_Fejervarya_Kotaki10_hp2
USNM586895_Fejervarya_Kotaki10_hp2
USNM586894_Fejervarya_Kotaki10_hp2
USNM586891_Fejervarya_Kotaki10_hp2
USNM586890_Fejervarya_Kotaki10_hp2
USNM586889_Fejervarya_Kotaki10_hp2
USNM586888_Fejervarya_Kotaki10_hp2
USNM586887_Fejervarya_Kotaki10_hp2
USNM586886_Fejervarya_Kotaki10_hp2
USNM586885_Fejervarya_Kotaki10_hp2
USNM586884_Fejervarya_Kotaki10_hp2
USNM586883_Fejervarya_Kotaki10_hp2
USNM586882_Fejervarya_Kotaki10_hp2
USNM586881_Fejervarya_Kotaki10_hp2
USNM586900_Fejervarya_Kotaki10_hp2
USNM586892_Fejervarya_Kotaki10_hp2
USNM586880_Fejervarya_Kotaki10_hp2
AB162444_Fejervarya_limnocharis
AB277299_Fejervarya_limnocharis
KR827761_Fejervarya_triora
KR827756_Fejervarya_triora
KR827757_Fejervarya_triora
KR827760_Fejervarya_triora
KR827758_Fejervarya_triora
KR827759_Fejervarya_triora
AB488883_Fejervarya_triora
DQ860095_Fejervarya_triora
DQ860094_Fejervarya_triora
HQ226064_Fejervarya_limnocharis
HQ226065_Fejervarya_limnocharis
HQ226062_Fejervarya_limnocharis
HQ226061_Fejervarya_limnocharis
HQ226063_Fejervarya_limnocharis
AB488885_Fejervarya_sp._Taiwan
AB070736_Fejervarya_limnocharis
AB070735_Fejervarya_limnocharis
AB488886_Fejervarya_sakishimensis
HQ226066_Fejervarya_limnocharis
HQ226067_Fejervarya_limnocharis
AB488887_Fejervarya_sp._Hiroshima
AB070732_Fejervarya_limnocharis
AB277298_Fejervarya_limnocharis
EU365386_Fejervarya_cancrivora
EU365385_Fejervarya_cancrivora
HQ226056_Fejervarya_limnocharis
HQ226060_Fejervarya_limnocharis
HQ226059_Fejervarya_limnocharis
EU604201_Fejervarya_limnocharis
EU604200_Fejervarya_limnocharis
AF285211_Fejervarya_limnocharis
EU979847_Fejervarya_limnocharis
AF285212_Fejervarya_limnocharis
HQ226057_Fejervarya_limnocharis
AB570262_Fejervarya_limnocharis
HQ226055_Fejervarya_limnocharis
KU840567_Fejervarya_limnocharis
HQ226058_Fejervarya_limnocharis
AB070737_Fejervarya_limnocharis
AB070733_Fejervarya_limnocharis
AB354241_Fejervarya_multistriata
AB070734_Fejervarya_limnocharis
KU840566_Fejervarya_limnocharis
EU979848_Fejervarya_limnocharis
DQ458253_Fejervarya_limnocharis
KU840568_Fejervarya_limnocharis
GU934327_Fejervarya_limnocharis
AB277295_Fejervarya_limnocharis
AF215416_Fejervarya_limnocharis
DQ019602_Fejervarya_sp._AvdM_2005
AB162445_Fejervarya_limnocharis
EU604202_Fejervarya_limnocharis
AB277294_Fejervarya_limnocharis
AB277292_Fejervarya_limnocharis
AB296101_Fejervarya_multistriata
AB277293_Fejervarya_limnocharis
AB296098_Fejervarya_limnocharis
AB296097_Fejervarya_limnocharis
AB354237_Fejervarya_multistriata
AB354238_Fejervarya_multistriata
AB296099_Fejervarya_limnocharis
AB296100_Fejervarya_limnocharis
AB354242_Fejervarya_multistriata
AB354239_Fejervarya_multistriata
AB354240_Fejervarya_multistriata
AB570266_Fejervarya_limnocharis
EU435283_Fejervarya_cancrivora
AB570265_Fejervarya_limnocharis
AB570264_Fejervarya_limnocharis
AB570263_Fejervarya_limnocharis
JQ621940_Fejervarya_limnocharis
AB570267_Fejervarya_limnocharis
AB277302_Fejervarya_limnocharis
AB277297_Fejervarya_limnocharis
AB277301_Fejervarya_limnocharis
AB277296_Fejervarya_limnocharis
AB488884_Fejervarya_multistriata
AF206462_Fejervarya_limnocharis
AY843588_Fejervarya_limnocharis
AF261262_Fejervarya_limnocharis
RLU55272_Fejervarya_limnocharis
AB530612_Fejervarya_multistriata
AB530611_Fejervarya_multistriata
AB530625_Fejervarya_limnocharis
AJ292015_Fejervarya_limnocharis
AJ292014_Fejervarya_limnocharis
AJ292020_Fejervarya_limnocharis
AJ292019_Fejervarya_limnocharis
AF346811_Fejervarya_limnocharis
AJ292021_Fejervarya_limnocharis
KR827746_Fejervarya_limnocharis
KR827751_Fejervarya_limnocharis
KR827753_Fejervarya_limnocharis
KR827754_Fejervarya_limnocharis
KR827752_Fejervarya_limnocharis
KR827739_Fejervarya_limnocharis
KR827755_Fejervarya_limnocharis
KR827744_Fejervarya_limnocharis
KR827750_Fejervarya_limnocharis
KR827749_Fejervarya_limnocharis
KR827748_Fejervarya_limnocharis
KR827747_Fejervarya_limnocharis
KR827745_Fejervarya_limnocharis

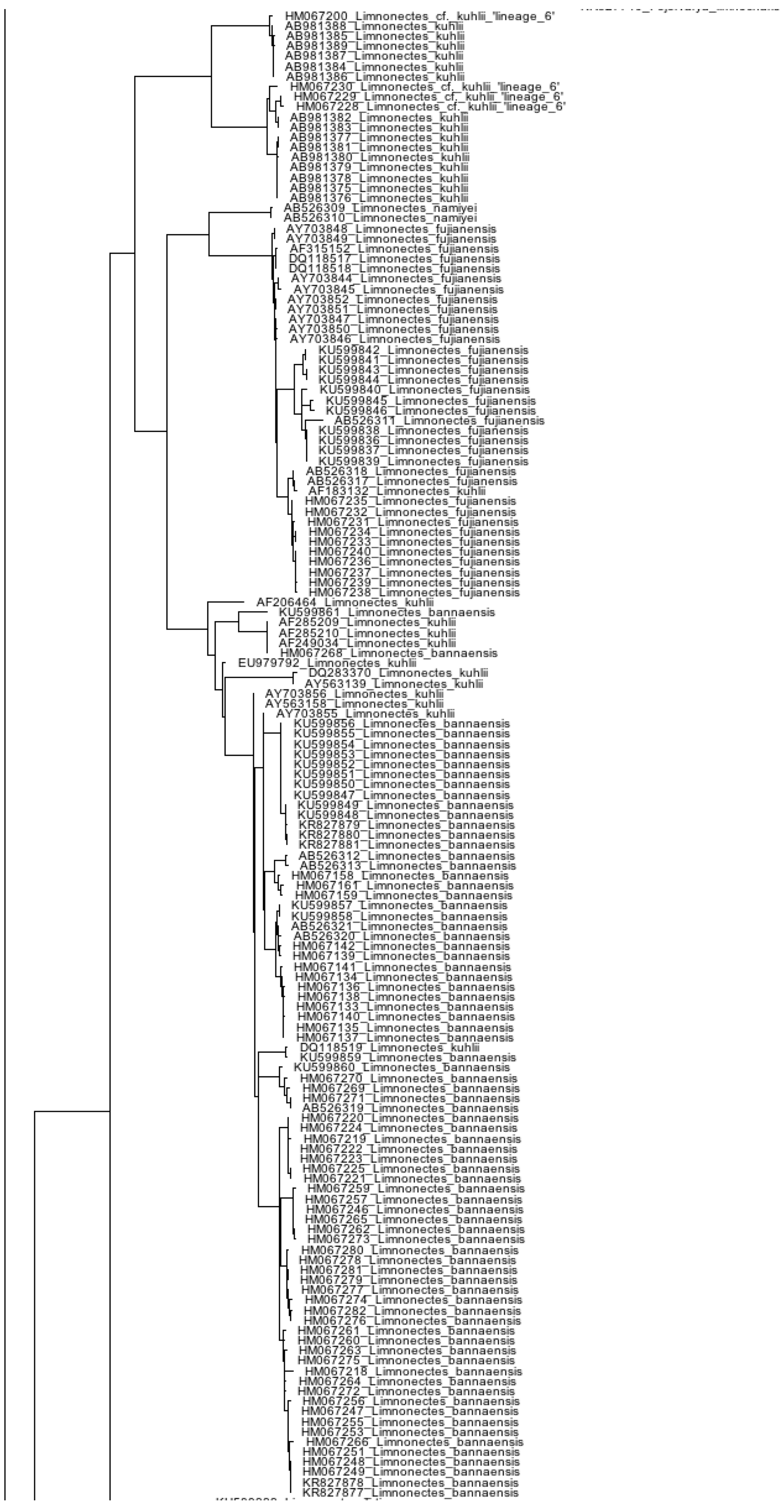

HM067200_Limnonectes_cf._kuhlii_'lineage_6'
AB981388_Limnonectes_kuhlii
AB981385_Limnonectes_kuhlii
AB981389_Limnonectes_kuhlii
AB981387_Limnonectes_kuhlii
AB981384_Limnonectes_kuhlii
AB981386_Limnonectes_kuhlii
HM067230_Limnonectes_cf._kuhlii_'lineage_6'
HM067229_Limnonectes_cf._kuhlii_'lineage_6'
HM067228_Limnonectes_cf._kuhlii_'lineage_6'
AB981382_Limnonectes_kuhlii
AB981383_Limnonectes_kuhlii
AB981377_Limnonectes_kuhlii
AB981381_Limnonectes_kuhlii
AB981380_Limnonectes_kuhlii
AB981379_Limnonectes_kuhlii
AB981378_Limnonectes_kuhlii
AB981375_Limnonectes_kuhlii
AB981376_Limnonectes_kuhlii
AB526309_Limnonectes_namiyei
AB526310_Limnonectes_namiyei
AY703848_Limnonectes_fujianensis
AY703849_Limnonectes_fujianensis
AF315152_Limnonectes_fujianensis
DQ118517_Limnonectes_fujianensis
DQ118518_Limnonectes_fujianensis
AY703844_Limnonectes_fujianensis
AY703845_Limnonectes_fujianensis
AY703852_Limnonectes_fujianensis
AY703851_Limnonectes_fujianensis
AY703847_Limnonectes_fujianensis
AY703850_Limnonectes_fujianensis
AY703846_Limnonectes_fujianensis
KU599842_Limnonectes_fujianensis
KU599841_Limnonectes_fujianensis
KU599843_Limnonectes_fujianensis
KU599844_Limnonectes_fujianensis
KU599840_Limnonectes_fujianensis
KU599845_Limnonectes_fujianensis
KU599846_Limnonectes_fujianensis
AB526311_Limnonectes_fujianensis
KU599838_Limnonectes_fujianensis
KU599836_Limnonectes_fujianensis
KU599837_Limnonectes_fujianensis
KU599839_Limnonectes_fujianensis
AB526318_Limnonectes_fujianensis
AB526317_Limnonectes_fujianensis
AF183132_Limnonectes_kuhlii
HM067235_Limnonectes_fujianensis
HM067232_Limnonectes_fujianensis
HM067231_Limnonectes_fujianensis
HM067234_Limnonectes_fujianensis
HM067233_Limnonectes_fujianensis
HM067240_Limnonectes_fujianensis
HM067236_Limnonectes_fujianensis
HM067237_Limnonectes_fujianensis
HM067239_Limnonectes_fujianensis
HM067238_Limnonectes_fujianensis
AF206464_Limnonectes_kuhlii
KU599861_Limnonectes_bannaensis
AF285209_Limnonectes_kuhlii
AF285210_Limnonectes_kuhlii
AF249034_Limnonectes_kuhlii
HM067268_Limnonectes_bannaensis
EU979792_Limnonectes_kuhlii
DQ283370_Limnonectes_kuhlii
AY563139_Limnonectes_kuhlii
AY703856_Limnonectes_kuhlii
AY563158_Limnonectes_kuhlii
AY703855_Limnonectes_kuhlii
KU599856_Limnonectes_bannaensis
KU599855_Limnonectes_bannaensis
KU599854_Limnonectes_bannaensis
KU599853_Limnonectes_bannaensis
KU599852_Limnonectes_bannaensis
KU599851_Limnonectes_bannaensis
KU599850_Limnonectes_bannaensis
KU599847_Limnonectes_bannaensis
KU599849_Limnonectes_bannaensis
KU599848_Limnonectes_bannaensis
KR827879_Limnonectes_bannaensis
KR827880_Limnonectes_bannaensis
KR827881_Limnonectes_bannaensis
AB526312_Limnonectes_bannaensis
AB526313_Limnonectes_bannaensis
HM067158_Limnonectes_bannaensis
HM067161_Limnonectes_bannaensis
HM067159_Limnonectes_bannaensis
KU599857_Limnonectes_bannaensis
KU599858_Limnonectes_bannaensis
AB526321_Limnonectes_bannaensis
AB526320_Limnonectes_bannaensis
HM067142_Limnonectes_bannaensis
HM067139_Limnonectes_bannaensis
HM067141_Limnonectes_bannaensis
HM067134_Limnonectes_bannaensis
HM067136_Limnonectes_bannaensis
HM067138_Limnonectes_bannaensis
HM067133_Limnonectes_bannaensis
HM067140_Limnonectes_bannaensis
HM067135_Limnonectes_bannaensis
HM067137_Limnonectes_bannaensis
DQ118519_Limnonectes_kuhlii
KU599859_Limnonectes_bannaensis
KU599860_Limnonectes_bannaensis
HM067270_Limnonectes_bannaensis
HM067269_Limnonectes_bannaensis
HM067271_Limnonectes_bannaensis
AB526319_Limnonectes_bannaensis
HM067220_Limnonectes_bannaensis
HM067224_Limnonectes_bannaensis
HM067219_Limnonectes_bannaensis
HM067222_Limnonectes_bannaensis
HM067223_Limnonectes_bannaensis
HM067225_Limnonectes_bannaensis
HM067221_Limnonectes_bannaensis
HM067259_Limnonectes_bannaensis
HM067257_Limnonectes_bannaensis
HM067246_Limnonectes_bannaensis
HM067265_Limnonectes_bannaensis
HM067262_Limnonectes_bannaensis
HM067273_Limnonectes_bannaensis
HM067280_Limnonectes_bannaensis
HM067278_Limnonectes_bannaensis
HM067281_Limnonectes_bannaensis
HM067279_Limnonectes_bannaensis
HM067277_Limnonectes_bannaensis
HM067274_Limnonectes_bannaensis
HM067282_Limnonectes_bannaensis
HM067276_Limnonectes_bannaensis
HM067261_Limnonectes_bannaensis
HM067260_Limnonectes_bannaensis
HM067263_Limnonectes_bannaensis
HM067275_Limnonectes_bannaensis
HM067218_Limnonectes_bannaensis
HM067264_Limnonectes_bannaensis
HM067272_Limnonectes_bannaensis
HM067256_Limnonectes_bannaensis
HM067247_Limnonectes_bannaensis
HM067255_Limnonectes_bannaensis
HM067253_Limnonectes_bannaensis
HM067266_Limnonectes_bannaensis
HM067251_Limnonectes_bannaensis
HM067248_Limnonectes_bannaensis
HM067249_Limnonectes_bannaensis
KR827878_Limnonectes_bannaensis
KR827877_Limnonectes_bannaensis

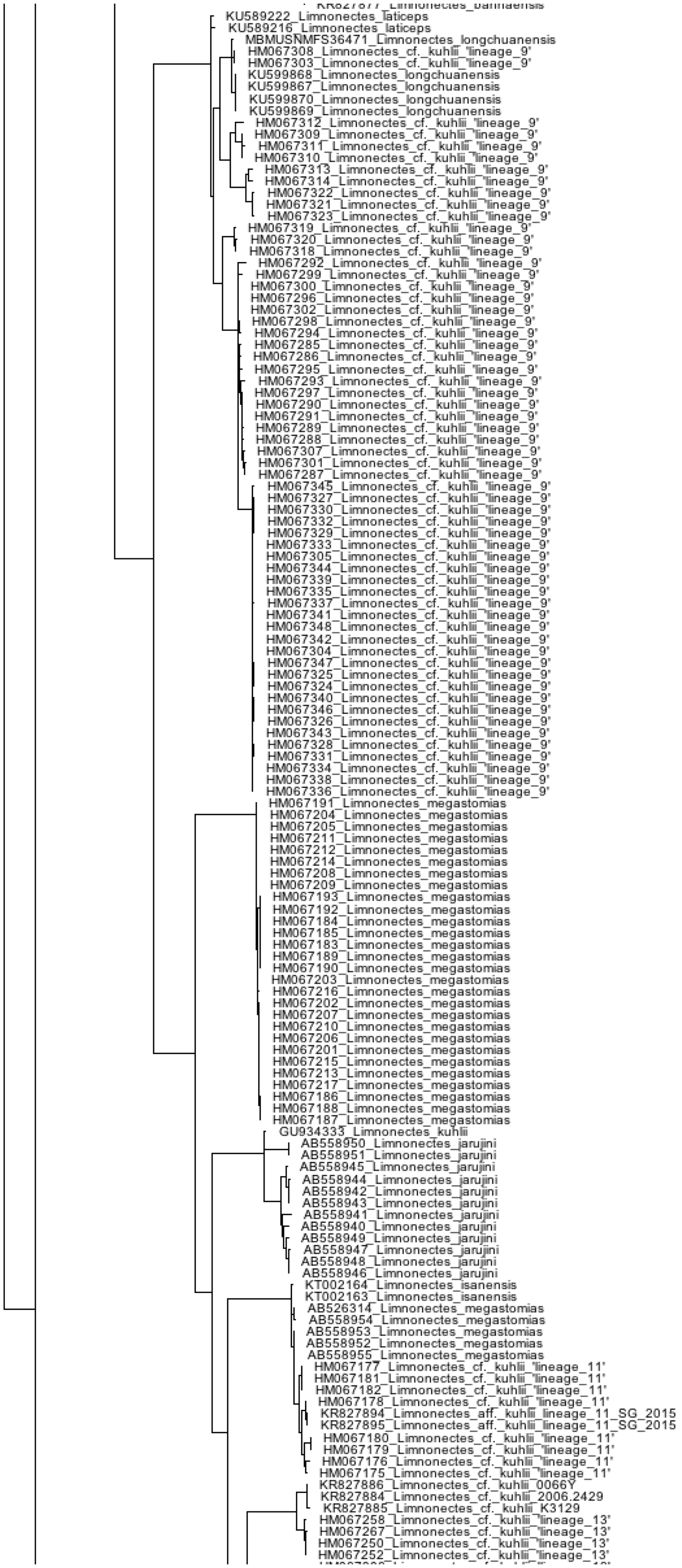
KU589222_Limnonectes_laticeps
KU589216_Limnonectes_laticeps
MBMUSNMFS36471_Limnonectes_longchuanensis
HM067308_Limnonectes_cf._kuhlii_'lineage_9'
HM067303_Limnonectes_cf._kuhlii_'lineage_9'
KU599868_Limnonectes_longchuanensis
KU599867_Limnonectes_longchuanensis
KU599870_Limnonectes_longchuanensis
KU599869_Limnonectes_longchuanensis
HM067312_Limnonectes_cf._kuhlii_'lineage_9'
HM067309_Limnonectes_cf._kuhlii_'lineage_9'
HM067311_Limnonectes_cf._kuhlii_'lineage_9'
HM067310_Limnonectes_cf._kuhlii_'lineage_9'
HM067313_Limnonectes_cf._kuhlii_'lineage_9'
HM067314_Limnonectes_cf._kuhlii_'lineage_9'
HM067322_Limnonectes_cf._kuhlii_'lineage_9'
HM067321_Limnonectes_cf._kuhlii_'lineage_9'
HM067323_Limnonectes_cf._kuhlii_'lineage_9'
HM067319_Limnonectes_cf._kuhlii_'lineage_9'
HM067320_Limnonectes_cf._kuhlii_'lineage_9'
HM067318_Limnonectes_cf._kuhlii_'lineage_9'
HM067292_Limnonectes_cf._kuhlii_'lineage_9'
HM067299_Limnonectes_cf._kuhlii_'lineage_9'
HM067300_Limnonectes_cf._kuhlii_'lineage_9'
HM067296_Limnonectes_cf._kuhlii_'lineage_9'
HM067302_Limnonectes_cf._kuhlii_'lineage_9'
HM067298_Limnonectes_cf._kuhlii_'lineage_9'
HM067294_Limnonectes_cf._kuhlii_'lineage_9'
HM067285_Limnonectes_cf._kuhlii_'lineage_9'
HM067286_Limnonectes_cf._kuhlii_'lineage_9'
HM067295_Limnonectes_cf._kuhlii_'lineage_9'
HM067293_Limnonectes_cf._kuhlii_'lineage_9'
HM067297_Limnonectes_cf._kuhlii_'lineage_9'
HM067290_Limnonectes_cf._kuhlii_'lineage_9'
HM067291_Limnonectes_cf._kuhlii_'lineage_9'
HM067289_Limnonectes_cf._kuhlii_'lineage_9'
HM067288_Limnonectes_cf._kuhlii_'lineage_9'
HM067307_Limnonectes_cf._kuhlii_'lineage_9'
HM067301_Limnonectes_cf._kuhlii_'lineage_9'
HM067287_Limnonectes_cf._kuhlii_'lineage_9'
HM067345_Limnonectes_cf._kuhlii_'lineage_9'
HM067327_Limnonectes_cf._kuhlii_'lineage_9'
HM067330_Limnonectes_cf._kuhlii_'lineage_9'
HM067332_Limnonectes_cf._kuhlii_'lineage_9'
HM067329_Limnonectes_cf._kuhlii_'lineage_9'
HM067333_Limnonectes_cf._kuhlii_'lineage_9'
HM067305_Limnonectes_cf._kuhlii_'lineage_9'
HM067344_Limnonectes_cf._kuhlii_'lineage_9'
HM067339_Limnonectes_cf._kuhlii_'lineage_9'
HM067335_Limnonectes_cf._kuhlii_'lineage_9'
HM067337_Limnonectes_cf._kuhlii_'lineage_9'
HM067341_Limnonectes_cf._kuhlii_'lineage_9'
HM067348_Limnonectes_cf._kuhlii_'lineage_9'
HM067342_Limnonectes_cf._kuhlii_'lineage_9'
HM067304_Limnonectes_cf._kuhlii_'lineage_9'
HM067347_Limnonectes_cf._kuhlii_'lineage_9'
HM067325_Limnonectes_cf._kuhlii_'lineage_9'
HM067324_Limnonectes_cf._kuhlii_'lineage_9'
HM067340_Limnonectes_cf._kuhlii_'lineage_9'
HM067346_Limnonectes_cf._kuhlii_'lineage_9'
HM067326_Limnonectes_cf._kuhlii_'lineage_9'
HM067343_Limnonectes_cf._kuhlii_'lineage_9'
HM067328_Limnonectes_cf._kuhlii_'lineage_9'
HM067331_Limnonectes_cf._kuhlii_'lineage_9'
HM067334_Limnonectes_cf._kuhlii_'lineage_9'
HM067338_Limnonectes_cf._kuhlii_'lineage_9'
HM067336_Limnonectes_cf._kuhlii_'lineage_9'
HM067191_Limnonectes_megastomias
HM067204_Limnonectes_megastomias
HM067205_Limnonectes_megastomias
HM067211_Limnonectes_megastomias
HM067212_Limnonectes_megastomias
HM067214_Limnonectes_megastomias
HM067208_Limnonectes_megastomias
HM067209_Limnonectes_megastomias
HM067193_Limnonectes_megastomias
HM067192_Limnonectes_megastomias
HM067184_Limnonectes_megastomias
HM067185_Limnonectes_megastomias
HM067183_Limnonectes_megastomias
HM067189_Limnonectes_megastomias
HM067190_Limnonectes_megastomias
HM067203_Limnonectes_megastomias
HM067216_Limnonectes_megastomias
HM067202_Limnonectes_megastomias
HM067207_Limnonectes_megastomias
HM067210_Limnonectes_megastomias
HM067206_Limnonectes_megastomias
HM067201_Limnonectes_megastomias
HM067215_Limnonectes_megastomias
HM067213_Limnonectes_megastomias
HM067217_Limnonectes_megastomias
HM067186_Limnonectes_megastomias
HM067188_Limnonectes_megastomias
HM067187_Limnonectes_megastomias
GU934333_Limnonectes_kuhlii
AB558950_Limnonectes_jarujini
AB558951_Limnonectes_jarujini
AB558945_Limnonectes_jarujini
AB558944_Limnonectes_jarujini
AB558942_Limnonectes_jarujini
AB558943_Limnonectes_jarujini
AB558941_Limnonectes_jarujini
AB558940_Limnonectes_jarujini
AB558949_Limnonectes_jarujini
AB558947_Limnonectes_jarujini
AB558948_Limnonectes_jarujini
AB558946_Limnonectes_jarujini
KT002164_Limnonectes_isanensis
KT002163_Limnonectes_isanensis
AB526314_Limnonectes_megastomias
AB558954_Limnonectes_megastomias
AB558953_Limnonectes_megastomias
AB558952_Limnonectes_megastomias
AB558955_Limnonectes_megastomias
HM067177_Limnonectes_cf._kuhlii_'lineage_11'
HM067181_Limnonectes_cf._kuhlii_'lineage_11'
HM067182_Limnonectes_cf._kuhlii_'lineage_11'
HM067178_Limnonectes_cf._kuhlii_'lineage_11'
KR827894_Limnonectes_aff._kuhlii_lineage_11_SG_2015
KR827895_Limnonectes_aff._kuhlii_lineage_11_SG_2015
HM067180_Limnonectes_cf._kuhlii_'lineage_11'
HM067179_Limnonectes_cf._kuhlii_'lineage_11'
HM067176_Limnonectes_cf._kuhlii_'lineage_11'
HM067175_Limnonectes_cf._kuhlii_'lineage_11'
KR827886_Limnonectes_cf._kuhlii_0066Y
KR827884_Limnonectes_cf._kuhlii_2006.2429
KR827885_Limnonectes_cf._kuhlii_K3129
HM067258_Limnonectes_cf._kuhlii_'lineage_13'
HM067267_Limnonectes_cf._kuhlii_'lineage_13'
HM067250_Limnonectes_cf._kuhlii_'lineage_13'
HM067252_Limnonectes_cf._kuhlii_'lineage_13'

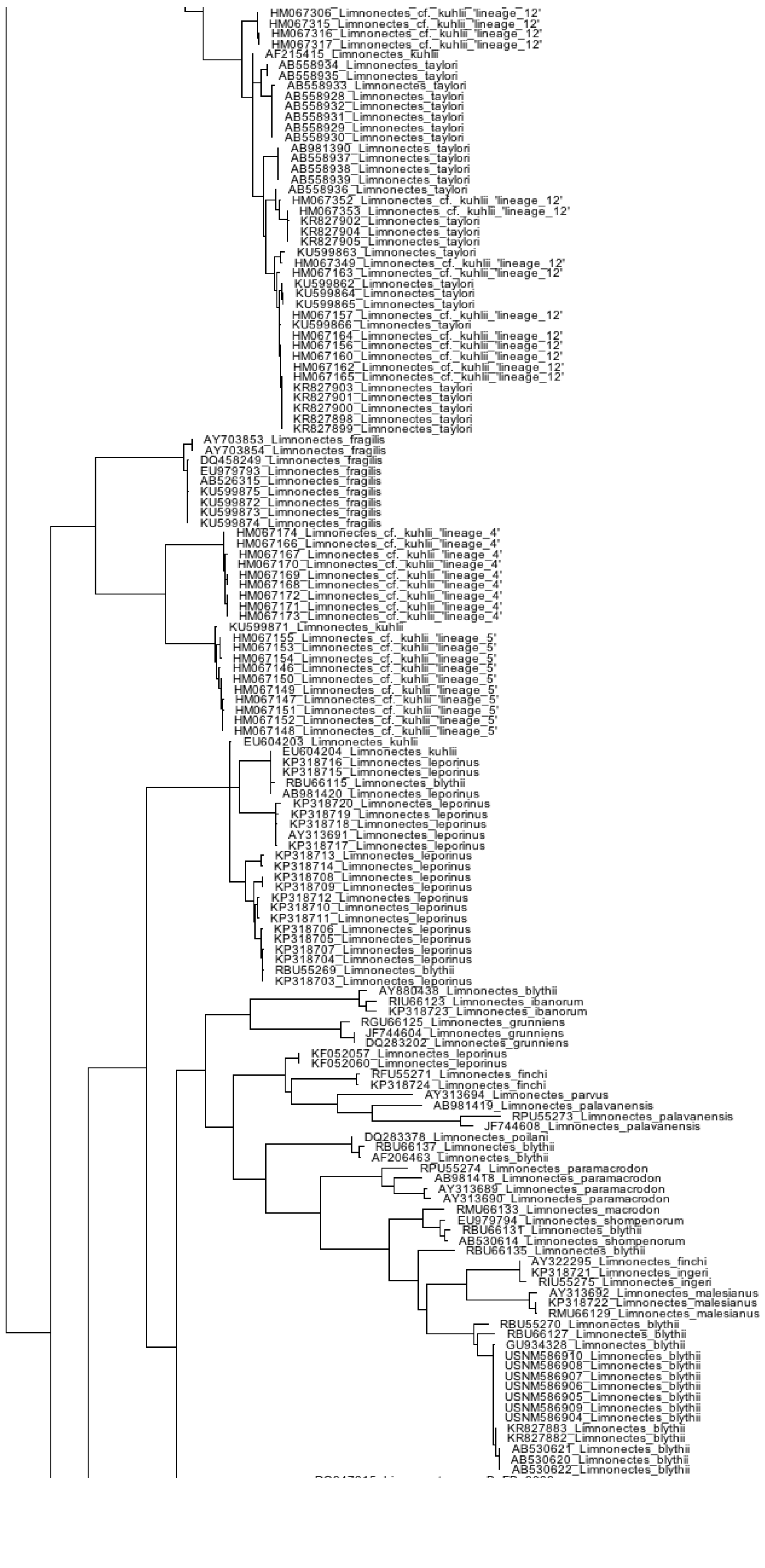
HM067306_Limnonectes_cf._kuhlii_'lineage_12'
HM067315_Limnonectes_cf._kuhlii_'lineage_12'
HM067316_Limnonectes_cf._kuhlii_'lineage_12'
HM067317_Limnonectes_cf._kuhlii_'lineage_12'
AF215415_Limnonectes_kuhlii
AB558934_Limnonectes_taylori
AB558935_Limnonectes_taylori
AB558933_Limnonectes_taylori
AB558928_Limnonectes_taylori
AB558932_Limnonectes_taylori
AB558931_Limnonectes_taylori
AB558929_Limnonectes_taylori
AB558930_Limnonectes_taylori
AB981390_Limnonectes_taylori
AB558937_Limnonectes_taylori
AB558938_Limnonectes_taylori
AB558939_Limnonectes_taylori
AB558936_Limnonectes_taylori
HM067352_Limnonectes_cf._kuhlii_'lineage_12'
HM067353_Limnonectes_cf._kuhlii_'lineage_12'
KR827902_Limnonectes_taylori
KR827904_Limnonectes_taylori
KR827905_Limnonectes_taylori
KU599863_Limnonectes_taylori
HM067349_Limnonectes_cf._kuhlii_'lineage_12'
HM067163_Limnonectes_cf._kuhlii_'lineage_12'
KU599862_Limnonectes_taylori
KU599864_Limnonectes_taylori
KU599865_Limnonectes_taylori
HM067157_Limnonectes_cf._kuhlii_'lineage_12'
KU599866_Limnonectes_taylori
HM067164_Limnonectes_cf._kuhlii_'lineage_12'
HM067156_Limnonectes_cf._kuhlii_'lineage_12'
HM067160_Limnonectes_cf._kuhlii_'lineage_12'
HM067162_Limnonectes_cf._kuhlii_'lineage_12'
HM067165_Limnonectes_cf._kuhlii_'lineage_12'
KR827903_Limnonectes_taylori
KR827901_Limnonectes_taylori
KR827900_Limnonectes_taylori
KR827898_Limnonectes_taylori
KR827899_Limnonectes_taylori
AY703853_Limnonectes_fragilis
AY703854_Limnonectes_fragilis
DQ458249_Limnonectes_fragilis
EU979793_Limnonectes_fragilis
AB526315_Limnonectes_fragilis
KU599875_Limnonectes_fragilis
KU599872_Limnonectes_fragilis
KU599873_Limnonectes_fragilis
KU599874_Limnonectes_fragilis
HM067174_Limnonectes_cf._kuhlii_'lineage_4'
HM067166_Limnonectes_cf._kuhlii_'lineage_4'
HM067167_Limnonectes_cf._kuhlii_'lineage_4'
HM067170_Limnonectes_cf._kuhlii_'lineage_4'
HM067169_Limnonectes_cf._kuhlii_'lineage_4'
HM067168_Limnonectes_cf._kuhlii_'lineage_4'
HM067172_Limnonectes_cf._kuhlii_'lineage_4'
HM067171_Limnonectes_cf._kuhlii_'lineage_4'
HM067173_Limnonectes_cf._kuhlii_'lineage_4'
KU599871_Limnonectes_kuhlii
HM067155_Limnonectes_cf._kuhlii_'lineage_5'
HM067153_Limnonectes_cf._kuhlii_'lineage_5'
HM067154_Limnonectes_cf._kuhlii_'lineage_5'
HM067146_Limnonectes_cf._kuhlii_'lineage_5'
HM067150_Limnonectes_cf._kuhlii_'lineage_5'
HM067149_Limnonectes_cf._kuhlii_'lineage_5'
HM067147_Limnonectes_cf._kuhlii_'lineage_5'
HM067151_Limnonectes_cf._kuhlii_'lineage_5'
HM067152_Limnonectes_cf._kuhlii_'lineage_5'
HM067148_Limnonectes_cf._kuhlii_'lineage_5'
EU604203_Limnonectes_kuhlii
EU604204_Limnonectes_kuhlii
KP318716_Limnonectes_leporinus
KP318715_Limnonectes_leporinus
RBU66115_Limnonectes_blythii
AB981420_Limnonectes_leporinus
KP318720_Limnonectes_leporinus
KP318719_Limnonectes_leporinus
KP318718_Limnonectes_leporinus
AY313691_Limnonectes_leporinus
KP318717_Limnonectes_leporinus
KP318713_Limnonectes_leporinus
KP318714_Limnonectes_leporinus
KP318708_Limnonectes_leporinus
KP318709_Limnonectes_leporinus
KP318712_Limnonectes_leporinus
KP318710_Limnonectes_leporinus
KP318711_Limnonectes_leporinus
KP318706_Limnonectes_leporinus
KP318705_Limnonectes_leporinus
KP318707_Limnonectes_leporinus
KP318704_Limnonectes_leporinus
RBU55269_Limnonectes_blythii
KP318703_Limnonectes_leporinus
AY880438_Limnonectes_blythii
RIU66123_Limnonectes_ibanorum
KP318723_Limnonectes_ibanorum
RGU66125_Limnonectes_grunniens
JF744604_Limnonectes_grunniens
DQ283202_Limnonectes_grunniens
KF052057_Limnonectes_leporinus
KF052060_Limnonectes_leporinus
RFU55271_Limnonectes_finchi
KP318724_Limnonectes_finchi
AY313694_Limnonectes_parvus
AB981419_Limnonectes_palavanensis
RPU55273_Limnonectes_palavanensis
JF744608_Limnonectes_palavanensis
DQ283378_Limnonectes_poilani
RBU66137_Limnonectes_blythii
AF206463_Limnonectes_blythii
RPU55274_Limnonectes_paramacrodon
AB981418_Limnonectes_paramacrodon
AY313689_Limnonectes_paramacrodon
AY313690_Limnonectes_paramacrodon
RMU66133_Limnonectes_macrodon
EU979794_Limnonectes_shompenorum
RBU66131_Limnonectes_blythii
AB530614_Limnonectes_shompenorum
RBU66135_Limnonectes_blythii
AY322295_Limnonectes_finchi
KP318721_Limnonectes_ingeri
RIU55275_Limnonectes_ingeri
AY313692_Limnonectes_malesianus
KP318722_Limnonectes_malesianus
RMU66129_Limnonectes_malesianus
RBU55270_Limnonectes_blythii
RBU66127_Limnonectes_blythii
GU934328_Limnonectes_blythii
USNM586910_Limnonectes_blythii
USNM586908_Limnonectes_blythii
USNM586907_Limnonectes_blythii
USNM586906_Limnonectes_blythii
USNM586905_Limnonectes_blythii
USNM586909_Limnonectes_blythii
USNM586904_Limnonectes_blythii
KR827883_Limnonectes_blythii
KR827882_Limnonectes_blythii
AB530621_Limnonectes_blythii
AB530620_Limnonectes_blythii
AB530622_Limnonectes_blythii

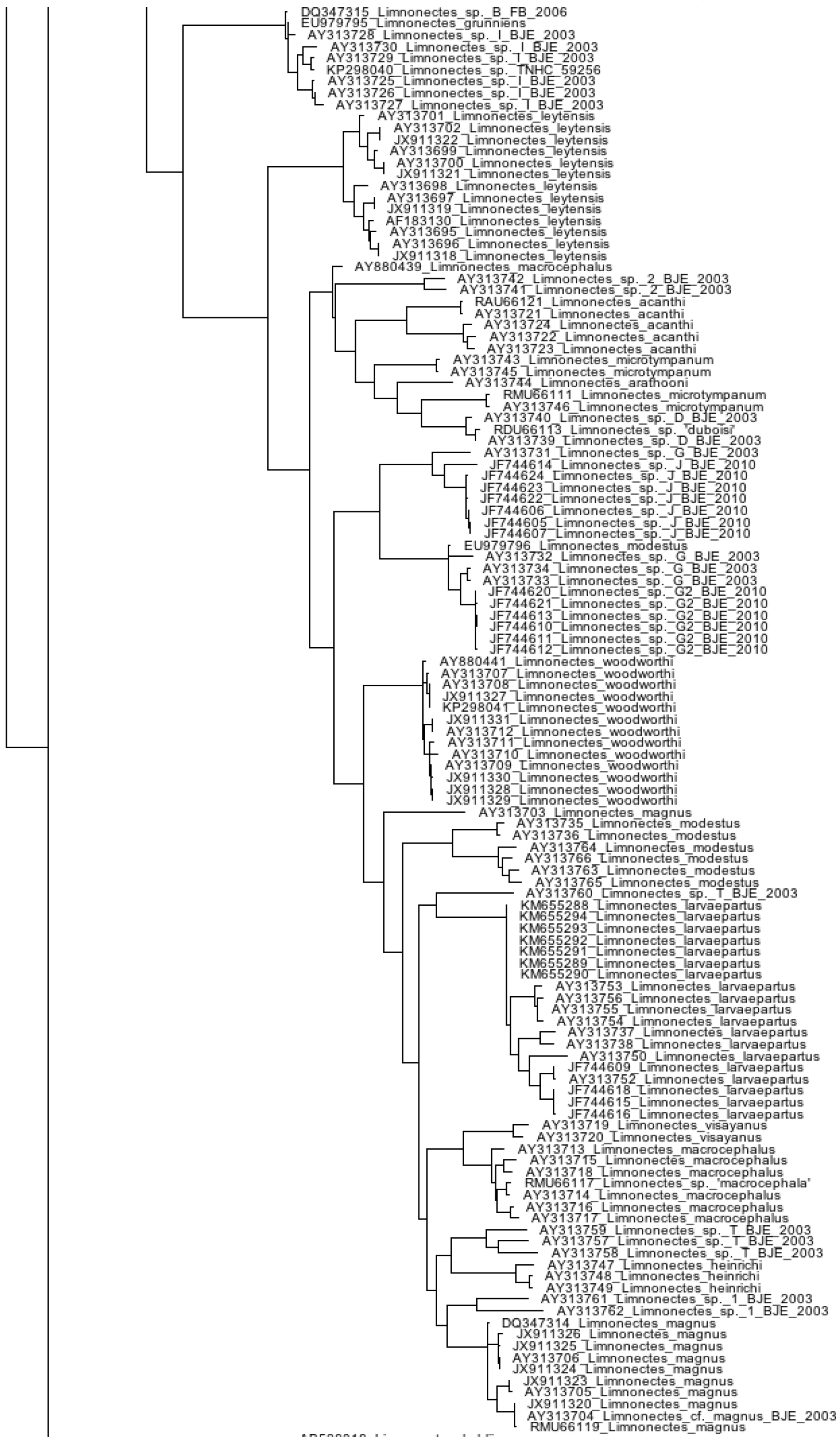
DQ347315_Limnonectes_sp._B_FB_2006
EU979795_Limnonectes_grunniens
AY313728_Limnonectes_sp._I_BJE_2003
AY313730_Limnonectes_sp._I_BJE_2003
AY313729_Limnonectes_sp._I_BJE_2003
KP298040_Limnonectes_sp._TNHC_59256
AY313725_Limnonectes_sp._I_BJE_2003
AY313726_Limnonectes_sp._I_BJE_2003
AY313727_Limnonectes_sp._I_BJE_2003
AY313701_Limnonectes_leytensis
AY313702_Limnonectes_leytensis
JX911322_Limnonectes_leytensis
AY313699_Limnonectes_leytensis
AY313700_Limnonectes_leytensis
JX911321_Limnonectes_leytensis
AY313698_Limnonectes_leytensis
AY313697_Limnonectes_leytensis
JX911319_Limnonectes_leytensis
AF183130_Limnonectes_leytensis
AY313695_Limnonectes_leytensis
AY313696_Limnonectes_leytensis
JX911318_Limnonectes_leytensis
AY880439_Limnonectes_macrocephalus
AY313742_Limnonectes_sp._2_BJE_2003
AY313741_Limnonectes_sp._2_BJE_2003
RAU66121_Limnonectes_acanthi
AY313721_Limnonectes_acanthi
AY313724_Limnonectes_acanthi
AY313722_Limnonectes_acanthi
AY313723_Limnonectes_acanthi
AY313743_Limnonectes_microtympanum
AY313745_Limnonectes_microtympanum
AY313744_Limnonectes_arathooni
RMU66111_Limnonectes_microtympanum
AY313746_Limnonectes_microtympanum
AY313740_Limnonectes_sp._D_BJE_2003
RDU66113_Limnonectes_sp._'duboisi'
AY313739_Limnonectes_sp._D_BJE_2003
AY313731_Limnonectes_sp._G_BJE_2003
JF744614_Limnonectes_sp._J_BJE_2010
JF744624_Limnonectes_sp._J_BJE_2010
JF744623_Limnonectes_sp._J_BJE_2010
JF744622_Limnonectes_sp._J_BJE_2010
JF744606_Limnonectes_sp._J_BJE_2010
JF744605_Limnonectes_sp._J_BJE_2010
JF744607_Limnonectes_sp._J_BJE_2010
EU979796_Limnonectes_modestus
AY313732_Limnonectes_sp._G_BJE_2003
AY313734_Limnonectes_sp._G_BJE_2003
AY313733_Limnonectes_sp._G_BJE_2003
JF744620_Limnonectes_sp._G2_BJE_2010
JF744621_Limnonectes_sp._G2_BJE_2010
JF744613_Limnonectes_sp._G2_BJE_2010
JF744610_Limnonectes_sp._G2_BJE_2010
JF744611_Limnonectes_sp._G2_BJE_2010
JF744612_Limnonectes_sp._G2_BJE_2010
AY880441_Limnonectes_woodworthi
AY313707_Limnonectes_woodworthi
AY313708_Limnonectes_woodworthi
JX911327_Limnonectes_woodworthi
KP298041_Limnonectes_woodworthi
JX911331_Limnonectes_woodworthi
AY313712_Limnonectes_woodworthi
AY313711_Limnonectes_woodworthi
AY313710_Limnonectes_woodworthi
AY313709_Limnonectes_woodworthi
JX911330_Limnonectes_woodworthi
JX911328_Limnonectes_woodworthi
JX911329_Limnonectes_woodworthi
AY313703_Limnonectes_magnus
AY313735_Limnonectes_modestus
AY313736_Limnonectes_modestus
AY313764_Limnonectes_modestus
AY313766_Limnonectes_modestus
AY313763_Limnonectes_modestus
AY313765_Limnonectes_modestus
AY313760_Limnonectes_sp._T_BJE_2003
KM655288_Limnonectes_larvaepartus
KM655294_Limnonectes_larvaepartus
KM655293_Limnonectes_larvaepartus
KM655292_Limnonectes_larvaepartus
KM655291_Limnonectes_larvaepartus
KM655289_Limnonectes_larvaepartus
KM655290_Limnonectes_larvaepartus
AY313753_Limnonectes_larvaepartus
AY313756_Limnonectes_larvaepartus
AY313755_Limnonectes_larvaepartus
AY313754_Limnonectes_larvaepartus
AY313737_Limnonectes_larvaepartus
AY313738_Limnonectes_larvaepartus
AY313750_Limnonectes_larvaepartus
JF744609_Limnonectes_larvaepartus
AY313752_Limnonectes_larvaepartus
JF744618_Limnonectes_larvaepartus
JF744615_Limnonectes_larvaepartus
JF744616_Limnonectes_larvaepartus
AY313719_Limnonectes_visayanus
AY313720_Limnonectes_visayanus
AY313713_Limnonectes_macrocephalus
AY313715_Limnonectes_macrocephalus
AY313718_Limnonectes_macrocephalus
RMU66117_Limnonectes_sp._'macrocephala'
AY313714_Limnonectes_macrocephalus
AY313716_Limnonectes_macrocephalus
AY313717_Limnonectes_macrocephalus
AY313759_Limnonectes_sp._T_BJE_2003
AY313757_Limnonectes_sp._T_BJE_2003
AY313758_Limnonectes_sp._T_BJE_2003
AY313747_Limnonectes_heinrichi
AY313748_Limnonectes_heinrichi
AY313749_Limnonectes_heinrichi
AY313761_Limnonectes_sp._1_BJE_2003
AY313762_Limnonectes_sp._1_BJE_2003
DQ347314_Limnonectes_magnus
JX911326_Limnonectes_magnus
JX911325_Limnonectes_magnus
AY313706_Limnonectes_magnus
JX911324_Limnonectes_magnus
JX911323_Limnonectes_magnus
AY313705_Limnonectes_magnus
JX911320_Limnonectes_magnus
AY313704_Limnonectes_cf._magnus_BJE_2003
RMU66119_Limnonectes_magnus

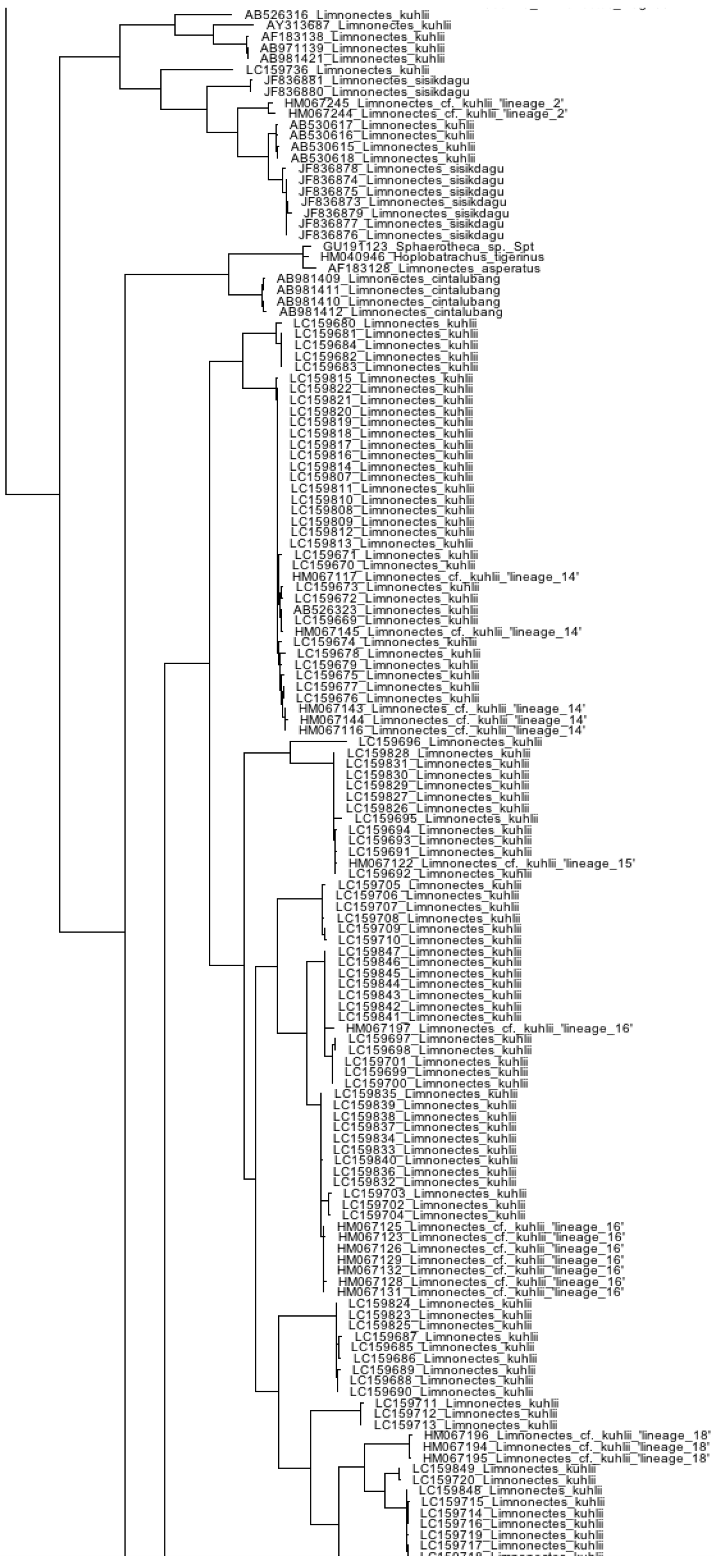
AB526316_Limnonectes_kuhlii
AY313687_Limnonectes_kuhlii
AF183138_Limnonectes_kuhlii
AB971139_Limnonectes_kuhlii
AB981421_Limnonectes_kuhlii
LC159736_Limnonectes_kuhlii
JF836881_Limnonectes_sisikdagu
JF836880_Limnonectes_sisikdagu
HM067245_Limnonectes_cf._kuhlii_'lineage_2'
HM067244_Limnonectes_cf._kuhlii_'lineage_2'
AB530617_Limnonectes_kuhlii
AB530616_Limnonectes_kuhlii
AB530615_Limnonectes_kuhlii
AB530618_Limnonectes_kuhlii
JF836878_Limnonectes_sisikdagu
JF836874_Limnonectes_sisikdagu
JF836875_Limnonectes_sisikdagu
JF836873_Limnonectes_sisikdagu
JF836879_Limnonectes_sisikdagu
JF836877_Limnonectes_sisikdagu
JF836876_Limnonectes_sisikdagu
GU191123_Sphaerotheca_sp._Spt
HM040946_Hoplobatrachus_tigerinus
AF183128_Limnonectes_asperatus
AB981409_Limnonectes_cintalubang
AB981411_Limnonectes_cintalubang
AB981410_Limnonectes_cintalubang
AB981412_Limnonectes_cintalubang
LC159680_Limnonectes_kuhlii
LC159681_Limnonectes_kuhlii
LC159684_Limnonectes_kuhlii
LC159682_Limnonectes_kuhlii
LC159683_Limnonectes_kuhlii
LC159815_Limnonectes_kuhlii
LC159822_Limnonectes_kuhlii
LC159821_Limnonectes_kuhlii
LC159820_Limnonectes_kuhlii
LC159819_Limnonectes_kuhlii
LC159818_Limnonectes_kuhlii
LC159817_Limnonectes_kuhlii
LC159816_Limnonectes_kuhlii
LC159814_Limnonectes_kuhlii
LC159807_Limnonectes_kuhlii
LC159811_Limnonectes_kuhlii
LC159810_Limnonectes_kuhlii
LC159808_Limnonectes_kuhlii
LC159809_Limnonectes_kuhlii
LC159812_Limnonectes_kuhlii
LC159813_Limnonectes_kuhlii
LC159671_Limnonectes_kuhlii
LC159670_Limnonectes_kuhlii
HM067117_Limnonectes_cf._kuhlii_'lineage_14'
LC159673_Limnonectes_kuhlii
LC159672_Limnonectes_kuhlii
AB526323_Limnonectes_kuhlii
LC159669_Limnonectes_kuhlii
HM067145_Limnonectes_cf._kuhlii_'lineage_14'
LC159674_Limnonectes_kuhlii
LC159678_Limnonectes_kuhlii
LC159679_Limnonectes_kuhlii
LC159675_Limnonectes_kuhlii
LC159677_Limnonectes_kuhlii
LC159676_Limnonectes_kuhlii
HM067143_Limnonectes_cf._kuhlii_'lineage_14'
HM067144_Limnonectes_cf._kuhlii_'lineage_14'
HM067116_Limnonectes_cf._kuhlii_'lineage_14'
LC159696_Limnonectes_kuhlii
LC159828_Limnonectes_kuhlii
LC159831_Limnonectes_kuhlii
LC159830_Limnonectes_kuhlii
LC159829_Limnonectes_kuhlii
LC159827_Limnonectes_kuhlii
LC159826_Limnonectes_kuhlii
LC159695_Limnonectes_kuhlii
LC159694_Limnonectes_kuhlii
LC159693_Limnonectes_kuhlii
LC159691_Limnonectes_kuhlii
HM067122_Limnonectes_cf._kuhlii_'lineage_15'
LC159692_Limnonectes_kuhlii
LC159705_Limnonectes_kuhlii
LC159706_Limnonectes_kuhlii
LC159707_Limnonectes_kuhlii
LC159708_Limnonectes_kuhlii
LC159709_Limnonectes_kuhlii
LC159710_Limnonectes_kuhlii
LC159847_Limnonectes_kuhlii
LC159846_Limnonectes_kuhlii
LC159845_Limnonectes_kuhlii
LC159844_Limnonectes_kuhlii
LC159843_Limnonectes_kuhlii
LC159842_Limnonectes_kuhlii
LC159841_Limnonectes_kuhlii
HM067197_Limnonectes_cf._kuhlii_'lineage_16'
LC159697_Limnonectes_kuhlii
LC159698_Limnonectes_kuhlii
LC159701_Limnonectes_kuhlii
LC159699_Limnonectes_kuhlii
LC159700_Limnonectes_kuhlii
LC159835_Limnonectes_kuhlii
LC159839_Limnonectes_kuhlii
LC159838_Limnonectes_kuhlii
LC159837_Limnonectes_kuhlii
LC159834_Limnonectes_kuhlii
LC159833_Limnonectes_kuhlii
LC159840_Limnonectes_kuhlii
LC159836_Limnonectes_kuhlii
LC159832_Limnonectes_kuhlii
LC159703_Limnonectes_kuhlii
LC159702_Limnonectes_kuhlii
LC159704_Limnonectes_kuhlii
HM067125_Limnonectes_cf._kuhlii_'lineage_16'
HM067123_Limnonectes_cf._kuhlii_'lineage_16'
HM067126_Limnonectes_cf._kuhlii_'lineage_16'
HM067129_Limnonectes_cf._kuhlii_'lineage_16'
HM067132_Limnonectes_cf._kuhlii_'lineage_16'
HM067128_Limnonectes_cf._kuhlii_'lineage_16'
HM067131_Limnonectes_cf._kuhlii_'lineage_16'
LC159824_Limnonectes_kuhlii
LC159823_Limnonectes_kuhlii
LC159825_Limnonectes_kuhlii
LC159687_Limnonectes_kuhlii
LC159685_Limnonectes_kuhlii
LC159686_Limnonectes_kuhlii
LC159689_Limnonectes_kuhlii
LC159688_Limnonectes_kuhlii
LC159690_Limnonectes_kuhlii
LC159711_Limnonectes_kuhlii
LC159712_Limnonectes_kuhlii
LC159713_Limnonectes_kuhlii
HM067196_Limnonectes_cf._kuhlii_'lineage_18'
HM067194_Limnonectes_cf._kuhlii_'lineage_18'
HM067195_Limnonectes_cf._kuhlii_'lineage_18'
LC159849_Limnonectes_kuhlii
LC159720_Limnonectes_kuhlii
LC159848_Limnonectes_kuhlii
LC159715_Limnonectes_kuhlii
LC159714_Limnonectes_kuhlii
LC159716_Limnonectes_kuhlii
LC159719_Limnonectes_kuhlii
LC159717_Limnonectes_kuhlii

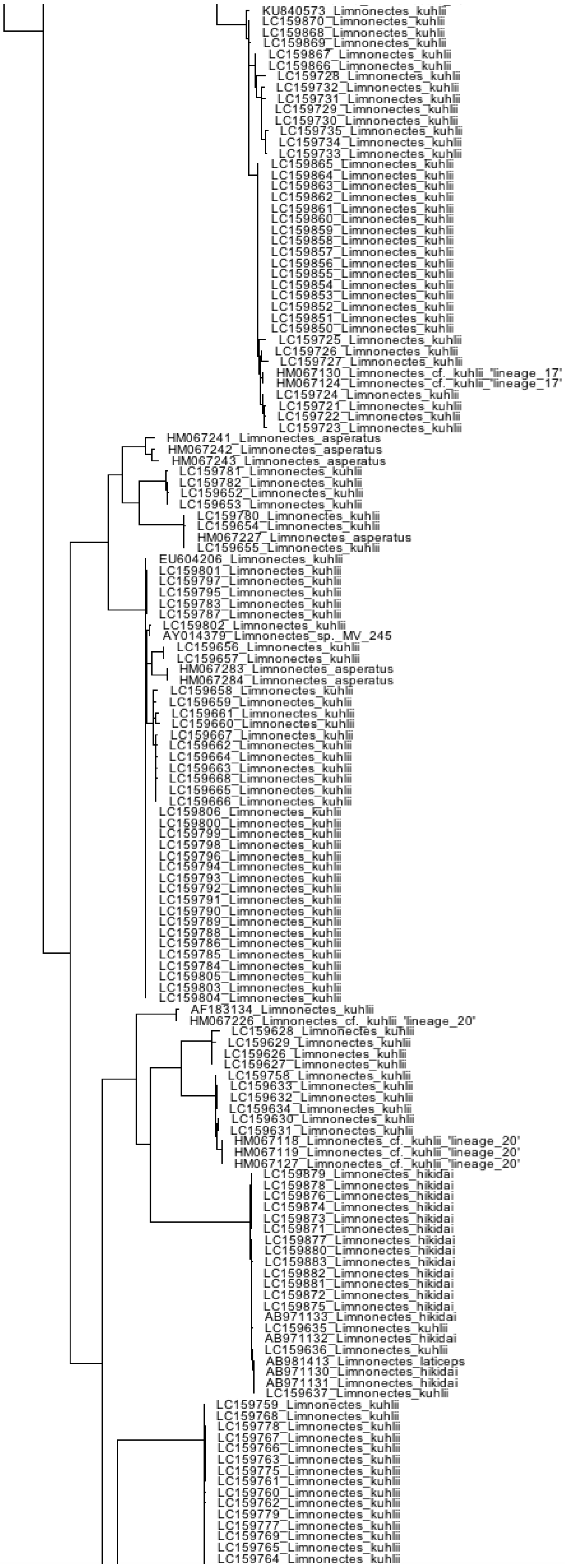

KU840573_Limnonectes_kuhlii
LC159870_Limnonectes_kuhlii
LC159868_Limnonectes_kuhlii
LC159869_Limnonectes_kuhlii
LC159867_Limnonectes_kuhlii
LC159866_Limnonectes_kuhlii
LC159728_Limnonectes_kuhlii
LC159732_Limnonectes_kuhlii
LC159731_Limnonectes_kuhlii
LC159729_Limnonectes_kuhlii
LC159730_Limnonectes_kuhlii
LC159735_Limnonectes_kuhlii
LC159734_Limnonectes_kuhlii
LC159733_Limnonectes_kuhlii
LC159865_Limnonectes_kuhlii
LC159864_Limnonectes_kuhlii
LC159863_Limnonectes_kuhlii
LC159862_Limnonectes_kuhlii
LC159861_Limnonectes_kuhlii
LC159860_Limnonectes_kuhlii
LC159859_Limnonectes_kuhlii
LC159858_Limnonectes_kuhlii
LC159857_Limnonectes_kuhlii
LC159856_Limnonectes_kuhlii
LC159855_Limnonectes_kuhlii
LC159854_Limnonectes_kuhlii
LC159853_Limnonectes_kuhlii
LC159852_Limnonectes_kuhlii
LC159851_Limnonectes_kuhlii
LC159850_Limnonectes_kuhlii
LC159725_Limnonectes_kuhlii
LC159726_Limnonectes_kuhlii
LC159727_Limnonectes_kuhlii
HM067130_Limnonectes_cf._kuhlii_"lineage_17"
HM067124_Limnonectes_cf._kuhlii_"lineage_17"
LC159724_Limnonectes_kuhlii
LC159721_Limnonectes_kuhlii
LC159722_Limnonectes_kuhlii
LC159723_Limnonectes_kuhlii
HM067241_Limnonectes_asperatus
HM067242_Limnonectes_asperatus
HM067243_Limnonectes_asperatus
LC159781_Limnonectes_kuhlii
LC159782_Limnonectes_kuhlii
LC159652_Limnonectes_kuhlii
LC159653_Limnonectes_kuhlii
LC159780_Limnonectes_kuhlii
LC159654_Limnonectes_kuhlii
HM067227_Limnonectes_asperatus
LC159655_Limnonectes_kuhlii
EU604206_Limnonectes_kuhlii
LC159801_Limnonectes_kuhlii
LC159797_Limnonectes_kuhlii
LC159795_Limnonectes_kuhlii
LC159783_Limnonectes_kuhlii
LC159787_Limnonectes_kuhlii
LC159802_Limnonectes_kuhlii
AY014379_Limnonectes_sp._MV_245
LC159656_Limnonectes_kuhlii
LC159657_Limnonectes_kuhlii
HM067283_Limnonectes_asperatus
HM067284_Limnonectes_asperatus
LC159658_Limnonectes_kuhlii
LC159659_Limnonectes_kuhlii
LC159661_Limnonectes_kuhlii
LC159660_Limnonectes_kuhlii
LC159667_Limnonectes_kuhlii
LC159662_Limnonectes_kuhlii
LC159664_Limnonectes_kuhlii
LC159663_Limnonectes_kuhlii
LC159668_Limnonectes_kuhlii
LC159665_Limnonectes_kuhlii
LC159666_Limnonectes_kuhlii
LC159806_Limnonectes_kuhlii
LC159800_Limnonectes_kuhlii
LC159799_Limnonectes_kuhlii
LC159798_Limnonectes_kuhlii
LC159796_Limnonectes_kuhlii
LC159794_Limnonectes_kuhlii
LC159793_Limnonectes_kuhlii
LC159792_Limnonectes_kuhlii
LC159791_Limnonectes_kuhlii
LC159790_Limnonectes_kuhlii
LC159789_Limnonectes_kuhlii
LC159788_Limnonectes_kuhlii
LC159786_Limnonectes_kuhlii
LC159785_Limnonectes_kuhlii
LC159784_Limnonectes_kuhlii
LC159805_Limnonectes_kuhlii
LC159803_Limnonectes_kuhlii
LC159804_Limnonectes_kuhlii
AF183134_Limnonectes_kuhlii
HM067226_Limnonectes_cf._kuhlii_"lineage_20"
LC159628_Limnonectes_kuhlii
LC159629_Limnonectes_kuhlii
LC159626_Limnonectes_kuhlii
LC159627_Limnonectes_kuhlii
LC159758_Limnonectes_kuhlii
LC159633_Limnonectes_kuhlii
LC159632_Limnonectes_kuhlii
LC159634_Limnonectes_kuhlii
LC159630_Limnonectes_kuhlii
LC159631_Limnonectes_kuhlii
HM067118_Limnonectes_cf._kuhlii_"lineage_20"
HM067119_Limnonectes_cf._kuhlii_"lineage_20"
HM067127_Limnonectes_cf._kuhlii_"lineage_20"
LC159879_Limnonectes_hikidai
LC159878_Limnonectes_hikidai
LC159876_Limnonectes_hikidai
LC159874_Limnonectes_hikidai
LC159873_Limnonectes_hikidai
LC159871_Limnonectes_hikidai
LC159877_Limnonectes_hikidai
LC159880_Limnonectes_hikidai
LC159883_Limnonectes_hikidai
LC159882_Limnonectes_hikidai
LC159881_Limnonectes_hikidai
LC159872_Limnonectes_hikidai
LC159875_Limnonectes_hikidai
AB971133_Limnonectes_hikidai
LC159635_Limnonectes_kuhlii
AB971132_Limnonectes_hikidai
LC159636_Limnonectes_kuhlii
AB981413_Limnonectes_laticeps
AB971130_Limnonectes_hikidai
AB971131_Limnonectes_hikidai
LC159637_Limnonectes_kuhlii
LC159759_Limnonectes_kuhlii
LC159768_Limnonectes_kuhlii
LC159778_Limnonectes_kuhlii
LC159767_Limnonectes_kuhlii
LC159766_Limnonectes_kuhlii
LC159763_Limnonectes_kuhlii
LC159775_Limnonectes_kuhlii
LC159761_Limnonectes_kuhlii
LC159760_Limnonectes_kuhlii
LC159762_Limnonectes_kuhlii
LC159779_Limnonectes_kuhlii
LC159777_Limnonectes_kuhlii
LC159769_Limnonectes_kuhlii
LC159765_Limnonectes_kuhlii
LC159764_Limnonectes_kuhlii

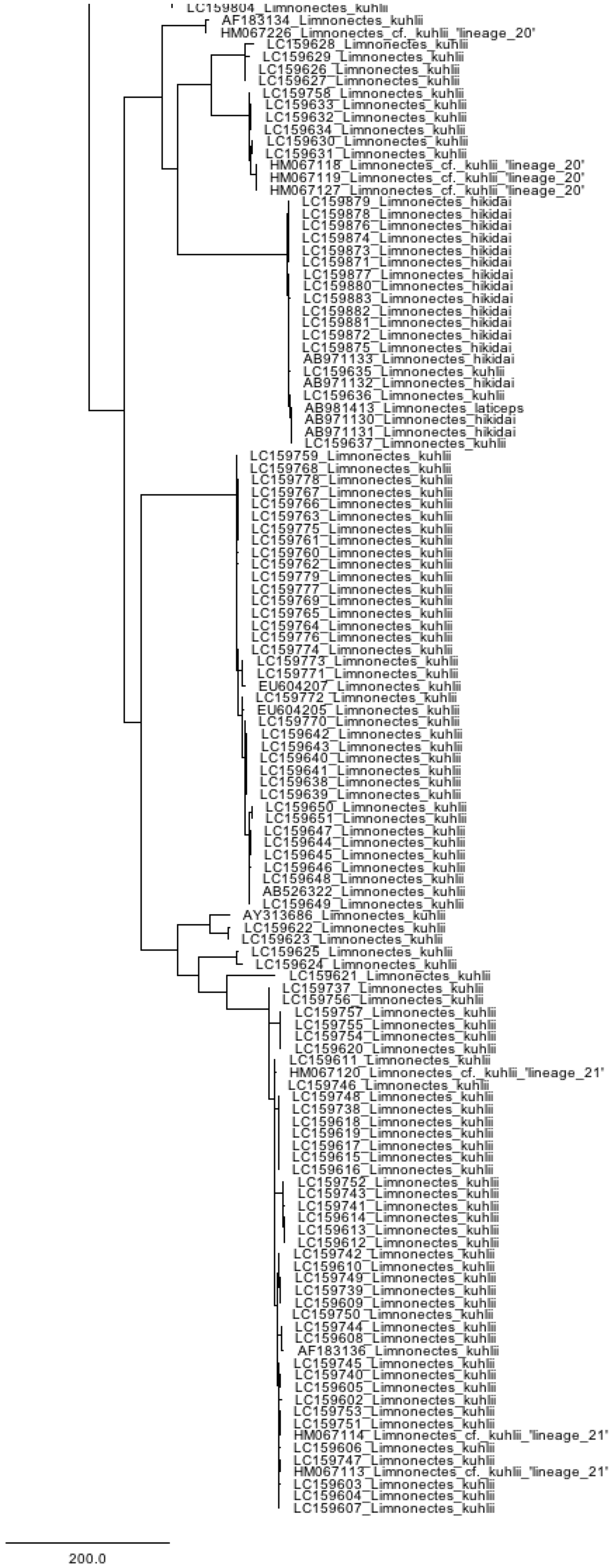

Fig. 3. Dicroglossidae 16S neighbor-joining tree.

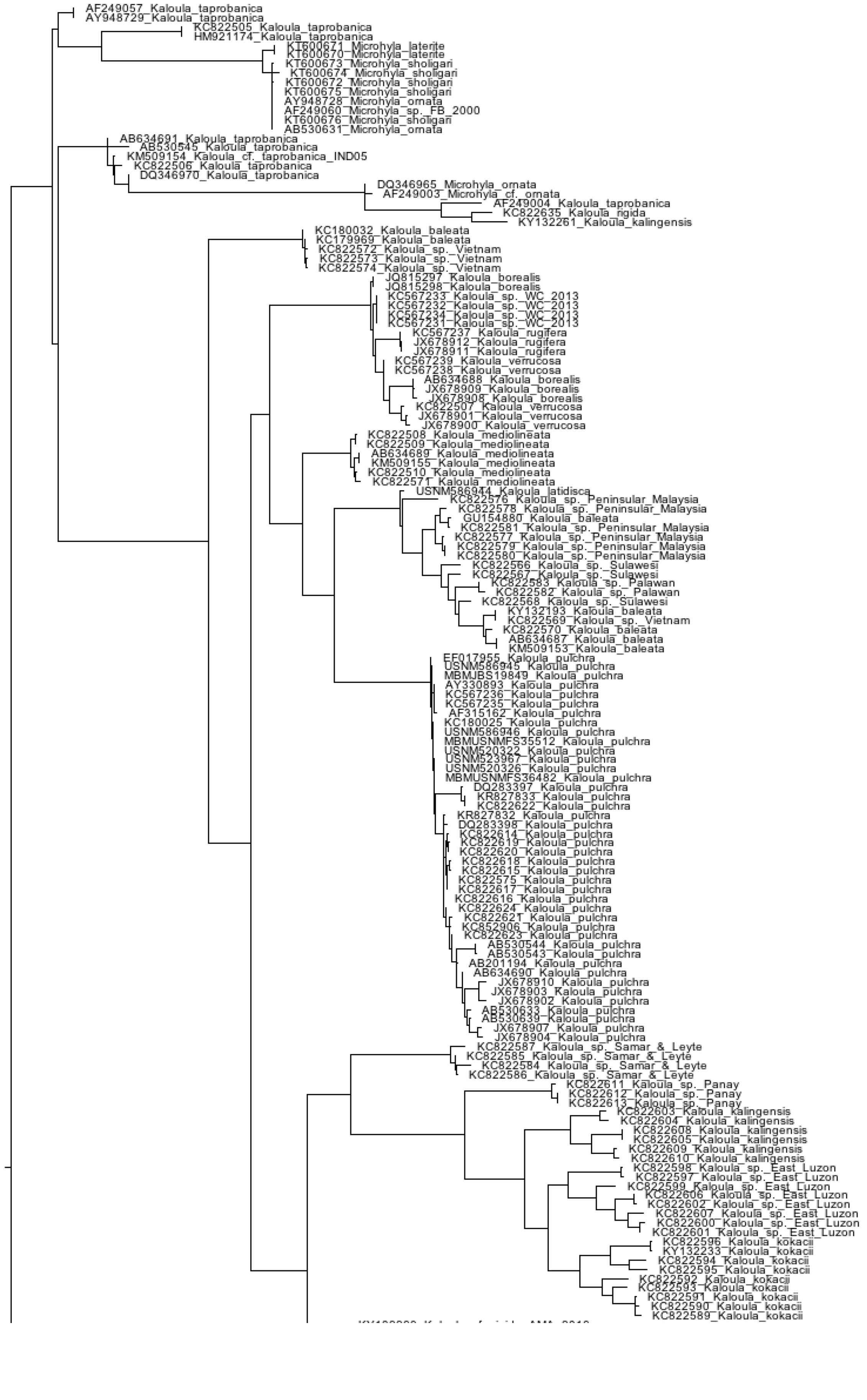
AF249057_Kaloula_taprobanica
AY948729_Kaloula_taprobanica
KC822505_Kaloula_taprobanica
HM921174_Kaloula_taprobanica
KT600671_Microhyla_laterite
KT600670_Microhyla_laterite
KT600673_Microhyla_sholigari
KT600674_Microhyla_sholigari
KT600672_Microhyla_sholigari
KT600675_Microhyla_sholigari
AY948728_Microhyla_ornata
AF249060_Microhyla_sp._FB_2000
KT600676_Microhyla_sholigari
AB530631_Microhyla_ornata
AB634691_Kaloula_taprobanica
AB530545_Kaloula_taprobanica
KM509154_Kaloula_cf._taprobanica_IND05
KC822506_Kaloula_taprobanica
DQ346970_Kaloula_taprobanica
DQ346965_Microhyla_ornata
AF249003_Microhyla_cf._ornata
AF249004_Kaloula_taprobanica
KC822635_Kaloula_rigida
KY132261_Kaloula_kalingensis
KC180032_Kaloula_baleata
KC179969_Kaloula_baleata
KC822572_Kaloula_sp._Vietnam
KC822573_Kaloula_sp._Vietnam
KC822574_Kaloula_sp._Vietnam
JQ815297_Kaloula_borealis
JQ815298_Kaloula_borealis
KC567233_Kaloula_sp._WC_2013
KC567232_Kaloula_sp._WC_2013
KC567234_Kaloula_sp._WC_2013
KC567231_Kaloula_sp._WC_2013
KC567237_Kaloula_rugifera
JX678912_Kaloula_rugifera
JX678911_Kaloula_rugifera
KC567239_Kaloula_verrucosa
KC567238_Kaloula_verrucosa
AB634688_Kaloula_borealis
JX678909_Kaloula_borealis
JX678908_Kaloula_borealis
KC822507_Kaloula_verrucosa
JX678901_Kaloula_verrucosa
JX678900_Kaloula_verrucosa
KC822508_Kaloula_mediolineata
KC822509_Kaloula_mediolineata
AB634689_Kaloula_mediolineata
KM509155_Kaloula_mediolineata
KC822510_Kaloula_mediolineata
KC822571_Kaloula_mediolineata
USNM586944_Kaloula_latidisca
KC822576_Kaloula_sp._Peninsular_Malaysia
KC822578_Kaloula_sp._Peninsular_Malaysia
GU154880_Kaloula_baleata
KC822581_Kaloula_sp._Peninsular_Malaysia
KC822577_Kaloula_sp._Peninsular_Malaysia
KC822579_Kaloula_sp._Peninsular_Malaysia
KC822580_Kaloula_sp._Peninsular_Malaysia
KC822566_Kaloula_sp._Sulawesi
KC822567_Kaloula_sp._Sulawesi
KC822583_Kaloula_sp._Palawan
KC822582_Kaloula_sp._Palawan
KC822568_Kaloula_sp._Sulawesi
KY132193_Kaloula_baleata
KC822569_Kaloula_sp._Vietnam
KC822570_Kaloula_baleata
AB634687_Kaloula_baleata
KM509153_Kaloula_baleata
EF017955_Kaloula_pulchra
USNM586945_Kaloula_pulchra
MBMJBS19849_Kaloula_pulchra
AY330893_Kaloula_pulchra
KC567236_Kaloula_pulchra
KC567235_Kaloula_pulchra
AF315162_Kaloula_pulchra
KC180025_Kaloula_pulchra
USNM586946_Kaloula_pulchra
MBMUSNMFS35512_Kaloula_pulchra
USNM520322_Kaloula_pulchra
USNM523967_Kaloula_pulchra
USNM520326_Kaloula_pulchra
MBMUSNMFS36482_Kaloula_pulchra
DQ283397_Kaloula_pulchra
KR827833_Kaloula_pulchra
KC822622_Kaloula_pulchra
KR827832_Kaloula_pulchra
DQ283398_Kaloula_pulchra
KC822614_Kaloula_pulchra
KC822619_Kaloula_pulchra
KC822620_Kaloula_pulchra
KC822618_Kaloula_pulchra
KC822615_Kaloula_pulchra
KC822575_Kaloula_pulchra
KC822617_Kaloula_pulchra
KC822616_Kaloula_pulchra
KC822624_Kaloula_pulchra
KC822621_Kaloula_pulchra
KC852906_Kaloula_pulchra
KC822623_Kaloula_pulchra
AB530544_Kaloula_pulchra
AB530543_Kaloula_pulchra
AB201194_Kaloula_pulchra
AB634690_Kaloula_pulchra
JX678910_Kaloula_pulchra
JX678903_Kaloula_pulchra
JX678902_Kaloula_pulchra
AB530633_Kaloula_pulchra
AB530639_Kaloula_pulchra
JX678907_Kaloula_pulchra
JX678904_Kaloula_pulchra
KC822587_Kaloula_sp._Samar_&_Leyte
KC822585_Kaloula_sp._Samar_&_Leyte
KC822584_Kaloula_sp._Samar_&_Leyte
KC822586_Kaloula_sp._Samar_&_Leyte
KC822611_Kaloula_sp._Panay
KC822612_Kaloula_sp._Panay
KC822613_Kaloula_sp._Panay
KC822603_Kaloula_kalingensis
KC822604_Kaloula_kalingensis
KC822608_Kaloula_kalingensis
KC822605_Kaloula_kalingensis
KC822609_Kaloula_kalingensis
KC822610_Kaloula_kalingensis
KC822598_Kaloula_sp._East_Luzon
KC822597_Kaloula_sp._East_Luzon
KC822599_Kaloula_sp._East_Luzon
KC822606_Kaloula_sp._East_Luzon
KC822602_Kaloula_sp._East_Luzon
KC822607_Kaloula_sp._East_Luzon
KC822600_Kaloula_sp._East_Luzon
KC822601_Kaloula_sp._East_Luzon
KC822596_Kaloula_kokacii
KY132233_Kaloula_kokacii
KC822594_Kaloula_kokacii
KC822595_Kaloula_kokacii
KC822592_Kaloula_kokacii
KC822593_Kaloula_kokacii
KC822591_Kaloula_kokacii
KC822590_Kaloula_kokacii
KC822589_Kaloula_kokacii

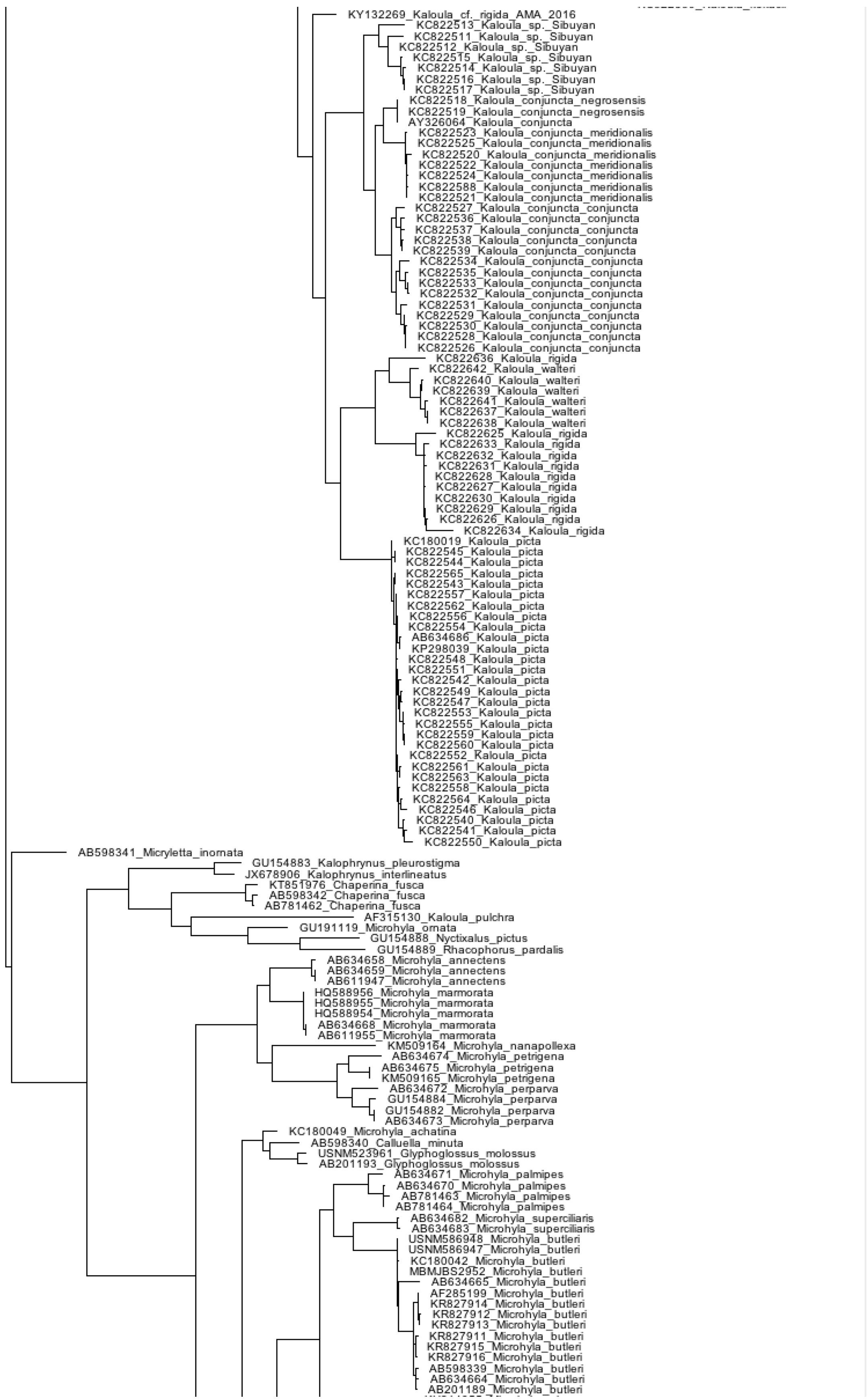
KY132269_Kaloula_cf._rigida_AMA_2016
KC822513_Kaloula_sp._Sibuyan
KC822511_Kaloula_sp._Sibuyan
KC822512_Kaloula_sp._Sibuyan
KC822515_Kaloula_sp._Sibuyan
KC822514_Kaloula_sp._Sibuyan
KC822516_Kaloula_sp._Sibuyan
KC822517_Kaloula_sp._Sibuyan
KC822518_Kaloula_conjuncta_negrosensis
KC822519_Kaloula_conjuncta_negrosensis
AY326064_Kaloula_conjuncta
KC822523_Kaloula_conjuncta_meridionalis
KC822525_Kaloula_conjuncta_meridionalis
KC822520_Kaloula_conjuncta_meridionalis
KC822522_Kaloula_conjuncta_meridionalis
KC822524_Kaloula_conjuncta_meridionalis
KC822588_Kaloula_conjuncta_meridionalis
KC822521_Kaloula_conjuncta_meridionalis
KC822527_Kaloula_conjuncta_conjuncta
KC822536_Kaloula_conjuncta_conjuncta
KC822537_Kaloula_conjuncta_conjuncta
KC822538_Kaloula_conjuncta_conjuncta
KC822539_Kaloula_conjuncta_conjuncta
KC822534_Kaloula_conjuncta_conjuncta
KC822535_Kaloula_conjuncta_conjuncta
KC822533_Kaloula_conjuncta_conjuncta
KC822532_Kaloula_conjuncta_conjuncta
KC822531_Kaloula_conjuncta_conjuncta
KC822529_Kaloula_conjuncta_conjuncta
KC822530_Kaloula_conjuncta_conjuncta
KC822528_Kaloula_conjuncta_conjuncta
KC822526_Kaloula_conjuncta_conjuncta
KC822636_Kaloula_rigida
KC822642_Kaloula_walteri
KC822640_Kaloula_walteri
KC822639_Kaloula_walteri
KC822641_Kaloula_walteri
KC822637_Kaloula_walteri
KC822638_Kaloula_walteri
KC822625_Kaloula_rigida
KC822633_Kaloula_rigida
KC822632_Kaloula_rigida
KC822631_Kaloula_rigida
KC822628_Kaloula_rigida
KC822627_Kaloula_rigida
KC822630_Kaloula_rigida
KC822629_Kaloula_rigida
KC822626_Kaloula_rigida
KC822634_Kaloula_rigida
KC180019_Kaloula_picta
KC822545_Kaloula_picta
KC822544_Kaloula_picta
KC822565_Kaloula_picta
KC822543_Kaloula_picta
KC822557_Kaloula_picta
KC822562_Kaloula_picta
KC822556_Kaloula_picta
KC822554_Kaloula_picta
AB634686_Kaloula_picta
KP298039_Kaloula_picta
KC822548_Kaloula_picta
KC822551_Kaloula_picta
KC822542_Kaloula_picta
KC822549_Kaloula_picta
KC822547_Kaloula_picta
KC822553_Kaloula_picta
KC822555_Kaloula_picta
KC822559_Kaloula_picta
KC822560_Kaloula_picta
KC822552_Kaloula_picta
KC822561_Kaloula_picta
KC822563_Kaloula_picta
KC822558_Kaloula_picta
KC822564_Kaloula_picta
KC822546_Kaloula_picta
KC822540_Kaloula_picta
KC822541_Kaloula_picta
KC822550_Kaloula_picta
AB598341_Micryletta_inornata
GU154883_Kalophrynus_pleurostigma
JX678906_Kalophrynus_interlineatus
KT851976_Chaperina_fusca
AB598342_Chaperina_fusca
AB781462_Chaperina_fusca
AF315130_Kaloula_pulchra
GU191119_Microhyla_ornata
GU154888_Nyctixalus_pictus
GU154889_Rhacophorus_pardalis
AB634658_Microhyla_annectens
AB634659_Microhyla_annectens
AB611947_Microhyla_annectens
HQ588956_Microhyla_marmorata
HQ588955_Microhyla_marmorata
HQ588954_Microhyla_marmorata
AB634668_Microhyla_marmorata
AB611955_Microhyla_marmorata
KM509164_Microhyla_nanapollexa
AB634674_Microhyla_petrigena
AB634675_Microhyla_petrigena
KM509165_Microhyla_petrigena
AB634672_Microhyla_perparva
GU154884_Microhyla_perparva
GU154882_Microhyla_perparva
AB634673_Microhyla_perparva
KC180049_Microhyla_achatina
AB598340_Calluella_minuta
USNM523961_Glyphoglossus_molossus
AB201193_Glyphoglossus_molossus
AB634671_Microhyla_palmipes
AB634670_Microhyla_palmipes
AB781463_Microhyla_palmipes
AB781464_Microhyla_palmipes
AB634682_Microhyla_superciliaris
AB634683_Microhyla_superciliaris
USNM586948_Microhyla_butleri
USNM586947_Microhyla_butleri
KC180042_Microhyla_butleri
MBMJBS2952_Microhyla_butleri
AB634665_Microhyla_butleri
AF285199_Microhyla_butleri
KR827914_Microhyla_butleri
KR827912_Microhyla_butleri
KR827913_Microhyla_butleri
KR827911_Microhyla_butleri
KR827915_Microhyla_butleri
KR827916_Microhyla_butleri
AB598339_Microhyla_butleri
AB634664_Microhyla_butleri
AB201189_Microhyla_butleri

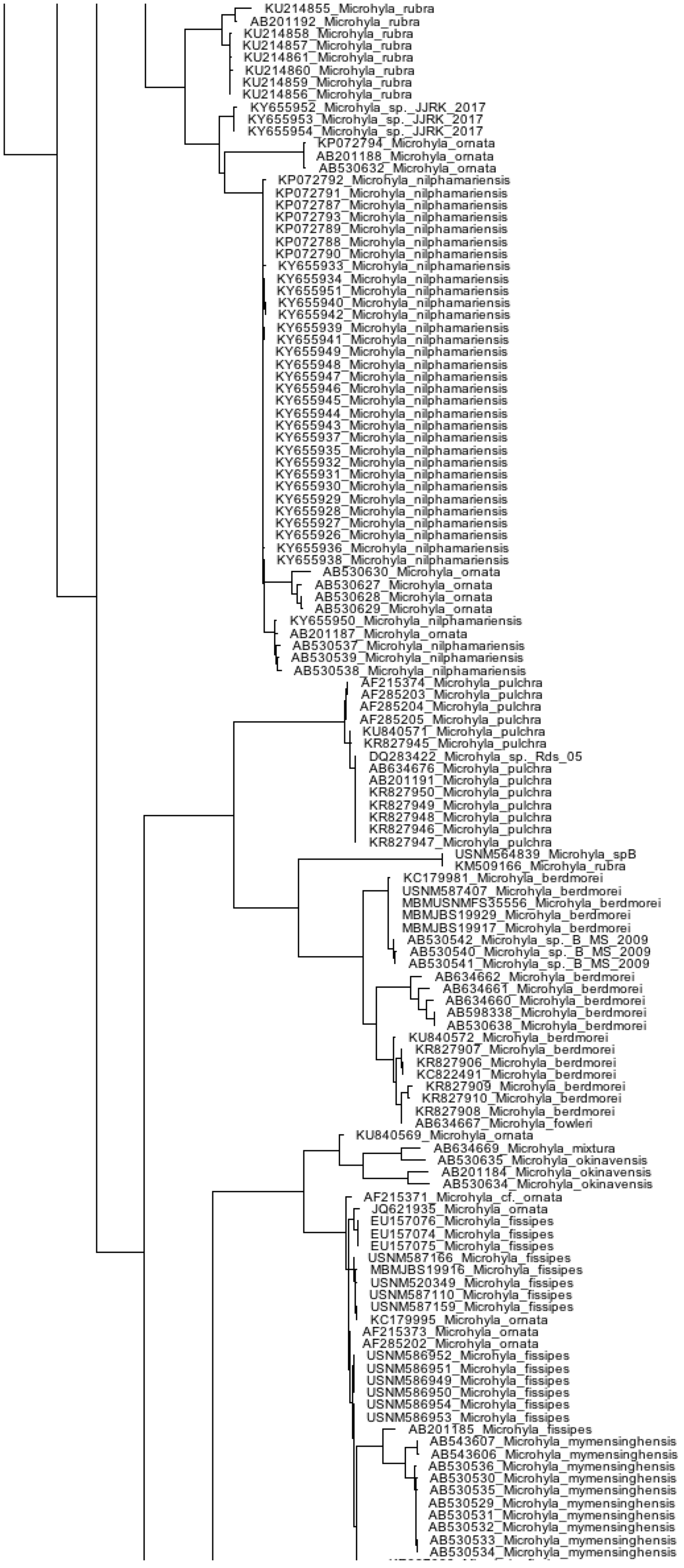
KU214855_Microhyla_rubra
AB201192_Microhyla_rubra
KU214858_Microhyla_rubra
KU214857_Microhyla_rubra
KU214861_Microhyla_rubra
KU214860_Microhyla_rubra
KU214859_Microhyla_rubra
KU214856_Microhyla_rubra
KY655952_Microhyla_sp._JJRK_2017
KY655953_Microhyla_sp._JJRK_2017
KY655954_Microhyla_sp._JJRK_2017
KP072794_Microhyla_ornata
AB201188_Microhyla_ornata
AB530632_Microhyla_ornata
KP072792_Microhyla_nilphamariensis
KP072791_Microhyla_nilphamariensis
KP072787_Microhyla_nilphamariensis
KP072793_Microhyla_nilphamariensis
KP072789_Microhyla_nilphamariensis
KP072788_Microhyla_nilphamariensis
KP072790_Microhyla_nilphamariensis
KY655933_Microhyla_nilphamariensis
KY655934_Microhyla_nilphamariensis
KY655951_Microhyla_nilphamariensis
KY655940_Microhyla_nilphamariensis
KY655942_Microhyla_nilphamariensis
KY655939_Microhyla_nilphamariensis
KY655941_Microhyla_nilphamariensis
KY655949_Microhyla_nilphamariensis
KY655948_Microhyla_nilphamariensis
KY655947_Microhyla_nilphamariensis
KY655946_Microhyla_nilphamariensis
KY655945_Microhyla_nilphamariensis
KY655944_Microhyla_nilphamariensis
KY655943_Microhyla_nilphamariensis
KY655937_Microhyla_nilphamariensis
KY655935_Microhyla_nilphamariensis
KY655932_Microhyla_nilphamariensis
KY655931_Microhyla_nilphamariensis
KY655930_Microhyla_nilphamariensis
KY655929_Microhyla_nilphamariensis
KY655928_Microhyla_nilphamariensis
KY655927_Microhyla_nilphamariensis
KY655926_Microhyla_nilphamariensis
KY655936_Microhyla_nilphamariensis
KY655938_Microhyla_nilphamariensis
AB530630_Microhyla_ornata
AB530627_Microhyla_ornata
AB530628_Microhyla_ornata
AB530629_Microhyla_ornata
KY655950_Microhyla_nilphamariensis
AB201187_Microhyla_ornata
AB530537_Microhyla_nilphamariensis
AB530539_Microhyla_nilphamariensis
AB530538_Microhyla_nilphamariensis
AF215374_Microhyla_pulchra
AF285203_Microhyla_pulchra
AF285204_Microhyla_pulchra
AF285205_Microhyla_pulchra
KU840571_Microhyla_pulchra
KR827945_Microhyla_pulchra
DQ283422_Microhyla_sp._Rds_05
AB634676_Microhyla_pulchra
AB201191_Microhyla_pulchra
KR827950_Microhyla_pulchra
KR827949_Microhyla_pulchra
KR827948_Microhyla_pulchra
KR827946_Microhyla_pulchra
KR827947_Microhyla_pulchra
USNM564839_Microhyla_spB
KM509166_Microhyla_rubra
KC179981_Microhyla_berdmorei
USNM587407_Microhyla_berdmorei
MBMUSNMFS35556_Microhyla_berdmorei
MBMJBS19929_Microhyla_berdmorei
MBMJBS19917_Microhyla_berdmorei
AB530542_Microhyla_sp._B_MS_2009
AB530540_Microhyla_sp._B_MS_2009
AB530541_Microhyla_sp._B_MS_2009
AB634662_Microhyla_berdmorei
AB634661_Microhyla_berdmorei
AB634660_Microhyla_berdmorei
AB598338_Microhyla_berdmorei
AB530638_Microhyla_berdmorei
KU840572_Microhyla_berdmorei
KR827907_Microhyla_berdmorei
KR827906_Microhyla_berdmorei
KC822491_Microhyla_berdmorei
KR827909_Microhyla_berdmorei
KR827910_Microhyla_berdmorei
KR827908_Microhyla_berdmorei
AB634667_Microhyla_fowleri
KU840569_Microhyla_ornata
AB634669_Microhyla_mixtura
AB530635_Microhyla_okinavensis
AB201184_Microhyla_okinavensis
AB530634_Microhyla_okinavensis
AF215371_Microhyla_cf._ornata
JQ621935_Microhyla_ornata
EU157076_Microhyla_fissipes
EU157074_Microhyla_fissipes
EU157075_Microhyla_fissipes
USNM587166_Microhyla_fissipes
MBMJBS19916_Microhyla_fissipes
USNM520349_Microhyla_fissipes
USNM587110_Microhyla_fissipes
USNM587159_Microhyla_fissipes
KC179995_Microhyla_ornata
AF215373_Microhyla_ornata
AF285202_Microhyla_ornata
USNM586952_Microhyla_fissipes
USNM586951_Microhyla_fissipes
USNM586949_Microhyla_fissipes
USNM586950_Microhyla_fissipes
USNM586954_Microhyla_fissipes
USNM586953_Microhyla_fissipes
AB201185_Microhyla_fissipes
AB543607_Microhyla_mymensinghensis
AB543606_Microhyla_mymensinghensis
AB530536_Microhyla_mymensinghensis
AB530530_Microhyla_mymensinghensis
AB530535_Microhyla_mymensinghensis
AB530529_Microhyla_mymensinghensis
AB530531_Microhyla_mymensinghensis
AB530532_Microhyla_mymensinghensis
AB530533_Microhyla_mymensinghensis
AB530534_Microhyla_mymensinghensis

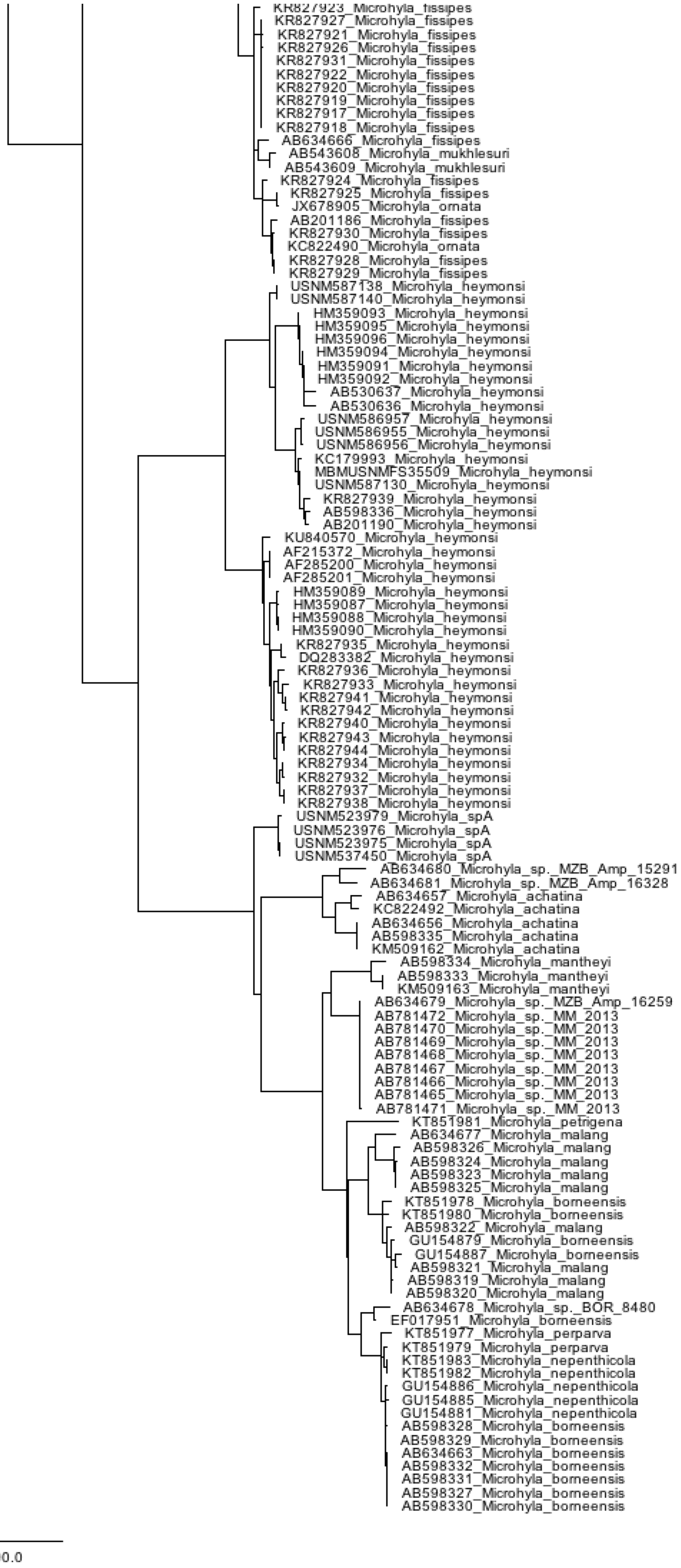

Fig. 4. Microhylidae 16S neighbor-joining tree.

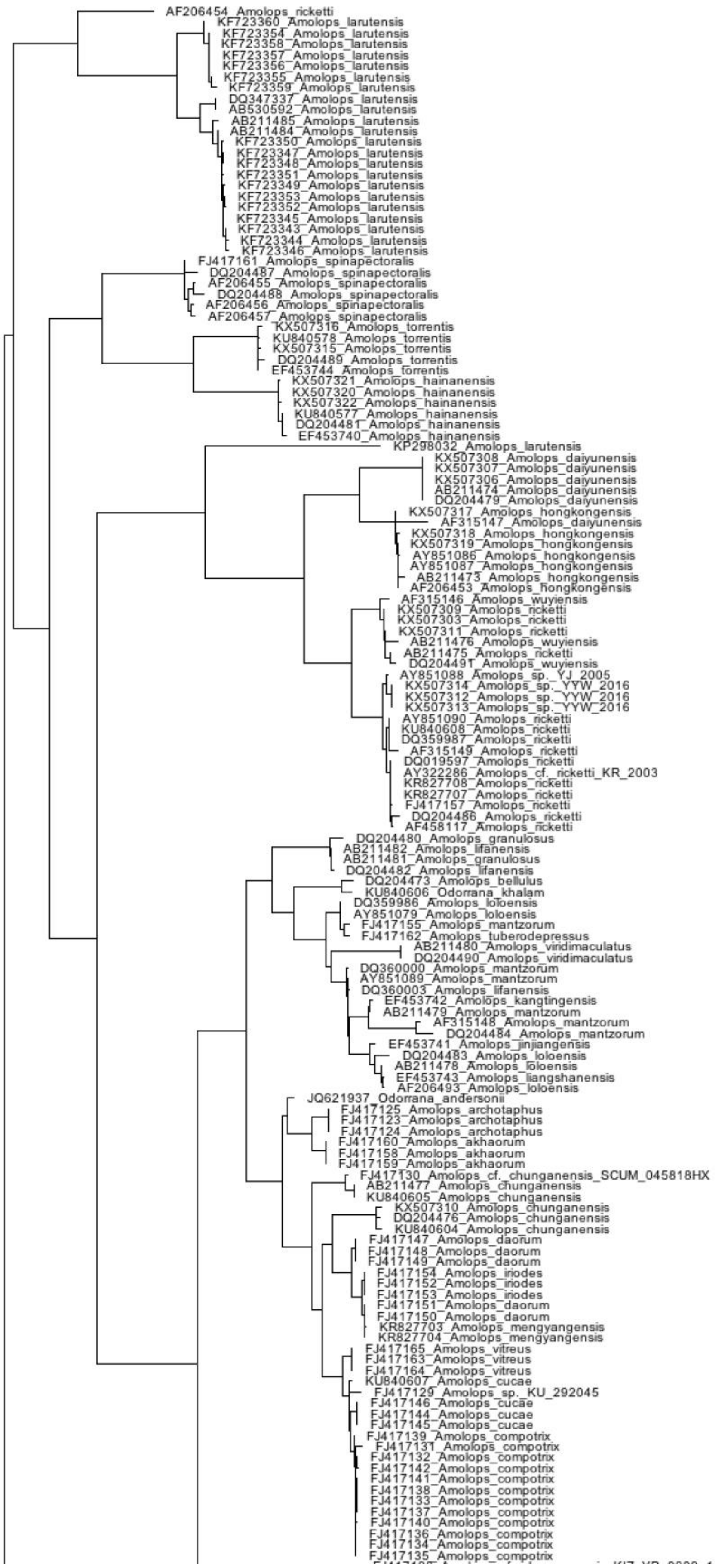
AF206454_Amolops_ricketti
KF723360_Amolops_larutensis
KF723354_Amolops_larutensis
KF723358_Amolops_larutensis
KF723357_Amolops_larutensis
KF723356_Amolops_larutensis
KF723355_Amolops_larutensis
KF723359_Amolops_larutensis
DQ347337_Amolops_larutensis
AB530592_Amolops_larutensis
AB211485_Amolops_larutensis
AB211484_Amolops_larutensis
KF723350_Amolops_larutensis
KF723347_Amolops_larutensis
KF723348_Amolops_larutensis
KF723351_Amolops_larutensis
KF723349_Amolops_larutensis
KF723353_Amolops_larutensis
KF723352_Amolops_larutensis
KF723345_Amolops_larutensis
KF723343_Amolops_larutensis
KF723344_Amolops_larutensis
KF723346_Amolops_larutensis
FJ417161_Amolops_spinapectoralis
DQ204487_Amolops_spinapectoralis
AF206455_Amolops_spinapectoralis
DQ204488_Amolops_spinapectoralis
AF206456_Amolops_spinapectoralis
AF206457_Amolops_spinapectoralis
KX507316_Amolops_torrentis
KU840578_Amolops_torrentis
KX507315_Amolops_torrentis
DQ204489_Amolops_torrentis
EF453744_Amolops_torrentis
KX507321_Amolops_hainanensis
KX507320_Amolops_hainanensis
KX507322_Amolops_hainanensis
KU840577_Amolops_hainanensis
DQ204481_Amolops_hainanensis
EF453740_Amolops_hainanensis
KP298032_Amolops_larutensis
KX507308_Amolops_daiyunensis
KX507307_Amolops_daiyunensis
KX507306_Amolops_daiyunensis
AB211474_Amolops_daiyunensis
DQ204479_Amolops_daiyunensis
KX507317_Amolops_hongkongensis
AF315147_Amolops_daiyunensis
KX507318_Amolops_hongkongensis
KX507319_Amolops_hongkongensis
AY851086_Amolops_hongkongensis
AY851087_Amolops_hongkongensis
AB211473_Amolops_hongkongensis
AF206453_Amolops_hongkongensis
AF315146_Amolops_wuyiensis
KX507309_Amolops_ricketti
KX507303_Amolops_ricketti
KX507311_Amolops_ricketti
AB211476_Amolops_wuyiensis
AB211475_Amolops_ricketti
DQ204491_Amolops_wuyiensis
AY851088_Amolops_sp._YJ_2005
KX507314_Amolops_sp._YYW_2016
KX507312_Amolops_sp._YYW_2016
KX507313_Amolops_sp._YYW_2016
AY851090_Amolops_ricketti
KU840608_Amolops_ricketti
DQ359987_Amolops_ricketti
AF315149_Amolops_ricketti
DQ019597_Amolops_ricketti
AY322286_Amolops_cf._ricketti_KR_2003
KR827708_Amolops_ricketti
KR827707_Amolops_ricketti
FJ417157_Amolops_ricketti
DQ204486_Amolops_ricketti
AF458117_Amolops_ricketti
DQ204480_Amolops_granulosus
AB211482_Amolops_lifanensis
AB211481_Amolops_granulosus
DQ204482_Amolops_lifanensis
DQ204473_Amolops_bellulus
KU840606_Odorrana_khalam
DQ359986_Amolops_loloensis
AY851079_Amolops_loloensis
FJ417155_Amolops_mantzorum
FJ417162_Amolops_tuberodepressus
AB211480_Amolops_viridimaculatus
DQ204490_Amolops_viridimaculatus
DQ360000_Amolops_mantzorum
AY851089_Amolops_mantzorum
DQ360003_Amolops_lifanensis
EF453742_Amolops_kangtingensis
AB211479_Amolops_mantzorum
AF315148_Amolops_mantzorum
DQ204484_Amolops_mantzorum
EF453741_Amolops_jinjiangensis
DQ204483_Amolops_loloensis
AB211478_Amolops_loloensis
EF453743_Amolops_liangshanensis
AF206493_Amolops_loloensis
JQ621937_Odorrana_andersonii
FJ417125_Amolops_archotaphus
FJ417123_Amolops_archotaphus
FJ417124_Amolops_archotaphus
FJ417160_Amolops_akhaorum
FJ417158_Amolops_akhaorum
FJ417159_Amolops_akhaorum
FJ417130_Amolops_cf._chunganensis_SCUM_045818HX
AB211477_Amolops_chunganensis
KU840605_Amolops_chunganensis
KX507310_Amolops_chunganensis
DQ204476_Amolops_chunganensis
KU840604_Amolops_chunganensis
FJ417147_Amolops_daorum
FJ417148_Amolops_daorum
FJ417149_Amolops_daorum
FJ417154_Amolops_iriodes
FJ417152_Amolops_iriodes
FJ417153_Amolops_iriodes
FJ417151_Amolops_daorum
FJ417150_Amolops_daorum
KR827703_Amolops_mengyangensis
KR827704_Amolops_mengyangensis
FJ417165_Amolops_vitreus
FJ417163_Amolops_vitreus
FJ417164_Amolops_vitreus
KU840607_Amolops_cucae
FJ417129_Amolops_sp._KU_292045
FJ417146_Amolops_cucae
FJ417144_Amolops_cucae
FJ417145_Amolops_cucae
FJ417139_Amolops_compotrix
FJ417131_Amolops_compotrix
FJ417132_Amolops_compotrix
FJ417142_Amolops_compotrix
FJ417141_Amolops_compotrix
FJ417138_Amolops_compotrix
FJ417133_Amolops_compotrix
FJ417137_Amolops_compotrix
FJ417140_Amolops_compotrix
FJ417136_Amolops_compotrix
FJ417134_Amolops_compotrix
FJ417135_Amolops_compotrix

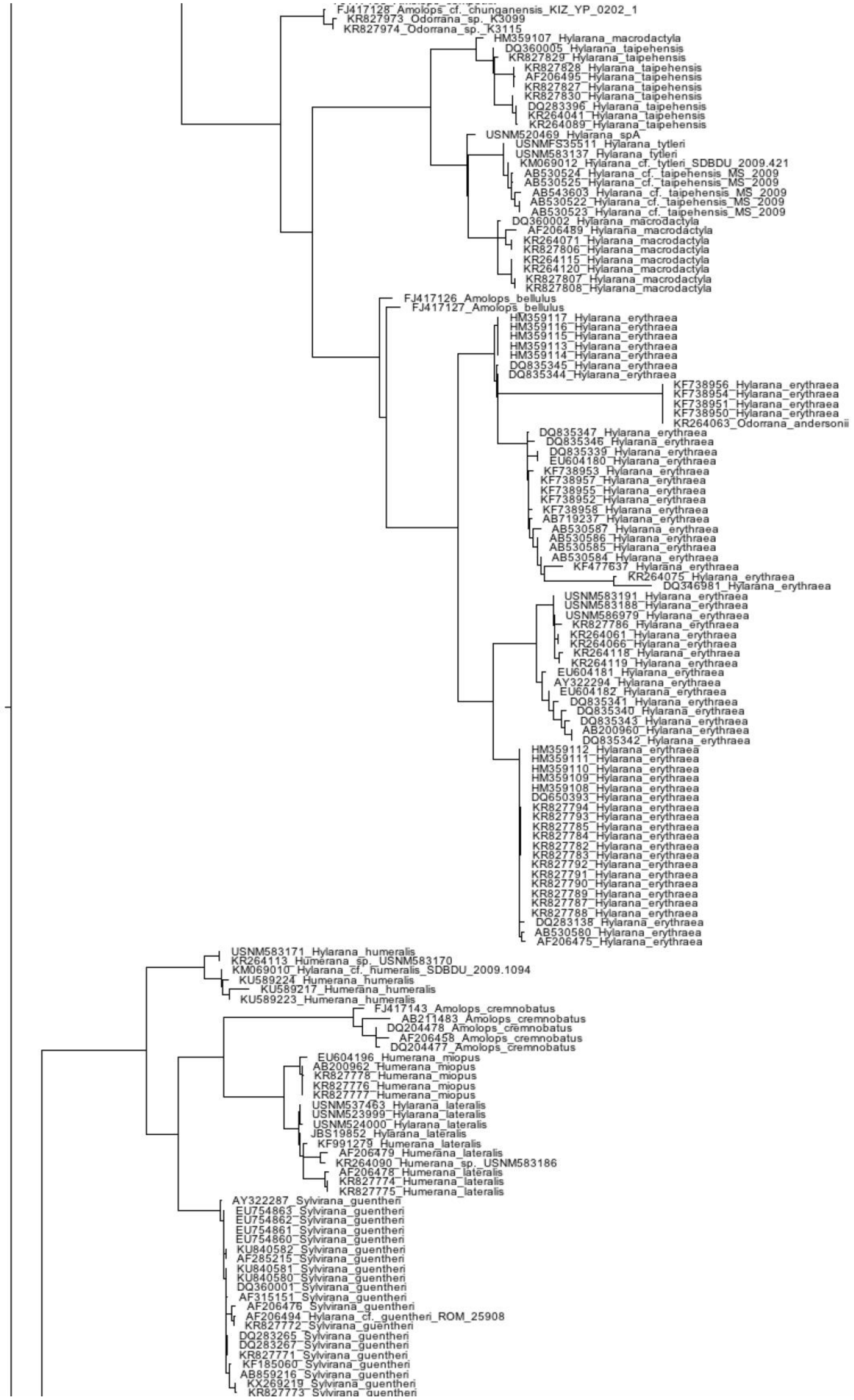
FJ417128_Amolops_cf._chunganensis_KIZ_YP_0202_1
KR827973_Odorrana_sp._K3099
KR827974_Odorrana_sp._K3115
HM359107_Hylarana_macrodactyla
DQ360005_Hylarana_taipehensis
KR827829_Hylarana_taipehensis
KR827828_Hylarana_taipehensis
AF206495_Hylarana_taipehensis
KR827827_Hylarana_taipehensis
KR827830_Hylarana_taipehensis
DQ283396_Hylarana_taipehensis
KR264041_Hylarana_taipehensis
KR264089_Hylarana_taipehensis
USNM520469_Hylarana_spA
USNMFS35511_Hylarana_tytleri
USNM583137_Hylarana_tytleri
KM069012_Hylarana_cf._tytleri_SDBDU_2009.421
AB530524_Hylarana_cf._taipehensis_MS_2009
AB530525_Hylarana_cf._taipehensis_MS_2009
AB543603_Hylarana_cf._taipehensis_MS_2009
AB530522_Hylarana_cf._taipehensis_MS_2009
AB530523_Hylarana_cf._taipehensis_MS_2009
DQ360002_Hylarana_macrodactyla
AF206489_Hylarana_macrodactyla
KR264071_Hylarana_macrodactyla
KR827806_Hylarana_macrodactyla
KR264115_Hylarana_macrodactyla
KR264120_Hylarana_macrodactyla
KR827807_Hylarana_macrodactyla
KR827808_Hylarana_macrodactyla
FJ417126_Amolops_bellulus
FJ417127_Amolops_bellulus
HM359117_Hylarana_erythraea
HM359116_Hylarana_erythraea
HM359115_Hylarana_erythraea
HM359113_Hylarana_erythraea
HM359114_Hylarana_erythraea
DQ835345_Hylarana_erythraea
DQ835344_Hylarana_erythraea
KF738956_Hylarana_erythraea
KF738954_Hylarana_erythraea
KF738951_Hylarana_erythraea
KF738950_Hylarana_erythraea
KR264063_Odorrana_andersonii
DQ835347_Hylarana_erythraea
DQ835346_Hylarana_erythraea
DQ835339_Hylarana_erythraea
EU604180_Hylarana_erythraea
KF738953_Hylarana_erythraea
KF738957_Hylarana_erythraea
KF738955_Hylarana_erythraea
KF738952_Hylarana_erythraea
KF738958_Hylarana_erythraea
AB719237_Hylarana_erythraea
AB530587_Hylarana_erythraea
AB530586_Hylarana_erythraea
AB530585_Hylarana_erythraea
AB530584_Hylarana_erythraea
KF477637_Hylarana_erythraea
KR264075_Hylarana_erythraea
DQ346981_Hylarana_erythraea
USNM583191_Hylarana_erythraea
USNM583188_Hylarana_erythraea
USNM586979_Hylarana_erythraea
KR827786_Hylarana_erythraea
KR264061_Hylarana_erythraea
KR264066_Hylarana_erythraea
KR264118_Hylarana_erythraea
KR264119_Hylarana_erythraea
EU604181_Hylarana_erythraea
AY322294_Hylarana_erythraea
EU604182_Hylarana_erythraea
DQ835341_Hylarana_erythraea
DQ835340_Hylarana_erythraea
DQ835343_Hylarana_erythraea
AB200960_Hylarana_erythraea
DQ835342_Hylarana_erythraea
HM359112_Hylarana_erythraea
HM359111_Hylarana_erythraea
HM359110_Hylarana_erythraea
HM359109_Hylarana_erythraea
HM359108_Hylarana_erythraea
DQ650393_Hylarana_erythraea
KR827794_Hylarana_erythraea
KR827793_Hylarana_erythraea
KR827785_Hylarana_erythraea
KR827784_Hylarana_erythraea
KR827782_Hylarana_erythraea
KR827783_Hylarana_erythraea
KR827792_Hylarana_erythraea
KR827791_Hylarana_erythraea
KR827790_Hylarana_erythraea
KR827789_Hylarana_erythraea
KR827787_Hylarana_erythraea
KR827788_Hylarana_erythraea
DQ283138_Hylarana_erythraea
AB530580_Hylarana_erythraea
AF206475_Hylarana_erythraea
USNM583171_Hylarana_humeralis
KR264113_Humerana_sp._USNM583170
KM069010_Hylarana_cf._humeralis_SDBDU_2009.1094
KU589224_Humerana_humeralis
KU589217_Humerana_humeralis
KU589223_Humerana_humeralis
FJ417143_Amolops_cremnobatus
AB211483_Amolops_cremnobatus
DQ204478_Amolops_cremnobatus
AF206458_Amolops_cremnobatus
DQ204477_Amolops_cremnobatus
EU604196_Humerana_miopus
AB200962_Humerana_miopus
KR827778_Humerana_miopus
KR827776_Humerana_miopus
KR827777_Humerana_miopus
USNM537463_Hylarana_lateralis
USNM523999_Hylarana_lateralis
USNM524000_Hylarana_lateralis
JBS19852_Hylarana_lateralis
KF991279_Humerana_lateralis
AF206479_Humerana_lateralis
KR264090_Humerana_sp._USNM583186
AF206478_Humerana_lateralis
KR827774_Humerana_lateralis
KR827775_Humerana_lateralis
AY322287_Sylvirana_guentheri
EU754863_Sylvirana_guentheri
EU754862_Sylvirana_guentheri
EU754861_Sylvirana_guentheri
EU754860_Sylvirana_guentheri
KU840582_Sylvirana_guentheri
AF285215_Sylvirana_guentheri
KU840581_Sylvirana_guentheri
KU840580_Sylvirana_guentheri
DQ360001_Sylvirana_guentheri
AF315151_Sylvirana_guentheri
AF206476_Sylvirana_guentheri
AF206494_Hylarana_cf._guentheri_ROM_25908
KR827772_Sylvirana_guentheri
DQ283265_Sylvirana_guentheri
DQ283267_Sylvirana_guentheri
KR827771_Sylvirana_guentheri
KF185060_Sylvirana_guentheri
AB859216_Sylvirana_guentheri
KX269219_Sylvirana_guentheri
KR827773_Sylvirana_guentheri

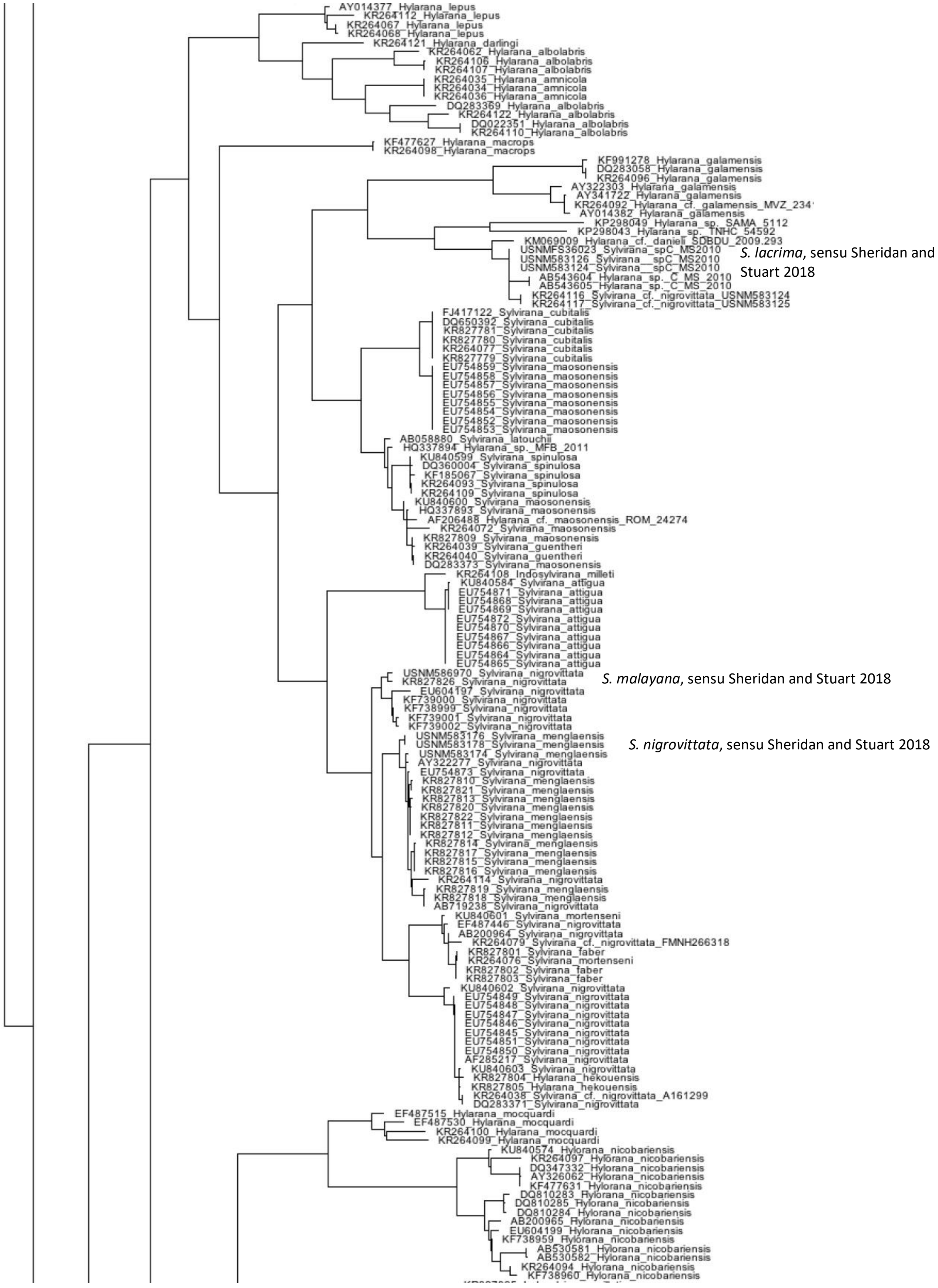
AY014377_Hylarana_lepus
KR264112_Hylarana_lepus
KR264067_Hylarana_lepus
KR264068_Hylarana_lepus
KR264121_Hylarana_darlingi
KR264062_Hylarana_albolabris
KR264106_Hylarana_albolabris
KR264107_Hylarana_albolabris
KR264035_Hylarana_amnicola
KR264034_Hylarana_amnicola
KR264036_Hylarana_amnicola
DQ283369_Hylarana_albolabris
KR264122_Hylarana_albolabris
DQ022351_Hylarana_albolabris
KR264110_Hylarana_albolabris
KF477627_Hylarana_macrops
KR264098_Hylarana_macrops
KF991278_Hylarana_galamensis
DQ283058_Hylarana_galamensis
KR264096_Hylarana_galamensis
AY322303_Hylarana_galamensis
AY341722_Hylarana_galamensis
KR264092_Hylarana_cf._galamensis_MVZ_234
AY014382_Hylarana_galamensis
KP298049_Hylarana_sp._SAMA_5112
KP298043_Hylarana_sp._TNHC_54592
KM069009_Hylarana_cf._danieli_SDBDU_2009.293
USNMFS36023_Sylvirana_spC_MS2010
USNM583126_Sylvirana__spC_MS2010
USNM583124_Sylvirana__spC_MS2010
AB543604_Hylarana_sp._C_MS_2010
AB543605_Hylarana_sp._C_MS_2010
KR264116_Sylvirana_cf._nigrovittata_USNM583124
KR264117_Sylvirana_cf._nigrovittata_USNM583125
S. lacrima, sensu Sheridan and Stuart 2018
FJ417122_Sylvirana_cubitalis
DQ650392_Sylvirana_cubitalis
KR827781_Sylvirana_cubitalis
KR827780_Sylvirana_cubitalis
KR264077_Sylvirana_cubitalis
KR827779_Sylvirana_cubitalis
EU754859_Sylvirana_maosonensis
EU754858_Sylvirana_maosonensis
EU754857_Sylvirana_maosonensis
EU754856_Sylvirana_maosonensis
EU754855_Sylvirana_maosonensis
EU754854_Sylvirana_maosonensis
EU754852_Sylvirana_maosonensis
EU754853_Sylvirana_maosonensis
AB058880_Sylvirana_latouchii
HQ337894_Hylarana_sp._MFB_2011
KU840599_Sylvirana_spinulosa
DQ360004_Sylvirana_spinulosa
KF185067_Sylvirana_spinulosa
KR264093_Sylvirana_spinulosa
KR264109_Sylvirana_spinulosa
KU840600_Sylvirana_maosonensis
HQ337893_Sylvirana_maosonensis
AF206488_Hylarana_cf._maosonensis_ROM_24274
KR264072_Sylvirana_maosonensis
KR827809_Sylvirana_maosonensis
KR264039_Sylvirana_guentheri
KR264040_Sylvirana_guentheri
DQ283373_Sylvirana_maosonensis
KR264108_Indosylvirana_milleti
KU840584_Sylvirana_attigua
EU754871_Sylvirana_attigua
EU754868_Sylvirana_attigua
EU754869_Sylvirana_attigua
EU754872_Sylvirana_attigua
EU754870_Sylvirana_attigua
EU754867_Sylvirana_attigua
EU754866_Sylvirana_attigua
EU754864_Sylvirana_attigua
EU754865_Sylvirana_attigua
USNM586970_Sylvirana_nigrovittata
KR827826_Sylvirana_nigrovittata
EU604197_Sylvirana_nigrovittata
KF739000_Sylvirana_nigrovittata
KF738999_Sylvirana_nigrovittata
KF739001_Sylvirana_nigrovittata
KF739002_Sylvirana_nigrovittata
S. malayana, sensu Sheridan and Stuart 2018
USNM583176_Sylvirana_menglaensis
USNM583178_Sylvirana_menglaensis
USNM583174_Sylvirana_menglaensis
AY322277_Sylvirana_nigrovittata
EU754873_Sylvirana_nigrovittata
KR827810_Sylvirana_menglaensis
KR827821_Sylvirana_menglaensis
KR827813_Sylvirana_menglaensis
KR827820_Sylvirana_menglaensis
KR827822_Sylvirana_menglaensis
KR827811_Sylvirana_menglaensis
KR827812_Sylvirana_menglaensis
KR827814_Sylvirana_menglaensis
KR827817_Sylvirana_menglaensis
KR827815_Sylvirana_menglaensis
KR827816_Sylvirana_menglaensis
KR264114_Sylvirana_nigrovittata
KR827819_Sylvirana_menglaensis
KR827818_Sylvirana_menglaensis
AB719238_Sylvirana_nigrovittata
S. nigrovittata, sensu Sheridan and Stuart 2018
KU840601_Sylvirana_mortenseni
EF487446_Sylvirana_nigrovittata
AB200964_Sylvirana_nigrovittata
KR264079_Sylvirana_cf._nigrovittata_FMNH266318
KR827801_Sylvirana_faber
KR264076_Sylvirana_mortenseni
KR827802_Sylvirana_faber
KR827803_Sylvirana_faber
KU840602_Sylvirana_nigrovittata
EU754849_Sylvirana_nigrovittata
EU754848_Sylvirana_nigrovittata
EU754847_Sylvirana_nigrovittata
EU754846_Sylvirana_nigrovittata
EU754845_Sylvirana_nigrovittata
EU754851_Sylvirana_nigrovittata
EU754850_Sylvirana_nigrovittata
AF285217_Sylvirana_nigrovittata
KU840603_Sylvirana_nigrovittata
KR827804_Hylarana_hekouensis
KR827805_Hylarana_hekouensis
KR264038_Sylvirana_cf._nigrovittata_A161299
DQ283371_Sylvirana_nigrovittata
EF487515_Hylarana_mocquardi
EF487530_Hylarana_mocquardi
KR264100_Hylarana_mocquardi
KR264099_Hylarana_mocquardi
KU840574_Hylorana_nicobariensis
KR264097_Hylorana_nicobariensis
DQ347332_Hylorana_nicobariensis
AY326062_Hylorana_nicobariensis
KF477631_Hylorana_nicobariensis
DQ810283_Hylorana_nicobariensis
DQ810285_Hylorana_nicobariensis
DQ810284_Hylorana_nicobariensis
AB200965_Hylorana_nicobariensis
EU604199_Hylorana_nicobariensis
KF738959_Hylorana_nicobariensis
AB530581_Hylorana_nicobariensis
AB530582_Hylorana_nicobariensis
KR264094_Hylorana_nicobariensis
KF738960_Hylorana_nicobariensis

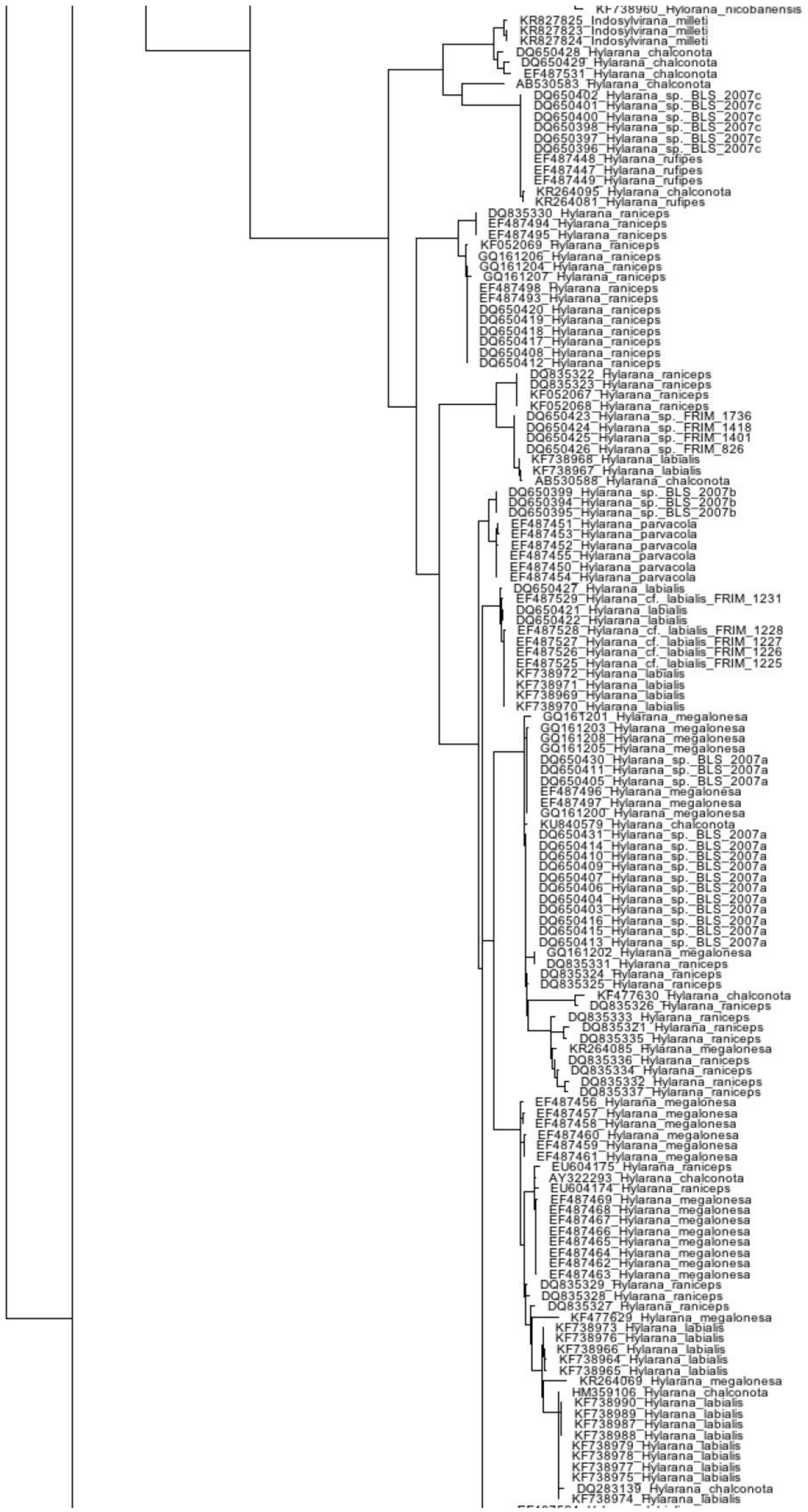
KF738960_Hylorana_nicobariensis
KR827825_Indosylvirana_milleti
KR827823_Indosylvirana_milleti
KR827824_Indosylvirana_milleti
DQ650428_Hylarana_chalconota
DQ650429_Hylarana_chalconota
EF487531_Hylarana_chalconota
AB530583_Hylarana_chalconota
DQ650402_Hylarana_sp._BLS_2007c
DQ650401_Hylarana_sp._BLS_2007c
DQ650400_Hylarana_sp._BLS_2007c
DQ650398_Hylarana_sp._BLS_2007c
DQ650397_Hylarana_sp._BLS_2007c
DQ650396_Hylarana_sp._BLS_2007c
EF487448_Hylarana_rufipes
EF487447_Hylarana_rufipes
EF487449_Hylarana_rufipes
KR264095_Hylarana_chalconota
KR264081_Hylarana_rufipes
DQ835330_Hylarana_raniceps
EF487494_Hylarana_raniceps
EF487495_Hylarana_raniceps
KF052069_Hylarana_raniceps
GQ161206_Hylarana_raniceps
GQ161204_Hylarana_raniceps
GQ161207_Hylarana_raniceps
EF487498_Hylarana_raniceps
EF487493_Hylarana_raniceps
DQ650420_Hylarana_raniceps
DQ650419_Hylarana_raniceps
DQ650418_Hylarana_raniceps
DQ650417_Hylarana_raniceps
DQ650408_Hylarana_raniceps
DQ650412_Hylarana_raniceps
DQ835322_Hylarana_raniceps
DQ835323_Hylarana_raniceps
KF052067_Hylarana_raniceps
KF052068_Hylarana_raniceps
DQ650423_Hylarana_sp._FRIM_1736
DQ650424_Hylarana_sp._FRIM_1418
DQ650425_Hylarana_sp._FRIM_1401
DQ650426_Hylarana_sp._FRIM_826
KF738968_Hylarana_labialis
KF738967_Hylarana_labialis
AB530588_Hylarana_chalconota
DQ650399_Hylarana_sp._BLS_2007b
DQ650394_Hylarana_sp._BLS_2007b
DQ650395_Hylarana_sp._BLS_2007b
EF487451_Hylarana_parvacola
EF487453_Hylarana_parvacola
EF487452_Hylarana_parvacola
EF487455_Hylarana_parvacola
EF487450_Hylarana_parvacola
EF487454_Hylarana_parvacola
DQ650427_Hylarana_labialis
EF487529_Hylarana_cf._labialis_FRIM_1231
DQ650421_Hylarana_labialis
DQ650422_Hylarana_labialis
EF487528_Hylarana_cf._labialis_FRIM_1228
EF487527_Hylarana_cf._labialis_FRIM_1227
EF487526_Hylarana_cf._labialis_FRIM_1226
EF487525_Hylarana_cf._labialis_FRIM_1225
KF738972_Hylarana_labialis
KF738971_Hylarana_labialis
KF738969_Hylarana_labialis
KF738970_Hylarana_labialis
GQ161201_Hylarana_megalonesa
GQ161203_Hylarana_megalonesa
GQ161208_Hylarana_megalonesa
GQ161205_Hylarana_megalonesa
DQ650430_Hylarana_sp._BLS_2007a
DQ650411_Hylarana_sp._BLS_2007a
DQ650405_Hylarana_sp._BLS_2007a
EF487496_Hylarana_megalonesa
EF487497_Hylarana_megalonesa
GQ161200_Hylarana_megalonesa
KU840579_Hylarana_chalconota
DQ650431_Hylarana_sp._BLS_2007a
DQ650414_Hylarana_sp._BLS_2007a
DQ650410_Hylarana_sp._BLS_2007a
DQ650409_Hylarana_sp._BLS_2007a
DQ650407_Hylarana_sp._BLS_2007a
DQ650406_Hylarana_sp._BLS_2007a
DQ650404_Hylarana_sp._BLS_2007a
DQ650403_Hylarana_sp._BLS_2007a
DQ650416_Hylarana_sp._BLS_2007a
DQ650415_Hylarana_sp._BLS_2007a
DQ650413_Hylarana_sp._BLS_2007a
GQ161202_Hylarana_megalonesa
DQ835331_Hylarana_raniceps
DQ835324_Hylarana_raniceps
DQ835325_Hylarana_raniceps
KF477630_Hylarana_chalconota
DQ835326_Hylarana_raniceps
DQ835333_Hylarana_raniceps
DQ835321_Hylarana_raniceps
DQ835335_Hylarana_raniceps
KR264085_Hylarana_megalonesa
DQ835336_Hylarana_raniceps
DQ835334_Hylarana_raniceps
DQ835332_Hylarana_raniceps
DQ835337_Hylarana_raniceps
EF487456_Hylarana_megalonesa
EF487457_Hylarana_megalonesa
EF487458_Hylarana_megalonesa
EF487460_Hylarana_megalonesa
EF487459_Hylarana_megalonesa
EF487461_Hylarana_megalonesa
EU604175_Hylarana_raniceps
AY322293_Hylarana_chalconota
EU604174_Hylarana_raniceps
EF487469_Hylarana_megalonesa
EF487468_Hylarana_megalonesa
EF487467_Hylarana_megalonesa
EF487466_Hylarana_megalonesa
EF487465_Hylarana_megalonesa
EF487464_Hylarana_megalonesa
EF487462_Hylarana_megalonesa
EF487463_Hylarana_megalonesa
DQ835329_Hylarana_raniceps
DQ835328_Hylarana_raniceps
DQ835327_Hylarana_raniceps
KF477629_Hylarana_megalonesa
KF738973_Hylarana_labialis
KF738976_Hylarana_labialis
KF738966_Hylarana_labialis
KF738964_Hylarana_labialis
KF738965_Hylarana_labialis
KR264069_Hylarana_megalonesa
HM359106_Hylarana_chalconota
KF738990_Hylarana_labialis
KF738989_Hylarana_labialis
KF738987_Hylarana_labialis
KF738988_Hylarana_labialis
KF738979_Hylarana_labialis
KF738978_Hylarana_labialis
KF738977_Hylarana_labialis
KF738975_Hylarana_labialis
DQ283139_Hylarana_chalconota
KF738974_Hylarana_labialis

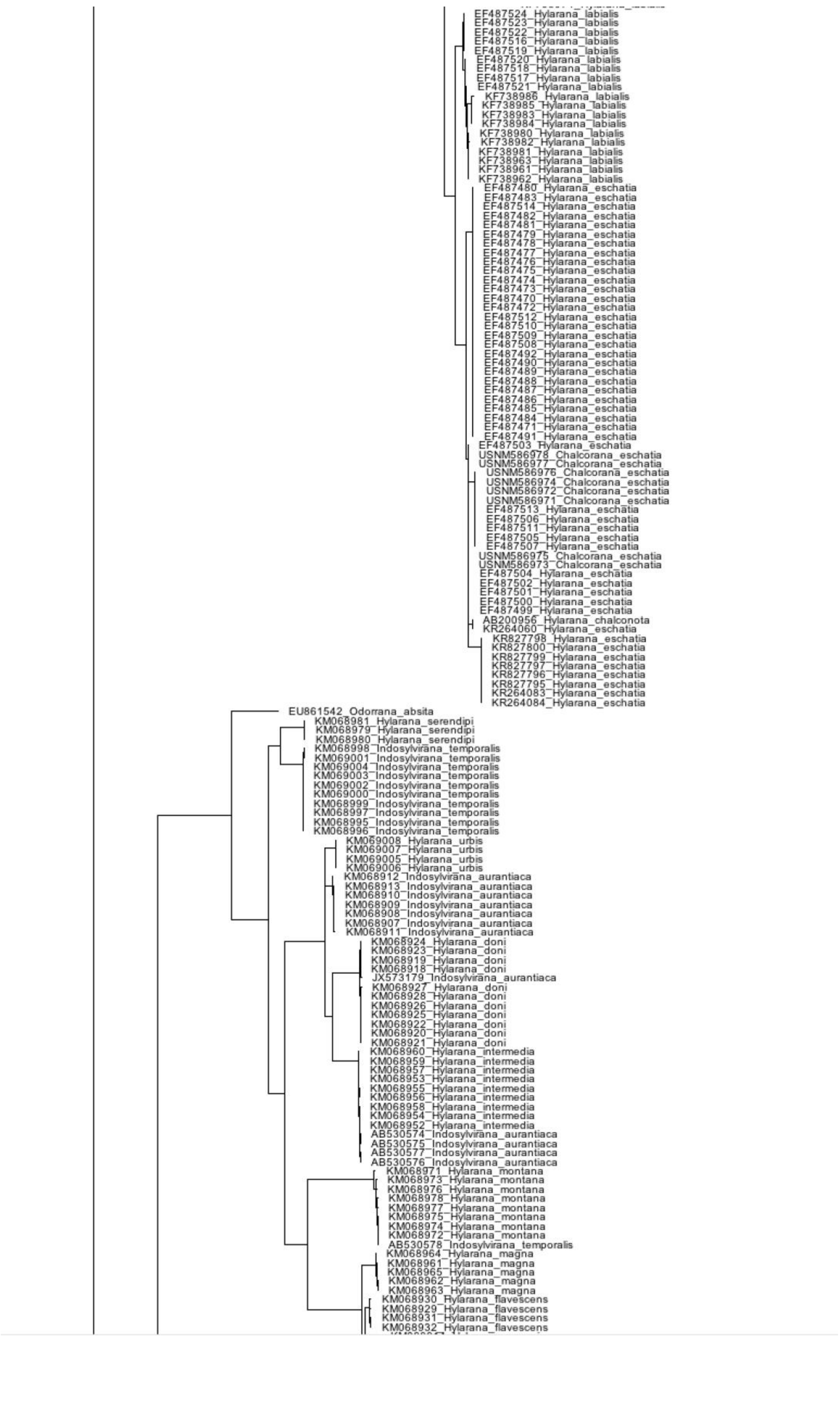
EF487524_Hylarana_labialis
EF487523_Hylarana_labialis
EF487522_Hylarana_labialis
EF487516_Hylarana_labialis
EF487519_Hylarana_labialis
EF487520_Hylarana_labialis
EF487518_Hylarana_labialis
EF487517_Hylarana_labialis
EF487521_Hylarana_labialis
KF738986_Hylarana_labialis
KF738985_Hylarana_labialis
KF738983_Hylarana_labialis
KF738984_Hylarana_labialis
KF738980_Hylarana_labialis
KF738982_Hylarana_labialis
KF738981_Hylarana_labialis
KF738963_Hylarana_labialis
KF738961_Hylarana_labialis
KF738962_Hylarana_labialis
EF487480_Hylarana_eschatia
EF487483_Hylarana_eschatia
EF487514_Hylarana_eschatia
EF487482_Hylarana_eschatia
EF487481_Hylarana_eschatia
EF487479_Hylarana_eschatia
EF487478_Hylarana_eschatia
EF487477_Hylarana_eschatia
EF487476_Hylarana_eschatia
EF487475_Hylarana_eschatia
EF487474_Hylarana_eschatia
EF487473_Hylarana_eschatia
EF487470_Hylarana_eschatia
EF487472_Hylarana_eschatia
EF487512_Hylarana_eschatia
EF487510_Hylarana_eschatia
EF487509_Hylarana_eschatia
EF487508_Hylarana_eschatia
EF487492_Hylarana_eschatia
EF487490_Hylarana_eschatia
EF487489_Hylarana_eschatia
EF487488_Hylarana_eschatia
EF487487_Hylarana_eschatia
EF487486_Hylarana_eschatia
EF487485_Hylarana_eschatia
EF487484_Hylarana_eschatia
EF487471_Hylarana_eschatia
EF487491_Hylarana_eschatia
EF487503_Hylarana_eschatia
USNM586978_Chalcorana_eschatia
USNM586977_Chalcorana_eschatia
USNM586976_Chalcorana_eschatia
USNM586974_Chalcorana_eschatia
USNM586972_Chalcorana_eschatia
USNM586971_Chalcorana_eschatia
EF487513_Hylarana_eschatia
EF487506_Hylarana_eschatia
EF487511_Hylarana_eschatia
EF487505_Hylarana_eschatia
EF487507_Hylarana_eschatia
USNM586975_Chalcorana_eschatia
USNM586973_Chalcorana_eschatia
EF487504_Hylarana_eschatia
EF487502_Hylarana_eschatia
EF487501_Hylarana_eschatia
EF487500_Hylarana_eschatia
EF487499_Hylarana_eschatia
AB200956_Hylarana_chalconota
KR264060_Hylarana_eschatia
KR827798_Hylarana_eschatia
KR827800_Hylarana_eschatia
KR827799_Hylarana_eschatia
KR827797_Hylarana_eschatia
KR827796_Hylarana_eschatia
KR827795_Hylarana_eschatia
KR264083_Hylarana_eschatia
KR264084_Hylarana_eschatia
EU861542_Odorrana_absita
KM068981_Hylarana_serendipi
KM068979_Hylarana_serendipi
KM068980_Hylarana_serendipi
KM068998_Indosylvirana_temporalis
KM069001_Indosylvirana_temporalis
KM069004_Indosylvirana_temporalis
KM069003_Indosylvirana_temporalis
KM069002_Indosylvirana_temporalis
KM069000_Indosylvirana_temporalis
KM068999_Indosylvirana_temporalis
KM068997_Indosylvirana_temporalis
KM068995_Indosylvirana_temporalis
KM068996_Indosylvirana_temporalis
KM069008_Hylarana_urbis
KM069007_Hylarana_urbis
KM069005_Hylarana_urbis
KM069006_Hylarana_urbis
KM068912_Indosylvirana_aurantiaca
KM068913_Indosylvirana_aurantiaca
KM068910_Indosylvirana_aurantiaca
KM068909_Indosylvirana_aurantiaca
KM068908_Indosylvirana_aurantiaca
KM068907_Indosylvirana_aurantiaca
KM068911_Indosylvirana_aurantiaca
KM068924_Hylarana_doni
KM068923_Hylarana_doni
KM068919_Hylarana_doni
KM068918_Hylarana_doni
JX573179_Indosylvirana_aurantiaca
KM068927_Hylarana_doni
KM068928_Hylarana_doni
KM068926_Hylarana_doni
KM068925_Hylarana_doni
KM068922_Hylarana_doni
KM068920_Hylarana_doni
KM068921_Hylarana_doni
KM068960_Hylarana_intermedia
KM068959_Hylarana_intermedia
KM068957_Hylarana_intermedia
KM068953_Hylarana_intermedia
KM068955_Hylarana_intermedia
KM068956_Hylarana_intermedia
KM068958_Hylarana_intermedia
KM068954_Hylarana_intermedia
KM068952_Hylarana_intermedia
AB530574_Indosylvirana_aurantiaca
AB530575_Indosylvirana_aurantiaca
AB530577_Indosylvirana_aurantiaca
AB530576_Indosylvirana_aurantiaca
KM068971_Hylarana_montana
KM068973_Hylarana_montana
KM068976_Hylarana_montana
KM068978_Hylarana_montana
KM068977_Hylarana_montana
KM068975_Hylarana_montana
KM068974_Hylarana_montana
KM068972_Hylarana_montana
AB530578_Indosylvirana_temporalis
KM068964_Hylarana_magna
KM068961_Hylarana_magna
KM068965_Hylarana_magna
KM068962_Hylarana_magna
KM068963_Hylarana_magna
KM068930_Hylarana_flavescens
KM068929_Hylarana_flavescens
KM068931_Hylarana_flavescens
KM068932_Hylarana_flavescens

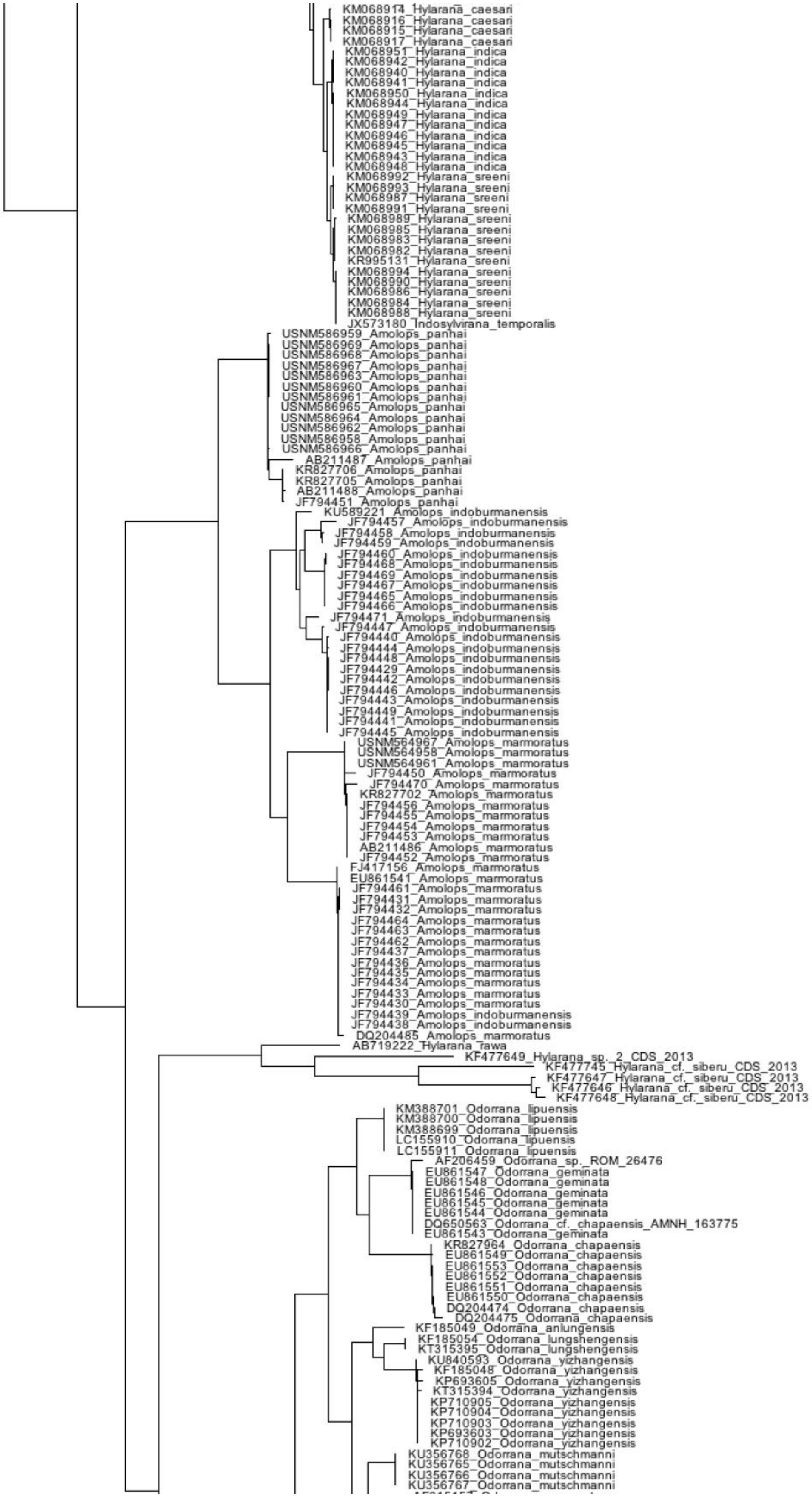
KM068914_Hylarana_caesari
KM068916_Hylarana_caesari
KM068915_Hylarana_caesari
KM068917_Hylarana_caesari
KM068951_Hylarana_indica
KM068942_Hylarana_indica
KM068940_Hylarana_indica
KM068941_Hylarana_indica
KM068950_Hylarana_indica
KM068944_Hylarana_indica
KM068949_Hylarana_indica
KM068947_Hylarana_indica
KM068946_Hylarana_indica
KM068945_Hylarana_indica
KM068943_Hylarana_indica
KM068948_Hylarana_indica
KM068992_Hylarana_sreeni
KM068993_Hylarana_sreeni
KM068987_Hylarana_sreeni
KM068991_Hylarana_sreeni
KM068989_Hylarana_sreeni
KM068985_Hylarana_sreeni
KM068983_Hylarana_sreeni
KM068982_Hylarana_sreeni
KR995131_Hylarana_sreeni
KM068994_Hylarana_sreeni
KM068990_Hylarana_sreeni
KM068986_Hylarana_sreeni
KM068984_Hylarana_sreeni
KM068988_Hylarana_sreeni
JX573180_Indosylvirana_temporalis
USNM586959_Amolops_panhai
USNM586969_Amolops_panhai
USNM586968_Amolops_panhai
USNM586967_Amolops_panhai
USNM586963_Amolops_panhai
USNM586960_Amolops_panhai
USNM586961_Amolops_panhai
USNM586965_Amolops_panhai
USNM586964_Amolops_panhai
USNM586962_Amolops_panhai
USNM586958_Amolops_panhai
USNM586966_Amolops_panhai
AB211487_Amolops_panhai
KR827706_Amolops_panhai
KR827705_Amolops_panhai
AB211488_Amolops_panhai
JF794451_Amolops_panhai
KU589221_Amolops_indoburmanensis
JF794457_Amolops_indoburmanensis
JF794458_Amolops_indoburmanensis
JF794459_Amolops_indoburmanensis
JF794460_Amolops_indoburmanensis
JF794468_Amolops_indoburmanensis
JF794469_Amolops_indoburmanensis
JF794467_Amolops_indoburmanensis
JF794465_Amolops_indoburmanensis
JF794466_Amolops_indoburmanensis
JF794471_Amolops_indoburmanensis
JF794447_Amolops_indoburmanensis
JF794440_Amolops_indoburmanensis
JF794444_Amolops_indoburmanensis
JF794448_Amolops_indoburmanensis
JF794429_Amolops_indoburmanensis
JF794442_Amolops_indoburmanensis
JF794446_Amolops_indoburmanensis
JF794443_Amolops_indoburmanensis
JF794449_Amolops_indoburmanensis
JF794441_Amolops_indoburmanensis
JF794445_Amolops_indoburmanensis
USNM564967_Amolops_marmoratus
USNM564958_Amolops_marmoratus
USNM564961_Amolops_marmoratus
JF794450_Amolops_marmoratus
JF794470_Amolops_marmoratus
KR827702_Amolops_marmoratus
JF794456_Amolops_marmoratus
JF794455_Amolops_marmoratus
JF794454_Amolops_marmoratus
JF794453_Amolops_marmoratus
AB211486_Amolops_marmoratus
JF794452_Amolops_marmoratus
FJ417156_Amolops_marmoratus
EU861541_Amolops_marmoratus
JF794461_Amolops_marmoratus
JF794431_Amolops_marmoratus
JF794432_Amolops_marmoratus
JF794464_Amolops_marmoratus
JF794463_Amolops_marmoratus
JF794462_Amolops_marmoratus
JF794437_Amolops_marmoratus
JF794436_Amolops_marmoratus
JF794435_Amolops_marmoratus
JF794434_Amolops_marmoratus
JF794433_Amolops_marmoratus
JF794430_Amolops_marmoratus
JF794439_Amolops_indoburmanensis
JF794438_Amolops_indoburmanensis
DQ204485_Amolops_marmoratus
AB719222_Hylarana_rawa
KF477649_Hylarana_sp._2_CDS_2013
KF477745_Hylarana_cf._siberu_CDS_2013
KF477647_Hylarana_cf._siberu_CDS_2013
KF477646_Hylarana_cf._siberu_CDS_2013
KF477648_Hylarana_cf._siberu_CDS_2013
KM388701_Odorrana_lipuensis
KM388700_Odorrana_lipuensis
KM388699_Odorrana_lipuensis
LC155910_Odorrana_lipuensis
LC155911_Odorrana_lipuensis
AF206459_Odorrana_sp._ROM_26476
EU861547_Odorrana_geminata
EU861548_Odorrana_geminata
EU861546_Odorrana_geminata
EU861545_Odorrana_geminata
EU861544_Odorrana_geminata
DQ650563_Odorrana_cf._chapaensis_AMNH_163775
EU861543_Odorrana_geminata
KR827964_Odorrana_chapaensis
EU861549_Odorrana_chapaensis
EU861553_Odorrana_chapaensis
EU861552_Odorrana_chapaensis
EU861551_Odorrana_chapaensis
EU861550_Odorrana_chapaensis
DQ204474_Odorrana_chapaensis
DQ204475_Odorrana_chapaensis
KF185049_Odorrana_anlungensis
KF185054_Odorrana_lungshengensis
KT315395_Odorrana_lungshengensis
KU840593_Odorrana_yizhangensis
KF185048_Odorrana_yizhangensis
KP693605_Odorrana_yizhangensis
KT315394_Odorrana_yizhangensis
KP710905_Odorrana_yizhangensis
KP710904_Odorrana_yizhangensis
KP710903_Odorrana_yizhangensis
KP693603_Odorrana_yizhangensis
KP710902_Odorrana_yizhangensis
KU356768_Odorrana_mutschmanni
KU356765_Odorrana_mutschmanni
KU356766_Odorrana_mutschmanni
KU356767_Odorrana_mutschmanni

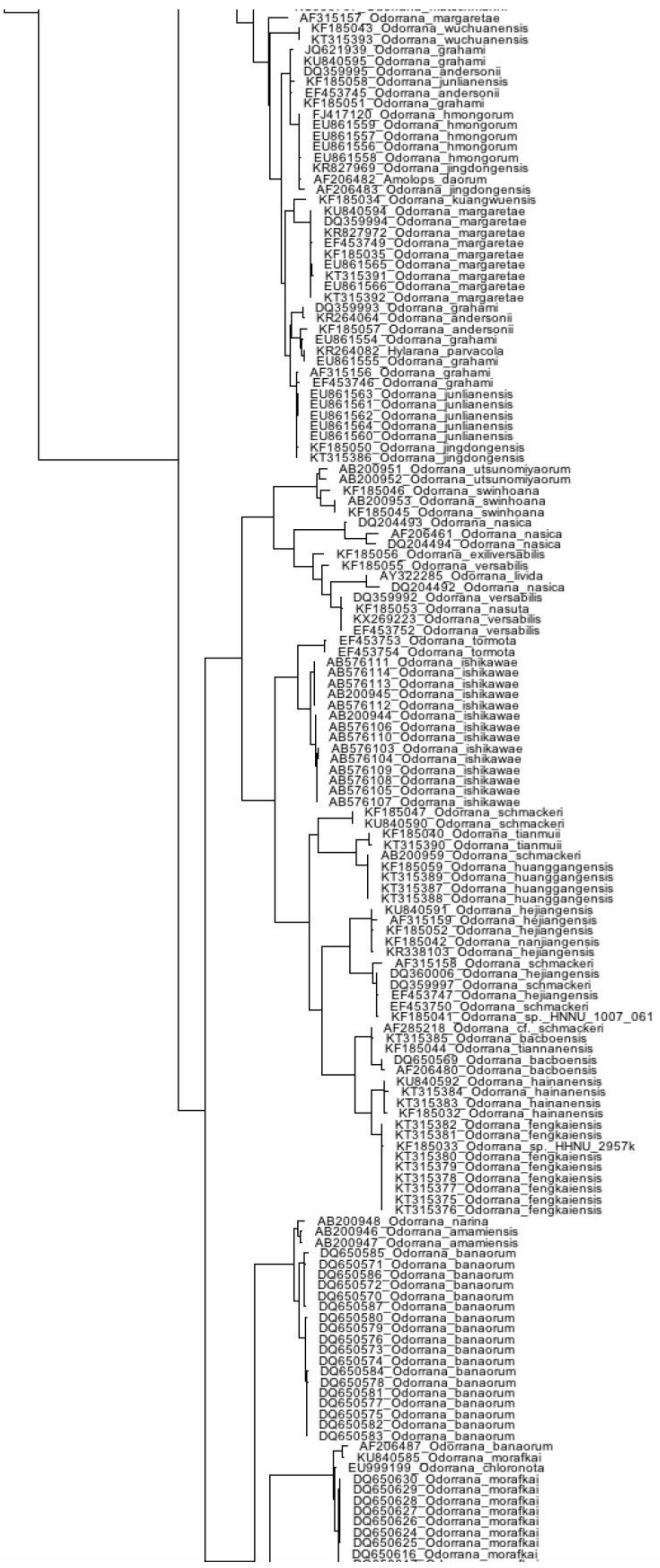
AF315157_Odorrana_margaretae
KF185043_Odorrana_wuchuanensis
KT315393_Odorrana_wuchuanensis
JQ621939_Odorrana_grahami
KU840595_Odorrana_grahami
DQ359995_Odorrana_andersonii
KF185058_Odorrana_junlianensis
EF453745_Odorrana_andersonii
KF185051_Odorrana_grahami
FJ417120_Odorrana_hmongorum
EU861559_Odorrana_hmongorum
EU861557_Odorrana_hmongorum
EU861556_Odorrana_hmongorum
EU861558_Odorrana_hmongorum
KR827969_Odorrana_jingdongensis
AF206482_Amolops_daorum
AF206483_Odorrana_jingdongensis
KF185034_Odorrana_kuangwuensis
KU840594_Odorrana_margaretae
DQ359994_Odorrana_margaretae
KR827972_Odorrana_margaretae
EF453749_Odorrana_margaretae
KF185035_Odorrana_margaretae
EU861565_Odorrana_margaretae
KT315391_Odorrana_margaretae
EU861566_Odorrana_margaretae
KT315392_Odorrana_margaretae
DQ359993_Odorrana_grahami
KR264064_Odorrana_andersonii
KF185057_Odorrana_andersonii
EU861554_Odorrana_grahami
KR264082_Hylarana_parvacola
EU861555_Odorrana_grahami
AF315156_Odorrana_grahami
EF453746_Odorrana_grahami
EU861563_Odorrana_junlianensis
EU861561_Odorrana_junlianensis
EU861562_Odorrana_junlianensis
EU861564_Odorrana_junlianensis
EU861560_Odorrana_junlianensis
KF185050_Odorrana_jingdongensis
KT315386_Odorrana_jingdongensis
AB200951_Odorrana_utsunomiyaorum
AB200952_Odorrana_utsunomiyaorum
KF185046_Odorrana_swinhoana
AB200953_Odorrana_swinhoana
KF185045_Odorrana_swinhoana
DQ204493_Odorrana_nasica
AF206461_Odorrana_nasica
DQ204494_Odorrana_nasica
KF185056_Odorrana_exiliversabilis
KF185055_Odorrana_versabilis
AY322285_Odorrana_livida
DQ204492_Odorrana_nasica
DQ359992_Odorrana_versabilis
KF185053_Odorrana_nasuta
KX269223_Odorrana_versabilis
EF453752_Odorrana_versabilis
EF453753_Odorrana_tormota
EF453754_Odorrana_tormota
AB576111_Odorrana_ishikawae
AB576114_Odorrana_ishikawae
AB576113_Odorrana_ishikawae
AB200945_Odorrana_ishikawae
AB576112_Odorrana_ishikawae
AB200944_Odorrana_ishikawae
AB576106_Odorrana_ishikawae
AB576110_Odorrana_ishikawae
AB576103_Odorrana_ishikawae
AB576104_Odorrana_ishikawae
AB576109_Odorrana_ishikawae
AB576108_Odorrana_ishikawae
AB576105_Odorrana_ishikawae
AB576107_Odorrana_ishikawae
KF185047_Odorrana_schmackeri
KU840590_Odorrana_schmackeri
KF185040_Odorrana_tianmuii
KT315390_Odorrana_tianmuii
AB200959_Odorrana_schmackeri
KF185059_Odorrana_huanggangensis
KT315389_Odorrana_huanggangensis
KT315387_Odorrana_huanggangensis
KT315388_Odorrana_huanggangensis
KU840591_Odorrana_hejiangensis
AF315159_Odorrana_hejiangensis
KF185052_Odorrana_hejiangensis
KF185042_Odorrana_nanjiangensis
KR338103_Odorrana_hejiangensis
AF315158_Odorrana_schmackeri
DQ360006_Odorrana_hejiangensis
DQ359997_Odorrana_schmackeri
EF453747_Odorrana_hejiangensis
EF453750_Odorrana_schmackeri
KF185041_Odorrana_sp._HNNU_1007_061
AF285218_Odorrana_cf._schmackeri
KT315385_Odorrana_bacboensis
KF185044_Odorrana_tiannanensis
DQ650569_Odorrana_bacboensis
AF206480_Odorrana_bacboensis
KU840592_Odorrana_hainanensis
KT315384_Odorrana_hainanensis
KT315383_Odorrana_hainanensis
KF185032_Odorrana_hainanensis
KT315382_Odorrana_fengkaiensis
KT315381_Odorrana_fengkaiensis
KF185033_Odorrana_sp._HHNU_2957k
KT315380_Odorrana_fengkaiensis
KT315379_Odorrana_fengkaiensis
KT315378_Odorrana_fengkaiensis
KT315377_Odorrana_fengkaiensis
KT315375_Odorrana_fengkaiensis
KT315376_Odorrana_fengkaiensis
AB200948_Odorrana_narina
AB200946_Odorrana_amamiensis
AB200947_Odorrana_amamiensis
DQ650585_Odorrana_banaorum
DQ650571_Odorrana_banaorum
DQ650586_Odorrana_banaorum
DQ650572_Odorrana_banaorum
DQ650570_Odorrana_banaorum
DQ650587_Odorrana_banaorum
DQ650580_Odorrana_banaorum
DQ650579_Odorrana_banaorum
DQ650576_Odorrana_banaorum
DQ650573_Odorrana_banaorum
DQ650574_Odorrana_banaorum
DQ650584_Odorrana_banaorum
DQ650578_Odorrana_banaorum
DQ650581_Odorrana_banaorum
DQ650577_Odorrana_banaorum
DQ650575_Odorrana_banaorum
DQ650582_Odorrana_banaorum
DQ650583_Odorrana_banaorum
AF206487_Odorrana_banaorum
KU840585_Odorrana_morafkai
EU999199_Odorrana_chloronota
DQ650630_Odorrana_morafkai
DQ650629_Odorrana_morafkai
DQ650628_Odorrana_morafkai
DQ650627_Odorrana_morafkai
DQ650626_Odorrana_morafkai
DQ650624_Odorrana_morafkai
DQ650625_Odorrana_morafkai
DQ650616_Odorrana_morafkai

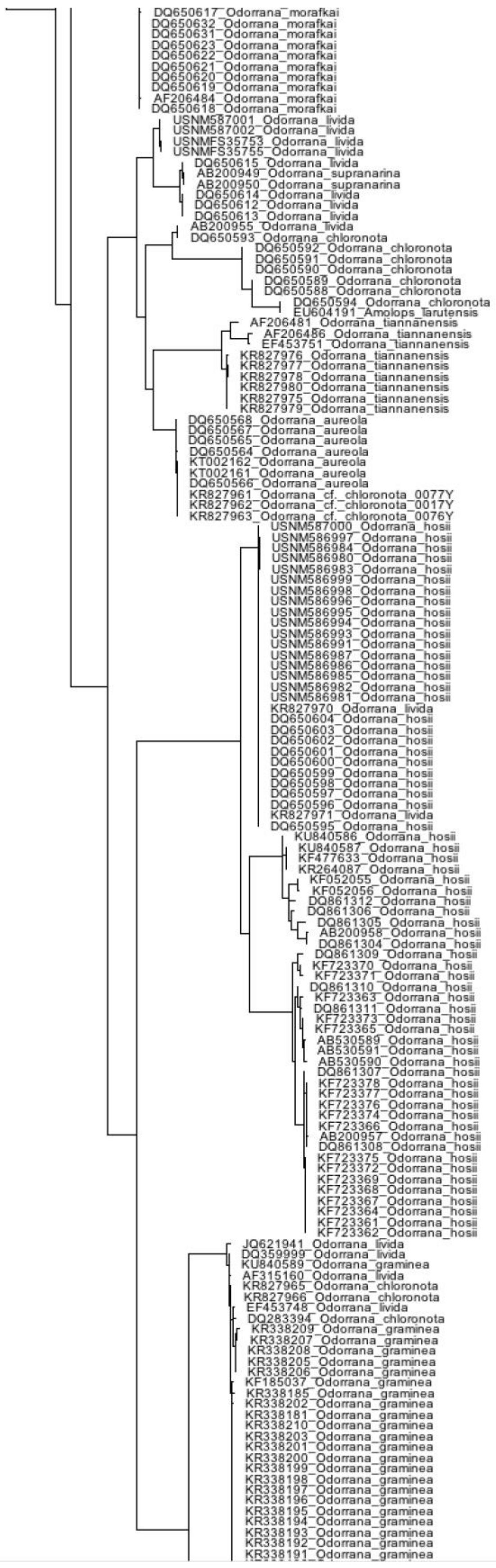
DQ650617_Odorrana_morafkai
DQ650632_Odorrana_morafkai
DQ650631_Odorrana_morafkai
DQ650623_Odorrana_morafkai
DQ650622_Odorrana_morafkai
DQ650621_Odorrana_morafkai
DQ650620_Odorrana_morafkai
DQ650619_Odorrana_morafkai
AF206484_Odorrana_morafkai
DQ650618_Odorrana_morafkai
USNM587001_Odorrana_livida
USNM587002_Odorrana_livida
USNMFS35753_Odorrana_livida
USNMFS35755_Odorrana_livida
DQ650615_Odorrana_livida
AB200949_Odorrana_supranarina
AB200950_Odorrana_supranarina
DQ650614_Odorrana_livida
DQ650612_Odorrana_livida
DQ650613_Odorrana_livida
AB200955_Odorrana_livida
DQ650593_Odorrana_chloronota
DQ650592_Odorrana_chloronota
DQ650591_Odorrana_chloronota
DQ650590_Odorrana_chloronota
DQ650589_Odorrana_chloronota
DQ650588_Odorrana_chloronota
DQ650594_Odorrana_chloronota
EU604191_Amolops_larutensis
AF206481_Odorrana_tiannanensis
AF206486_Odorrana_tiannanensis
EF453751_Odorrana_tiannanensis
KR827976_Odorrana_tiannanensis
KR827977_Odorrana_tiannanensis
KR827978_Odorrana_tiannanensis
KR827980_Odorrana_tiannanensis
KR827975_Odorrana_tiannanensis
KR827979_Odorrana_tiannanensis
DQ650568_Odorrana_aureola
DQ650567_Odorrana_aureola
DQ650565_Odorrana_aureola
DQ650564_Odorrana_aureola
KT002162_Odorrana_aureola
KT002161_Odorrana_aureola
DQ650566_Odorrana_aureola
KR827961_Odorrana_cf._chloronota_0077Y
KR827962_Odorrana_cf._chloronota_0017Y
KR827963_Odorrana_cf._chloronota_0076Y
USNM587000_Odorrana_hosii
USNM586997_Odorrana_hosii
USNM586984_Odorrana_hosii
USNM586980_Odorrana_hosii
USNM586983_Odorrana_hosii
USNM586999_Odorrana_hosii
USNM586998_Odorrana_hosii
USNM586996_Odorrana_hosii
USNM586995_Odorrana_hosii
USNM586994_Odorrana_hosii
USNM586993_Odorrana_hosii
USNM586991_Odorrana_hosii
USNM586987_Odorrana_hosii
USNM586986_Odorrana_hosii
USNM586985_Odorrana_hosii
USNM586982_Odorrana_hosii
USNM586981_Odorrana_hosii
KR827970_Odorrana_livida
DQ650604_Odorrana_hosii
DQ650603_Odorrana_hosii
DQ650602_Odorrana_hosii
DQ650601_Odorrana_hosii
DQ650600_Odorrana_hosii
DQ650599_Odorrana_hosii
DQ650598_Odorrana_hosii
DQ650597_Odorrana_hosii
DQ650596_Odorrana_hosii
KR827971_Odorrana_livida
DQ650595_Odorrana_hosii
KU840586_Odorrana_hosii
KU840587_Odorrana_hosii
KF477633_Odorrana_hosii
KR264087_Odorrana_hosii
KF052055_Odorrana_hosii
KF052056_Odorrana_hosii
DQ861312_Odorrana_hosii
DQ861306_Odorrana_hosii
DQ861305_Odorrana_hosii
AB200958_Odorrana_hosii
DQ861304_Odorrana_hosii
DQ861309_Odorrana_hosii
KF723370_Odorrana_hosii
KF723371_Odorrana_hosii
DQ861310_Odorrana_hosii
KF723363_Odorrana_hosii
DQ861311_Odorrana_hosii
KF723373_Odorrana_hosii
KF723365_Odorrana_hosii
AB530589_Odorrana_hosii
AB530591_Odorrana_hosii
AB530590_Odorrana_hosii
DQ861307_Odorrana_hosii
KF723378_Odorrana_hosii
KF723377_Odorrana_hosii
KF723376_Odorrana_hosii
KF723374_Odorrana_hosii
KF723366_Odorrana_hosii
AB200957_Odorrana_hosii
DQ861308_Odorrana_hosii
KF723375_Odorrana_hosii
KF723372_Odorrana_hosii
KF723369_Odorrana_hosii
KF723368_Odorrana_hosii
KF723367_Odorrana_hosii
KF723364_Odorrana_hosii
KF723361_Odorrana_hosii
KF723362_Odorrana_hosii
JQ621941_Odorrana_livida
DQ359999_Odorrana_livida
KU840589_Odorrana_graminea
AF315160_Odorrana_livida
KR827965_Odorrana_chloronota
KR827966_Odorrana_chloronota
EF453748_Odorrana_livida
DQ283394_Odorrana_chloronota
KR338209_Odorrana_graminea
KR338207_Odorrana_graminea
KR338208_Odorrana_graminea
KR338205_Odorrana_graminea
KR338206_Odorrana_graminea
KF185037_Odorrana_graminea
KR338185_Odorrana_graminea
KR338202_Odorrana_graminea
KR338181_Odorrana_graminea
KR338210_Odorrana_graminea
KR338203_Odorrana_graminea
KR338201_Odorrana_graminea
KR338200_Odorrana_graminea
KR338199_Odorrana_graminea
KR338198_Odorrana_graminea
KR338197_Odorrana_graminea
KR338196_Odorrana_graminea
KR338195_Odorrana_graminea
KR338194_Odorrana_graminea
KR338193_Odorrana_graminea
KR338192_Odorrana_graminea
KR338191_Odorrana_graminea

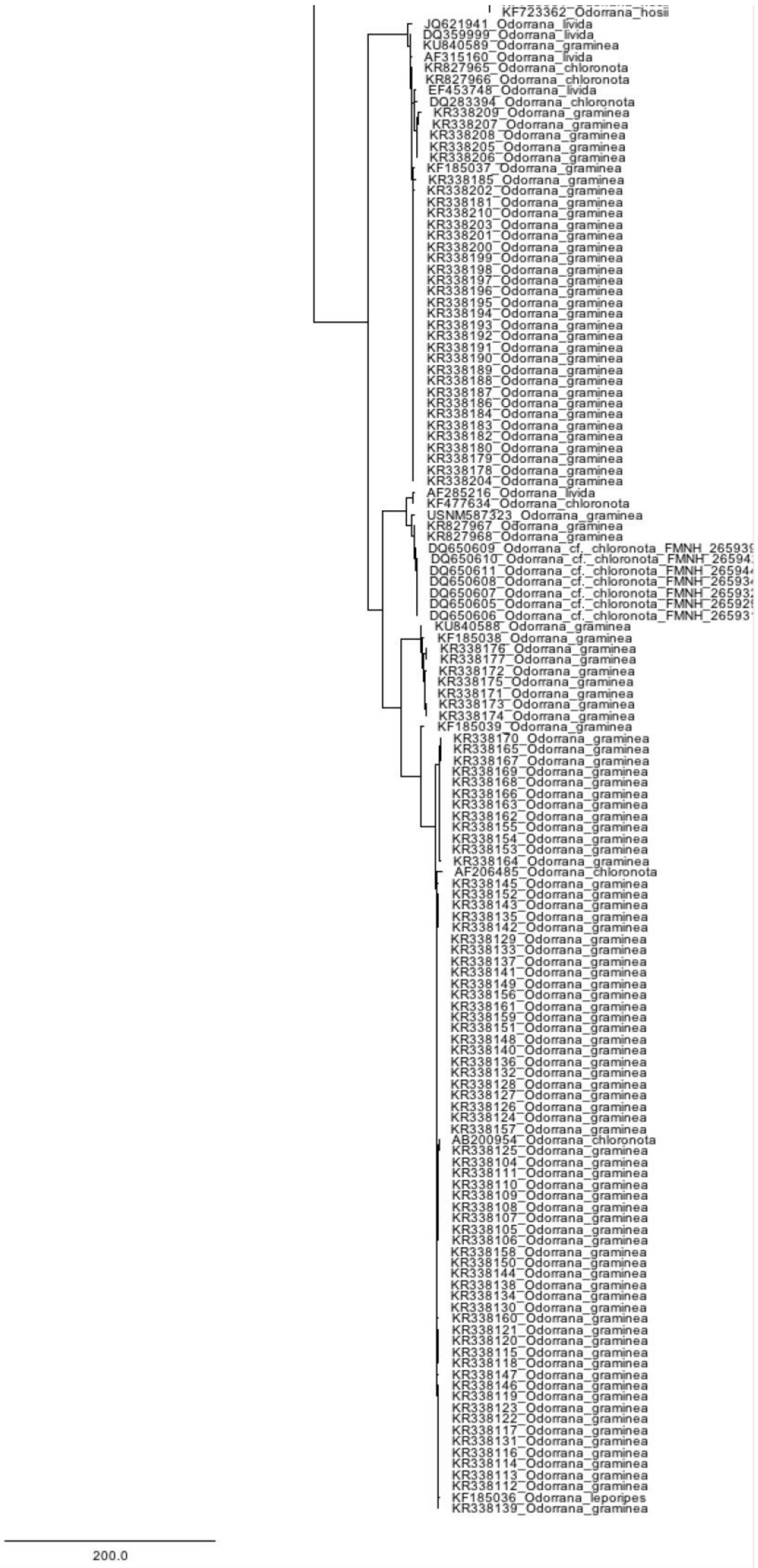

Fig. 5. Ranidae 16S neighbor-joining tree.

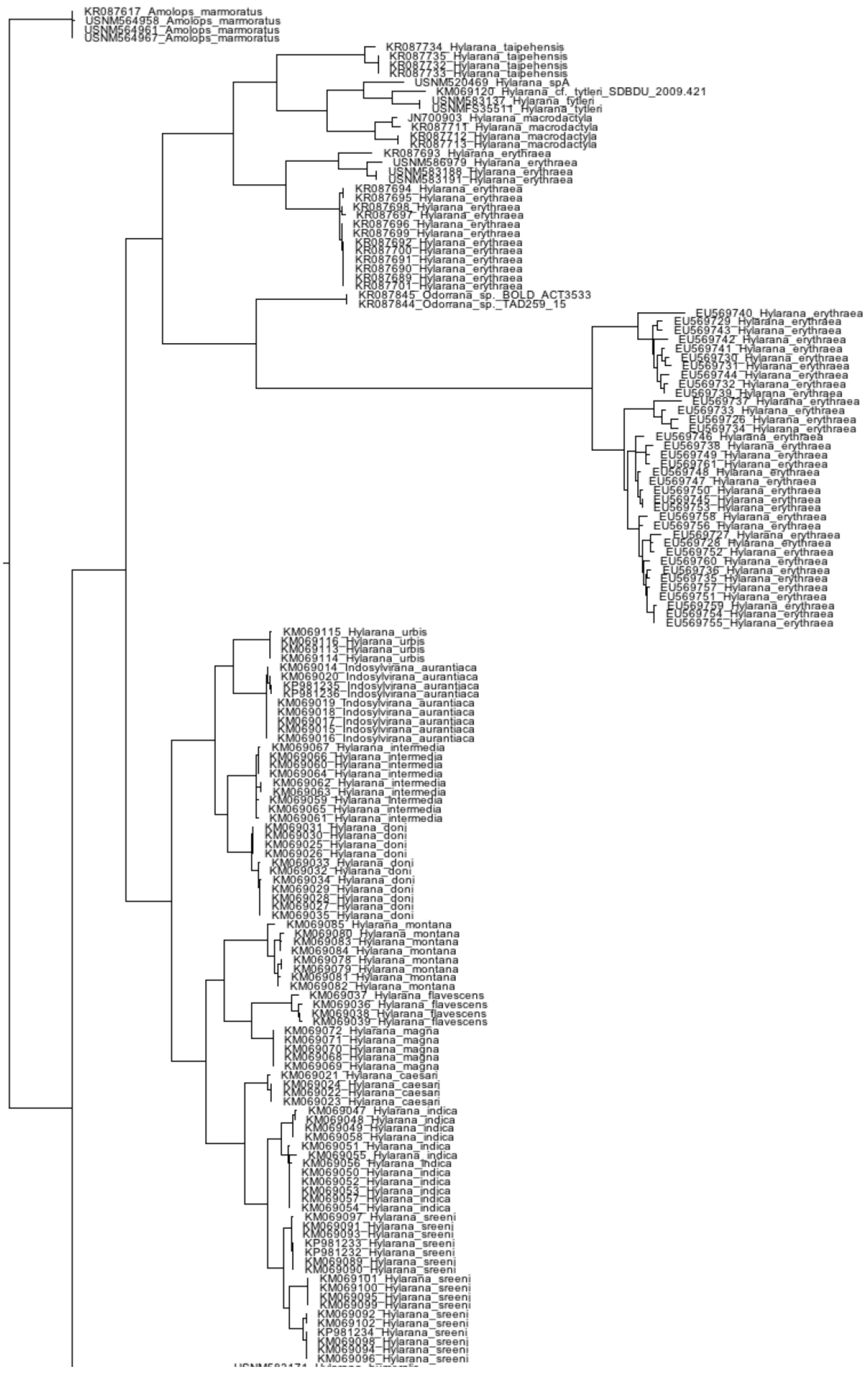
KR087617_Amolops_marmoratus
USNM564958_Amolops_marmoratus
USNM564961_Amolops_marmoratus
USNM564967_Amolops_marmoratus
KR087734_Hylarana_taipehensis
KR087735_Hylarana_taipehensis
KR087732_Hylarana_taipehensis
KR087733_Hylarana_taipehensis
USNM520469_Hylarana_spA
KM069120_Hylarana_cf._tytleri_SDBDU_2009.421
USNM583137_Hylarana_tytleri
USNMFS35511_Hylarana_tytleri
JN700903_Hylarana_macrodactyla
KR087711_Hylarana_macrodactyla
KR087712_Hylarana_macrodactyla
KR087713_Hylarana_macrodactyla
KR087693_Hylarana_erythraea
USNM586979_Hylarana_erythraea
USNM583188_Hylarana_erythraea
USNM583191_Hylarana_erythraea
KR087694_Hylarana_erythraea
KR087695_Hylarana_erythraea
KR087698_Hylarana_erythraea
KR087697_Hylarana_erythraea
KR087696_Hylarana_erythraea
KR087699_Hylarana_erythraea
KR087692_Hylarana_erythraea
KR087700_Hylarana_erythraea
KR087691_Hylarana_erythraea
KR087690_Hylarana_erythraea
KR087689_Hylarana_erythraea
KR087701_Hylarana_erythraea
KR087845_Odorrana_sp._BOLD_ACT3533
KR087844_Odorrana_sp._TAD259_15
EU569740_Hylarana_erythraea
EU569729_Hylarana_erythraea
EU569743_Hylarana_erythraea
EU569742_Hylarana_erythraea
EU569741_Hylarana_erythraea
EU569730_Hylarana_erythraea
EU569731_Hylarana_erythraea
EU569744_Hylarana_erythraea
EU569732_Hylarana_erythraea
EU569739_Hylarana_erythraea
EU569737_Hylarana_erythraea
EU569733_Hylarana_erythraea
EU569726_Hylarana_erythraea
EU569734_Hylarana_erythraea
EU569746_Hylarana_erythraea
EU569738_Hylarana_erythraea
EU569749_Hylarana_erythraea
EU569761_Hylarana_erythraea
EU569748_Hylarana_erythraea
EU569747_Hylarana_erythraea
EU569750_Hylarana_erythraea
EU569745_Hylarana_erythraea
EU569753_Hylarana_erythraea
EU569758_Hylarana_erythraea
EU569756_Hylarana_erythraea
EU569727_Hylarana_erythraea
EU569728_Hylarana_erythraea
EU569752_Hylarana_erythraea
EU569760_Hylarana_erythraea
EU569736_Hylarana_erythraea
EU569735_Hylarana_erythraea
EU569757_Hylarana_erythraea
EU569751_Hylarana_erythraea
EU569759_Hylarana_erythraea
EU569754_Hylarana_erythraea
EU569755_Hylarana_erythraea
KM069115_Hylarana_urbis
KM069116_Hylarana_urbis
KM069113_Hylarana_urbis
KM069114_Hylarana_urbis
KM069014_Indosylvirana_aurantiaca
KM069020_Indosylvirana_aurantiaca
KP981235_Indosylvirana_aurantiaca
KP981236_Indosylvirana_aurantiaca
KM069019_Indosylvirana_aurantiaca
KM069018_Indosylvirana_aurantiaca
KM069017_Indosylvirana_aurantiaca
KM069015_Indosylvirana_aurantiaca
KM069016_Indosylvirana_aurantiaca
KM069067_Hylarana_intermedia
KM069066_Hylarana_intermedia
KM069060_Hylarana_intermedia
KM069064_Hylarana_intermedia
KM069062_Hylarana_intermedia
KM069063_Hylarana_intermedia
KM069059_Hylarana_intermedia
KM069065_Hylarana_intermedia
KM069061_Hylarana_intermedia
KM069031_Hylarana_doni
KM069030_Hylarana_doni
KM069025_Hylarana_doni
KM069026_Hylarana_doni
KM069033_Hylarana_doni
KM069032_Hylarana_doni
KM069034_Hylarana_doni
KM069029_Hylarana_doni
KM069028_Hylarana_doni
KM069027_Hylarana_doni
KM069035_Hylarana_doni
KM069085_Hylarana_montana
KM069080_Hylarana_montana
KM069083_Hylarana_montana
KM069084_Hylarana_montana
KM069078_Hylarana_montana
KM069079_Hylarana_montana
KM069081_Hylarana_montana
KM069082_Hylarana_montana
KM069037_Hylarana_flavescens
KM069036_Hylarana_flavescens
KM069038_Hylarana_flavescens
KM069039_Hylarana_flavescens
KM069072_Hylarana_magna
KM069071_Hylarana_magna
KM069070_Hylarana_magna
KM069068_Hylarana_magna
KM069069_Hylarana_magna
KM069021_Hylarana_caesari
KM069024_Hylarana_caesari
KM069022_Hylarana_caesari
KM069023_Hylarana_caesari
KM069047_Hylarana_indica
KM069048_Hylarana_indica
KM069049_Hylarana_indica
KM069058_Hylarana_indica
KM069051_Hylarana_indica
KM069055_Hylarana_indica
KM069056_Hylarana_indica
KM069050_Hylarana_indica
KM069052_Hylarana_indica
KM069053_Hylarana_indica
KM069057_Hylarana_indica
KM069054_Hylarana_indica
KM069097_Hylarana_sreeni
KM069091_Hylarana_sreeni
KM069093_Hylarana_sreeni
KP981233_Hylarana_sreeni
KP981232_Hylarana_sreeni
KM069089_Hylarana_sreeni
KM069090_Hylarana_sreeni
KM069101_Hylarana_sreeni
KM069100_Hylarana_sreeni
KM069095_Hylarana_sreeni
KM069099_Hylarana_sreeni
KM069092_Hylarana_sreeni
KM069102_Hylarana_sreeni
KP981234_Hylarana_sreeni
KM069098_Hylarana_sreeni
KM069094_Hylarana_sreeni
KM069096_Hylarana_sreeni

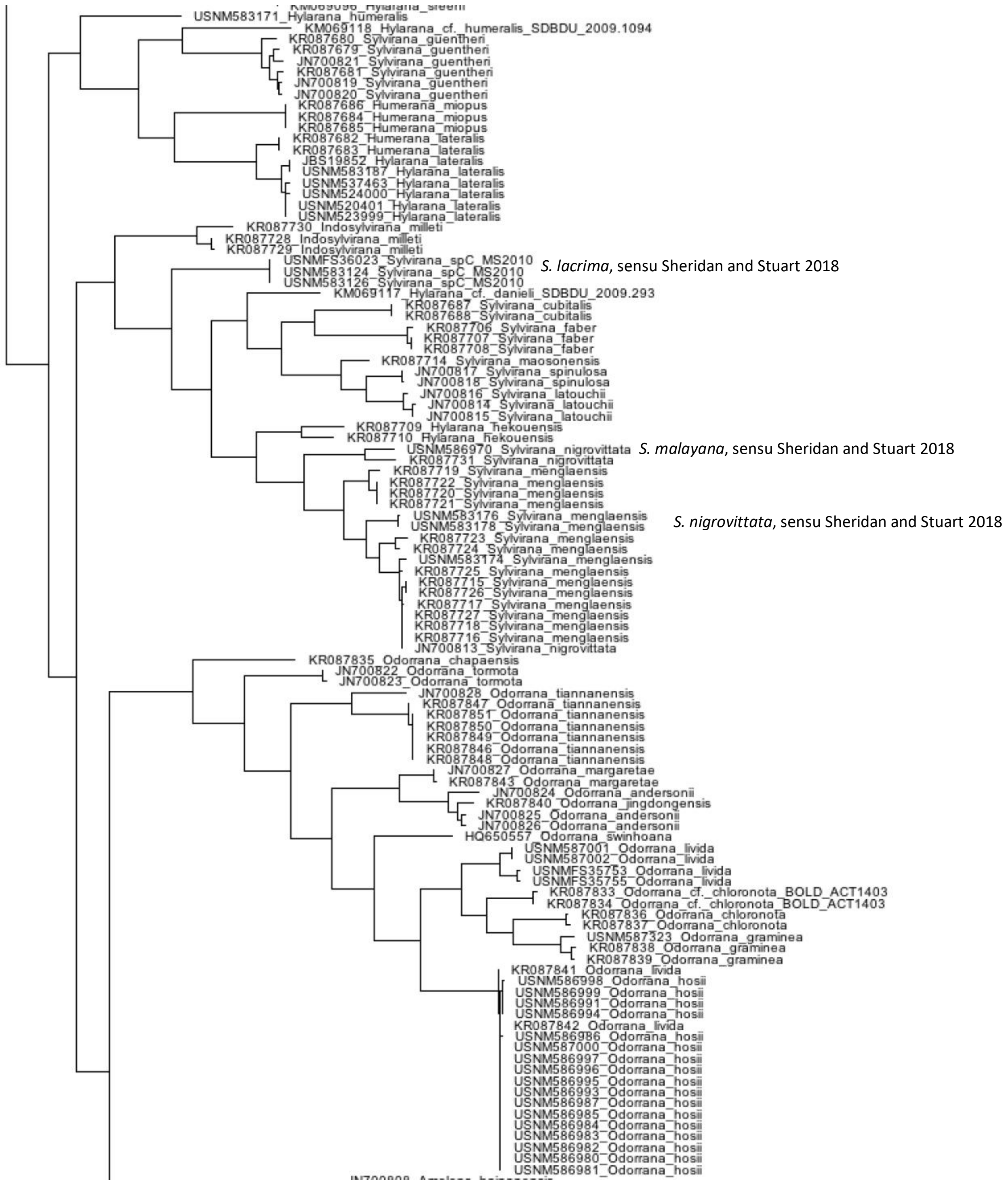
USNM583171_Hylarana_humeralis
KM069118_Hylarana_cf._humeralis_SDBDU_2009.1094
KR087680_Sylvirana_guentheri
KR087679_Sylvirana_guentheri
JN700821_Sylvirana_guentheri
KR087681_Sylvirana_guentheri
JN700819_Sylvirana_guentheri
JN700820_Sylvirana_guentheri
KR087686_Humerana_miopus
KR087684_Humerana_miopus
KR087685_Humerana_miopus
KR087682_Humerana_lateralis
KR087683_Humerana_lateralis
JBS19852_Hylarana_lateralis
USNM583187_Hylarana_lateralis
USNM537463_Hylarana_lateralis
USNM524000_Hylarana_lateralis
USNM520401_Hylarana_lateralis
USNM523999_Hylarana_lateralis
KR087730_Indosylvirana_milleti
KR087728_Indosylvirana_milleti
KR087729_Indosylvirana_milleti
USNMFS36023_Sylvirana_spC_MS2010
USNM583124_Sylvirana_spC_MS2010
USNM583126_Sylvirana_spC_MS2010
S. lacrima, sensu Sheridan and Stuart 2018
KM069117_Hylarana_cf._danieli_SDBDU_2009.293
KR087687_Sylvirana_cubitalis
KR087688_Sylvirana_cubitalis
KR087706_Sylvirana_faber
KR087707_Sylvirana_faber
KR087708_Sylvirana_faber
KR087714_Sylvirana_maosonensis
JN700817_Sylvirana_spinulosa
JN700818_Sylvirana_spinulosa
JN700816_Sylvirana_latouchii
JN700814_Sylvirana_latouchii
JN700815_Sylvirana_latouchii
KR087709_Hylarana_hekouensis
KR087710_Hylarana_hekouensis
USNM586970_Sylvirana_nigrovittata
S. malayana, sensu Sheridan and Stuart 2018
KR087731_Sylvirana_nigrovittata
KR087719_Sylvirana_menglaensis
KR087722_Sylvirana_menglaensis
KR087720_Sylvirana_menglaensis
KR087721_Sylvirana_menglaensis
USNM583176_Sylvirana_menglaensis
USNM583178_Sylvirana_menglaensis
S. nigrovittata, sensu Sheridan and Stuart 2018
KR087723_Sylvirana_menglaensis
KR087724_Sylvirana_menglaensis
USNM583174_Sylvirana_menglaensis
KR087725_Sylvirana_menglaensis
KR087715_Sylvirana_menglaensis
KR087726_Sylvirana_menglaensis
KR087717_Sylvirana_menglaensis
KR087727_Sylvirana_menglaensis
KR087718_Sylvirana_menglaensis
KR087716_Sylvirana_menglaensis
JN700813_Sylvirana_nigrovittata
KR087835_Odorrana_chapaensis
JN700822_Odorrana_tormota
JN700823_Odorrana_tormota
JN700828_Odorrana_tiannanensis
KR087847_Odorrana_tiannanensis
KR087851_Odorrana_tiannanensis
KR087850_Odorrana_tiannanensis
KR087849_Odorrana_tiannanensis
KR087846_Odorrana_tiannanensis
KR087848_Odorrana_tiannanensis
JN700827_Odorrana_margaretae
KR087843_Odorrana_margaretae
JN700824_Odorrana_andersonii
KR087840_Odorrana_jingdongensis
JN700825_Odorrana_andersonii
JN700826_Odorrana_andersonii
HQ650557_Odorrana_swinhoana
USNM587001_Odorrana_livida
USNM587002_Odorrana_livida
USNMFS35753_Odorrana_livida
USNMFS35755_Odorrana_livida
KR087833_Odorrana_cf._chloronota_BOLD_ACT1403
KR087834_Odorrana_cf._chloronota_BOLD_ACT1403
KR087836_Odorrana_chloronota
KR087837_Odorrana_chloronota
USNM587323_Odorrana_graminea
KR087838_Odorrana_graminea
KR087839_Odorrana_graminea
KR087841_Odorrana_livida
USNM586998_Odorrana_hosii
USNM586999_Odorrana_hosii
USNM586991_Odorrana_hosii
USNM586994_Odorrana_hosii
KR087842_Odorrana_livida
USNM586986_Odorrana_hosii
USNM587000_Odorrana_hosii
USNM586997_Odorrana_hosii
USNM586996_Odorrana_hosii
USNM586995_Odorrana_hosii
USNM586993_Odorrana_hosii
USNM586987_Odorrana_hosii
USNM586985_Odorrana_hosii
USNM586984_Odorrana_hosii
USNM586983_Odorrana_hosii
USNM586982_Odorrana_hosii
USNM586980_Odorrana_hosii
USNM586981_Odorrana_hosii

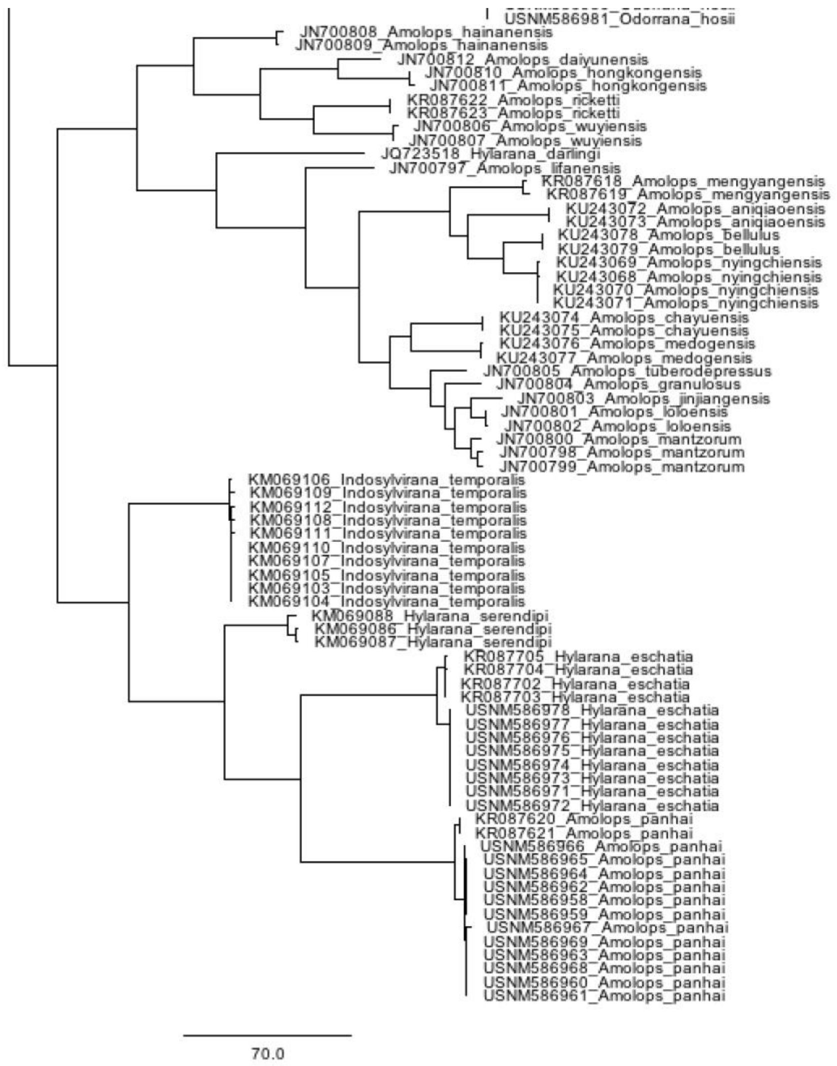

Fig. 6. Ranidae COI neighbor-joining tree.

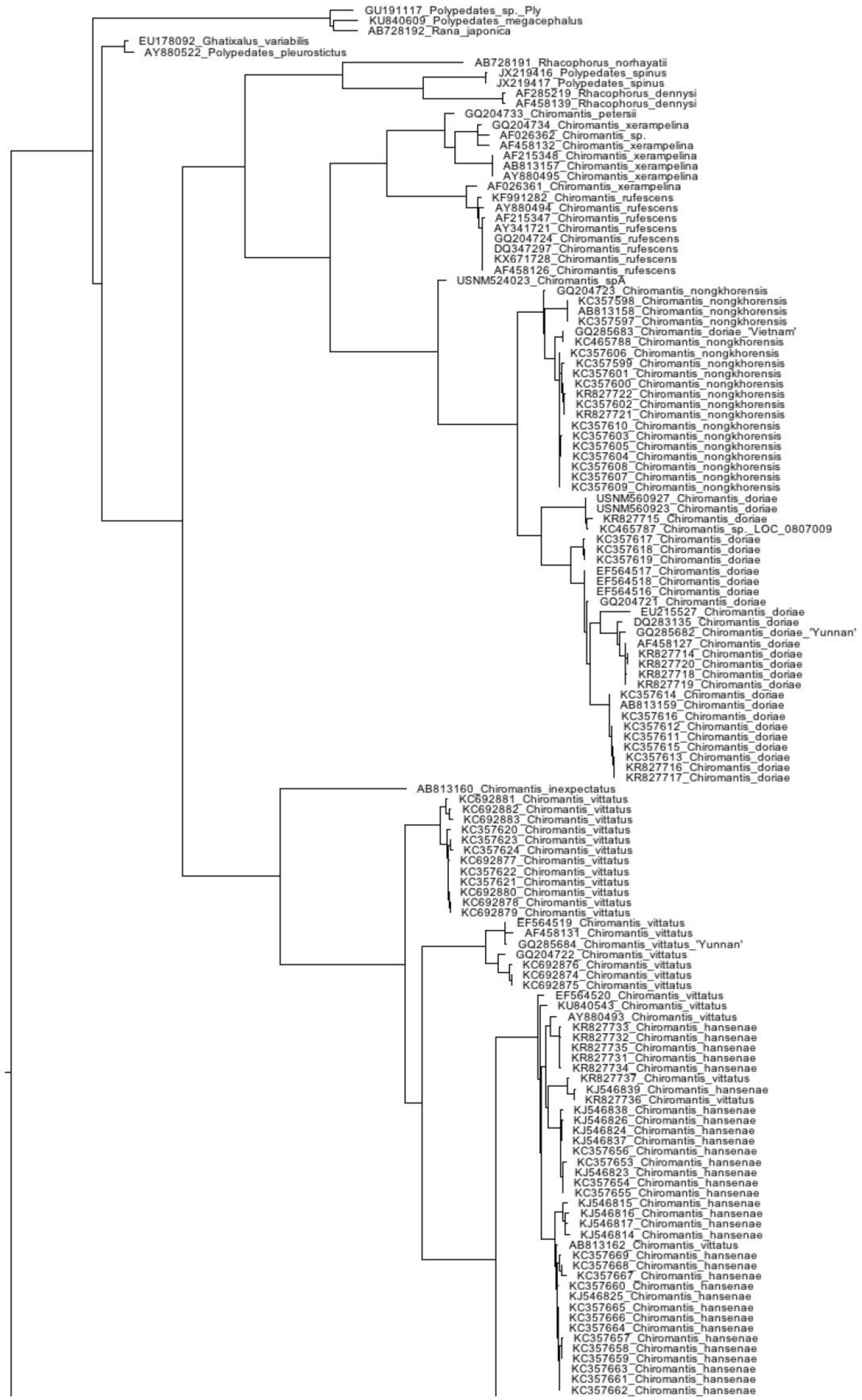
GU191117_Polypedates_sp._Ply
KU840609_Polypedates_megacephalus
AB728192_Rana_japonica
EU178092_Ghatixalus_variabilis
AY880522_Polypedates_pleurostictus
AB728191_Rhacophorus_norhayatii
JX219416_Polypedates_spinus
JX219417_Polypedates_spinus
AF285219_Rhacophorus_dennysi
AF458139_Rhacophorus_dennysi
GQ204733_Chiromantis_petersii
GQ204734_Chiromantis_xerampelina
AF026362_Chiromantis_sp.
AF458132_Chiromantis_xerampelina
AF215348_Chiromantis_xerampelina
AB813157_Chiromantis_xerampelina
AY880495_Chiromantis_xerampelina
AF026361_Chiromantis_xerampelina
KF991282_Chiromantis_rufescens
AY880494_Chiromantis_rufescens
AF215347_Chiromantis_rufescens
AY341721_Chiromantis_rufescens
GQ204724_Chiromantis_rufescens
DQ347297_Chiromantis_rufescens
KX671728_Chiromantis_rufescens
AF458126_Chiromantis_rufescens
USNM524023_Chiromantis_spA
GQ204723_Chiromantis_nongkhorensis
KC357598_Chiromantis_nongkhorensis
AB813158_Chiromantis_nongkhorensis
KC357597_Chiromantis_nongkhorensis
GQ285683_Chiromantis_doriae_'Vietnam'
KC465788_Chiromantis_nongkhorensis
KC357606_Chiromantis_nongkhorensis
KC357599_Chiromantis_nongkhorensis
KC357601_Chiromantis_nongkhorensis
KC357600_Chiromantis_nongkhorensis
KR827722_Chiromantis_nongkhorensis
KC357602_Chiromantis_nongkhorensis
KR827721_Chiromantis_nongkhorensis
KC357610_Chiromantis_nongkhorensis
KC357603_Chiromantis_nongkhorensis
KC357605_Chiromantis_nongkhorensis
KC357604_Chiromantis_nongkhorensis
KC357608_Chiromantis_nongkhorensis
KC357607_Chiromantis_nongkhorensis
KC357609_Chiromantis_nongkhorensis
USNM560927_Chiromantis_doriae
USNM560923_Chiromantis_doriae
KR827715_Chiromantis_doriae
KC465787_Chiromantis_sp._LOC_0807009
KC357617_Chiromantis_doriae
KC357618_Chiromantis_doriae
KC357619_Chiromantis_doriae
EF564517_Chiromantis_doriae
EF564518_Chiromantis_doriae
EF564516_Chiromantis_doriae
GQ204721_Chiromantis_doriae
EU215527_Chiromantis_doriae
DQ283135_Chiromantis_doriae
GQ285682_Chiromantis_doriae_'Yunnan'
AF458127_Chiromantis_doriae
KR827714_Chiromantis_doriae
KR827720_Chiromantis_doriae
KR827718_Chiromantis_doriae
KR827719_Chiromantis_doriae
KC357614_Chiromantis_doriae
AB813159_Chiromantis_doriae
KC357616_Chiromantis_doriae
KC357612_Chiromantis_doriae
KC357611_Chiromantis_doriae
KC357615_Chiromantis_doriae
KC357613_Chiromantis_doriae
KR827716_Chiromantis_doriae
KR827717_Chiromantis_doriae
AB813160_Chiromantis_inexpectatus
KC692881_Chiromantis_vittatus
KC692882_Chiromantis_vittatus
KC692883_Chiromantis_vittatus
KC357620_Chiromantis_vittatus
KC357623_Chiromantis_vittatus
KC357624_Chiromantis_vittatus
KC692877_Chiromantis_vittatus
KC357622_Chiromantis_vittatus
KC357621_Chiromantis_vittatus
KC692880_Chiromantis_vittatus
KC692878_Chiromantis_vittatus
KC692879_Chiromantis_vittatus
EF564519_Chiromantis_vittatus
AF458131_Chiromantis_vittatus
GQ285684_Chiromantis_vittatus_'Yunnan'
GQ204722_Chiromantis_vittatus
KC692876_Chiromantis_vittatus
KC692874_Chiromantis_vittatus
KC692875_Chiromantis_vittatus
EF564520_Chiromantis_vittatus
KU840543_Chiromantis_vittatus
AY880493_Chiromantis_vittatus
KR827733_Chiromantis_hansenae
KR827732_Chiromantis_hansenae
KR827735_Chiromantis_hansenae
KR827731_Chiromantis_hansenae
KR827734_Chiromantis_hansenae
KR827737_Chiromantis_vittatus
KJ546839_Chiromantis_hansenae
KR827736_Chiromantis_vittatus
KJ546838_Chiromantis_hansenae
KJ546826_Chiromantis_hansenae
KJ546824_Chiromantis_hansenae
KJ546837_Chiromantis_hansenae
KC357656_Chiromantis_hansenae
KC357653_Chiromantis_hansenae
KJ546823_Chiromantis_hansenae
KC357654_Chiromantis_hansenae
KC357655_Chiromantis_hansenae
KJ546815_Chiromantis_hansenae
KJ546816_Chiromantis_hansenae
KJ546817_Chiromantis_hansenae
KJ546814_Chiromantis_hansenae
AB813162_Chiromantis_vittatus
KC357669_Chiromantis_hansenae
KC357668_Chiromantis_hansenae
KC357667_Chiromantis_hansenae
KC357660_Chiromantis_hansenae
KJ546825_Chiromantis_hansenae
KC357665_Chiromantis_hansenae
KC357666_Chiromantis_hansenae
KC357664_Chiromantis_hansenae
KC357657_Chiromantis_hansenae
KC357658_Chiromantis_hansenae
KC357659_Chiromantis_hansenae
KC357663_Chiromantis_hansenae
KC357661_Chiromantis_hansenae
KC357662_Chiromantis_hansenae

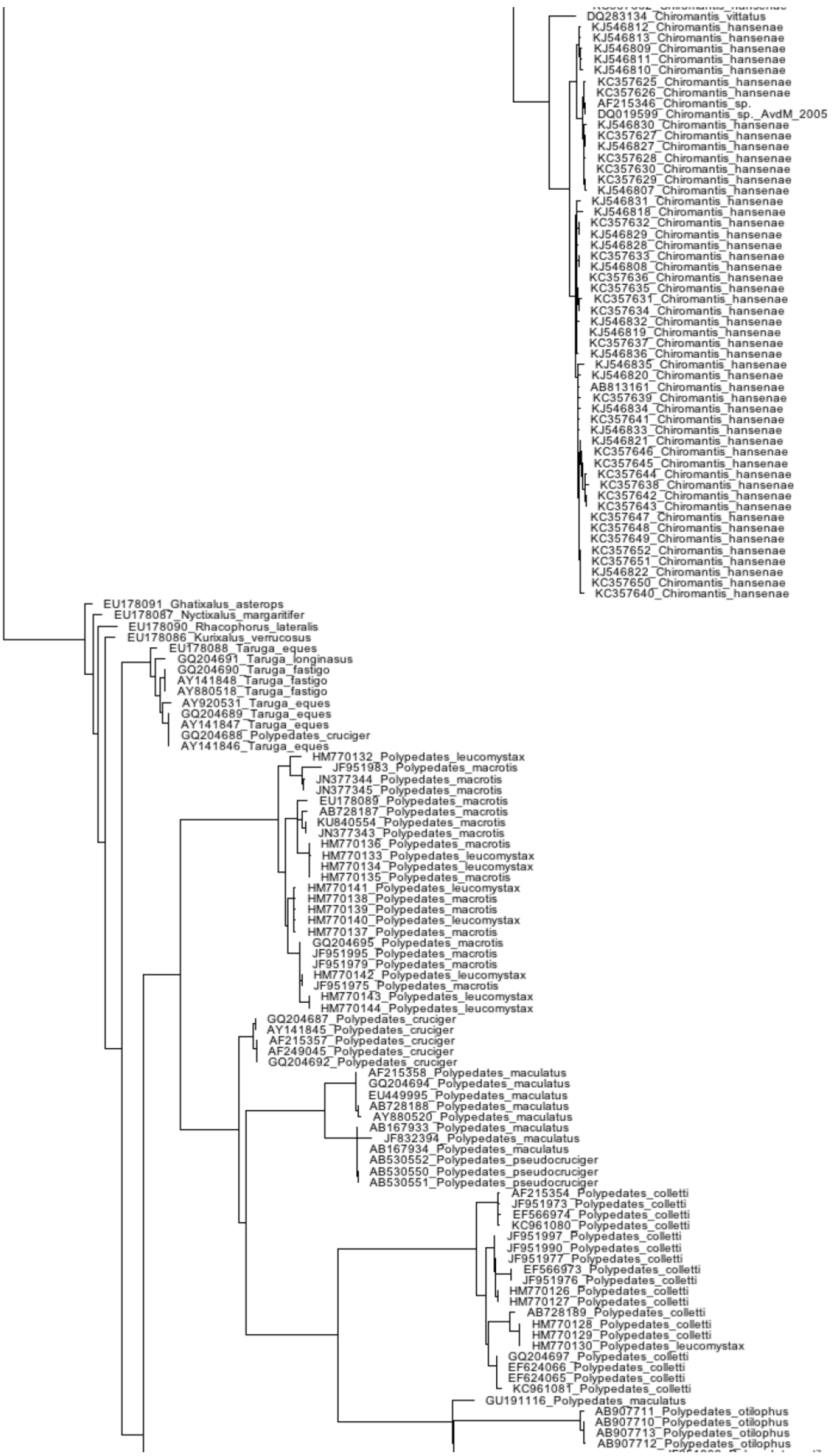

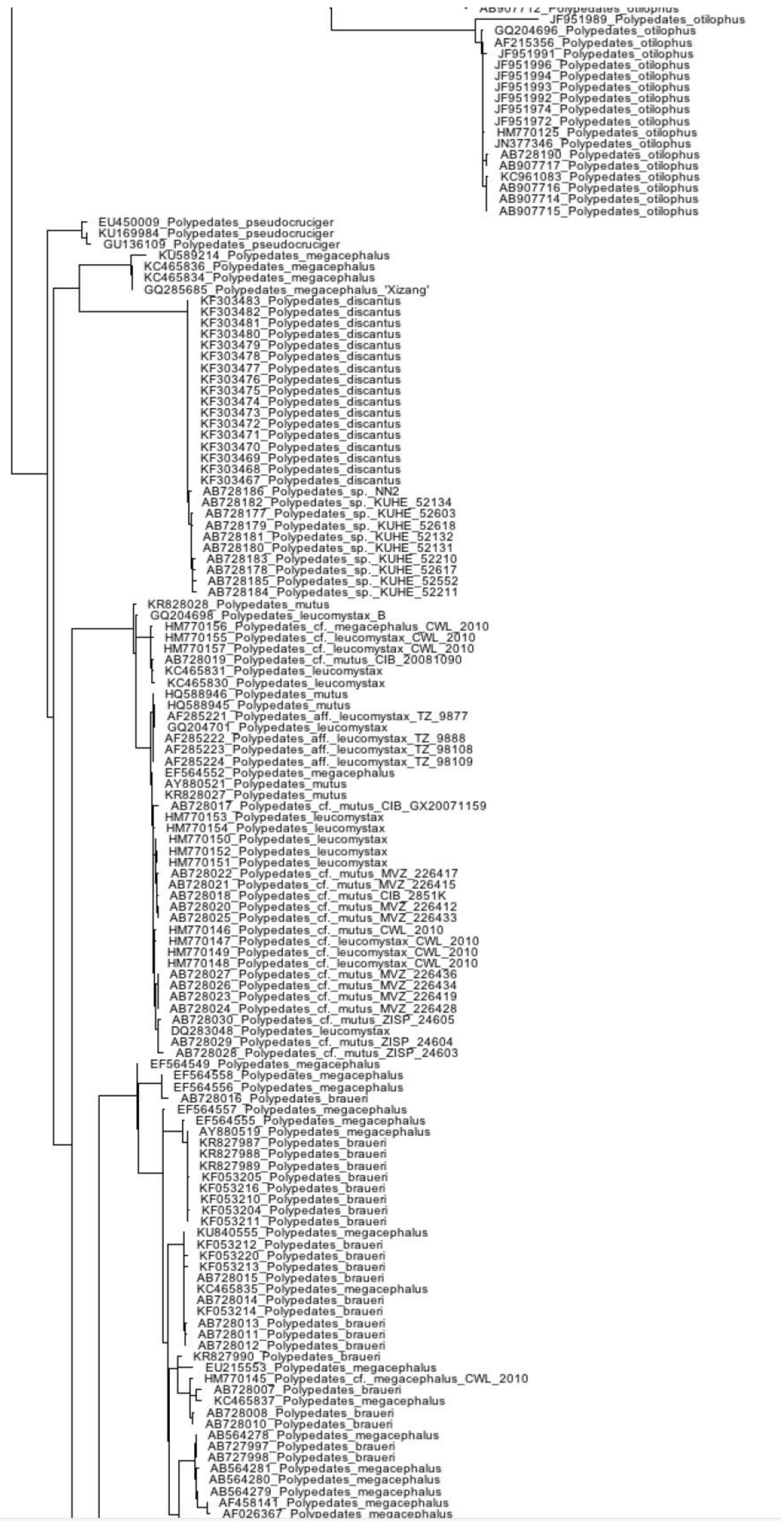

JF951989_Polypedates_otilophus
GQ204696_Polypedates_otilophus
AF215356_Polypedates_otilophus
JF951991_Polypedates_otilophus
JF951996_Polypedates_otilophus
JF951994_Polypedates_otilophus
JF951993_Polypedates_otilophus
JF951992_Polypedates_otilophus
JF951974_Polypedates_otilophus
JF951972_Polypedates_otilophus
HM770125_Polypedates_otilophus
JN377346_Polypedates_otilophus
AB728190_Polypedates_otilophus
AB907717_Polypedates_otilophus
KC961083_Polypedates_otilophus
AB907716_Polypedates_otilophus
AB907714_Polypedates_otilophus
AB907715_Polypedates_otilophus
EU450009_Polypedates_pseudocruciger
KU169984_Polypedates_pseudocruciger
GU136109_Polypedates_pseudocruciger
KU589214_Polypedates_megacephalus
KC465836_Polypedates_megacephalus
KC465834_Polypedates_megacephalus
GQ285685_Polypedates_megacephalus_'Xizang'
KF303483_Polypedates_discantus
KF303482_Polypedates_discantus
KF303481_Polypedates_discantus
KF303480_Polypedates_discantus
KF303479_Polypedates_discantus
KF303478_Polypedates_discantus
KF303477_Polypedates_discantus
KF303476_Polypedates_discantus
KF303475_Polypedates_discantus
KF303474_Polypedates_discantus
KF303473_Polypedates_discantus
KF303472_Polypedates_discantus
KF303471_Polypedates_discantus
KF303470_Polypedates_discantus
KF303469_Polypedates_discantus
KF303468_Polypedates_discantus
KF303467_Polypedates_discantus
AB728186_Polypedates_sp._NN2
AB728182_Polypedates_sp._KUHE_52134
AB728177_Polypedates_sp._KUHE_52603
AB728179_Polypedates_sp._KUHE_52618
AB728181_Polypedates_sp._KUHE_52132
AB728180_Polypedates_sp._KUHE_52131
AB728183_Polypedates_sp._KUHE_52210
AB728178_Polypedates_sp._KUHE_52617
AB728185_Polypedates_sp._KUHE_52552
AB728184_Polypedates_sp._KUHE_52211
KR828028_Polypedates_mutus
GQ204698_Polypedates_leucomystax_B
HM770156_Polypedates_cf._megacephalus_CWL_2010
HM770155_Polypedates_cf._leucomystax_CWL_2010
HM770157_Polypedates_cf._leucomystax_CWL_2010
AB728019_Polypedates_cf._mutus_CIB_20081090
KC465831_Polypedates_leucomystax
KC465830_Polypedates_leucomystax
HQ588946_Polypedates_mutus
HQ588945_Polypedates_mutus
AF285221_Polypedates_aff._leucomystax_TZ_9877
GQ204701_Polypedates_leucomystax
AF285222_Polypedates_aff._leucomystax_TZ_9888
AF285223_Polypedates_aff._leucomystax_TZ_98108
AF285224_Polypedates_aff._leucomystax_TZ_98109
EF564552_Polypedates_megacephalus
AY880521_Polypedates_mutus
KR828027_Polypedates_mutus
AB728017_Polypedates_cf._mutus_CIB_GX20071159
HM770153_Polypedates_leucomystax
HM770154_Polypedates_leucomystax
HM770150_Polypedates_leucomystax
HM770152_Polypedates_leucomystax
HM770151_Polypedates_leucomystax
AB728022_Polypedates_cf._mutus_MVZ_226417
AB728021_Polypedates_cf._mutus_MVZ_226415
AB728018_Polypedates_cf._mutus_CIB_2851K
AB728020_Polypedates_cf._mutus_MVZ_226412
AB728025_Polypedates_cf._mutus_MVZ_226433
HM770146_Polypedates_cf._mutus_CWL_2010
HM770147_Polypedates_cf._leucomystax_CWL_2010
HM770149_Polypedates_cf._leucomystax_CWL_2010
HM770148_Polypedates_cf._leucomystax_CWL_2010
AB728027_Polypedates_cf._mutus_MVZ_226436
AB728026_Polypedates_cf._mutus_MVZ_226434
AB728023_Polypedates_cf._mutus_MVZ_226419
AB728024_Polypedates_cf._mutus_MVZ_226428
AB728030_Polypedates_cf._mutus_ZISP_24605
DQ283048_Polypedates_leucomystax
AB728029_Polypedates_cf._mutus_ZISP_24604
AB728028_Polypedates_cf._mutus_ZISP_24603
EF564549_Polypedates_megacephalus
EF564558_Polypedates_megacephalus
EF564556_Polypedates_megacephalus
AB728016_Polypedates_braueri
EF564557_Polypedates_megacephalus
EF564555_Polypedates_megacephalus
AY880519_Polypedates_megacephalus
KR827987_Polypedates_braueri
KR827988_Polypedates_braueri
KR827989_Polypedates_braueri
KF053205_Polypedates_braueri
KF053216_Polypedates_braueri
KF053210_Polypedates_braueri
KF053204_Polypedates_braueri
KF053211_Polypedates_braueri
KU840555_Polypedates_megacephalus
KF053212_Polypedates_braueri
KF053220_Polypedates_braueri
KF053213_Polypedates_braueri
AB728015_Polypedates_braueri
KC465835_Polypedates_megacephalus
AB728014_Polypedates_braueri
KF053214_Polypedates_braueri
AB728013_Polypedates_braueri
AB728011_Polypedates_braueri
AB728012_Polypedates_braueri
KR827990_Polypedates_braueri
EU215553_Polypedates_megacephalus
HM770145_Polypedates_cf._megacephalus_CWL_2010
AB728007_Polypedates_braueri
KC465837_Polypedates_megacephalus
AB728008_Polypedates_braueri
AB728010_Polypedates_braueri
AB564278_Polypedates_megacephalus
AB727997_Polypedates_braueri
AB727998_Polypedates_braueri
AB564281_Polypedates_megacephalus
AB564280_Polypedates_megacephalus
AB564279_Polypedates_megacephalus
AF458141_Polypedates_megacephalus
AF026367_Polypedates_megacephalus

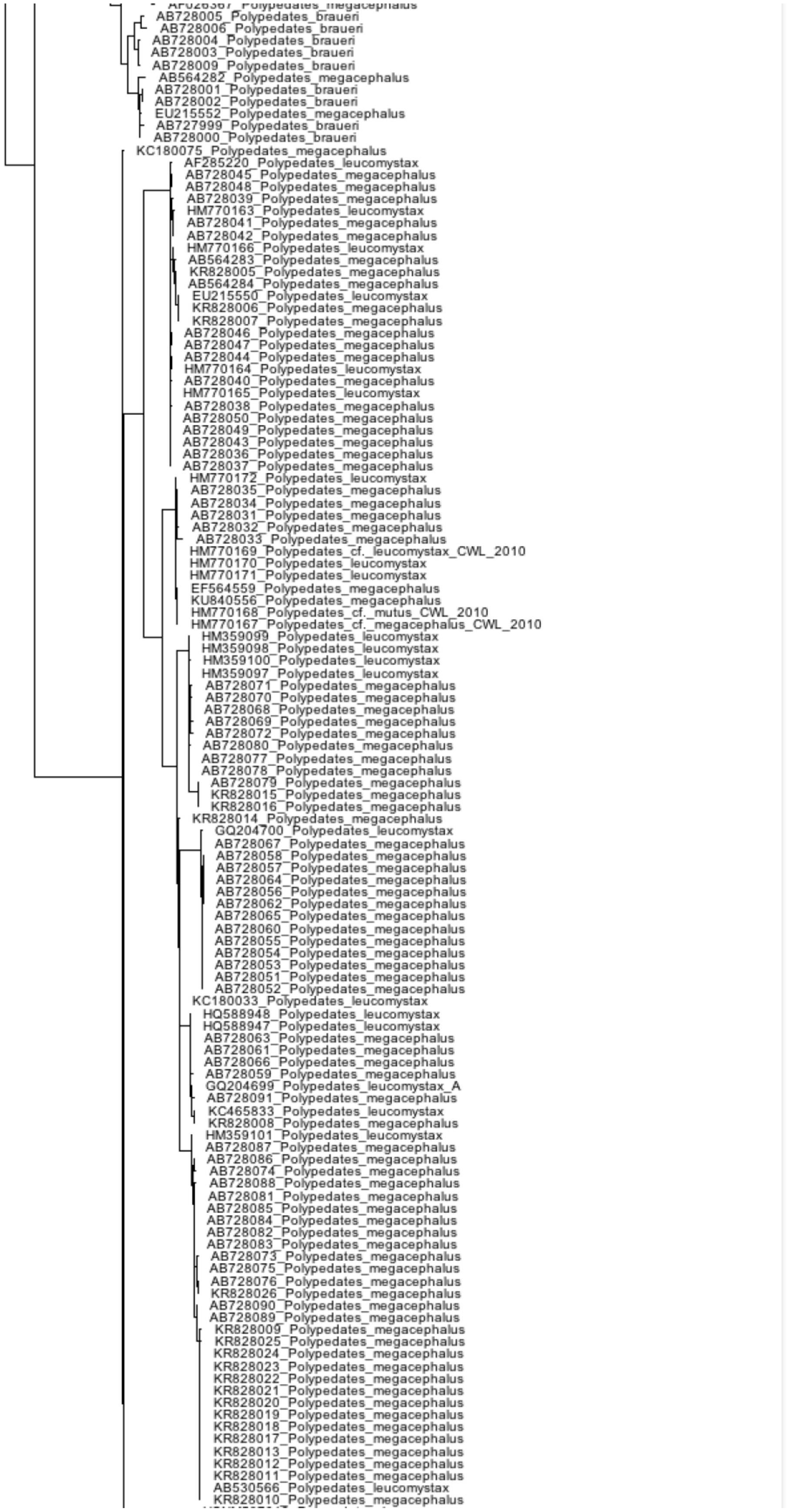
AB728005_Polypedates_braueri
AB728006_Polypedates_braueri
AB728004_Polypedates_braueri
AB728003_Polypedates_braueri
AB728009_Polypedates_braueri
AB564282_Polypedates_megacephalus
AB728001_Polypedates_braueri
AB728002_Polypedates_braueri
EU215552_Polypedates_megacephalus
AB727999_Polypedates_braueri
AB728000_Polypedates_braueri
KC180075_Polypedates_megacephalus
AF285220_Polypedates_leucomystax
AB728045_Polypedates_megacephalus
AB728048_Polypedates_megacephalus
AB728039_Polypedates_megacephalus
HM770163_Polypedates_leucomystax
AB728041_Polypedates_megacephalus
AB728042_Polypedates_megacephalus
HM770166_Polypedates_leucomystax
AB564283_Polypedates_megacephalus
KR828005_Polypedates_megacephalus
AB564284_Polypedates_megacephalus
EU215550_Polypedates_leucomystax
KR828006_Polypedates_megacephalus
KR828007_Polypedates_megacephalus
AB728046_Polypedates_megacephalus
AB728047_Polypedates_megacephalus
AB728044_Polypedates_megacephalus
HM770164_Polypedates_leucomystax
AB728040_Polypedates_megacephalus
HM770165_Polypedates_leucomystax
AB728038_Polypedates_megacephalus
AB728050_Polypedates_megacephalus
AB728049_Polypedates_megacephalus
AB728043_Polypedates_megacephalus
AB728036_Polypedates_megacephalus
AB728037_Polypedates_megacephalus
HM770172_Polypedates_leucomystax
AB728035_Polypedates_megacephalus
AB728034_Polypedates_megacephalus
AB728031_Polypedates_megacephalus
AB728032_Polypedates_megacephalus
AB728033_Polypedates_megacephalus
HM770169_Polypedates_cf._leucomystax_CWL_2010
HM770170_Polypedates_leucomystax
HM770171_Polypedates_leucomystax
EF564559_Polypedates_megacephalus
KU840556_Polypedates_megacephalus
HM770168_Polypedates_cf._mutus_CWL_2010
HM770167_Polypedates_cf._megacephalus_CWL_2010
HM359099_Polypedates_leucomystax
HM359098_Polypedates_leucomystax
HM359100_Polypedates_leucomystax
HM359097_Polypedates_leucomystax
AB728071_Polypedates_megacephalus
AB728070_Polypedates_megacephalus
AB728068_Polypedates_megacephalus
AB728069_Polypedates_megacephalus
AB728072_Polypedates_megacephalus
AB728080_Polypedates_megacephalus
AB728077_Polypedates_megacephalus
AB728078_Polypedates_megacephalus
AB728079_Polypedates_megacephalus
KR828015_Polypedates_megacephalus
KR828016_Polypedates_megacephalus
KR828014_Polypedates_megacephalus
GQ204700_Polypedates_leucomystax
AB728067_Polypedates_megacephalus
AB728058_Polypedates_megacephalus
AB728057_Polypedates_megacephalus
AB728064_Polypedates_megacephalus
AB728056_Polypedates_megacephalus
AB728062_Polypedates_megacephalus
AB728065_Polypedates_megacephalus
AB728060_Polypedates_megacephalus
AB728055_Polypedates_megacephalus
AB728054_Polypedates_megacephalus
AB728053_Polypedates_megacephalus
AB728051_Polypedates_megacephalus
AB728052_Polypedates_megacephalus
KC180033_Polypedates_leucomystax
HQ588948_Polypedates_leucomystax
HQ588947_Polypedates_leucomystax
AB728063_Polypedates_megacephalus
AB728061_Polypedates_megacephalus
AB728066_Polypedates_megacephalus
AB728059_Polypedates_megacephalus
GQ204699_Polypedates_leucomystax_A
AB728091_Polypedates_megacephalus
KC465833_Polypedates_leucomystax
KR828008_Polypedates_megacephalus
HM359101_Polypedates_leucomystax
AB728087_Polypedates_megacephalus
AB728086_Polypedates_megacephalus
AB728074_Polypedates_megacephalus
AB728088_Polypedates_megacephalus
AB728081_Polypedates_megacephalus
AB728085_Polypedates_megacephalus
AB728084_Polypedates_megacephalus
AB728082_Polypedates_megacephalus
AB728083_Polypedates_megacephalus
AB728073_Polypedates_megacephalus
AB728075_Polypedates_megacephalus
AB728076_Polypedates_megacephalus
KR828026_Polypedates_megacephalus
AB728090_Polypedates_megacephalus
AB728089_Polypedates_megacephalus
KR828009_Polypedates_megacephalus
KR828025_Polypedates_megacephalus
KR828024_Polypedates_megacephalus
KR828023_Polypedates_megacephalus
KR828022_Polypedates_megacephalus
KR828021_Polypedates_megacephalus
KR828020_Polypedates_megacephalus
KR828019_Polypedates_megacephalus
KR828018_Polypedates_megacephalus
KR828017_Polypedates_megacephalus
KR828013_Polypedates_megacephalus
KR828012_Polypedates_megacephalus
KR828011_Polypedates_megacephalus
AB530566_Polypedates_leucomystax
KR828010_Polypedates_megacephalus

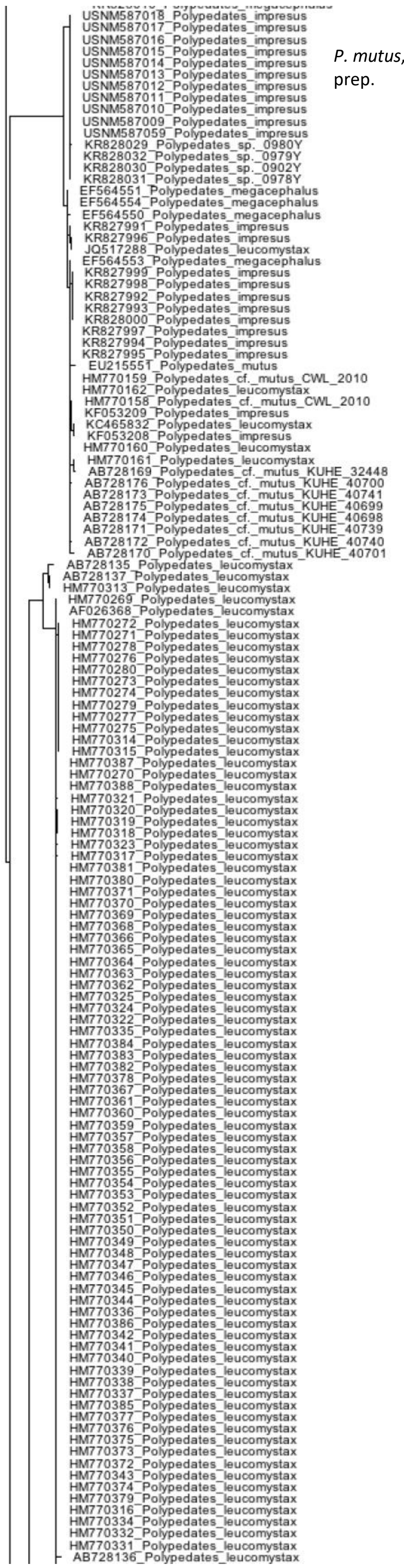

P. mutus, sensu Wilkinson, Mulcahy, Zug, in prep.

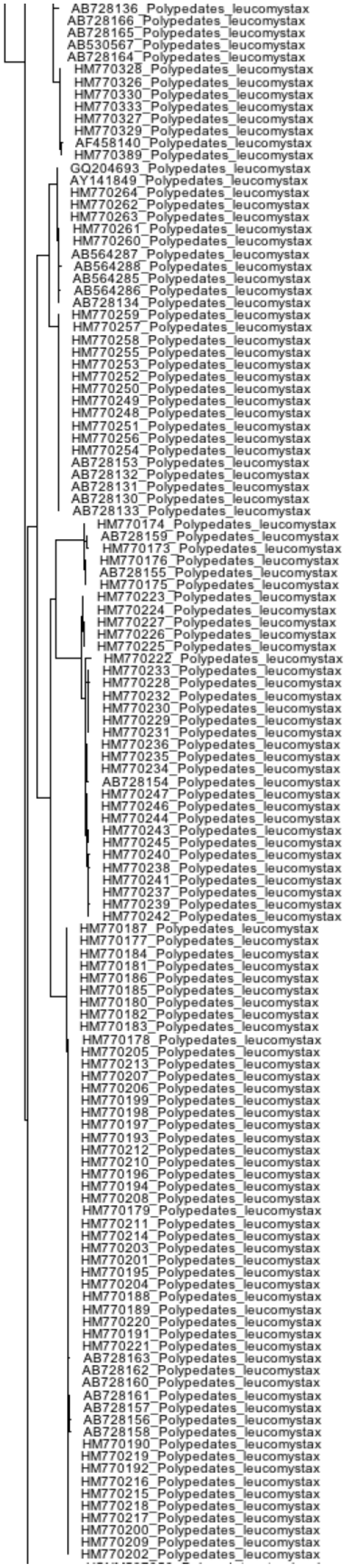
AB728136_Polypedates_leucomystax
AB728166_Polypedates_leucomystax
AB728165_Polypedates_leucomystax
AB530567_Polypedates_leucomystax
AB728164_Polypedates_leucomystax
HM770328_Polypedates_leucomystax
HM770326_Polypedates_leucomystax
HM770330_Polypedates_leucomystax
HM770333_Polypedates_leucomystax
HM770327_Polypedates_leucomystax
HM770329_Polypedates_leucomystax
AF458140_Polypedates_leucomystax
HM770389_Polypedates_leucomystax
GQ204693_Polypedates_leucomystax
AY141849_Polypedates_leucomystax
HM770264_Polypedates_leucomystax
HM770262_Polypedates_leucomystax
HM770263_Polypedates_leucomystax
HM770261_Polypedates_leucomystax
HM770260_Polypedates_leucomystax
AB564287_Polypedates_leucomystax
AB564288_Polypedates_leucomystax
AB564285_Polypedates_leucomystax
AB564286_Polypedates_leucomystax
AB728134_Polypedates_leucomystax
HM770259_Polypedates_leucomystax
HM770257_Polypedates_leucomystax
HM770258_Polypedates_leucomystax
HM770255_Polypedates_leucomystax
HM770253_Polypedates_leucomystax
HM770252_Polypedates_leucomystax
HM770250_Polypedates_leucomystax
HM770249_Polypedates_leucomystax
HM770248_Polypedates_leucomystax
HM770251_Polypedates_leucomystax
HM770256_Polypedates_leucomystax
HM770254_Polypedates_leucomystax
AB728153_Polypedates_leucomystax
AB728132_Polypedates_leucomystax
AB728131_Polypedates_leucomystax
AB728130_Polypedates_leucomystax
AB728133_Polypedates_leucomystax
HM770174_Polypedates_leucomystax
AB728159_Polypedates_leucomystax
HM770173_Polypedates_leucomystax
HM770176_Polypedates_leucomystax
AB728155_Polypedates_leucomystax
HM770175_Polypedates_leucomystax
HM770223_Polypedates_leucomystax
HM770224_Polypedates_leucomystax
HM770227_Polypedates_leucomystax
HM770226_Polypedates_leucomystax
HM770225_Polypedates_leucomystax
HM770222_Polypedates_leucomystax
HM770233_Polypedates_leucomystax
HM770228_Polypedates_leucomystax
HM770232_Polypedates_leucomystax
HM770230_Polypedates_leucomystax
HM770229_Polypedates_leucomystax
HM770231_Polypedates_leucomystax
HM770236_Polypedates_leucomystax
HM770235_Polypedates_leucomystax
HM770234_Polypedates_leucomystax
AB728154_Polypedates_leucomystax
HM770247_Polypedates_leucomystax
HM770246_Polypedates_leucomystax
HM770244_Polypedates_leucomystax
HM770243_Polypedates_leucomystax
HM770245_Polypedates_leucomystax
HM770240_Polypedates_leucomystax
HM770238_Polypedates_leucomystax
HM770241_Polypedates_leucomystax
HM770237_Polypedates_leucomystax
HM770239_Polypedates_leucomystax
HM770242_Polypedates_leucomystax
HM770187_Polypedates_leucomystax
HM770177_Polypedates_leucomystax
HM770184_Polypedates_leucomystax
HM770181_Polypedates_leucomystax
HM770186_Polypedates_leucomystax
HM770185_Polypedates_leucomystax
HM770180_Polypedates_leucomystax
HM770182_Polypedates_leucomystax
HM770183_Polypedates_leucomystax
HM770178_Polypedates_leucomystax
HM770205_Polypedates_leucomystax
HM770213_Polypedates_leucomystax
HM770207_Polypedates_leucomystax
HM770206_Polypedates_leucomystax
HM770199_Polypedates_leucomystax
HM770198_Polypedates_leucomystax
HM770197_Polypedates_leucomystax
HM770193_Polypedates_leucomystax
HM770212_Polypedates_leucomystax
HM770210_Polypedates_leucomystax
HM770196_Polypedates_leucomystax
HM770194_Polypedates_leucomystax
HM770208_Polypedates_leucomystax
HM770179_Polypedates_leucomystax
HM770211_Polypedates_leucomystax
HM770214_Polypedates_leucomystax
HM770203_Polypedates_leucomystax
HM770201_Polypedates_leucomystax
HM770195_Polypedates_leucomystax
HM770204_Polypedates_leucomystax
HM770188_Polypedates_leucomystax
HM770189_Polypedates_leucomystax
HM770220_Polypedates_leucomystax
HM770191_Polypedates_leucomystax
HM770221_Polypedates_leucomystax
AB728163_Polypedates_leucomystax
AB728162_Polypedates_leucomystax
AB728160_Polypedates_leucomystax
AB728161_Polypedates_leucomystax
AB728157_Polypedates_leucomystax
AB728156_Polypedates_leucomystax
AB728158_Polypedates_leucomystax
HM770190_Polypedates_leucomystax
HM770219_Polypedates_leucomystax
HM770192_Polypedates_leucomystax
HM770216_Polypedates_leucomystax
HM770215_Polypedates_leucomystax
HM770218_Polypedates_leucomystax
HM770217_Polypedates_leucomystax
HM770200_Polypedates_leucomystax
HM770209_Polypedates_leucomystax
HM770202_Polypedates_leucomystax

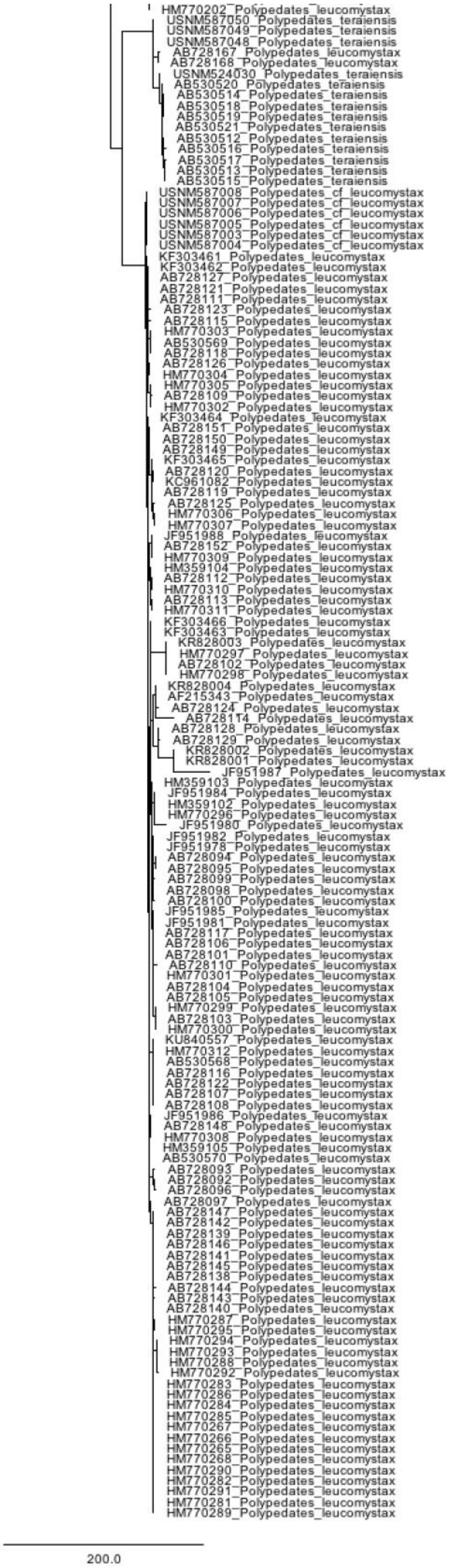

Fig. 7. Rhacophoridae 16S neighbor-joining tree.

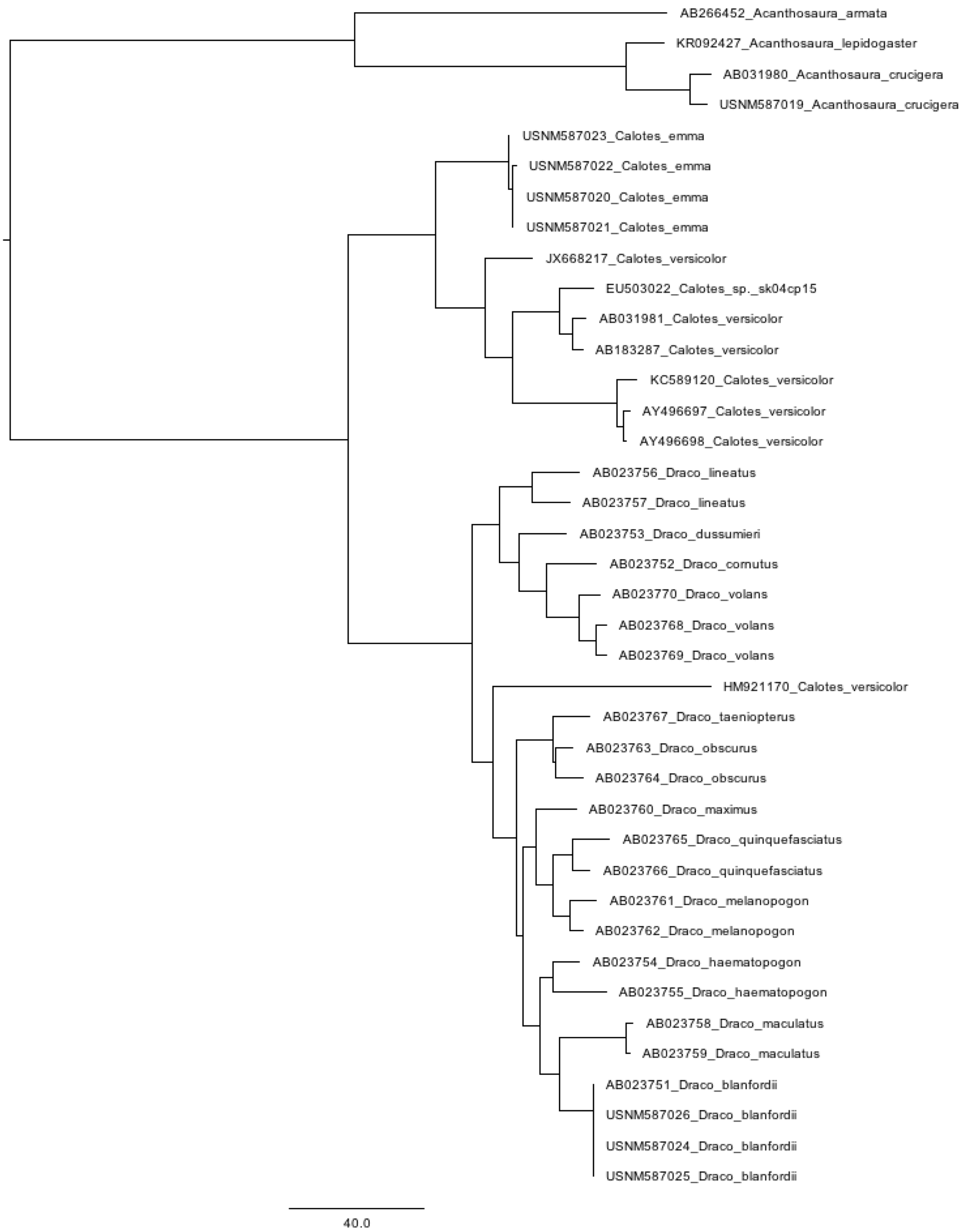

Fig. 8. Agamidae 16S neighbor-joining tree.

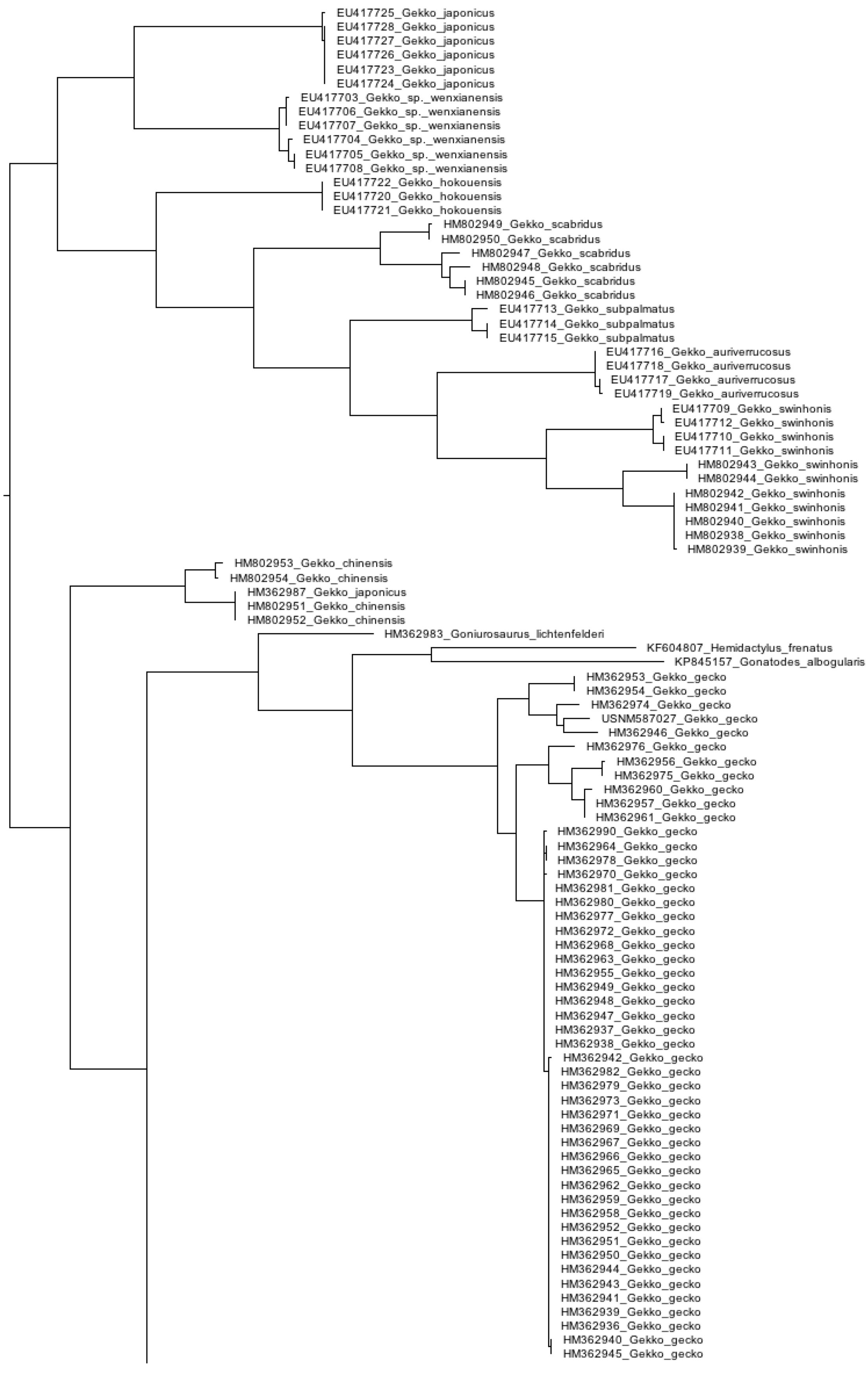

EU417725_Gekko_japonicus
EU417728_Gekko_japonicus
EU417727_Gekko_japonicus
EU417726_Gekko_japonicus
EU417723_Gekko_japonicus
EU417724_Gekko_japonicus
EU417703_Gekko_sp._wenxianensis
EU417706_Gekko_sp._wenxianensis
EU417707_Gekko_sp._wenxianensis
EU417704_Gekko_sp._wenxianensis
EU417705_Gekko_sp._wenxianensis
EU417708_Gekko_sp._wenxianensis
EU417722_Gekko_hokouensis
EU417720_Gekko_hokouensis
EU417721_Gekko_hokouensis
HM802949_Gekko_scabridus
HM802950_Gekko_scabridus
HM802947_Gekko_scabridus
HM802948_Gekko_scabridus
HM802945_Gekko_scabridus
HM802946_Gekko_scabridus
EU417713_Gekko_subpalmatus
EU417714_Gekko_subpalmatus
EU417715_Gekko_subpalmatus
EU417716_Gekko_auriverrucosus
EU417718_Gekko_auriverrucosus
EU417717_Gekko_auriverrucosus
EU417719_Gekko_auriverrucosus
EU417709_Gekko_swinhonis
EU417712_Gekko_swinhonis
EU417710_Gekko_swinhonis
EU417711_Gekko_swinhonis
HM802943_Gekko_swinhonis
HM802944_Gekko_swinhonis
HM802942_Gekko_swinhonis
HM802941_Gekko_swinhonis
HM802940_Gekko_swinhonis
HM802938_Gekko_swinhonis
HM802939_Gekko_swinhonis
HM802953_Gekko_chinensis
HM802954_Gekko_chinensis
HM362987_Gekko_japonicus
HM802951_Gekko_chinensis
HM802952_Gekko_chinensis
HM362983_Goniurosaurus_lichtenfelderi
KF604807_Hemidactylus_frenatus
KP845157_Gonatodes_albogularis
HM362953_Gekko_gecko
HM362954_Gekko_gecko
HM362974_Gekko_gecko
USNM587027_Gekko_gecko
HM362946_Gekko_gecko
HM362976_Gekko_gecko
HM362956_Gekko_gecko
HM362975_Gekko_gecko
HM362960_Gekko_gecko
HM362957_Gekko_gecko
HM362961_Gekko_gecko
HM362990_Gekko_gecko
HM362964_Gekko_gecko
HM362978_Gekko_gecko
HM362970_Gekko_gecko
HM362981_Gekko_gecko
HM362980_Gekko_gecko
HM362977_Gekko_gecko
HM362972_Gekko_gecko
HM362968_Gekko_gecko
HM362963_Gekko_gecko
HM362955_Gekko_gecko
HM362949_Gekko_gecko
HM362948_Gekko_gecko
HM362947_Gekko_gecko
HM362937_Gekko_gecko
HM362938_Gekko_gecko
HM362942_Gekko_gecko
HM362982_Gekko_gecko
HM362979_Gekko_gecko
HM362973_Gekko_gecko
HM362971_Gekko_gecko
HM362969_Gekko_gecko
HM362967_Gekko_gecko
HM362966_Gekko_gecko
HM362965_Gekko_gecko
HM362962_Gekko_gecko
HM362959_Gekko_gecko
HM362958_Gekko_gecko
HM362952_Gekko_gecko
HM362951_Gekko_gecko
HM362950_Gekko_gecko
HM362944_Gekko_gecko
HM362943_Gekko_gecko
HM362941_Gekko_gecko
HM362939_Gekko_gecko
HM362936_Gekko_gecko
HM362940_Gekko_gecko
HM362945_Gekko_gecko

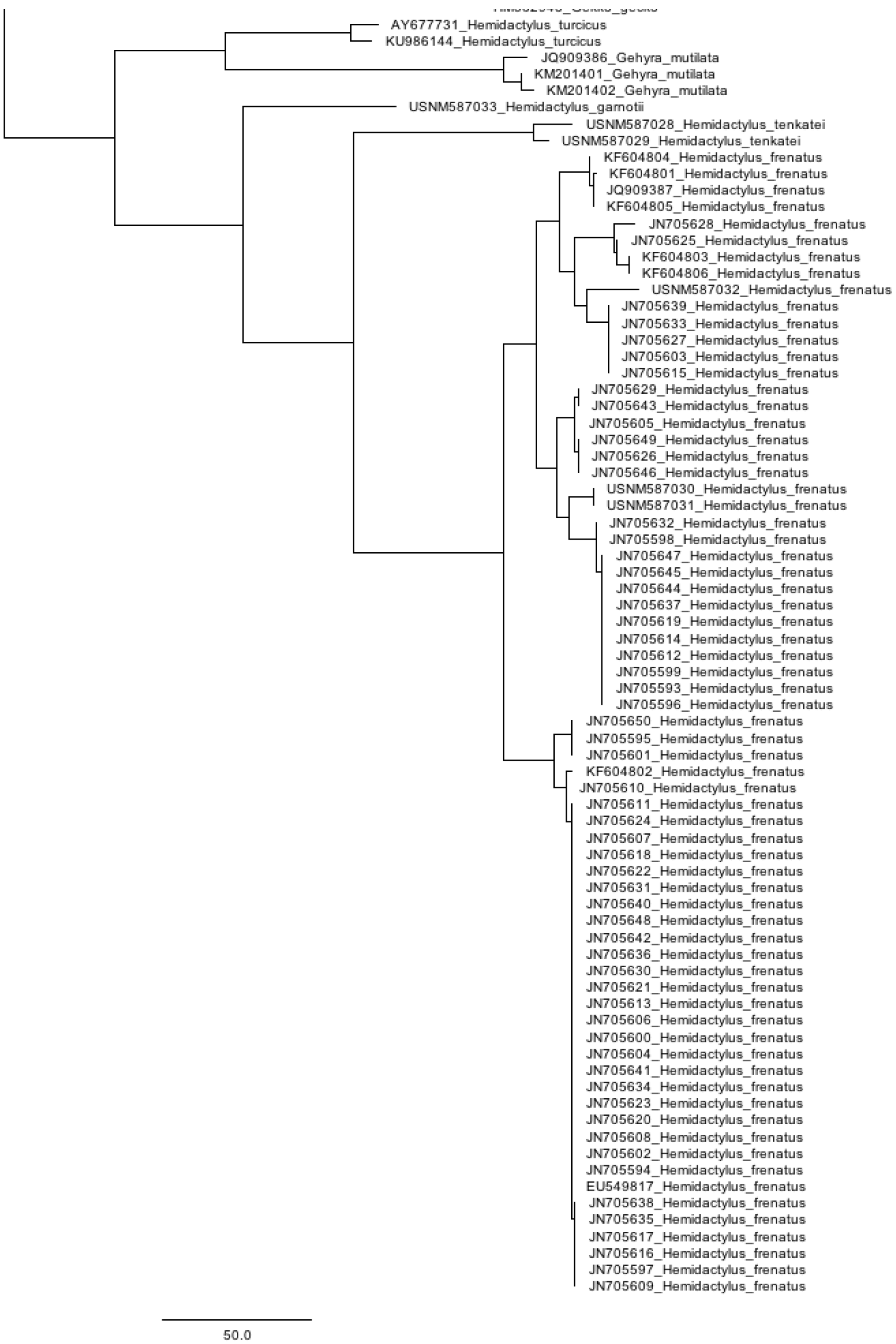

Fig. 9. Gekkonidae 16S neighbor-joining tree.

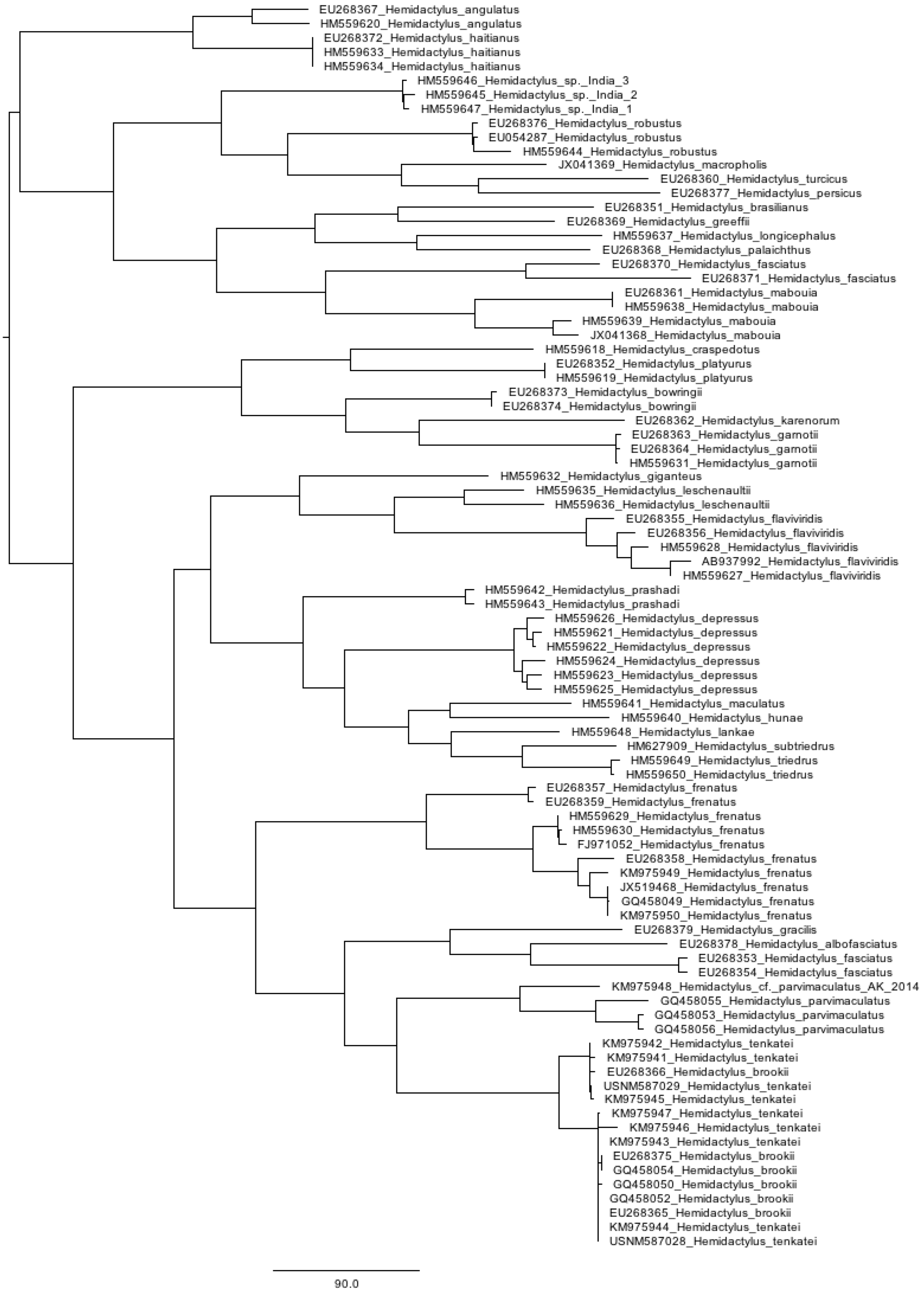

Fig. 10. Gekkonidae ND2 neighbor-joining tree.

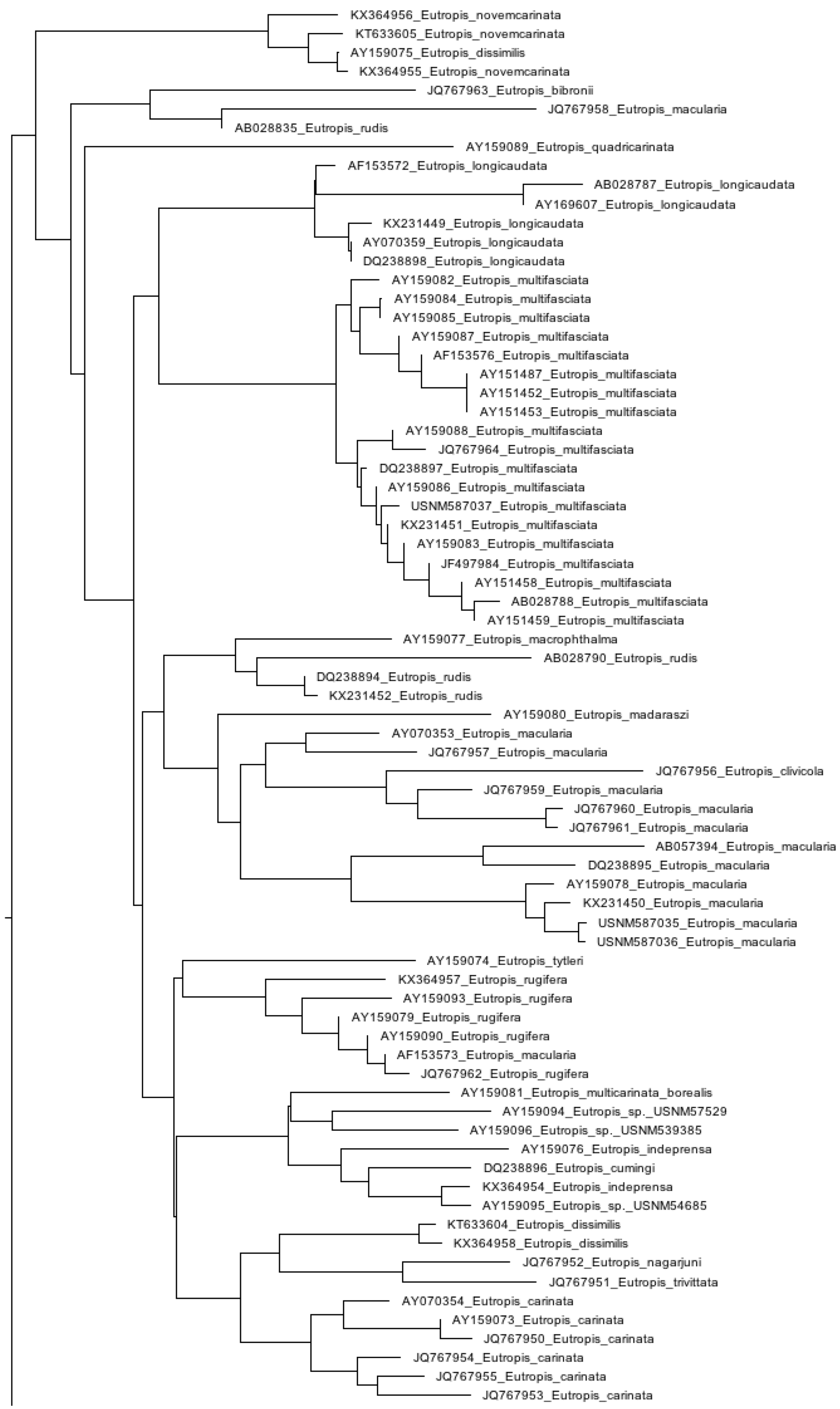
KX364956_Eutropis_novemcarinata
KT633605_Eutropis_novemcarinata
AY159075_Eutropis_dissimilis
KX364955_Eutropis_novemcarinata
JQ767963_Eutropis_bibronii
JQ767958_Eutropis_macularia
AB028835_Eutropis_rudis
AY159089_Eutropis_quadricarinata
AF153572_Eutropis_longicaudata
AB028787_Eutropis_longicaudata
AY169607_Eutropis_longicaudata
KX231449_Eutropis_longicaudata
AY070359_Eutropis_longicaudata
DQ238898_Eutropis_longicaudata
AY159082_Eutropis_multifasciata
AY159084_Eutropis_multifasciata
AY159085_Eutropis_multifasciata
AY159087_Eutropis_multifasciata
AF153576_Eutropis_multifasciata
AY151487_Eutropis_multifasciata
AY151452_Eutropis_multifasciata
AY151453_Eutropis_multifasciata
AY159088_Eutropis_multifasciata
JQ767964_Eutropis_multifasciata
DQ238897_Eutropis_multifasciata
AY159086_Eutropis_multifasciata
USNM587037_Eutropis_multifasciata
KX231451_Eutropis_multifasciata
AY159083_Eutropis_multifasciata
JF497984_Eutropis_multifasciata
AY151458_Eutropis_multifasciata
AB028788_Eutropis_multifasciata
AY151459_Eutropis_multifasciata
AY159077_Eutropis_macrophthalma
AB028790_Eutropis_rudis
DQ238894_Eutropis_rudis
KX231452_Eutropis_rudis
AY159080_Eutropis_madaraszi
AY070353_Eutropis_macularia
JQ767957_Eutropis_macularia
JQ767956_Eutropis_clivicola
JQ767959_Eutropis_macularia
JQ767960_Eutropis_macularia
JQ767961_Eutropis_macularia
AB057394_Eutropis_macularia
DQ238895_Eutropis_macularia
AY159078_Eutropis_macularia
KX231450_Eutropis_macularia
USNM587035_Eutropis_macularia
USNM587036_Eutropis_macularia
AY159074_Eutropis_tytleri
KX364957_Eutropis_rugifera
AY159093_Eutropis_rugifera
AY159079_Eutropis_rugifera
AY159090_Eutropis_rugifera
AF153573_Eutropis_macularia
JQ767962_Eutropis_rugifera
AY159081_Eutropis_multicarinata_borealis
AY159094_Eutropis_sp._USNM57529
AY159096_Eutropis_sp._USNM539385
AY159076_Eutropis_indeprensa
DQ238896_Eutropis_cumingi
KX364954_Eutropis_indeprensa
AY159095_Eutropis_sp._USNM54685
KT633604_Eutropis_dissimilis
KX364958_Eutropis_dissimilis
JQ767952_Eutropis_nagarjuni
JQ767951_Eutropis_trivittata
AY070354_Eutropis_carinata
AY159073_Eutropis_carinata
JQ767950_Eutropis_carinata
JQ767954_Eutropis_carinata
JQ767955_Eutropis_carinata
JQ767953_Eutropis_carinata

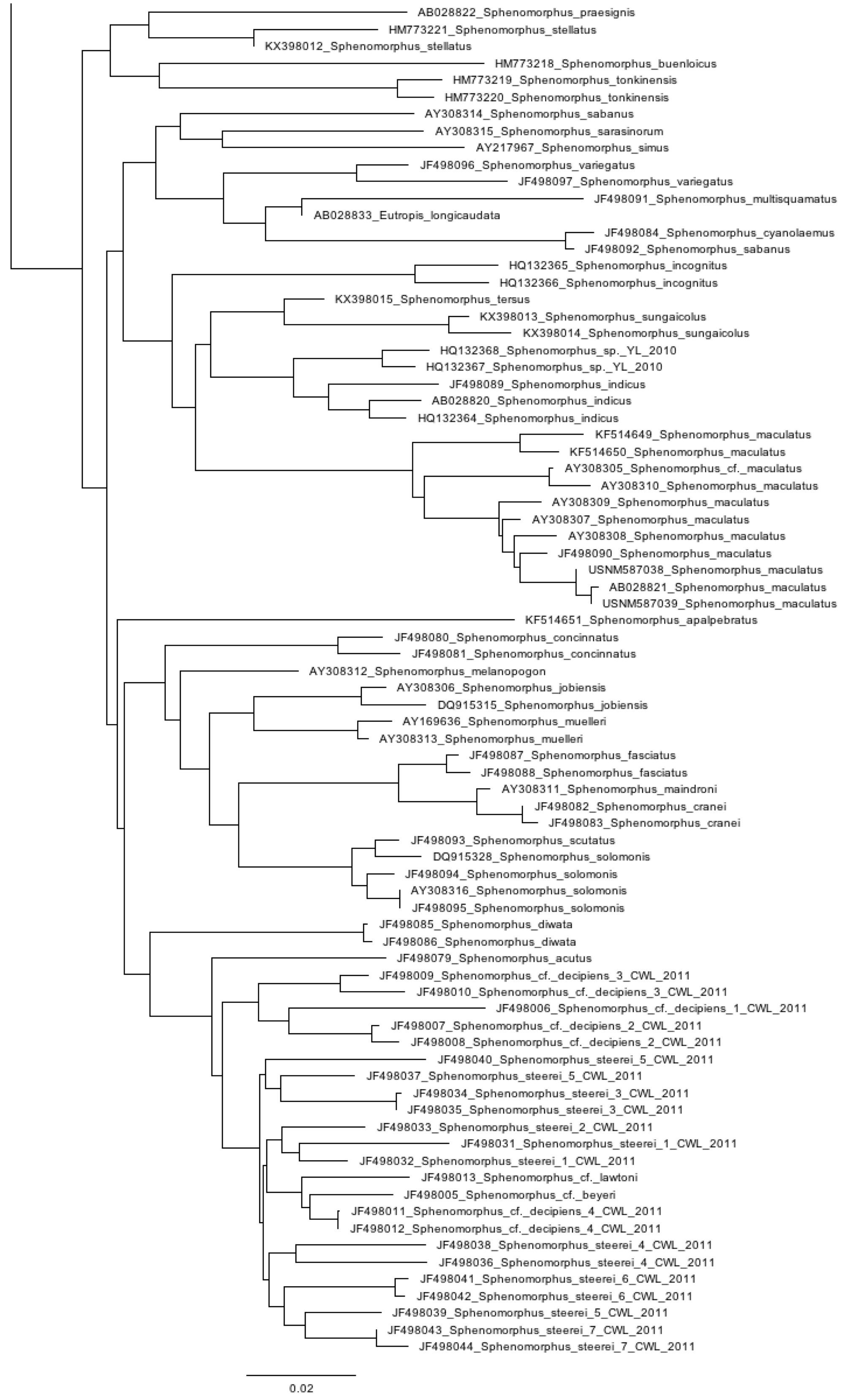

Fig. 11. Scincidae 16S neighbor-joining tree.
